# Human UPF3A and UPF3B enable fault‐tolerant activation of nonsense‐mediated mRNA decay

**DOI:** 10.15252/embj.2021109191

**Published:** 2022-04-22

**Authors:** Damaris Wallmeroth, Jan‐Wilm Lackmann, Sabrina Kueckelmann, Janine Altmüller, Christoph Dieterich, Volker Boehm, Niels H Gehring

**Affiliations:** ^1^ Institute for Genetics University of Cologne Cologne Germany; ^2^ Center for Molecular Medicine Cologne (CMMC) University of Cologne Cologne Germany; ^3^ CECAD Research Center University of Cologne Cologne Germany; ^4^ Cologne Center for Genomics (CCG) University of Cologne Cologne Germany; ^5^ Section of Bioinformatics and Systems Cardiology Department of Internal Medicine III and Klaus Tschira Institute for Integrative Computational Cardiology Heidelberg University Hospital Heidelberg Germany; ^6^ DZHK (German Centre for Cardiovascular Research) Partner site Heidelberg/Mannheim Heidelberg Germany; ^7^ Present address: Core Facility Genomics Berlin Institute of Health at Charité – Universitätsmedizin Berlin Berlin Germany; ^8^ Present address: Max Delbrück Center for Molecular Medicine in the Helmholtz Association (MDC) Berlin Germany

**Keywords:** gene paralogs, mRNA turnover, nonsense‐mediated mRNA decay, UPF3, Chromatin, Transcription & Genomics, RNA Biology

## Abstract

The paralogous human proteins UPF3A and UPF3B are involved in recognizing mRNAs targeted by nonsense‐mediated mRNA decay (NMD). UPF3B has been demonstrated to support NMD, presumably by bridging an exon junction complex (EJC) to the NMD factor UPF2. The role of UPF3A has been described either as a weak NMD activator or an NMD inhibitor. Here, we present a comprehensive functional analysis of UPF3A and UPF3B in human cells using combinatory experimental approaches. Overexpression or knockout of UPF3A as well as knockout of UPF3B did not substantially change global NMD activity. In contrast, the co‐depletion of UPF3A and UPF3B resulted in a marked NMD inhibition and a transcriptome‐wide upregulation of NMD substrates, demonstrating a functional redundancy between both NMD factors. In rescue experiments, UPF2 or EJC binding‐deficient UPF3B largely retained NMD activity. However, combinations of different mutants, including deletion of the middle domain, showed additive or synergistic effects and therefore failed to maintain NMD. Collectively, UPF3A and UPF3B emerge as fault‐tolerant, functionally redundant NMD activators in human cells.

## Graphical abstract

Co‐depletion of UPF3A and UPF3B leads to NMD inhibition and transcriptome‐wide upregulation of NMD substrates.

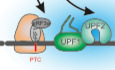

## Introduction

Precisely regulated expression of correct gene products is indispensable for eukaryotic life. This is underlined by the existence of several quality control mechanisms for gene expression, one of which is the nonsense‐mediated mRNA decay (NMD). NMD is primarily known for its ability to eliminate mature mRNAs that contain a premature termination codon (PTC). Thereby, NMD prevents the synthesis and accumulation of C‐terminally truncated proteins, which may possess undesirable and potentially disease‐causing properties (Frischmeyer & Dietz, 1999). Although the removal of PTC‐containing mRNAs was initially considered the most important function of NMD, later studies showed that NMD plays an important role in the post‐transcriptional regulation of a substantial part of the transcriptome (Lelivelt & Culbertson, 1999; He *et al*, 2003; Mendell *et al*, 2004; Rehwinkel *et al*, 2005). The importance of the factors involved in NMD is underscored by the severe impact that mutations in components of this machinery have on development in metazoans, up to causing embryonic lethality in mammals (Medghalchi *et al*, 2001; Metzstein & Krasnow, 2006; Weischenfeldt *et al*, 2008; Wittkopp *et al*, 2009; McIlwain *et al*, 2010; Hwang & Maquat, 2011; Li *et al*, 2015).

The final step of gene expression is the cytoplasmic translation of the mRNA by ribosomes. Previous studies suggested that prolonged ribosome stalling at a termination codon indicates improper translation termination and thereby triggers NMD (Amrani *et al*, 2004; Peixeiro *et al*, 2012). This could be caused by a long 3’ untranslated region (UTR) that increases the distance between the stalled ribosome and the poly(A)‐binding protein (PABPC1), which normally promotes proper translation termination (Amrani *et al*, 2004). Alternatively, NMD can also be activated by any PTC located more than 50–55 nt upstream of the 3’‐most exon–exon junction. Transcripts with such a PTC may be transcribed from mutant genes with nonsense mutations but could also be generated by defective or alternative splicing (Kervestin & Jacobson, 2012). The aforementioned 50–55 nt boundary between NMD‐activating and NMD‐resistant PTCs is determined by the RNA‐binding exon junction complex (EJC), which is deposited by the spliceosome 20–24 nt upstream of every spliced exon–exon junction (Le Hir *et al*, 2000). The EJCs remain attached on the mature mRNA during export into the cytoplasm, where they are removed by translating ribosomes or the disassembly factor PYM1 (Le Hir *et al*, 2000; Dostie & Dreyfuss, 2002). If translation terminates prematurely due to the presence of a PTC, EJCs bound downstream of the PTC serve as a marker for the NMD machinery and the initial activation of NMD (Kim *et al*, 2001; Le Hir *et al*, 2001).

Extensive research over many decades has resulted in a model for EJC‐dependent NMD. According to this model, the central factor UPF1 is bound non‐specifically to all mRNAs in the cell and is removed from the coding sequence by translating ribosomes (Hogg & Goff, 2010; Hurt *et al*, 2013; Kurosaki & Maquat, 2013; Zund *et al*, 2013). If translation terminates prematurely, UPF1 interacts with the stalled ribosome and serves as the anchoring point for the other NMD factors. According to the literature, the presence of a downstream EJC is detected by a bridge to UPF1, which is established via one of the EJC‐binding UPF3 proteins (UPF3A or UPF3B, see below) and the UPF1‐ as well as UPF3‐binding protein UPF2 (Weng *et al*, 1996; Kim *et al*, 2001; Le Hir *et al*, 2001; Chamieh *et al*, 2008). This series of interactions marks the termination codon as premature and stimulates the phosphorylation of N‐ and C‐terminal SQ motif‐containing regions of UPF1 by the kinase SMG1 (Yamashita *et al*, 2001). In its phosphorylated state UPF1 recruits the heterodimer SMG5‐SMG7 and/or SMG6, which are responsible for both exoribonucleolytic and endoribonucleolytic degradation of the mRNA, respectively (Chen & Shyu, 2003; Lejeune *et al*, 2003; Boehm *et al*, 2021). The endonuclease SMG6 cleaves the mRNA in close proximity to the PTC, resulting in two mRNA fragments (Eberle *et al*, 2009) of which the 3’ fragment is degraded by the 5’‐to‐3’ exoribonuclease XRN1 (Huntzinger *et al*, 2008; Eberle *et al*, 2009).

In vertebrates, two independent genes referred to as UPF3A and UPF3B encode UPF3 paralogs. Both of them express two major isoforms by alternative splicing, resulting in at least four different UPF3 isoforms (Lykke‐Andersen *et al*, 2000; Serin *et al*, 2001). These four human UPF3 proteins show a similar architecture and contain the same domains—with the notable exception of the UPF3A isoform lacking exon 4—but differ in details regarding their interactions to NMD‐relevant proteins. As described above, the main function of UPF3A and UPF3B is believed to physically bridge the NMD protein UPF2 to the EJC (Lykke‐Andersen *et al*, 2000; Serin *et al*, 2001; Kashima *et al*, 2006; Chamieh *et al*, 2008). Both, UPF3A and UPF3B interact via their conserved RNA recognition motif (RRM) in the N terminus with the C‐terminal MIF4G (middle portion of EIF4G) domain of UPF2 (Kadlec *et al*, 2004). In cells in which both paralogs are expressed, UPF3A and UPF3B compete for their binding partner UPF2 due to their identical mode of binding (Chan *et al*, 2009). Since UPF3A molecules that are not complexed with UFP2 are inherently unstable, high UPF3B levels impede the binding of UPF3A to UPF2 and result in a decrease of UPF3A levels. To emphasize the extent of this steady‐state UPF3 protein imbalance, a recent study using HEK293 cells estimated that although UPF3B mRNA is only 3‐fold higher expressed than UPF3A, UPF3B protein levels are 100‐fold higher than UPF3A (Cho *et al*, 2022). Conversely, when UPF3B levels are low, a larger proportion of UPF3A molecules can bind to UPF2, thereby stabilizing UPF3A. Hence, UPF3A levels are regulated by both, its paralog UPF3B, and its binding partner UPF2 (Chan *et al*, 2009). UPF3B interacts via a C‐terminal sequence referred to as EJC binding motif (EBM) with a contiguous surface formed by the EJC core components EIF4A3, MAGOH and RBM8A (Gehring *et al*, 2003; Buchwald *et al*, 2010). UPF3A also contains an EBM and could therefore in principle interact with the EJC. However, its EBM sequence binds weaker to the EJC than that of UPF3B (Kunz *et al*, 2006). In addition, UPF3A is expressed considerably less due to the above‐described competition with UPF3B. This suggests that under normal conditions, UPF3B represents the main EJC‐interacting UPF3 paralog in the cell. Recently, UPF3B, but not UPF3A, was reported to interact with the eukaryotic release factor 3a (eRF3a, official symbol: GSPT1) via the so far uncharacterized middle domain (amino acids (aa) 147–256) (Neu‐Yilik *et al*, 2017). Due to this interaction and binding of the terminating ribosome, it can delay translation termination, which is known to define aberrant termination events and trigger NMD (Amrani *et al*, 2004; Peixeiro *et al*, 2012; Neu‐Yilik *et al*, 2017).

Based on the reasons stated above, UPF3B is considered to be the major NMD‐acting UPF3 paralog in mammalian cells. Over the last 10 years, several studies have investigated the impact of reduced UPF3B expression on NMD activity with different technologies in different human or murine cell types. Using reporter systems (e.g., PTC‐containing β‐globin) in human HeLa or HEK293 cells, stabilizing effects could be observed upon UPF3B knockdown, although mostly with mild effects (Metze *et al*, 2013; Baird *et al*, 2018). Contrary to other core NMD factors, UPF3B was only ranked at position 470 in a recent NMD‐targeted siRNA screen (Baird *et al*, 2018) and not found in the top 24 hits of a CRISPR‐based forward genetic screen for NMD pathway defects (Alexandrov *et al*, 2017), suggesting that reduced UPF3B expression does not severely impair NMD activity. Analyses of RNA‐seq, microarray, and qPCR experiments from several patient‐derived lymphoblastoid cell lines showed significant upregulation of hundreds of genes, however with limiting overlap between patients (Domingo *et al*, 2020) or with previously identified NMD targets (Nguyen *et al*, 2012). Similar technologies identified varying numbers of UPF3B‐dependent NMD targets in several studies using different mouse knockout or antisense oligonucleotide (ASO)‐mediated knockdown tissues/cell lines (Jolly *et al*, 2013; Huang *et al*, 2018a, 2018b; Tan *et al*, 2020). The consensus from these studies is that UPF3B is not required for all NMD events and may rather act on a subset of targets. This phenomenon is described in the concept of “branched NMD”, which postulates that distinct branches of NMD are dependent on different NMD factors (reviewed in Yi *et al*, 2021).

However, there is an alternative explanation why depletion of UPF3B may only lead to seemingly modest effects on NMD activity. As mentioned above, the paralog UPF3A becomes more abundant when UPF3B protein levels are reduced and thus accumulating UPF3A could functionally replace UPF3B. Some early studies showed that indeed UPF3A and UPF3B both trigger degradation of a reporter construct when tethered downstream of a termination codon (Lykke‐Andersen *et al*, 2000; Gehring *et al*, 2003). The efficiency of UPF3A to elicit NMD was weaker in comparison to UPF3B, which was attributed to a weaker interaction with the EJC (Kunz *et al*, 2006). Notably, two patient‐derived lymphoblastoid cell lines with different loss‐of‐function UPF3B mutations showed differentially increased UPF3A expression, which was inversely correlated with the severity of the patients' phenotype (Nguyen *et al*, 2012). This potential functional redundancy might explain why loss of UPF3B is—in contrast to other core NMD factors (mentioned above)—not embryonically lethal in humans and mice. In addition, a number of NMD substrates were only stabilized after a combined knockdown of UPF3A and UPF3B, but not after individual knockdowns (Chan *et al*, 2009). Taken together, these observations would suggest that, at least with respect to their NMD activity, UPF3A and UPF3B serve a similar, perhaps even redundant, function. On the other hand, it was recently reported that loss/downregulation of UPF3A in different murine cell lines/types or HeLa cells results in increased transcript destabilization, and UFP3A overexpression leads to NMD inhibition (Shum *et al*, 2016). These results would rather indicate opposing functions of the two UPF3 paralogs with UPF3A being either inactive as an NMD factor or an antagonist of UPF3B and acting as an NMD inhibitor. These different observations could also be interpreted as evolutionary “subfunctionalization” of UPF3A, acting as an NMD inhibitor on some transcripts and as an NMD activator on others (Jones & Wilkinson, 2017).

In this study, we elucidate the functions and molecularly dissect the UPF3 paralogs UPF3A and UPF3B in the NMD pathway using different UPF3A and UPF3B overexpression and knockout (KO) HEK293 cell lines. We found that neither overexpression nor genomic KO of UPF3A resulted in substantial changes of NMD activity. In UPF3B KO cells, UPF3A protein levels were upregulated, but NMD activity was maintained at almost normal level. In contrast, the co‐depletion of both UPF3 paralogs resulted in a marked NMD inhibition and a global upregulation of PTC‐containing transcripts. Moreover, rescue experiments revealed that UPF3A and UPF3B proteins have additional functions besides bridging the EJC and the NMD machinery. Taken together, our data support a model of human NMD, in which UPF3A and UPF3B can replace each other and therefore perform redundant functions.

## Results

### UPF3A overexpression or knockout does not affect NMD efficiency

**Figure 1:**
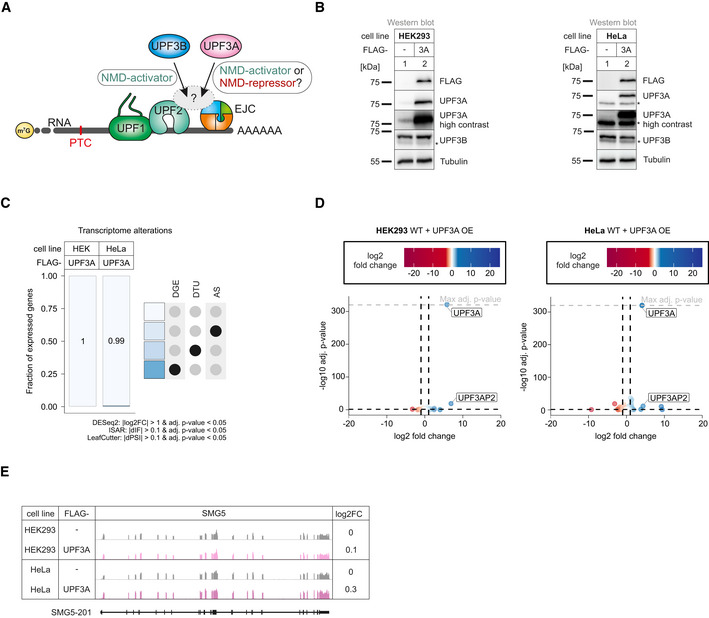
UPF3A overexpression does not affect NMD Schematic representation of the bridge between UPF1 and the EJC during NMD. Binding of UPF3A instead of the stronger bound UPF3B is discussed to either activate or repress NMD.Western blot analyses after induced expression of FLAG‐tagged UPF3A in WT HEK293 and HeLa cells (*n* = 1). Tubulin serves as control. The asterisk indicates unspecific bands.Fraction of expressed genes (genes with non‐zero counts in DESeq2) were calculated which exhibit individual or combinations of differential gene expression (DGE), differential transcript usage (DTU), and/or alternative splicing (AS) events in HEK293 and HeLa WT cells overexpressing UPF3A using the respective computational analysis (cutoffs are indicated). AS and DTU events were collapsed on the gene level. For DGE, *P*‐values were calculated by DESeq2 using a two‐sided Wald test and corrected for multiple testing using the Benjamini–Hochberg method. For DTU, *P*‐values were calculated by IsoformSwitchAnalyzeR (ISAR) using a DEXSeq‐based test and corrected for multiple testing using the Benjamini–Hochberg method. For AS, *P*‐values were calculated by LeafCutter using an asymptotic chi‐squared distribution and corrected for multiple testing using the Benjamini–Hochberg method.Volcano plot showing the differential gene expression analyses from the RNA‐Seq dataset of HEK293 and HeLa WT cells overexpressing UPF3A. The log2 fold change is plotted against the ‐log10 adjusted *P*‐value (*P*_adj_). *P*‐values were calculated by DESeq2 using a two‐sided Wald test and corrected for multiple testing using the Benjamini–Hochberg method. OE = overexpression.Read coverage of SMG5 from WT HEK293 and HeLa RNA‐seq data with or without induced UPF3A overexpression shown as Integrative Genomics Viewer (IGV) snapshot. Differential gene expression (from DESeq2) is indicated as log2 fold change (log2FC) on the right. Schematic representation of the protein coding transcript below. Source data are available online for this figure.

**Figure EV1:**
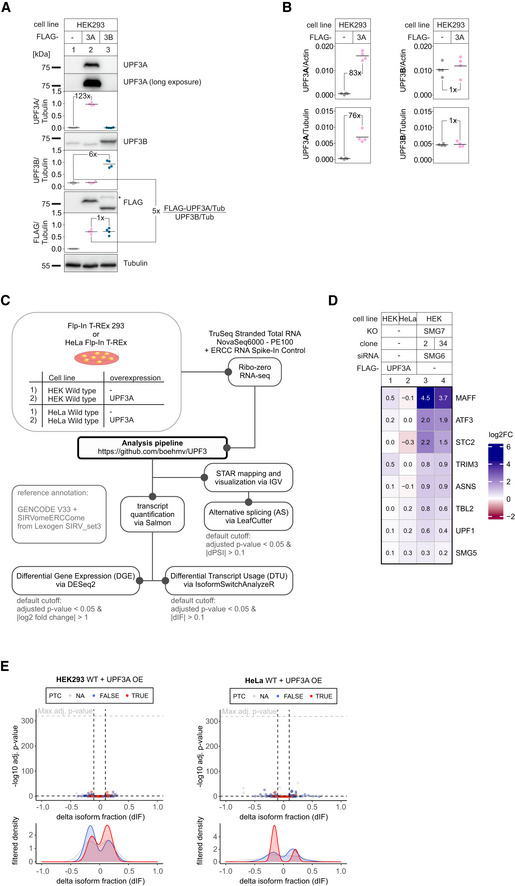
UPF3A overexpression does not cause upregulation of NMD‐targets Western blot analysis of unaltered HEK293 WT cells or with induced FLAG‐UPF3A or FLAG‐UPF3B expression. UPF3A, UPF3B, and FLAG levels were detected. Tubulin serves as control. Protein levels were quantified, normalized to tubulin expression, and shown as datapoints and mean (*n* = 4). Fold‐changes of relevant conditions are shown.Skyline analysis of WT and UPF3A‐overexpressing cells after whole proteome mass spec analysis. Quantifier intensities of UPF3A (left) and UPF3B (right) were normalized to actin (top) and tubulin (bottom) which were used as “loading controls”. Results are shown as datapoints and mean (*n* = 4). The means were used to calculate the respective fold‐changes.Schematic overview of the analysis pipeline.Heatmap of mean log2 fold changes (log2FC) of previously reported UPF3A‐responsive NMD targets (Fig 3C of Shum *et al*, 2016) as determined by DESeq2 using the indicated RNA‐Seq data. The data from SMG7 KO with SMG6 KD (Data ref: Boehm *et al*, 2021) serve as positive control for NMD inhibition.Volcano plot showing the differential transcript usage (via IsoformSwitchAnalyzeR) in RNA‐Seq data of HEK293 and HeLa WT cells overexpressing UPF3A. Isoforms containing GENCODE (release 33) annotated PTC (red, TRUE), regular stop codons (blue, FALSE) or having no annotated open reading frame (gray, NA) are indicated. The change in isoform fraction (dIF) is plotted against the ‐log10 adjusted *P*‐value (*P*_adj_). Density plots show the distribution of filtered isoforms in respect to the dIF, cutoffs were |dIF| > 0.1 and *P*_adj_ < 0.05. *P*‐values were calculated by IsoformSwitchAnalyzeR using a DEXSeq‐based test and corrected for multiple testing using the Benjamini–Hochberg method. OE = overexpression. Source data are available online for this figure.

Prior work using different mammalian models and various experimental approaches reached different conclusions regarding as to whether UPF3A is an NMD activator or repressor (Fig 1A). Therefore, we set out to re‐examine the role of UPF3A in human cells by specifically manipulating its expression levels. Compared to UPF3B, UPF3A is barely present in commonly cultured human cells under regular conditions, because it is destabilized when not bound to the interaction partner UPF2, resulting in a rapid turnover of “free” UPF3A (Chan *et al*, 2009). We hypothesized that increasing the abundance of UPF3A should lead to the stabilization of NMD targets if UPF3A is an NMD inhibitor. To test this hypothesis, we generated Flp‐In T‐REx 293 (HEK293) and Flp‐In T‐REx HeLa (HeLa) cells inducibly overexpressing FLAG‐tagged wildtype UPF3A to high protein levels (Fig 1B). Quantification of the FLAG‐UPF3A protein levels via Western blot and whole proteome mass spectrometry analysis showed an average increase of 123‐ and 80‐fold, respectively, compared to endogenous UPF3A, which was nearly undetectable in wildtype (WT) conditions (Fig EV1A and B). Western blot analyses also revealed that FLAG‐UPF3A was approximately 5‐fold higher expressed than endogenous UPF3B (Fig EV1A). We conclude that the obtained FLAG‐UPF3A expression levels should be sufficient to observe a potential NMD‐inhibitory effect. Global analysis of the transcriptome using RNA‐seq (Fig EV1C and Datasets [Link], [Link]) revealed, except for UPF3A itself, barely any significant differential gene expression (DGE), differential transcript usage (DTU) or alternative splicing (AS) events upon UPF3A overexpression compared to control conditions (Fig 1C and D, for total numbers see Appendix Fig S1A and B). Using these RNA‐seq data, we analyzed NMD targets that were previously described to be upregulated in UPF3A overexpressing HeLa cells (Shum *et al*, 2016). The DGE analysis of eight selected targets and visualization of the read coverage of the NMD substrate SMG5 showed neither in HEK293 nor in HeLa cells substantial up‐ or downregulation when UPF3A was overexpressed (Figs 1E and EV1D). Contrary to the hypothesis that overexpressed UPF3A acts as an NMD inhibitor, quantification of differential transcript usage via IsoformSwitchAnalyzeR (Vitting‐Seerup & Sandelin, 2019) could not detect an accumulation of PTC‐containing transcripts (Fig EV1E). Collectively, these analyses indicated that UPF3A overexpression in HEK293 or HeLa cells does not negatively affect NMD in particular.

**Figure 2:**
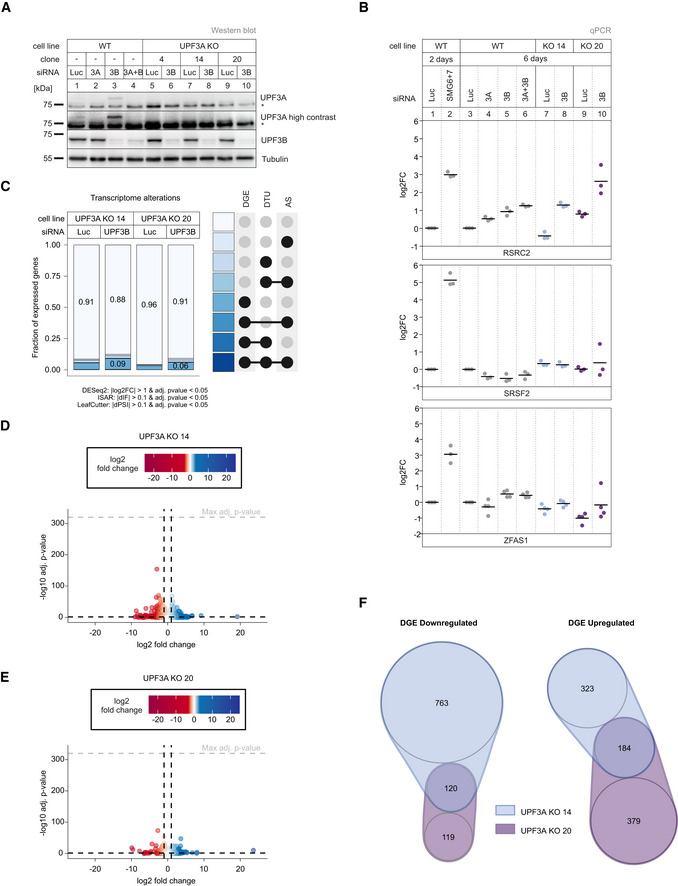
UPF3A KOs show light NMD‐independent transcriptome alterations AWestern blot analysis of WT and UPF3A KO cells (clones 4, 14, and 20) with the indicated siRNA treatments (*n* = 1). UPF3A and UPF3B protein levels were detected, Tubulin serves as control. The asterisk indicates unspecific bands.BQuantitative RT–PCR of the indicated cell lines treated with the indicated siRNAs for 2 or 6 days. For RSRC2 and SRSF2 the ratio of NMD isoform to canonical isoform was calculated. ZFAS1 expression was normalized to C1orf43 reference. Data points and means are plotted as log2 fold change (log2FC) (*n* = 3 for RSRC2 and SRSF2, *n* = 4 for ZFAS1).CFraction of expressed genes (genes with non‐zero counts in DESeq2) were calculated which exhibit individual or combinations of differential gene expression (DGE), differential transcript usage (DTU) and/or alternative splicing (AS) events in the indicated conditions using the respective computational analysis (cutoffs are indicated). AS and DTU events were collapsed on the gene level. For DGE, *P*‐values were calculated by DESeq2 using a two‐sided Wald test and corrected for multiple testing using the Benjamini–Hochberg method. For DTU, *P*‐values were calculated by IsoformSwitchAnalyzeR using a DEXSeq‐based test and corrected for multiple testing using the Benjamini–Hochberg method. For AS, *P*‐values were calculated by LeafCutter using an asymptotic chi‐squared distribution and corrected for multiple testing using the Benjamini–Hochberg method.D, EVolcano plots showing the differential gene expression analyses from the indicated RNA‐Seq datasets (D UPF3A KO clone 14, E UPF3A KO clone 20). The log2 fold change is plotted against the −log10 adjusted *P*‐value (*P*_adj_). *P*‐values were calculated by DESeq2 using a two‐sided Wald test and corrected for multiple testing using the Benjamini–Hochberg method.FnVenn Diagram showing the overlap of up‐ or downregulated genes in the UPF3A KO cell lines 14 and 20. Log2 fold change < 1 (downregulated) or > 1 (upregulated) and adjusted *P*‐value (*P*_adj_) < 0.05. DGE = Differential Gene Expression. Source data are available online for this figure.

Next, we approached the question of UPF3A function in the opposite way by generating UPF3A knockout (KO) HEK293 cell lines. Using CRISPR‐Cas9 genome editing, we isolated three clones that lacked the UPF3A‐specific band on the Western blot even after downregulation of UPF3B (Fig 2A). Two clones (14 and 20) were characterized in detail.

**Figure EV2:**
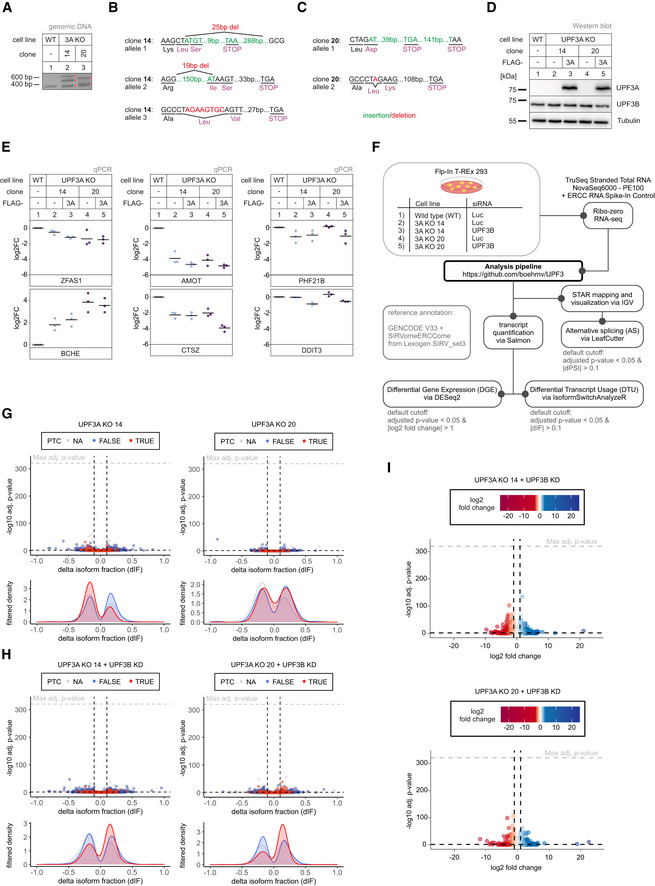
Light NMD inhibition after UPF3B KD in UPF3A KO cells APCR amplification of targeted genomic locus of UPF3A for Sanger sequencing analysis.B, CThe targeted exon region and anticipated PTC location following insertions (green) or deletions (red) are indicated for detected alleles of UPF3A in clone 14 (B) and 20 (C).DWestern blot analysis of WT and UPF3A KO cells (clones 14 and 20) with or without expression of FLAG‐tagged UPF3A rescue construct. UPF3A and UPF3B protein levels were detected, Tubulin serves as control (*n* = 1).EQuantitative RT–PCR of the samples from (D). Expression of four targets with significant DGE in both UPF3A KO clones (ZFAS1, BCHE, AMOT, CTSZ) was normalized to C1orf43 reference. For PHF21B and DDIT3, DGE analysis showed significant downregulation in both UPF3A KO clones and upregulation in SMG7 KO + SMG6 KD and UPF3 dKO cells. Expression was normalized to EMC7 reference. Data points and means are plotted as log2 fold change (log2FC) (*n* = 3).FSchematic overview of the analysis pipeline.G, HVolcano plots showing the differential transcript usage (via IsoformSwitchAnalyzeR) in various RNA‐Seq data. Isoforms containing GENCODE (release 33) annotated PTC (red, TRUE), regular stop codons (blue, FALSE) or having no annotated open reading frame (gray, NA) are indicated. The change in isoform fraction (dIF) is plotted against the ‐log10 adjusted *P*‐value (*P*_adj_). Density plots show the distribution of filtered isoforms in respect to the dIF, cutoffs were |dIF| > 0.1 and *P*_adj_ < 0.05. *P*‐values were calculated by IsoformSwitchAnalyzeR using a DEXSeq‐based test and corrected for multiple testing using the Benjamini–Hochberg method.IVolcano plots showing the differential gene expression analyses from the indicated RNA‐Seq datasets. The log2 fold change is plotted against the ‐log10 adjusted *P*‐value (*P*_adj_). *P*‐values were calculated by DESeq2 using a two‐sided Wald test and corrected for multiple testing using the Benjamini–Hochberg method. Source data are available online for this figure.

In both cell lines, the UPF3A genomic locus contained insertions and/or deletions causing frame‐shifts and eventually PTCs (Figs EV2A–C). To gain a first impression of the NMD activity in the UPF3A KO cells, the transcript levels of three known exemplary endogenous NMD targets, RSRC2, SRSF2, and ZFAS1 were determined by qPCR (Sureau *et al*, 2001; Lykke‐Andersen *et al*, 2014; Boehm *et al*, 2021). These targets represent three different classes of NMD substrates: RSRC2 mRNAs can acquire a PTC by alternative splicing, SRSF2 mRNAs can be spliced in the 3' UTR, and ZFAS1 is a non‐coding snoRNA host gene containing only a short open reading frame. WT HEK293 cells treated with SMG6 and SMG7 siRNAs were used as a positive control for severe NMD inhibition (Fig 2B) (Boehm *et al*, 2021). While the absence of UPF3A did not result in strong abundance changes of the NMD‐sensitive isoforms of SRSF2 and RSRC2 (mean log2 fold change between −0.44 and 0.79), ZFAS1 mRNA levels were slightly decreased in the UPF3A KO cells compared to WT cells (mean log2 fold change −0.42 and −1.01 for UPF3A KO clones 14 and 20, respectively; Fig 2B). However, this effect was not rescued by the (over)expression of transgenic UPF3A, indicating that it is not caused by the lack of UPF3A but rather represents random variations in gene expression or clonal effects (Fig EV2D and E). To get a complete overview of the effects of the UPF3A KO, we performed RNA‐seq of two UPF3A KO cell lines with or without an additional UPF3B knockdown (KD; Fig EV2F and Datasets [Link], [Link]). Initially, we focused on the UPF3A KO cell lines without KDs, for which the global transcriptome analysis (DGE, DTU, AS) revealed that about 4–9% of the expressed genes were altered (Fig 2C–E, for total numbers see Appendix Fig S1A and B). The observation that in the absence of UPF3A slightly more genes were downregulated than upregulated (1,002 vs. 886) could indicate that UPF3A inhibits NMD (Fig 2F). However, the majority of genes with altered expression were clone specific and only 120 genes showed downregulation in both UPF3A KO cell lines, whereas 184 genes were upregulated in both clones (Fig 2F). This low overlap between both clones made it difficult to determine high confidence UPF3A‐dependent repressed NMD targets. Nevertheless, the greater overlap of up‐ than downregulated targets would rather indicate an NMD activating role of UPF3A. Investigation of selected targets that were significantly up‐ or downregulated in both clones revealed that the changes were not rescued after UPF3A overexpression, suggesting that they are UPF3A‐independent (Fig EV2D and E). Another indication that UPF3A depletion does not generally affect NMD efficiency came from the DTU analysis. Although clone 14 showed a minor downregulation of PTC‐containing transcripts, which could indicate more active NMD, this effect was not reproducible in the second clone (Fig EV2G). In combination with the results shown in Fig 1, this strongly suggests that neither the overexpression nor the depletion of UPF3A substantially alters (negatively or positively) the efficiency of NMD. In conclusion, the data obtained in our tested cell lines argue against a role for UPF3A as a general negative NMD regulator in human cells.

### NMD is functional in the absence of UPF3B

**Figure 3:**
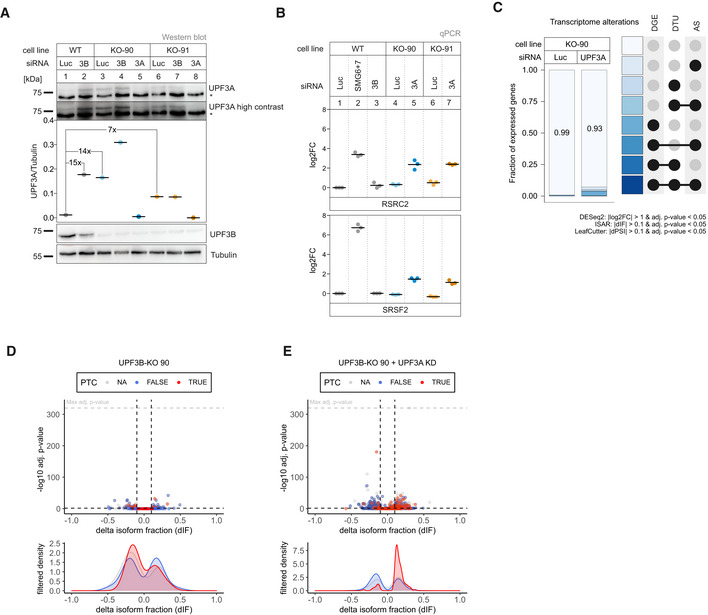
Loss of UPF3B does not affect NMD efficiency, only in combination with KD of UPF3A AWestern blot analysis of WT and UPF3B KO cells (clones 90 and 91) combined with the indicated knockdowns (*n* = 1). UPF3A and UPF3B (AK‐141) protein levels were detected, Tubulin serves as control. Protein levels of UPF3A were quantified, normalized to tubulin expression, and shown as datapoints and mean. Fold‐changes of relevant conditions are shown. The asterisk indicates unspecific bands.BQuantitative RT–PCR of the indicated cell lines with the indicated knockdowns. For RSRC2 and SRSF2 the ratio of NMD isoform to canonical isoform was calculated. Data points and means are plotted as log2 fold change (log2FC, *n* = 3).CFraction of expressed genes (genes with non‐zero counts in DESeq2) were calculated which exhibit individual or combinations of differential gene expression (DGE), differential transcript usage (DTU), and/or alternative splicing (AS) events in the indicated conditions using the respective computational analysis (cutoffs are indicated). AS and DTU events were collapsed on the gene level. For DGE, *P*‐values were calculated by DESeq2 using a two‐sided Wald test and corrected for multiple testing using the Benjamini–Hochberg method. For DTU, *P*‐values were calculated by IsoformSwitchAnalyzeR using a DEXSeq‐based test and corrected for multiple testing using the Benjamini–Hochberg method. For AS, *P*‐values were calculated by LeafCutter using an asymptotic chi‐squared distribution and corrected for multiple testing using the Benjamini–Hochberg method.D, EVolcano plots showing the differential transcript usage (via IsoformSwitchAnalyzeR) in various RNA‐Seq data. Isoforms containing GENCODE (release 33) annotated PTC (red, TRUE), regular stop codons (blue, FALSE) or having no annotated open reading frame (gray, NA) are indicated. The change in isoform fraction (dIF) is plotted against the ‐log10 adjusted *P*‐value (*P*_adj_). Density plots show the distribution of filtered isoforms in respect to the dIF, cutoffs were |dIF| > 0.1 and *P*_adj_ < 0.05. *P*‐values were calculated by IsoformSwitchAnalyzeR using a DEXSeq‐based test and corrected for multiple testing using the Benjamini–Hochberg method. Source data are available online for this figure.

**Figure EV3:**
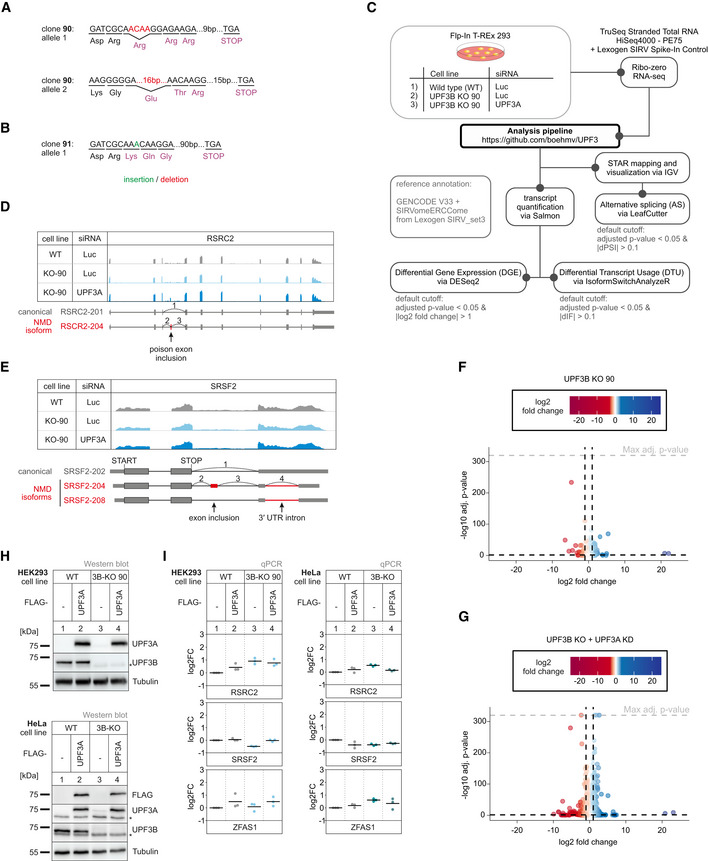
No NMD inhibition by UPF3A in the absence of UPF3B A, BThe targeted exon region and anticipated PTC location following insertions (green) or deletions (red) are indicated for detected alleles of UPF3B in KO clone 90 (A) and 91 (B).CSchematic overview of the analysis pipeline.D, ERead coverage of RSRC2 (D) and SRSF2 (E) from the indicated RNA‐seq sample data with or without UPF3A siRNA treatment shown as Integrative Genomics Viewer (IGV) snapshot. The canonical and NMD‐sensitive isoforms are schematically indicated below.F, GVolcano plots showing the differential gene expression analyses from the indicated RNA‐Seq datasets. The log2 fold change is plotted against the ‐log10 adjusted *P*‐value (*P*_adj_). *P*‐values were calculated by DESeq2 using a two‐sided Wald test and corrected for multiple testing using the Benjamini–Hochberg method.HWestern blot analysis of HEK293 and HeLa WT and UPF3B KO cells (clone 90 for HEK293 cells) with or without expression of FLAG‐tagged UPF3A rescue construct (*n* = 1). UPF3A, UPF3B and FLAG protein levels were detected, Tubulin serves as control. The asterisk indicates unspecific bands.IQuantitative RT–PCR of the HEK293 and HeLa samples from (H). For RSRC2 and SRSF2 the ratio of NMD isoform to canonical isoform was calculated. ZFAS1 expression was normalized to C1orf43 reference. Data points and means are plotted as log2 fold change (log2FC) (*n* = 3). Source data are available online for this figure.

Next, we investigated the RNA‐seq data of UPF3B knockdowns in the UPF3A KO cells (Datasets [Link], [Link]). We observed that this combination resulted in more transcriptome alterations and a mild increase of PTC‐containing isoforms (Figs 2B and C, and EV2H and I, for total numbers see Appendix Fig S1A and B). Although the UPF3B KD alone could be in principle responsible for this effect, the results could also be an indicator for redundant functions of the two UPF3 paralogs. To explore this hypothesis, we decided to generate UPF3B KOs in Flp‐In T‐REx 293 (HEK293; Figs 3A and EV3A and B) and Flp‐In T‐REx HeLa (HeLa; Fig EV3H) cells. Western blot analysis demonstrated that a UPF3B KD is less efficient in reducing the produced protein than the UPF3B KO in the two HEK293 clones designated as 90 and 91 (Fig 3A). In addition, we observed a 7‐ to 15‐fold upregulation of UPF3A after depletion (KO) or reduction (KD) of UPF3B, as described before (Chan *et al*, 2009). In the absence of UPF3B, only very moderate changes in the expression of the respective NMD‐sensitive isoforms of the NMD‐targets RSRC2 and SRSF2 were observed (Fig 3B). Comparably weak effects were detected for the HeLa UPF3B KO (|log2 fold change| < 1, Fig EV3I). This indicates that either UPF3B is not essential for NMD or that another protein is able to compensate for its loss. The most obvious candidate for this function is its own paralog UPF3A, which was also suggested previously to functionally replace UPF3B in NMD. Indeed, knocking down UPF3A in the UPF3B KO cells resulted in the increase of NMD‐sensitive RSRC2 and SRSF2 isoforms (Fig 3B). Of note, the combination of the UPF3B KO with UPF3A KD showed stronger effects than the previously analyzed UPF3A KO plus UPF3B KD. This is probably caused by the lower KD efficiency of the UPF3B siRNAs which can be observed by comparing the respective protein levels (Fig 2A vs. Fig 3A). We suspect that the remaining UPF3B levels after siRNA‐mediated UPF3B KD still support NMD.

To gain more transcriptome‐wide information, we performed RNA‐seq of the UPF3B KO clone 90, with and without UPF3A siRNA treatment (Fig EV3C and Datasets [Link], [Link]). The global effects detected in the RNA‐seq data correlated well with the NMD inhibition seen for single targets (Figs 3C and EV3D–G, for total numbers, see Appendix Fig S1A and B). Overall, the number of differentially expressed genes increased from 135 in the UPF3B KO cells to 932 upon additional UPF3A KD. More specific investigation of NMD‐relevant events by analysis of the differential transcript usage revealed a marked upregulation of transcripts annotated with a PTC only in the UPF3B KO cells with additionally downregulated UPF3A (Fig 3D and E). This increase of NMD‐sensitive transcripts could not be observed in the UPF3B KO cells with UPF3A naturally upregulated, indicating that the loss of UPF3B has minimal impact on NMD activity when UPF3A is present. All together these data strongly suggested at least partial redundancy of UPF3A and UPF3B, since KO of only one paralog was largely irrelevant for NMD functionality. Furthermore, we were able to show that also in the absence of UPF3B overexpressing UPF3A in HEK293 or HeLa cells had no detectable negative effects on NMD efficiency, supporting the previous conclusion of UPF3A not being a general negative NMD regulator in human cells (Fig EV3H and I).

### Stronger NMD impairment in UPF3A‐UPF3B double KO cells

**Figure 4:**
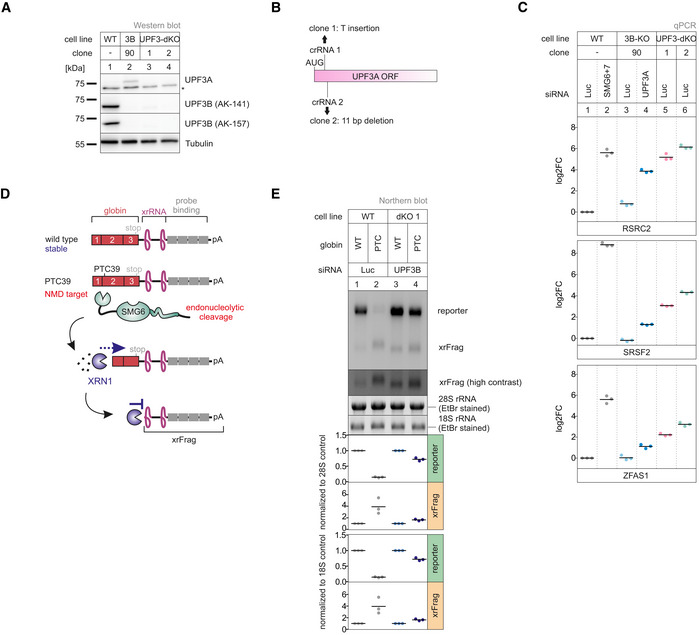
KO of both UPF3 paralogs results in strongly impaired NMD Western blot analysis of WT, UPF3B KO, and UPF3A‐UPF3B double KO cells (clones 1 and 2) (*n* = 1). UPF3A and UPF3B protein levels were detected, Tubulin serves as control. The asterisk indicates unspecific bands.Schematic depiction of the insertion/deletion in the UPF3A open reading frame resulting in the additional UPF3A KO in the UPF3B KO clone 90 generating UPF3 dKO clones.Quantitative RT–PCR of the indicated samples with the indicated KDs. For RSRC2 and SRSF2, the ratio of NMD isoform to canonical isoform was calculated. ZFAS1 expression was normalized to C1orf43 reference. Data points and means are plotted as log2 fold change (log2FC, *n* = 3).Schematic overview of the globin reporter constructs and their functional elements.Northern blot analysis of globin reporter and xrFrag. Ethidium bromide stained 28S and 18S rRNAs are shown as controls. Quantification results are shown as data points and mean (*n* = 3). Source data are available online for this figure.

Considering that UPF3A and UPF3B seemingly carry out redundant functions, we decided to create a cell line completely lacking both paralogs and aimed to generate UPF3A‐UPF3B double KO cells (UPF3 dKO). These cells should show stronger effects than the combination of a KO and a KD, since residual amounts of protein were typically still detected after siRNA treatment. Using the UPF3B‐KO clone 90 as parental cell line, two potential UPF3 dKO clones 1 and 2 were generated (Fig 4A), which differed in the guide RNAs used to target exon 1 of UPF3A. We confirmed that both cell lines contained frame shift‐inducing insertions/deletions at the respective positions in the UPF3A gene (Fig 4B, Appendix Fig S2A and B). We first explored by qPCR how strongly the UPF3 dKO affected NMD (Fig 4C). For all three tested genes, the expression of the NMD‐sensitive isoform was further increased compared to the previously used combination of UPF3B KO with additional UPF3A KD. Of note, the NMD inhibitory effect observed in the UPF3 dKOs became more pronounced after UPF3B siRNA transfection, suggesting that low levels of residual UPF3B protein were still present in the dKO cells (Appendix Fig S2C).

The expression levels of endogenous NMD substrates could be influenced by transcription rates or other indirect effects, which could lead to over‐ or underestimating NMD inhibition. Therefore, we investigated the NMD efficiency in the UPF3 dKO cells using the well‐established β‐globin NMD reporter. To this end, we stably integrated β‐globin WT or PTC39 constructs in WT and UPF3 dKO cells (Fig 4D). These reporters also contained XRN1‐resistant sequences (xrRNAs) in their 3’ UTRs, which allowed us to analyze not only the degradation of the full‐length reporter mRNA but also to quantify decay intermediates (called xrFrag) (Boehm *et al*, 2016; Voigt *et al*, 2019). The PTC39 mRNA was efficiently degraded and a strong xrFrag observed in WT cells, whereas in UPF3 dKO cells, the PTC39 reporter accumulated to high levels (72% compared to the WT mRNA), which was accompanied by a decrease in the amount of xrFrag (Fig 4E, lane 2 vs. lane 4). In line with the previous observations, this result indicated a strong decrease of NMD activity upon the KO of both UPF3 paralogs using a robust NMD reporter pair expressed independently of endogenous NMD substrates.

**Figure EV4:**
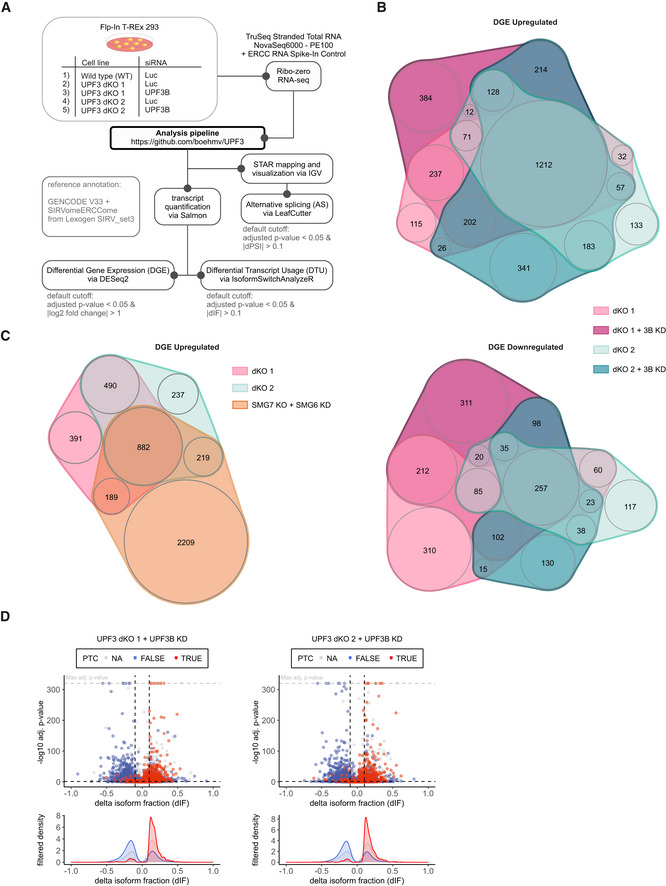
Genes upregulated in UPF3 dKO cells are high confidence NMD targets Schematic overview of the analysis pipeline.nVenn Diagrams showing the overlap of upregulated (upper panel) or downregulated genes (lower panel) in the UPF3 dKO cell lines 1 and 2, both with and without a supportive UPF3B KD. Log2 fold change < −1 (downregulated) or > 1 (upregulated) and adjusted *P*‐value (*P*_adj_) < 0.05. DGE = Differential Gene Expression.nVenn Diagram showing the overlap of upregulated genes in the two UPF3 dKO clones and previously analyzed SMG7 KO cells with SMG6 KD (Data ref.: Boehm *et al*, 2021) as control for cells with inhibited NMD. The overlap demonstrates high‐confidence NMD targets. Cut‐offs: log2FoldChange > 1 and adjusted *P*‐value (*P*_adj_) < 0.05. DGE = Differential Gene Expression.Volcano plots showing the differential transcript usage (via IsoformSwitchAnalyzeR) in various RNA‐Seq data. Isoforms containing GENCODE (release 33) annotated PTC (red, TRUE), regular stop codons (blue, FALSE) or having no annotated open reading frame (gray, NA) are indicated. The change in isoform fraction (dIF) is plotted against the ‐log10 adjusted *P*‐value (*P*_adj_). Density plots show the distribution of filtered isoforms in respect to the dIF, cutoffs were |dIF| > 0.1 and *P*_adj_ < 0.05. *P*‐values were calculated by IsoformSwitchAnalyzeR using a DEXSeq‐based test and corrected for multiple testing using the Benjamini–Hochberg method.

**Figure 5:**
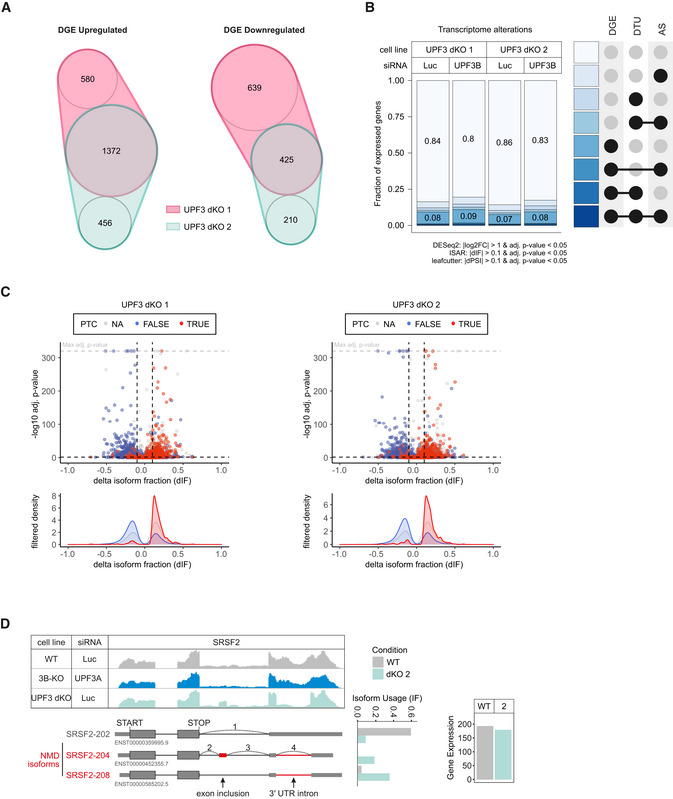
RNA‐seq reveals strong global upregulation of NMD‐sensitive targets upon UPF3 dKO in HEK293 cells nVenn Diagram showing the overlap of up‐ or downregulated genes in the UPF3 dKO cell lines 1 and 2. Log2 fold change < −1 (downregulated) or > 1 (upregulated) and adjusted *P*‐value (*P*_adj_) < 0.05. DGE = Differential Gene Expression.Fraction of expressed genes (genes with non‐zero counts in DESeq2) were calculated which exhibit individual or combinations of differential gene expression (DGE), differential transcript usage (DTU), and/or alternative splicing (AS) events in the indicated conditions using the respective computational analysis (cutoffs are indicated). AS and DTU events were collapsed on the gene level. For DGE, *P*‐values were calculated by DESeq2 using a two‐sided Wald test and corrected for multiple testing using the Benjamini–Hochberg method. For DTU, *P*‐values were calculated by IsoformSwitchAnalyzeR using a DEXSeq‐based test and corrected for multiple testing using the Benjamini–Hochberg method. For AS, *P*‐values were calculated by LeafCutter using an asymptotic chi‐squared distribution and corrected for multiple testing using the Benjamini–Hochberg method.Volcano plots showing the differential transcript usage (via IsoformSwitchAnalyzeR) in various RNA‐Seq data. Isoforms containing GENCODE (release 33) annotated PTC (red, TRUE), regular stop codons (blue, FALSE) or having no annotated open reading frame (gray, NA) are indicated. The change in isoform fraction (dIF) is plotted against the ‐log10 adjusted *P*‐value (*P*_adj_). Density plots show the distribution of filtered isoforms in respect to the dIF, cutoffs were |dIF| > 0.1 and *P*_adj_ < 0.05. *P*‐values were calculated by IsoformSwitchAnalyzeR using a DEXSeq‐based test and corrected for multiple testing using the Benjamini–Hochberg method.Read coverage of SRSF2 from the indicated RNA‐seq sample data with or without UPF3A siRNA treatment shown as Integrative Genomics Viewer (IGV) snapshot. The canonical and NMD‐sensitive isoforms are schematically indicated below. Quantification of gene expression and isoform usage by IsoformSwitchAnalyzeR (right).

To establish transcriptome‐wide insights into UPF3A and UPF3B function, we carried out RNA‐seq for both UPF3 dKO clones, which was combined with and without UPF3B KD treatment to eliminate potentially present residual UPF3B proteins (Fig EV4A and Datasets [Link], [Link], for total numbers see Appendix Fig S1A and B). Differential gene expression analysis showed that more than three times as many genes were upregulated than downregulated in both dKO cells (Figs 5A and EV4B). This is consistent with the redundant role of UPF3B and UPF3A as NMD‐supporting factors. The considerable overlap of 1,372 upregulated genes between both clones also suggests that we identified high‐confidence UPF3 targets. Furthermore, 882 of these genes were also significantly upregulated in previously generated SMG7 KO plus SMG6 KD data (Fig EV4C, ref data: (Boehm *et al*, 2021)) indicating that these are universal NMD‐targets and not specific to a certain branch of the NMD pathway. In addition to DGE, subsets of the expressed genes showed changes in alternative splicing or/and differential transcript usage (Fig 5B). In total, 14–16% of the detectable transcriptome showed single or combined changes (DGE, DTU and/or AS) and up to 20% when the cells were treated with an additional UPF3B KD.

In agreement with NMD inhibition in the UPF3 dKOs, we saw that many transcripts containing a PTC were upregulated, while the corresponding transcripts without a PTC were downregulated (Figs 5C and EV4D). Under these conditions, the IGV snapshot of the NMD‐target SRSF2 showed NMD‐inducing exon inclusion and 3’ UTR splicing events, which were not visible in combined UPF3B KO/UPF3A KD cells (Fig 5D). Collectively, the RNA‐seq data support the previously observed strong NMD inhibition in response to the complete absence of both UPF3 paralogs and hence their proposed redundancy.

### UPF3A supports NMD independent of a bridge function

**Figure 6:**
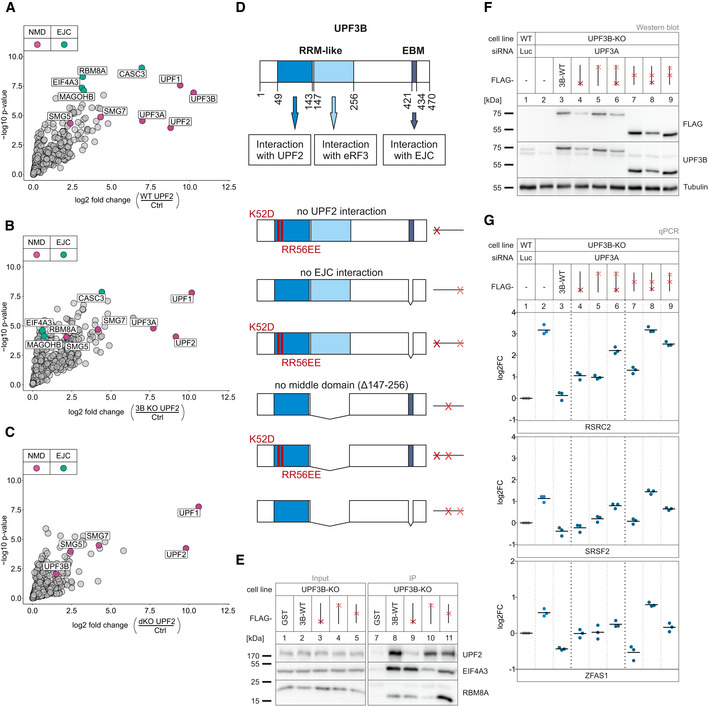
Interaction of UPF3A and UPF3B with the EJC is dispensable to elicit NMD A–CVolcano plots of label free mass spectrometry‐based analysis of the interaction partners of UPF2 in WT cells treated with control siRNAs and the UPF3B KO clone 90 and dKO clone 1 both treated with siRNAs targeting UPF3B (*n* = 4 biologically independent samples). (A) FLAG‐UPF2 in WT against FLAG‐GST control in WT cells, (B) UPF2 in 3B KO cells against FLAG control in WT cells, (C) UPF2 in dKO cells against FLAG control in WT cells. Points labeled in purple indicate NMD factors; points labeled in turquoise indicate EJC components. Cutoffs: Log2 fold change ≥ 0.DSchematic representation of the UPF3B protein domains and the respective functions. Below are the mutated rescue constructs and their respective abstract placeholders. RRM = RNA recognition motif, EBM = EJC binding motif.ERepresentative Western blot after FLAG co‐immunoprecipitation (IP) of FLAG‐tagged GST (control) or UPF3B WT and single mutant constructs in UPF3B KO cells (*n* = 3).FWestern blot analysis of WT and UPF3B KO clone 90 with Luciferase and UPF3A KDs respectively. Monitored expression of the FLAG‐tagged UPF3B rescue construct shown in (D). Rescue construct protein levels were detected with anti‐FLAG and anti‐UPF3B (AK‐141) antibodies. Tubulin serves as control (*n* = 1).GQuantitative RT–PCR of the samples from (F). For RSRC2 and SRSF2, the ratio of NMD isoform to canonical isoform was calculated. ZFAS1 expression was normalized to C1orf43 reference. Data points and means are plotted as log2 fold change (log2FC, *n* = 3). Source data are available online for this figure.

Next, we aimed to analyze whether the severe effects in the UPF3 dKOs are at least partly due to the loss of a protein–protein interaction bridge between UPF2 and the EJC, while the presence of UPF3A in the UPF3B KOs preserved this function ensuring NMD activity. Therefore, we expressed FLAG‐tagged UPF2 in WT, UPF3B KO, and UPF3 dKO cells and analyzed the UPF2 interactome using mass spectrometry (Dataset EV4). Consistent with the previously described interaction partners, we found many NMD factors as well as EJC proteins in the UPF2 interactome in WT cells (Fig 6A). Contrary to our expectation, the three EJC core components (EIF4A3, RBM8A, MAGOHB) barely co‐precipitated with UPF2 in the absence of UPF3B (compared to control: log2 fold change = 0.58, 0.63 and 0.74, respectively; Fig 6B) and were therefore strongly decreased in comparison to the WT cells (Appendix Fig S3A). Hence, the UPF2‐bound UPF3A was unable to establish a stable interaction with the EJC. Surprisingly, in the UPF3B KO cells the EJC‐associated CASC3 still showed relatively high levels of co‐precipitation (log2 fold change = 4.42), which therefore appears to be independent of the interaction with the other EJC components. In the UPF3 dKOs, all interactions with EJC proteins including CASC3 were completely lost (Fig 6C and Appendix Fig S3B). The latter was also observed in a comparable approach employing stable isotope labeling with amino acids in cell culture (SILAC) to analyze the UPF2 interactome in the WT and UPF3 dKO cells (Dataset EV5). All EJC core components that were highly co‐precipitated in WT cells were lost in the absence of both UPF3 paralogs (Appendix Fig S3C–E).

### Partially redundant UPF3B domains support NMD

**Figure EV5:**
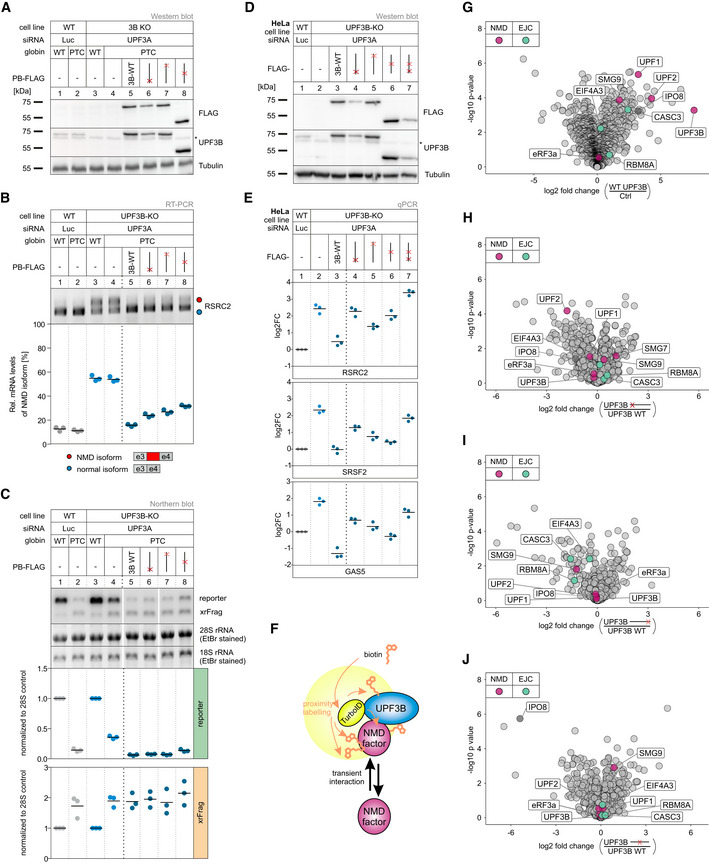
Single mutations do not destroy UPF3B NMD functionality in HEK293 and HeLa cells AWestern blot analysis of WT and UPF3B KO clone 90 cells with Luciferase and UPF3A KD, respectively. Monitored expression of globin WT and PTC39 reporters and the indicated UPF3B rescue constructs. Rescue construct protein levels were detected with anti‐FLAG and anti‐UPF3B (AK‐141) antibodies. Tubulin serves as control (*n* = 1). The asterisk indicates an unspecific band.BEnd‐point RT–PCR detection of RSRC2 transcript isoforms in the samples from (A). The detected RSRC2 isoforms are indicated on the bottom, the NMD‐inducing included exon is marked in red (e = exon). Relative mRNA levels of RSRC2 isoforms were quantified from bands of agarose gels (*n* = 3).CNorthern blot analysis of globin reporter and xrFrag. Ethidium bromide stained 28S and 18S rRNAs are shown as controls. Lanes 5 and 6 were mirrored because of a pipetting error. Quantification results are shown as data points and mean (*n* = 3).DWestern blot analysis of HeLa WT and UPF3B KO cells with Luciferase and UPF3A KDs respectively. Monitored expression of the FLAG‐tagged UPF3B rescue construct shown in (Fig 6D). Rescue construct protein levels were detected with anti‐FLAG and anti‐UPF3B (AK‐141) antibodies. Tubulin serves as control (*n* = 1). The asterisk indicates an unspecific band.EQuantitative RT–PCR of the samples from (D). For RSRC2 and SRSF2, the ratio of NMD isoform to canonical isoform was calculated. GAS5 expression was normalized to EMC7 reference. Data points and means are plotted as log2 fold change (*n* = 3).FOverview of TurboID‐mediated proximity labeling of UPF3B WT and UPF3B mutant construct binding partners in UPF3B KO clone 90 with additional UPF3A KD. Transient UPF3B interactors are marked with biotin via TurboID catalysis. Biotinylated proteins are subsequently enriched with streptavidin beads.G–JVolcano plots of mass spectrometry‐based analysis of streptavidin‐enriched biotinylated proteins in the respective comparison of conditions. (G) FLAG‐TurboID‐UPF3B against FLAG‐TurboID control, (H) FLAG‐TurboID‐UPF3B ΔUPF2 interaction (K52D/RR56EE) against FLAG‐TurboID‐UPF3B, (I) FLAG‐TurboID‐UPF3B ΔEJC interaction (Δ421–434) against FLAG‐TurboID‐UPF3B, (J) FLAG‐TurboID‐UPF3B Δmiddle domain (Δ147–256) against FLAG‐TurboID‐UPF3B, all in UPF3B KO + UPF3A KD cells. Points labeled in purple indicate NMD factors; points labeled in turquoise indicate EJC components (*n* = 3 biologically independent samples). Source data are available online for this figure.

With regard to the surprising observation that UPF3A apparently elicits NMD without interacting with the EJC, we aimed to investigate the molecular features required by UPF3A and UPF3B to support NMD via rescue experiments. In principle, the UPF3 dKO cells represent an ideal system for this approach. However, apart from the residual amounts of UPF3B that were still expressed, we noticed that the UPF3 dKOs were able to upregulate the expression of a shortened UPF3B variant after long‐term cultivation. Since this phenomenon did not occur in the single UPF3B KOs, we generated stable UPF3B KO cell lines expressing various inducible UPF3B constructs with individual or combined binding site mutations (Fig 6D–F and Appendix Fig S3F). Transfection of these cells with UPF3A siRNAs resulted in the robust depletion of both UPF3 paralogs, which enabled us to analyze the rescue activities of individual UPF3B variants. Considering the established role of UPF3B as a bridge between UPF2 and the EJC, which we validated in the mass spec analysis, it was surprising to see that disruption of these interactions either did not affect or only mildly affected the rescue ability of UPF3B, depending on the analyzed NMD substrate (Fig 6G, lane 3 vs. lanes 4 and 5). Both mutants also completely rescued degradation of the established NMD reporter β‐globin PTC39 (Fig EV5A–C). This is partially consistent with the observed functional NMD in UPF3B KOs, despite the apparent inability of UPF3A to form a bridge between UPF2 and the EJC (Figs 3C and 6B). However, mutating both UPF3B binding sites (disrupting UPF2 and EJC binding) strongly inhibited the NMD‐activating function of UPF3B (Fig 6G, lane 3 vs. lane 6).

It was recently reported that EJC‐bound or free UPF3B can interact with the eukaryotic release factor 3a (eRF3a) via the so far uncharacterized middle domain (aa 147–256) (Neu‐Yilik *et al*, 2017). With this interaction and binding of the terminating ribosome, UPF3B can delay translation termination, which defines aberrant termination events and triggers NMD (Amrani *et al*, 2004; Peixeiro *et al*, 2012; Neu‐Yilik *et al*, 2017). To investigate the impact of this interaction on NMD, we created UPF3B variants lacking that specific middle domain or combined the deletion with the previously used interaction mutations (Fig 6D–F). We observed a similar pattern as the UPF3B mutants examined in the previous experiment: when only the middle domain was deleted, UPF3B was able to rescue NMD comparable to the WT protein or slightly worse, depending on which NMD substrate was analyzed (Fig 6G, lane 7). Almost complete rescue of NMD activity by the UPF3B protein lacking the middle domain was also observed with the globin NMD reporter (Fig EV5A–C). However, in combination with a mutation in the UPF2‐ or the EJC‐binding site its function in NMD was severely impaired (Fig 6G, lanes 8 and 9). This suggests that if the classic bridge formation is inhibited by removing either of the interaction sites, UPF3B relied on the function carried out by the uncharacterized middle domain. Similar rescue effects of the UPF3B mutants were also observed in HeLa cells (with NMD substrate specific differences), indicating that the general function of the domains and their importance is conserved in other human cell lines (Fig EV5D and E).

Given that mutations in different domains of UPF3B slightly inhibited its NMD activity, but showed stronger effects when combined with each other, we wanted to determine general changes in the interactome of these UPF3B mutants. To this end, we performed TurboID‐based proximity labelling (Branon *et al*, 2018; Cho *et al*, 2020) to detect transient interactions of UPF3B WT and the three single mutants (Fig EV5F, Dataset EV6). As expected, UPF2 and EJC components were present in the UPF3B WT sample, but significantly reduced in the respective interaction mutant (Fig EV5G–I). To our surprise, the interaction of UPF1 with UPF3B was not reduced by any of the single mutations, suggesting an alternative and not strictly bridge‐dependent mechanism for physical proximity between UPF3B and UPF1. This observation agrees well with recent *in vitro* binding studies showing that UPF3B can directly interact with UPF1 (Neu‐Yilik *et al*, 2017). Furthermore, we could not confirm the loss of the eRF3a interaction for the mutant lacking the middle domain, because eRF3a was not enriched in the WT sample (Fig EV5G and J). However, some proteins appeared to interact specifically with the middle domain, for example the protein IPO8, which is a known factor involved in miRNA metabolism (Weinmann *et al*, 2009).

**Figure 7:**
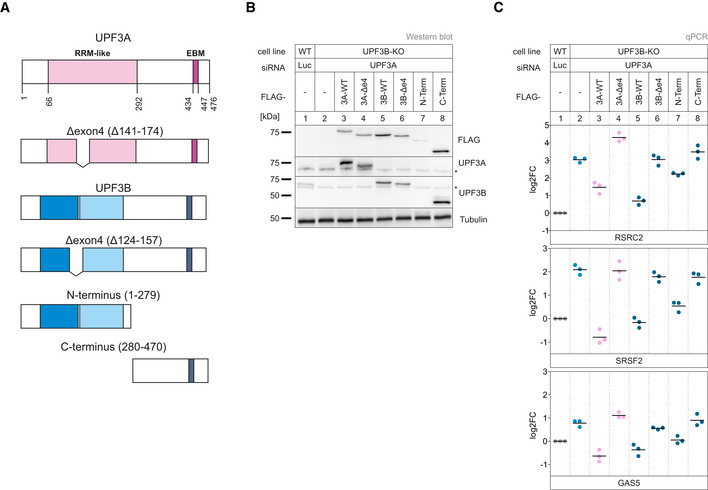
WT UPF3A can rescue NMD in full extent. Deletion on exon 4 disrupts functionality in both paralogs Schematic representation of the UPF3A and UPF3B protein domains. Below are the respective mutated rescue constructs.Western blot analysis of WT and UPF3B KO clone 90 with Luciferase and UPF3A KDs respectively. Monitored expression of the FLAG‐tagged UPF3A and UPF3B rescue construct shown in (A). Rescue construct protein levels were detected with anti‐FLAG, anti‐UPF3A, and anti‐UPF3B (AK‐141) antibodies. Tubulin serves as control (*n* = 1). The asterisk indicates unspecific bands.Quantitative RT–PCR of the samples from (B). For RSRC2 and SRSF2, the ratio of NMD isoform to canonical isoform was calculated. Data points and means are plotted as log2 fold change (log2FC) (*n* = 3). Source data are available online for this figure.

To better understand the NMD promoting function of UPF3A, we performed a rescue experiment in the above used UPF3B KO UPF3A KD cells (Fig 7A and B). Expression of the siRNA insensitive UPF3A construct restored NMD functionality with similar efficiency as the UPF3B rescue (Fig 7C lane 3 vs. lane 5), underlining our previous statement: UPF3A supports and elicits NMD comparably to its paralog UPF3B.

Also comparable to UPF3B, UPF3A has a second naturally occurring isoform but instead of skipping exon 8 (like UPF3B), it excludes its fourth exon. This alternative isoform is produced in approximately one third of the cases (Appendix Fig S3G) in WT HEK293 cells. We were interested, whether this isoform was as potent to elicit NMD as the full‐length construct. Expression of the exon 4 UPF3A deletion construct in the UPF3 depleted cells showed no rescue (Fig 7C lane 4). Hence, removing exon 4 inactivates the NMD‐supporting function of UPF3A. The exact reason for this observation could besides functional reasons also be due to problems of correct protein folding.

In view of these observations and the overlap of exon 4 (aa 124–157) with the middle domain (aa 147–256), we decided to investigate which effect the deletion of the homologous exon 4 in the paralog UPF3B has. The UPF3B Δe4 construct behaved like the corresponding UPF3A construct and showed no NMD rescue activity (lane 6). However, the expression of the N‐terminus of UPF3B was able to restore NMD comparably to the WT protein (lane 7). This is consistent with all our previous findings, since the first 279 amino acids contain the UPF2‐binding site as well as the middle domain, which was shown to be sufficient to elicit NMD (Fig 6G lane 5). Due to the fact that the C terminus contains only one interaction site and lacks exon 4, its incapability to rescue NMD is in line with our previous experiments. Overall, our results show that each of the UPF3 proteins lacking exon 4 are NMD inactive.

## Discussion

Methodological advances—be it improved analytics or novel experimental approaches—can help to find new answers to old biological problems. Equipped with powerful new molecular biology methods, we set out to answer the question which functions the two UPF3 paralogs carry out in human cells. Since the initial description of mammalian UPF3A and UPF3B (Lykke‐Andersen *et al*, 2000; Serin *et al*, 2001), many researchers have been engaged in determining the function and work distribution of these two proteins in NMD. It became clear relatively early that UPF3A and UPF3B can both interact with UPF2 as well as the EJC and activate NMD (Lykke‐Andersen *et al*, 2000; Kim *et al*, 2001; Serin *et al*, 2001; Gehring *et al*, 2003). But significant differences in these interactions and the amounts of UPF3A and UPF3B proteins were also found, leading to the hypothesis that UPF3B is the central player and UPF3A more its backup (Kunz *et al*, 2006; Chan *et al*, 2009; Metze *et al*, 2013). Later, it was reported that in various mouse cells UPF3B is an NMD activator and UPF3A is mainly an NMD inhibitor, but potentially acting as an NMD activator for a small number of transcripts (Shum *et al*, 2016). Accordingly, removal of UPF3A resulted in enhanced NMD, whereas removal of UPF3B inhibited NMD. Recently, another function of UPF3B was discovered, namely that it is involved in different phases of translation termination (Neu‐Yilik *et al*, 2017). UPF3B interacts with both release factors *in vitro* (pulldown of purified proteins) and *in vivo* (co‐immunoprecipitation of co‐expressed proteins). In addition, UPF3B slows down translation termination and promotes dissociation of post‐termination ribosomal complexes *in vitro*.

**Figure 8:**
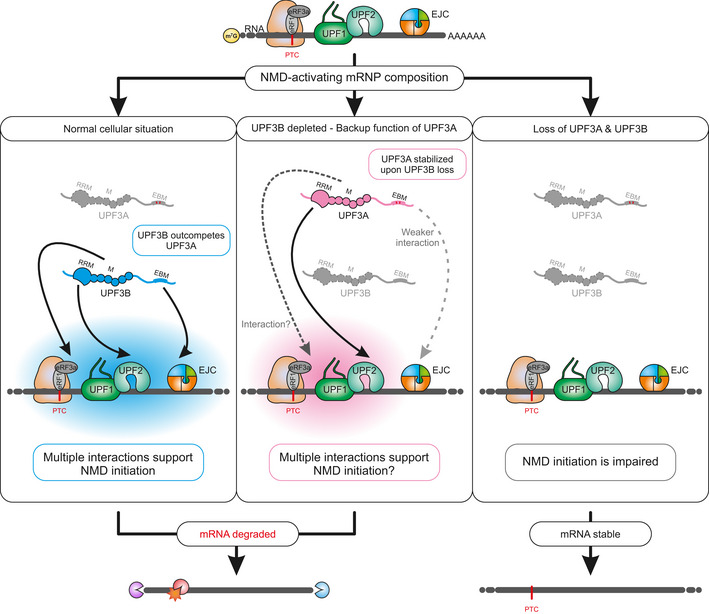
Model for functional redundancy of UPF3A and UPF3B An NMD activating mRNP composition will result in the activation of NMD in the presence of UPF3B (left part) or when UPF3B was depleted (middle part). In the absence of UPF3A and UPF3B, NMD is inactive (right part). In normal cells, UPF3B can engage in multiple, potentially transient interactions (depicted by blue sphere), for example, with the terminating ribosome via eRF3a, with UPF2 or with the EJC (left). When UPF3B is missing, UPF3A can at least partially compensate for it (depicted by pink sphere, middle). UPF3A interacts with UPF2 and possibly also with eRF3a. The interaction of UPF3A with the EJC is weaker than that of UPF3B, therefore UPF3A cannot establish a stable bridge between UPF2 and the EJC (middle).

With this work, we have set out to understand the functions of the two proteins UPF3A and UPF3B in NMD. All our data support the notion that the presence of either UPF3B or UPF3A is sufficient to maintain NMD activity in human cells (Fig 8). We see at most a weak inhibition of NMD in our HEK293 UPF3B KO cells (further discussed below). It is possible that the KO cells compensate for the loss of the knocked‐out gene—in this case UPF3B—over time, which would not be the case for siRNA knockdowns. However, the strength of the effects observed in UPF3A‐UPF3B dKO cells strongly argues against this possibility. Indeed, we also observed in earlier work that the effects of a KO on NMD activity are typically stronger than those of a KD (Gerbracht *et al*, 2020; Boehm *et al*, 2021). We also see no striking NMD inhibition in cells overexpressing UPF3A or NMD “boosting” in UPF3A KO cells. Only when we deplete in UPF3A or UPF3B KO cell lines the respective other protein by RNAi or genomic KO, NMD efficiency substantially decreases. Notably, even when we knocked out UPF3A and UPF3B together, the amount of stabilized NMD substrates as well as the extent of their stabilization was not comparable to a SMG7 KO in combination with either a SMG5 or a SMG6 knockdown (Boehm *et al*, 2021). We conclude that UPF3‐type proteins are either not absolutely essential for all NMD events or that the loss of both UPF3 paralogs is partially compensated for by another protein. We have no obvious candidate for this function, but it could be one of the other EJC‐interacting proteins that have been implicated to play a role in NMD (Baird *et al*, 2018; Mabin *et al*, 2018; Gerbracht *et al*, 2020).

We would like to justify our interpretation that the perturbations of either UPF3A or UPF3B protein levels (UPF3B KO, UPF3A KO or UPF3A overexpression) do not considerably influence NMD in the used human cell lines, which may contradict several previous studies (Nguyen *et al*, 2012; Shum *et al*, 2016; Domingo *et al*, 2020). One frequently used method to monitor the NMD status is to determine the number of differentially expressed genes by RNA‐seq. For example, a high number of upregulated genes upon UPF3B depletion is commonly interpreted as an inhibitory effect on NMD; especially if many of those genes express potentially NMD‐targeted transcripts (e.g., exhibiting an EJC downstream of stop codon or a long 3’ UTR) or have been found in previous NMD‐related studies. Based on this approach alone, we would conclude that the KO of UPF3B indeed impairs NMD, because 74 genes were upregulated (Appendix Fig S1A). However, if we consider the extent of differentially expressed genes in severely NMD‐inhibited conditions (SMG7 KO + SMG6 KD or UPF3 dKO), which display about 2,000–4,500 upregulated genes, the inhibitory effect of the UPF3B KO appears to be marginal (Appendix Fig S1A). This does not mean that the depletion of UPF3B has absolutely no effect on NMD, but rather that the extent of NMD inhibition is minimal, at least in our tested system. Of note, most of the previous studies using UPF3B depletion lacked matched positive controls with strong NMD inhibition, which makes it difficult to estimate to which extent NMD is impaired. Furthermore, differences in the used bioinformatic tools to determine differential gene expression, sequencing depth, and technology, but also inconsistent usage of effect strength or statistical cutoffs, further complicates the simple usage of the number of upregulated genes as a predictor of NMD status. Therefore, we have also employed transcript‐ and PTC‐specific analyses as orthogonal approaches to determine the NMD activity. Not only the DTU results (Appendix Fig S1B) but also a complementary differential transcript expression (DTE) analysis (Appendix Fig S4) both support our general conclusion that KO or overexpression of UPF3A, as well as single KO of UPF3B do not lead to mis‐regulation of many PTC‐positive transcripts. Again, this does not mean that no NMD‐targeted transcripts were differentially regulated at all in those conditions, but rather that compared to the positive controls the numbers are low. Altogether our data clearly indicate that the combined UPF3A and UPF3B depletion is required to substantially disturb NMD activity, which is best explained by the functional redundancy between UPF3B and UPF3A. We believe this phenomenon can also be described by the term “fault tolerance”, which is commonly used e.g. in software engineering. Fault‐tolerant systems continue to operate properly after the failure of one component, although with potentially reduced quality. Similarly, the functional redundancy of UPF3A and UPF3B makes NMD fault‐tolerant and allows almost normal NMD to be carried out after loss of either paralog.

Although the data from our human cell lines provide evidence against a general NMD‐inhibitory function of UPF3A, experimental differences exist between our work and the work describing the NMD inhibition by UPF3A. While we have used human HEK293 and HeLa cells, the results of Shum *et al* were primarily obtained in various mouse cell types, but also partially in HeLa cells. Both, the different studied organisms (mouse vs human) and the different types of cells (e.g., even using different HeLa sublines) could have influenced the results. To circumvent these potential technical issues, it would be required to carry out NMD activity measurements in different human and mouse cell types/lines with varying UPF3A and/or UPF3B levels, ideally with matching positive controls (e.g., depleted SMG6‐SMG7). Thus, it remains an open question how relevant the individual UPF3 paralogs and their potential NMD activating or repressing function are in different vertebrates and/or different cell types. Interestingly, in a parallel manuscript, Yi *et al* find that not only human but also mouse UPF3A was able to restore NMD activity in their HCT116 UPF3A‐UPF3B dKO cell line (Yi *et al*, 2022). In case of a general NMD inhibitory function of human or mouse UPF3A, no rescue effect should have been observed, which further questions the proposed broadly acting role of UPF3A as an NMD repressor.

The different KO cells that we have generated in the course of this project enabled us to conduct experiments that went beyond investigating UPF3‐dependent NMD substrates in human cells. Specifically, we were able to study the composition of NMD complexes without UPF3B or both UPF3 proteins and to carry out rescue experiments with different UPF3A and UPF3B protein variants. As expected, we observed that in the absence of UPF3A and UPF3B, the interaction between UPF2 and the EJC is lost. This bridging by UPF3A or UPF3B between UPF2‐containing NMD complexes and the EJC was previously considered to be essential for NMD. However, two observations argue against UPF3A and UPF3B being mainly bridging proteins. First, UPF3B mutants that cannot interact with either the EJC or UPF2 almost fully rescue NMD. Only when both interaction sites were mutated, UPF3B lost its NMD function. This indicates that the interaction with one of the two interaction partners is sufficient to maintain NMD. Second, we observed that not only in UPF3A‐UPF3B double KO cells but also in UPF3B KO cells, the bridge between UPF2 and the EJC was lost. Although quite surprising at first glance, this is in good agreement with previous results showing that the interaction between the EJC and UPF3A is substantially weaker than that between the EJC and UPF3B (Kunz *et al*, 2006). Indeed, earlier structural data also argue against a bridging function of UPF3B. In the cryo‐EM structure of an EJC‐UPF3B‐UPF2‐UPF1 complex, UPF1 did not face towards a possible terminating ribosome in the 5' direction, but instead in 3' direction (Melero *et al*, 2012). Therefore, we suggest that the interactions between all these proteins do not take place at a single time point during NMD and that the formation of a stable EJC‐UPF3B‐UPF2‐UPF1 complex is not necessary for NMD. However, the common denominator of all NMD‐active UPF3B mutants seems to be that they maintain their interaction with UPF1. It is unclear at this stage how UPF3 and UPF1 interact with each other in the absence of UPF2 and if another protein is bridging the interaction. As this UPF2‐independent interaction of UPF3 and UPF1 is a recurring theme in the NMD literature (Ivanov *et al*, 2008; Neu‐Yilik *et al*, 2017), addressing this finding will be an important direction for future research.

A possible functional role of UPF3B may be to act as a non‐rigid support during the assembly of early NMD complexes. Indeed, our rescue experiments showed that three domains of UPF3B play a role for its NMD activity: the UPF2‐binding site, the middle domain and the EJC binding motif (EBM) (Fig 8, Appendix Fig S5A). Our observations suggest a "two out of three" model in which inactivation of any two domains together disrupts UPF3B activity (Appendix Fig S5B). This property adds another layer of apparent NMD fault tolerance as UPF3B can still activate NMD even if one interaction is impaired.

One factor whose function needs to be examined in more detail in the context of UPF3A is CASC3. Our mass spectrometry analysis shows that CASC3 co‐immunoprecipitates very well with UPF2 in wild‐type cells. CASC3 still partially precipitates with UPF2 in UPF3B KO cells, although the interaction of the other EJC factors is reduced to background levels. In previous work, we observed that UPF3B interacts less well with the EJC when CASC3 is knocked out (Gerbracht *et al*, 2020). This indicates that an interaction between CASC3 and the UPF3 proteins exists that is not well understood so far. What kind of interaction this is and what function it has will be interesting to address in future experiments.

Our own work and the work of Yi *et al* have re‐examined the functions of the human UPF3 paralogs UPF3A and UPF3B (Yi *et al*, 2022). Together, the studies confirmed some previous findings and contradict others, thereby successfully (re‐)defining the role of UPF3 proteins and their functional domains in human cells. A few questions remain unanswered and need to be addressed in the future, for example, how exactly the middle domain supports NMD. As described above, our results have implications for the understanding of the NMD mechanism, as they are incompatible with, and thus exclude, certain models of NMD. In addition, they may also help to better understand the link between UPF3B and intellectual disability and which domains of UPF3A modulate the severity of the disease and may therefore be potential targets for therapy.

## Materials and Methods

### Cell culture

Flp‐In T‐REx‐293 (human, female, embryonic kidney, epithelial; Thermo Fisher Scientific, RRID:CVCL_U427) and HeLa Flp‐In T‐REx (HeLa FT; human, female, cervix; generated by Elena Dobrikova and Matthias Gromeier, Duke University Medical Center) cells were cultured in high‐glucose, GlutaMAX DMEM (Gibco) supplemented with 9% fetal bovine serum (Gibco) and 1× Penicillin Streptomycin (Gibco). The cells were cultivated at 37°C and 5% CO_2_ in a humidified incubator. The generation of knockout and stable cell lines is described below and all cell lines are summarized in Dataset EV7.

### siRNA‐mediated knockdowns

For reverse transfection, the cells were seeded at a density of 2.5 × 10^5^ cells per well. The transfection solution contained 2.5 µl Lipofectamine RNAiMAX and 60 pmol of the respective siRNAs. For the UPF3A and UPF3B knockdowns 30 pmol of both belonging siRNAs were used. In preparation for mass spectrometry, 2.5 × 10^6^ were reverse transfected in 10 cm plates using 6.25 µl Lipofectamine RNAiMAX and 150–200 pmol siRNA (or half of it for each of the two siRNAs for UPF3A or UPF3B). All siRNAs used in this study are listed in Dataset EV7.

### Plasmid transfection

For each stable transfection, 2.5–3.0 × 10^5^ cells were seeded one day prior transfection in 6‐wells. To express the N‐terminally FLAG‐tagged protein constructs and reporter mRNAs for northern blotting, they were stably integrated using the PiggyBac (PB) Transposon system with the cumate‐inducible PB‐CuO‐MCS‐BGH‐EF1‐CymR‐Puro vector. This vector was modified from the original vector (PB‐CuO‐MCS‐IRES‐GFP‐EF1α‐CymR‐Puro (System Biosciences)) by replacing the IRES‐GFP cassette with a BGH polyA signal. Per well 1.0 μg of the respective PB vector and 0.8 μg PB Transposase were transfected using a calcium phosphate‐based system with BES‐buffered saline (BBS). Additionally, 0.5 μg of pCI‐maxGFP was transfected as a visual feedback for transfection efficiency. In case of the dual expression of an mRNA reporter and a UPF3B rescue construct, the globin reporters were stably integrated via the Flp‐In T‐REx system. Therefore, 1.5 µg of pcDNA5 construct was transfected together with 1 µg of the Flp recombinase expressing plasmid pOG44, again using the calcium phosphate method. 48 h later, the cells were pooled in 10 cm plates and selected for positive cells by incubation in media containing 2 μg/ml puromycin (for PB system, InvivoGen) or 100 µg/ml hygromycin (for Flp‐In T‐Rex system, InvivoGen) for a week. To induce expression of the PB constructs, 30 µg/ml cumate was added, Flp‐In T‐REx constructs were induced with 1 µg/ml doxycycline. The cells were harvested 72 h after for continuing experiments.

The mRNA reporter constructs β‐globin WT and β‐globin PTC are available on Addgene (IDs 108375‐108376). All vectors used in this study are listed in Dataset EV7.

### Generation of knockout cells using CRISPR‐Cas9

The knockouts were performed using the Alt‐R CRISPR‐Cas9 system (Integrated DNA Technologies) and reverse transfection of a Cas9:guideRNA ribonucleoprotein complex using Lipofectamine RNAiMAX (Thermo Fisher Scientific) according to the manufacturer’s protocol. The crRNA sequence (Integrated DNA Technologies) to target UPF3B was /AltR1/rArGrArUrArArGrCrArGrGrArUrCrGrCrArArCrArGrUrUrUrUrArGrArGrCrUrArUrGrCrU/AltR2/. For UPF3A, the crRNA sequences were /AltR1/rCrCrGrCrArArCrCrGrGrArGrGrArCrGrArArGrUrGrUrUrUrUrArGrArGrCrUrArUrGrCrU/AltR2/ for clone 1 and /AltR1/rGrCrGrGrUrGrGrArArCrUrGrCrArCrUrUrCrUrArGrUrUrUrUrArGrArGrCrUrArUrGrCrU/AltR2/ for clone 2. Reverse transfection was performed on 1.5 × 10^5^ cells per crRNA in 12‐well plates. 48 h after transfection, the cells were trypsinized, counted, and seeded at a mean density of a single cell per well in 96‐well plates. Cell colonies originating from a single clone were then screened via Western blot and genome editing of UPF3A and UPF3B was analyzed on the genomic level via DNA extraction and Sanger sequencing. Alterations on the transcript level were analyzed via RNA extraction followed by reverse transcription and Sanger sequencing.

### DNA and RNA extraction

Genomic DNA extraction using QuickExtract DNA Extraction Solution (Lucigen) was performed according to manufacturer’s instruction. For RNA extraction, cells were harvested with 1 ml RNAsolv reagent (Omega Bio‐Tek) per 6 well and RNA was isolated according to manufacturer’s instruction, with the following changes: instead of 200 μl chloroform, 150 μl 1‐Bromo‐3‐chloropropane (Sigma‐Aldrich) was added to the RNAsolv. Additionally, in the last step, the RNA pellet was dissolved in 20 μl RNase‐free water by incubating for 10 min on a shaking 65°C heat block.

### Western blotting

SDS–polyacrylamide gel electrophoresis and immunoblot analysis were performed using protein samples harvested with RIPA buffer (50 mM Tris/HCl pH 8.0, 0.1% SDS, 150 mM NaCl, 1% IGEPAL, 0.5% deoxycholate) or samples eluted from Anti‐FLAG M2 magnetic beads. For protein quantification, the Pierce Detergent Compatible Bradford Assay Reagent (Thermo Fisher Scientific) was used. All antibodies used in this study are listed in Dataset EV7. Detection was performed with Western Lightning Plus‐ECL (PerkinElmer) or ECL Select Western Blotting Detection Reagent (Amersham) and the Vilber Fusion FX6 Edge imaging system (Vilber Lourmat). Protein levels were quantified in a semi‐automated manner using the ImageQuant TL 1D software with a rolling‐ball background correction. Signal intensities were normalized to the internal control (tubulin) before calculation of mean values. Quantification results are shown as data points and mean.

### Semi‐quantitative and quantitative reverse transcriptase (RT)–PCR

Reverse transcription was performed with 1–4 µg of total RNA in a 20 µl reaction volume with 10 µM VNN‐(dT)_20_ primer and the GoScript Reverse Transcriptase (Promega). For the semi‐quantitative end‐point PCRs, the MyTaq Red Mix (Bioline) was used. Quantitative RT–PCRs were performed with the GoTaq qPCR Master Mix (Promega), 2% of cDNA per reaction, and the CFX96 Touch Real‐Time PCR Detection System (Bio‐Rad). Each biological replicate was repeated in technical triplicates and the average Ct (threshold cycle) value was measured. When isoform switches were measured, values for NMD sensitive isoforms were normalized to the canonical isoforms to calculate ΔCt. For differentially expressed genes, the housekeeping gene C1orf43 or EMC7 values were subtracted from the target value to receive the ΔCt. To calculate the mean log2 fold changes, three biologically independent experiments were used. The log2 fold changes are visualized as single data points and mean. All primers used in this study are listed in Dataset EV7.

### RNA‐sequencing and computational analyses

Five different RNA‐seq experiments were performed: (i) unaltered Flp‐In T‐REx 293 wild type (WT) cells and WT cells overexpressing the UPF3A WT construct via the PB‐Transposase system. (ii) unaltered HeLa Flp‐In T‐REx wild type (WT) cells and WT cells overexpressing the UPF3A WT construct via the PB‐Transposase system. (iii) Flp‐In T‐REx 293 wild‐type (WT) cells transfected with Luciferase siRNA and the UPF3A KO clones 14 and 20 treated with either Luciferase or UPF3B siRNAs. (iv) The control Flp‐In T‐REx 293 wild type (WT) cells with a Luciferase KD and the UPF3B KO clone 90 transfected with Luciferase or UPF3A siRNAs. (v) WT cells transfected with Luciferase siRNA and the two UPF3 double KO cell lines 1 and 2 transfected with either Luciferase or UPF3B siRNAs. RNA was purified using peqGOLD TriFast (VWR Peqlab; for UPF3B KO samples) or the Direct‐zol RNA MiniPrep kit including the recommended DNase I treatment (Zymo Research; all other samples) according to manufacturer’s instructions. Three biological replicates were analyzed for each sample.

The Lexogen SIRV Set3 Spike‐In Control Mix (SKU: 051.0x; for UPF3B KO samples) or ERCC RNA Spike‐In Mix (for all other samples) that provides a set of external RNA controls was added to the total RNA to enable performance assessment. The Spike‐Ins were used for quality control purposes, but not used for the final analysis of DGE, DTU, or AS.

Using the Illumina TruSeq Stranded Total RNA kit, library preparation was accomplished. This includes removing ribosomal RNA via biotinylated target‐specific oligos combined with Ribo‐Zero gold rRNA removal beads from 1 μg total RNA input. Cytoplasmic and mitochondrial rRNA gets depleted by the Ribo‐Zero Human/Mouse/Rat kit. After a purification step, the RNA gets cleaved and fragmented. These fragments are then reverse transcribed into first‐strand cDNA using reverse transcriptase and random primers. In the next step, using DNA polymerase I and RNase H second‐strand cDNA synthesis is performed. The resulting cDNA fragments then have the extension of a single “A” base and adapter ligation. To create the final cDNA library, the products are purified and enriched with PCR. Next, library validation and quantification (Agilent tape station) are performed, followed by pooling of equimolar amounts of library. The pool itself was then quantified using the Peqlab KAPA Library Quantification Kit and the Applied Biosystems 7900HT Sequence Detection System and sequenced on an Illumina HiSeq4000 sequencing instrument with an PE75 protocol (UPF3B KO samples) or Illumina NovaSeq6000 sequencing instrument with an PE100 protocol (all other samples).

Reads were aligned against the human genome (version 38, GENCODE release 33 transcript annotations (Frankish *et al*, 2019) supplemented with SIRVomeERCCome annotations from Lexogen; obtained from https://www.lexogen.com/sirvs/download/) using the STAR read aligner (version 2.7.3a) (Dobin *et al*, 2013). Transcript abundance estimates were computed with Salmon (version 1.3.0) (Patro *et al*, 2017) with a decoy‐aware transcriptome. After the import of transcript abundances using tximport (Soneson *et al*, 2015), differential gene expression analysis was performed with the DESeq2 (Love *et al*, 2014) R package (version 1.28.1) with the significance thresholds |log2FoldChange| > 1 and adjusted *P*‐value (*P*_adj_) < 0.05. Differential splicing was detected with LeafCutter (version 0.2.9) (Li *et al*, 2018) with the significance thresholds |deltapsi| > 0.1 and *P*_adj_ < 0.05. Differential transcript usage was computed with IsoformSwitchAnalyzeR (version 1.10.0) and the DEXSeq method (Robinson & Oshlack, 2010; Anders *et al*, 2012; Ritchie *et al*, 2015; Soneson *et al*, 2015; Vitting‐Seerup & Sandelin, 2017, 2019). Significance thresholds were |dIF| > 0.1 and adjusted *P*‐value (isoform_switch_q_value) < 0.05.

PTC status of transcript isoforms with annotated open reading frame was determined by IsoformSwitchAnalyzeR using the 50 nucleotide (nt) rule of NMD (Weischenfeldt *et al*, 2012; Vitting‐Seerup *et al*, 2014; Huber *et al*, 2015; Vitting‐Seerup & Sandelin, 2017). Isoforms with no annotated open reading frame in GENCODE were designated “NA” in the PTC analysis.

Differential transcript expression (DTE, Appendix Fig S4) was computed with Salmon followed by DESeq2 as described above with the following differences (based on (Love *et al*, 2018)): ignoreTxVersion during tximport was set to FALSE, scaledTPM was used to generate counts from abundance and only transcripts with equal or more than 5 counts in all samples were kept for DESeq2 analysis. The transcript biotype of all transcripts for DTE analysis was extracted from the GENCODE annotation.

All scripts and parameters for the RNA‐Seq analysis are available at GitHub [https://github.com/boehmv/UPF3]. Overlaps of data sets were represented via nVenn (Perez‐Silva *et al*, 2018) or the ComplexHeatmap package (version 2.6.2)(Gu *et al*, 2016). Integrative Genomics Viewer (IGV) (version 2.8.12) (Robinson *et al*, 2011) snapshots were generated from mapped reads (BAM files) converted to binary tiled data (tdf), using Alfred(Rausch *et al*, 2019) with resolution set to 1 and IGVtools.

### FLAG co‐immunoprecipitation

Expression of FLAG‐tagged proteins was induced for 72 h with 30 µg/ml cumate (2.5 × 10^6^ cells were seeded per 10 cm dish). The cells were lysed in 200 μl Buffer E (20 mM HEPES‐KOH (pH 7.9), 100 mM KCl, 10% glycerol, 1 mM DTT, Protease Inhibitor, 1 µg/ml RNase A) and sonicated using the Bandelin Sonopuls mini20 with 15× 1 s pulses at 50% amplitude with a 2.5 mm tip. Protein concentrations were measured using the Bradford assay and protein samples were adjusted to 1.6–2.9 mg/ml total protein (equal concentration for each samples of one replicate). 500 μl of these samples were incubated with 20 μl Anti‐FLAG M2 magnetic beads (Sigma) for 2 h on an overhead shaker at 4°C. The beads were then washed three times for 5 min with mild EJC‐Buffer (20 mM HEPES‐KOH (pH 7.9), 137 mM NaCl, 2 mM MgCl_2_, 0.2% Triton X‐100, 0.1% NP‐40). Co‐immunoprecipitated proteins were eluted with SDS‐sample buffer, separated by SDS–PAGE, and analyzed by immunoblotting.

### SILAC and mass spectrometry

HEK293 WT cells and the UPF3 dKO clone 2 expressing either FLAG‐tagged GST or UPF2 were labeled by culturing them for at least five passages in DMEM for SILAC medium (Thermo Fisher Scientific) supplemented with 9% FBS (Silantes), 1% penicillin–streptomycin and the labeled amino acids Lysin and Arginine at final concentrations of 0.798 and 0.393 mmol/l, respectively. The three conditions were “light” (unlabeled Lys/ Arg), “medium” (Lys4/ Arg6), and “heavy” (Lys8/ Arg10). Unlabeled proline was added in all conditions to prevent enzymatic Arginine‐to‐Proline conversion.

### Experimental setup for SILAC with FLAG‐tagged UPF2

Expression of FLAG‐GST and FLAG‐UPF2 was induced for 72 h with 30 µg/ml cumate. The cells were lysed in 250–400 μl Buffer E with 1 μg/ml RNase and sonicated using the Bandelin Sonopuls mini20 with 15× 1 s pulses at 50% amplitude with a 2.5 mm tip. Protein concentrations were measured using the Bradford assay and protein samples were adjusted to 1.6–1.7 mg/ml total protein. 600 μl of these samples were incubated with 30 μl Anti‐FLAG M2 magnetic beads (Sigma) for 2 h on an overhead shaker at 4°C. The beads were then washed three times for 5 min with mild EJC‐Buffer before eluting twice with 22 μl of a 200 μg/ml dilution of FLAG‐peptides (Sigma) in 1× TBS for 10 min at RT and 200 rpm each elution step. Another elution with 1× SDS loading buffer was performed to analyze pull down efficiency via Western blot. The FLAG‐peptide eluates were then mixed as followed: 7 μl of both light conditions, 14 μl medium and 14 μl heavy. 1 volume of SP3 (10% SDS in PBS) was added and the samples were reduced with 5 mM DTT and alkylated with 40 mM CAA.

Tryptic protein digestion was achieved by following a modified version of the single pot solid phase‐enhanced sample preparation (SP3) (Hughes *et al*, 2014). In brief, paramagnetic Sera‐Mag speed beads (Thermo Fisher Scientific) were added to the reduced and alkylated protein samples and then mixed 1:1 with 100% acetonitrile (ACN). Protein‐beads‐complexes form during the 8‐min incubation step, followed by capture using an in‐house build magnetic rack. After two washing steps with 70% EtOH, the samples were washed once with 100% ACN. Then they were air‐dried, resuspended in 5 μl 50 mM Triethylamonium bicarbonate supplemented with trypsin and LysC in an enzyme:substrate ratio of 1:50 and incubated for 16 h at 37°C. The next day the beads were again resuspended in 200 μl ACN and after 8 min incubation placed on the magnetic rack. Tryptic peptides were washed with 100% ACN and air‐dried before dissolved in 4% DMSO and transfer into 96‐well PCR tubes. The last step was the acidification with 1 μl of 10% formic acid, and then, the samples were ready for mass spec analysis.

Proteomics analysis was performed by the proteomics core facility at CECAD via data‐dependent acquisition using an Easy nLC1200 ultra high‐performance liquid chromatography (UHPLC) system connected via nano electrospray ionization to a Q Exactive Plus instrument (all Thermo Scientific) running in DDA Top10 mode. Based on their hydrophobicity, the tryptic peptides were separated using a chromatographic gradient of 60 min with a binary system of buffer A (0.1% formic acid) and buffer B (80% ACN, 0.1% formic acid) with a total flow of 250 nl/min. For the separation, in‐house made analytical columns (length: 50 cm, inner diameter: 75 μm) containing 2.7 μm C18 Poroshell EC120 beads (Agilent) that were heated to 50°C in a column oven (Sonation) were used. Over a time period of 41 min, Buffer B was linearly increased from 3% to 27% and then more rapidly up to 50% in 8 min. Finally, buffer B was increased to 95% within 1 min followed by 10 min at 95% to wash the analytical column. Full MS spectra (300–1,750 *m/z*) were accomplished with a resolution of 70,000, a maximum injection time of 20 ms and an AGC target of 3e6. In each full MS spectrum, the top 10 most abundant ions were selected for HCD fragmentation (NCE:27) with a quadrupole isolation width of 1.8 *m/z* and 10 s dynamic exclusion. The MS/MS spectra were then measured with a 35,000 resolution, an injection time of maximum 110 ms and an AGC target of 5e5.

The MS RAW files were then analyzed with MaxQuant suite (version 1.5.3.8) on standard settings with the before mentioned SILAC labels (Cox & Mann, 2008). By matching against the human UniProt database, the peptides were then identified using the Andromeda scoring algorithm (Cox *et al*, 2011). Carbamidomethylation of cysteine was defined as a fixed modification, while methionine oxidation and N‐terminal acetylation were variable modifications. The digestion protein was Trypsin/P. A false discovery rate (FDR) < 0.01 was used to identify peptide‐spectrum matches and to quantify the proteins. Data processing and statistical analysis was performed in the Perseus software (version 1.6.1.1) (Tyanova *et al*, 2016). Using the one‐sample *t*‐test the significantly changed proteins were identified (H0 = 0, fudge factor S0 = 0.2). Visualization was performed with RStudio (version 1.2.5033).

### Label‐free quantitative mass spectrometry

Twenty‐four hours before expression of the FLAG‐tagged constructs, the HEK293 WT cells were treated with Luciferase siRNA and the UPF3B KO clone 90 and UPF3 dKO clone 1 cells were treated with siRNAs targeting residual UPF3B. The expression of either FLAG‐GST or FLAG‐UPF2 in WT cells and FLAG‐UPF2 in the clones 90 and 1 was induced for 48 h with 30 µg/ml cumate. Lysis and sample preparation were performed as described above. MS analysis was performed as described above with a slightly adjusted gradient as followed: 3–30% B in 41 min, 30–50% B in 8 min, 50–95% B in 1 min, followed by 10 min washing at 95%. LFQ values were calculated using the MaxLFQ algorithm (Cox *et al*, 2014) in MaxQuant. Significantly changed proteins were identified by two‐sample *t‐*testing (fudge factor S0 = 0.2).

### Whole proteome mass spectrometry and Skyline analysis

The expression of FLAG‐UPF3A in HEK293 WT cells and was induced for 72 h with 30 µg/ml cumate. WT and UPF3A‐overexpressing cells were harvested in PBS and lysed in 300 µl SP3 lysis buffer. After sonication using the Bandelin Sonopuls mini20 with 15× 1 s pulses at 50% amplitude with a 2.5 mm tip, protein concentrations were measured using the Bradford assay. Samples were adjusted to 25 µg whole protein, reduced with 5 mM DTT and alkylated with 40 mM CAA. After a 10‐min‐centrifugation at 20,000 *g* the samples were handed to the proteomics core facility at CECAD.

Samples were analyzed on an UltiMate 3000 coupled to an Orbitrap Exploris 480 equipped with FAIMS Pro via a Nanospray Flex source (all Thermo Scientific). Peptides were loaded onto a precolumn cartridge (Acclaim PepMap 100) for two minutes with a loading pump flow of 15 µl/min. Afterwards, peptides were separated on the following gradient running 0.1% FA (buffer A) against 80% acetonitrile + 0.1% FA (buffer B) on a self‐packed 40 cm pulled tip column (75 µm ID, filled with Poroshell 120, Agilent): initial 5% B, up to 30% B in 107 min, up to 50% B in 20 min, up to 95% B in 1 min, followed by washing at 95% B and reequilibration to initial conditions. Peptides eluted from the column were analyzed in data‐dependent mode using three different FAIMS compensation voltages (CVs). On each CV, a survey scan was performed with a resolution of 60k and an injection time of 25 ms in the range of 350–1,400 *m/z*. Adjusted TopN cycles were used for each CV as followed: −45 CV, Top14; −60 CV, Top12; −75 CV, Top10. All MS2 isolation were performed in a 1.4 Th window, fragmentations were performed with a collision energy of 30% and MS2 scans were all recorded with a resolution of 15 k with a maximum injection time of 22 ms.

Afterward, spectra were split by CV to be analyzed in MaxQuant 2.0.3.0 using standard parameters. Samples stemming from one MS run were named identically in MaxQuant to facilitate proper quantifications following (Hebert *et al*, 2018). Spectra were searched against the human Uniprot reference proteome (UP5640) including isoforms. Match between run option was used over samples from each group. Afterward, results were used to perform MS1 extraction in Skyline 21.0 (Pino *et al*, 2020). Suitable quantifier peptides for relative quantification were chosen for UPF3A and UPF3B as well as two additional qualifier peptides. Chosen quantifiers were only observed on a single CV in all samples to ease ratio calculations. The same process was performed for actin (P60709) and tubulin (P68366) used as “loading controls” to normalize quantifier intensities of UPF3A and UPF3B prior to ratio calculation.

### TurboID proximity labelling

The proximity labelling procedure was performed almost identically as described before (Boehm *et al*, 2021). Stable UPF3B KO cell lines were seeded (2.5 × 10^6^ cells per 10 cm dish) and reverse transfected using 6.25 µl Lipofectamine RNAiMAX and 200 pmol siRNA (UPF3A). The expression of FLAG‐TurboID‐tagged UPF3B WT, mutants or control proteins was induced 48 h later with 30 µg/ml cumate. In this step, the medium was also changed to high‐glucose, GlutaMAX DMEM (Gibco) supplemented with 9% dialyzed fetal bovine serum (Gibco; A3382001; to suppress background biotinylation) (May *et al*, 2020) and 1× penicillin streptomycin (Gibco). Proximity labelling by biotinylation was performed on the next day by the addition of 50 µM biotin for 15 min. Afterward, the cells were washed twice with PBS on ice, scraped in 1 ml ice cold PBS, collected for 5 min at 100 *g* and 4°C, and finally resuspended in 200 µl phospho‐RIPA buffer (50 mM Tris pH 8.0, 150 mM NaCl, 1% IGEPAL CA 630, 0.5% deoxycholate, 0.1% SDS, 1 µg/ml RNase A) supplemented with one tablet of PhosSTOP (Roche), 100 µl EDTA‐free HALT Protease & Phosphatase Inhibitor (Thermo Fisher) and per 10 ml buffer. Samples were sonicated using the Bandelin Sonopuls mini20 with 10 pulses (2.5 mm tip, 1 s pulse, 50% amplitude). 50 µl input aliquots containing 100 µg of total protein were prepared and mixed with SDS‐sample buffer. Concentration‐adjusted lysates containing 1 mg total protein in 500 µl buffer were concentrated to approximately 100 µl in 0.5 ml Amicon Ultra centrifugal filter devices (3K cutoff) for 45 min at 4°C and 14,000 *g*, to minimize excess biotin in the sample. The concentrated sample was combined with 200 µl RIPA buffer (wash of centrifugal filter), mixed with 25 µl pre‐washed Pierce Streptavidin Magnetic Beads (Thermo Fisher) and incubated for 2 h at 4°C with overhead shaking. The beads were washed four times for 5 min with 800 µl RIPA buffer, followed by one wash with 800 µl mild wash buffer (20 mM HEPES‐KOH (pH 7.9), 137 mM NaCl, 2 mM MgCl_2_, 0.2% Triton X‐100, 0.1% NP‐40). Biotinylated proteins were eluted first by incubating for 15 min @ RT with 50 µl 5% SDS, supplemented with 20 mM DTT and 3 mM biotin, then for 15 min at 96°C, followed by another elution with 25 µl at both temperatures and the two eluates were combined. After 30 min of incubation at 55°C, alkylation was performed by adding 8.5 µl of 400 mM CAA (final concentration of 40 mM) to the eluates and another 30 min incubation step in the dark. Tryptic protein digestion and proteomics analysis was performed as described above.

### Northern blotting

For stable cell lines with the globin WT or PTC39 reporter integrated via the PiggyBac system, expression was induced with 30 µg/ml cumate. In case of the dual expression, the Flp‐In T‐REx reporter constructs were induced with 1 µg/ml doxycycline and the UPF3B rescue constructs with 30 µg/ml cumate.

The cells were harvested in RNAsolv reagent and total RNA extraction was performed as described above. 3.0 µg total RNA were resolved on a 1% agarose/0.4 M formaldehyde gel using the tricine/triethanolamine buffer system (Mansour & Pestov, 2013). Next a transfer on a nylon membrane (Roth) in 10× SSC followed. The blot was incubated overnight at 65°C in Church buffer containing [α‐^32^P]‐GTP body‐labeled RNA‐probes for mRNA reporter detection (Voigt *et al*, 2019). Ethidium bromide stained 28S and 18S rRNA served as loading controls. RNA signal detected with the Typhoon FLA 7000 (GE Healthcare) was quantified in a semi‐automated manner using the ImageQuant TL 1D software with a rolling‐ball background correction. EtBr‐stained rRNA bands were quantified with the Image Lab 6.0.1 software (Bio‐Rad). Signal intensities were normalized to the internal control (rRNA) before calculation of mean values. The control condition was set to unity (TPI WT for reporter assays), quantification results are shown as data points and mean.

### Protein conservation

UPF3A (UniProt ID: Q9H1J1‐1) and UPF3B (UniProt ID: Q9BZI7‐2) protein sequences were aligned using Clustal Omega (https://www.ebi.ac.uk/Tools/msa/clustalo/) (Goujon *et al*, 2010; Sievers *et al*, 2011), viewed using Jalview (Waterhouse *et al*, 2009), the conservation score extracted and used for visualization.

### Data presentation

Quantifications and calculations for other experiments were performed—if not indicated otherwise—with Microsoft Excel (version 1808) or R (version 4.0.4) and all plots were generated using IGV (version 2.8.12), GraphPad Prism 5, ggplot2 (version 3.3.3), or ComplexHeatmap (version 2.6.2) (Gu *et al*, 2016).

## Author contributions

**Damaris Wallmeroth:** Conceptualization; Investigation; Visualization; Methodology; Writing—original draft; Writing—review & editing. **Jan‐Wilm Lackmann:** Resources; Data curation; Investigation. **Sabrina Kueckelmann:** Investigation. **Janine Altmüller:** Resources; Data curation. **Christoph Dieterich:** Funding acquisition. **Volker Boehm:** Conceptualization; Resources; Data curation; Software; Supervision; Investigation; Visualization; Methodology; Writing ‐ original draft; Writing—review & editing. **Niels H Gehring:** Conceptualization; Supervision; Funding acquisition; Methodology; Writing ‐ original draft; Writing—review & editing.

In addition to the CRediT author contributions listed above, the contributions in detail are:

Conceptualization: NHG, VB, DW; Methodology: VB, DW, NHG; Software: VB; Investigation: DW, VB, J‐WL, SK; Resources and Data Curation: VB, JA, J‐WL; Writing—Original Draft, Review, and Editing: DW, VB, NHG; Visualization: VB, DW; Supervision: NHG, VB; Funding Acquisition: NHG, CD.

## Disclosure of competing interests statement

The authors declare that they have no conflict of interest.

## Supporting information



Appendix

Expanded View Figures PDF

Dataset EV1

Dataset EV2

Dataset EV3

Dataset EV4

Dataset EV5

Dataset EV6

Dataset EV7

Source Data for Expanded View and Appendix

Source Data for Figure 1

Source Data for Figure 2

Source Data for Figure 3

Source Data for Figure 4

Source Data for Figure 6

Source Data for Figure 7

## Data Availability

The datasets and computer code produced in this study are available in the following databases:
RNA‐Seq data for UPF3B KO samples: ArrayExpress E‐MTAB‐10711 (https://www.ebi.ac.uk/arrayexpress/experiments/E‐MTAB‐10711) RNA‐Seq data for UPF3 dKO samples: ArrayExpress E‐MTAB‐10716 (https://www.ebi.ac.uk/arrayexpress/experiments/E‐MTAB‐10716) RNA‐Seq data for UPF3A KO/OE samples: ArrayExpress E‐MTAB‐10718 (https://www.ebi.ac.uk/arrayexpress/experiments/E‐MTAB‐10718) RNA‐Seq data for HeLa UPF3A OE samples: ArrayExpress E‐MTAB‐11184 (https://www.ebi.ac.uk/arrayexpress/experiments/E‐MTAB‐11184) Mass spectrometry proteomics data for UPF2 SILAC and label‐free mass spectrometry: PRIDE PXD027120 (https://www.ebi.ac.uk/pride/archive/projects/PXD027120) Mass spectrometry proteomics data for UPF3B TurboID mass spectrometry: PRIDE PXD029898 (https://www.ebi.ac.uk/pride/archive/projects/PXD029898) Mass spectrometry proteomics data for whole proteome mass spectrometry: PRIDE PXD032337 (https://www.ebi.ac.uk/pride/archive/projects/PXD032337) Codes used in this study: GitHub (https://github.com/boehmv/UPF3) All relevant data supporting the key findings of this study are available within the article and its Expanded View files or from the corresponding author upon reasonable request.
