# Supplementary material for: Human UPF3A and UPF3B enable fault‐tolerant activation of nonsense‐mediated mRNA decay
Source: EMBO J. 2022 Apr 22;41(10):e109191. doi: 10.15252/embj.2021109191 (PMC9108619; doi:10.15252/embj.2021109191)
Supplement: Supplementary file 1 — Appendix [file EMBJ-41-e109191-s009.pdf]

Appendix Figures

Human UPF3A and UPF3B enable fault-tolerant activation of nonsense-mediated mRNA decay

Damaris Wallmeroth[1,2], Jan-Wilm Lackmann[3], Sabrina Kueckelmann[1,2], Janine Altmüller[4,5], Christoph Dieterich[6,7], Volker Boehm[1,2], Niels H. Gehring[1,2]

1 Institute for Genetics, University of Cologne, 50674 Cologne, Germany

2 Center for Molecular Medicine Cologne (CMMC), University of Cologne, 50937 Cologne, Germany

3 CECAD Research Center, University of Cologne, Joseph-Stelzmann-Str. 26, 50931 Cologne, Germany

4 Cologne Center for Genomics (CCG), University of Cologne, 50931 Cologne, Germany

5 Present address: Berlin Institute of Health at Charité – Universitätsmedizin Berlin, Core Facility Genomics, Charitéplatz 1, 10117 Berlin, Germany and Max Delbrück Center for Molecular Medicine in the Helmholtz Association (MDC), Berlin, Germany

6 Section of Bioinformatics and Systems Cardiology, Department of Internal Medicine III and Klaus Tschira Institute for Integrative Computational Cardiology, Heidelberg University Hospital, 69120 Heidelberg, Germany

7 DZHK (German Centre for Cardiovascular Research), Partner site Heidelberg/Mannheim, 69120 Heidelberg, Germany

***Correspondence:** boehmv@uni-koeln.de **(V.B.),** ngehring@uni-koeln.de **(N.H.G.)**

<u>Table of contents</u>

Appendix figure S1 - Total numbers of up-/downregulated differentially expressed genes or differentially used transcripts 2

Appendix Figure S2 - UPF3 dKOs express residual amounts of UPF3B........ 3

Appendix Figure S3 - No EJC interaction in the absence of both UPF3 paralogs 4

Appendix Figure S4 - Overview of differential transcript expression in the RNA-seq datasets 5

Appendix Figure S5 - Schematic representation of the combinatorial UPF3B domain functions........ 6

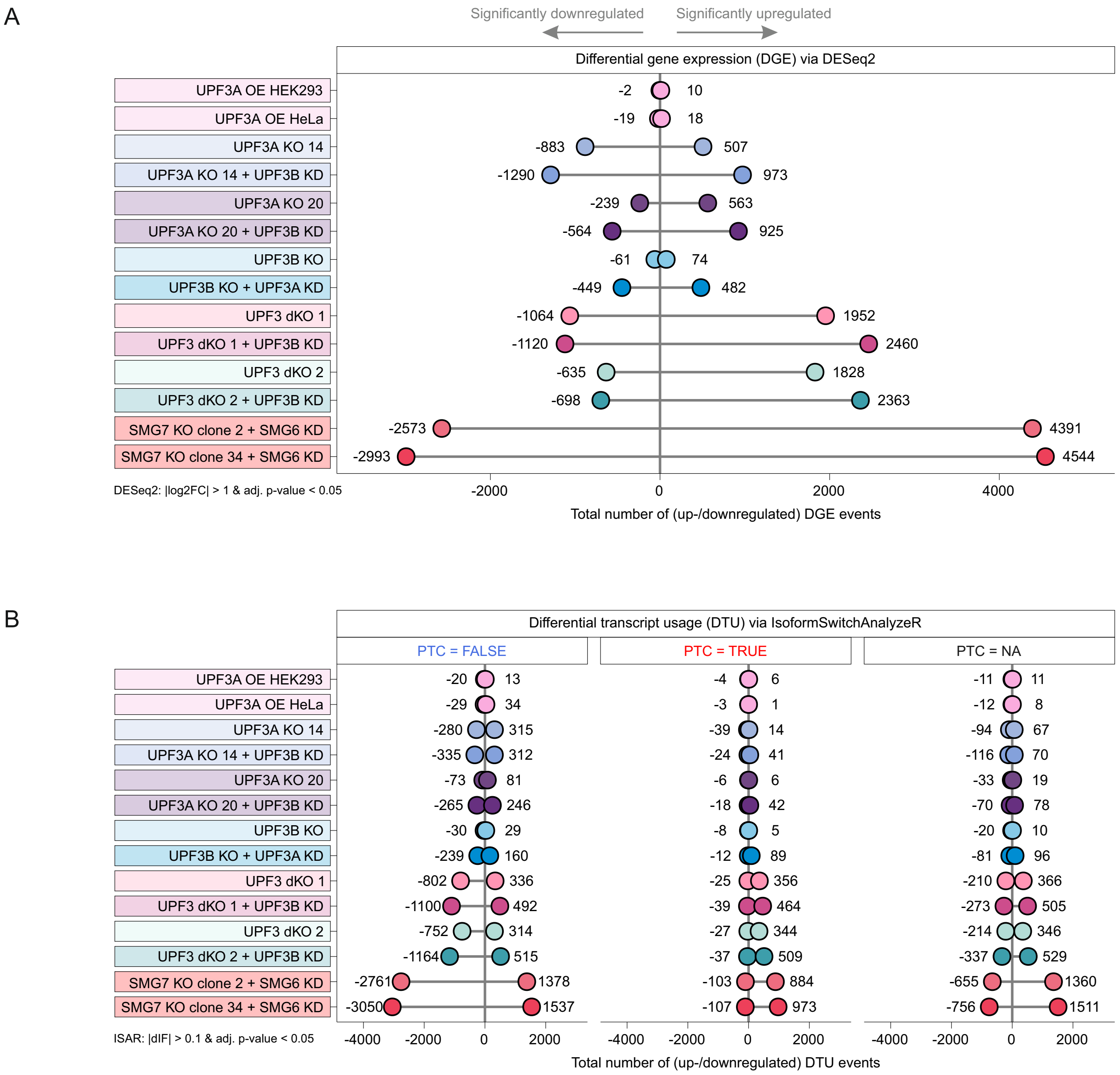

Appendix Figure S1 - Total numbers of up-/downregulated differentially expressed genes or differentially used transcripts
A,B The lollipop plots show the total number of upregulated (pointing to the right, positive numbers) and downregulated (pointing to the left, negative numbers) genes (**A**) or isoform switches (**B**) that were detected in the respective condition with the indicated cutoffs. For (**B**), the PTC status (TRUE = PTC-containing transcripts, FALSE = non-PTC-containing transcript, NA = no annotated ORF) was used to generate separate plots. Two HEK293 SMG7 KO clones + SMG6 KD served as positive control for strong NMD inhibition.

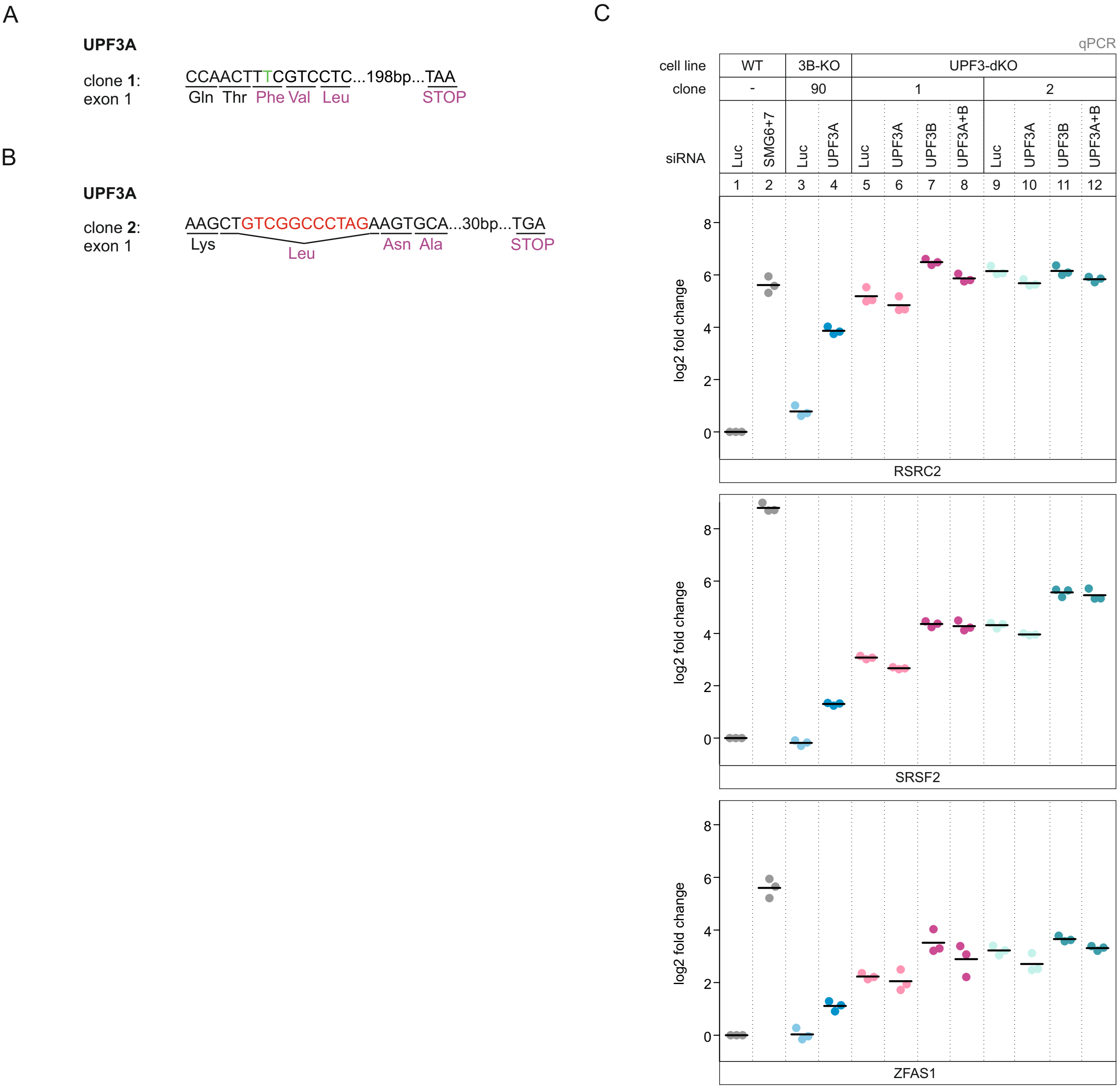

Appendix Figure S2 - UPF3 dKOs express residual amounts of UPF3B
A,B The targeted exon region and anticipated PTC location following insertions (green) or deletions (red) are indicated for detected alleles of UPF3A in dKO clone 1 (A) and 2 (B). The UPF3B genomic locus is shown in fig EV3A.
C Quantitative RT-PCR of the indicated cell lines with the indicated KDs. For RSRC2 and SRSF2 the ratio of NMD isoform to canonical isoform was calculated. ZFAS1 expression was normalized to C1orf43 reference. Data points and means are plotted as log2 fold change (n=3).

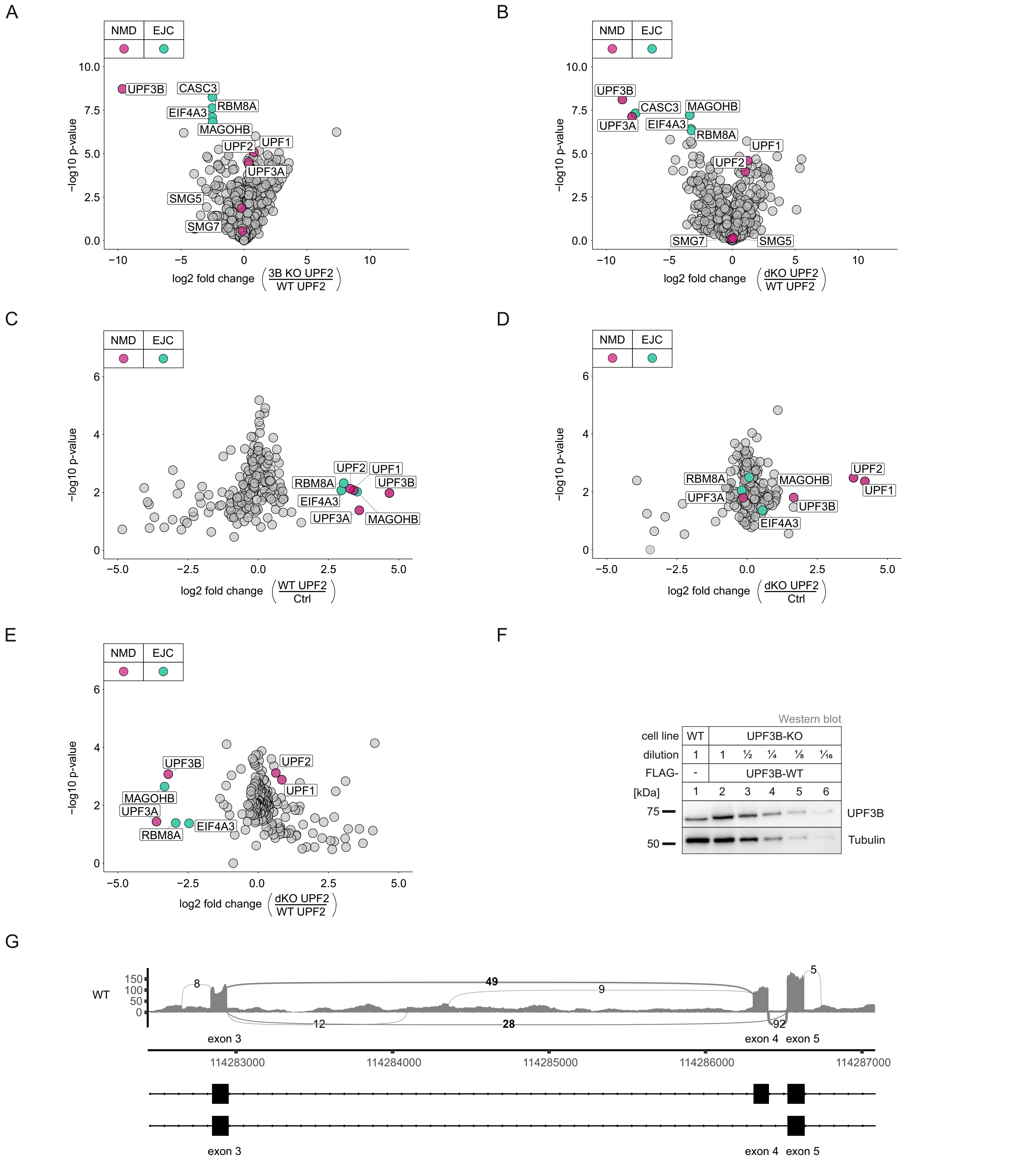

Appendix Figure S3 - No EJC interaction in the absence of both UPF3 paralogs

A,B Volcano plots of label free mass spectrometry-based analysis of the interaction partners of UPF2 in WT cells treated with control siRNAs and the UPF3B KO clone 90 and dKO clone 1 both treated with siRNAs targeting UPF3B (n = 4). **(A)** FLAG-UPF2 in UPF3B KOs against FLAG-UPF2 control in WT cells, **(B)** FLAG-UPF2 in UPF3 dKOs against FLAG-UPF2 control in WT cells.

C-E Volcano plots of SILAC mass spectrometry-based analysis of the interaction partners of FLAG-UPF2 in WT cells and in the UPF3 dKO cell line 2 (n=3). **(C)** FLAG-UPF2 against FLAG-GST control in WT cells, **(D)** FLAG-UPF2 against FLAG-GST control in dKO cells, **(E)** FLAG-UPF2 in dKO cells against UPF2 in WT cells.

F Western blot of WT cells and a dilution series of UPF3B KO cells expressing a UPF3B WT construct to determine exogenous expression levels. Construct expression is around 3 times higher than endogenous UPF3B.

G (top) Sashimi plot visualizing alternatively spliced exon and flanking exons of UPF3A in WT HEK 293 cells. Per-base expression is plotted on y-axis, genomic coordinates on x-axis. Number of junction reads are the means of three replicates (cut-off > 5). (bottom) mRNA isoforms with exons in black and introns as lines with points.

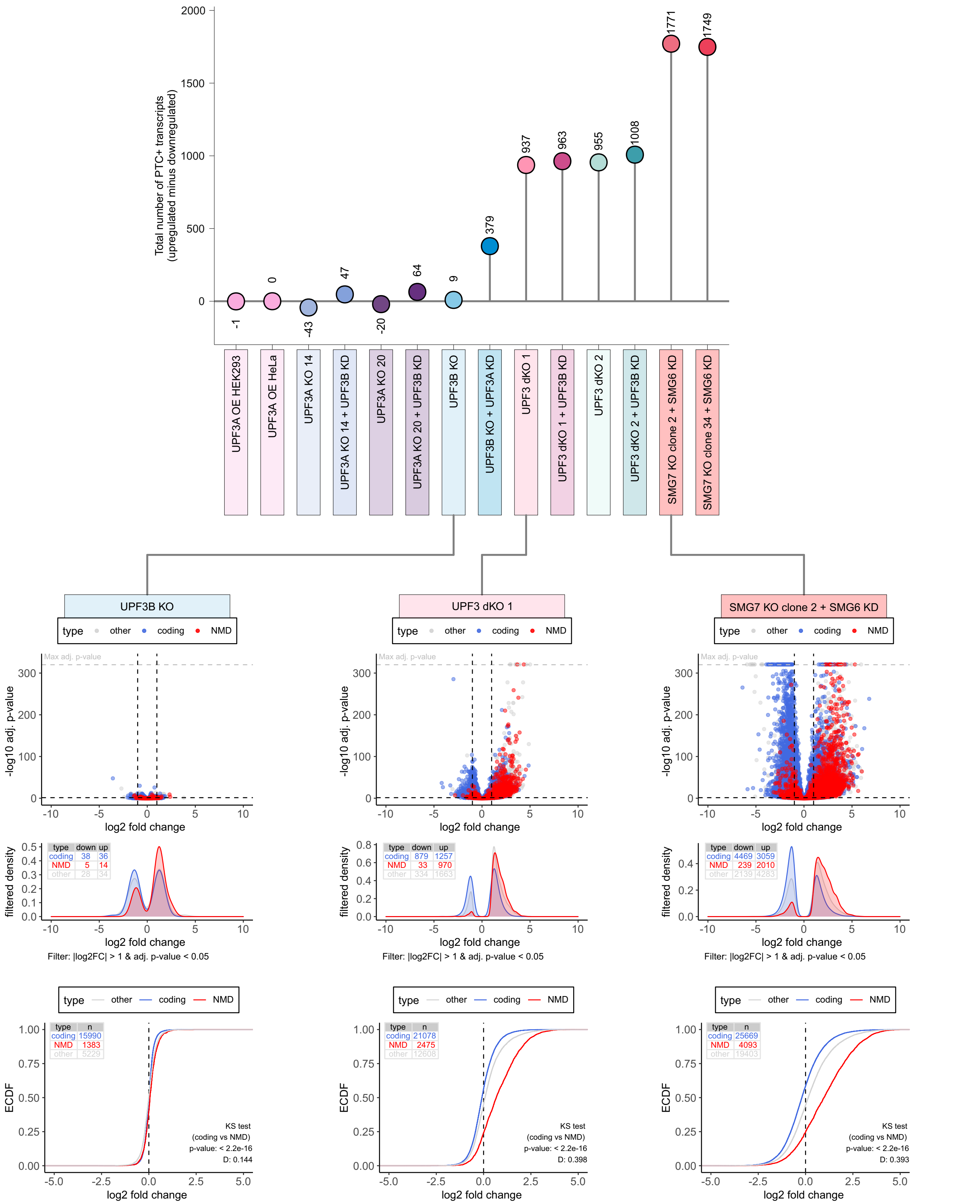

Appendix Figure S4 - Overview of differential transcript expression in the RNA-seq datasets
The lollipop plot (top) shows the total number of upregulated minus downregulated PTC-positive transcripts, based on a DESeq2 differential transcript expression (DTE) analysis, with the cutoffs |log2FoldChange| > 1 & adj. p-value < 0.05. Volcano and ECDF plots of selected conditions are shown below. Transcripts are classified according to their annotated biotypes: with an annotated PTC (red, NMD), protein coding transcripts with regular stop codon (blue, coding) and all other biotypes (grey, other). KS test = Kolmogorow-Smirnow test.

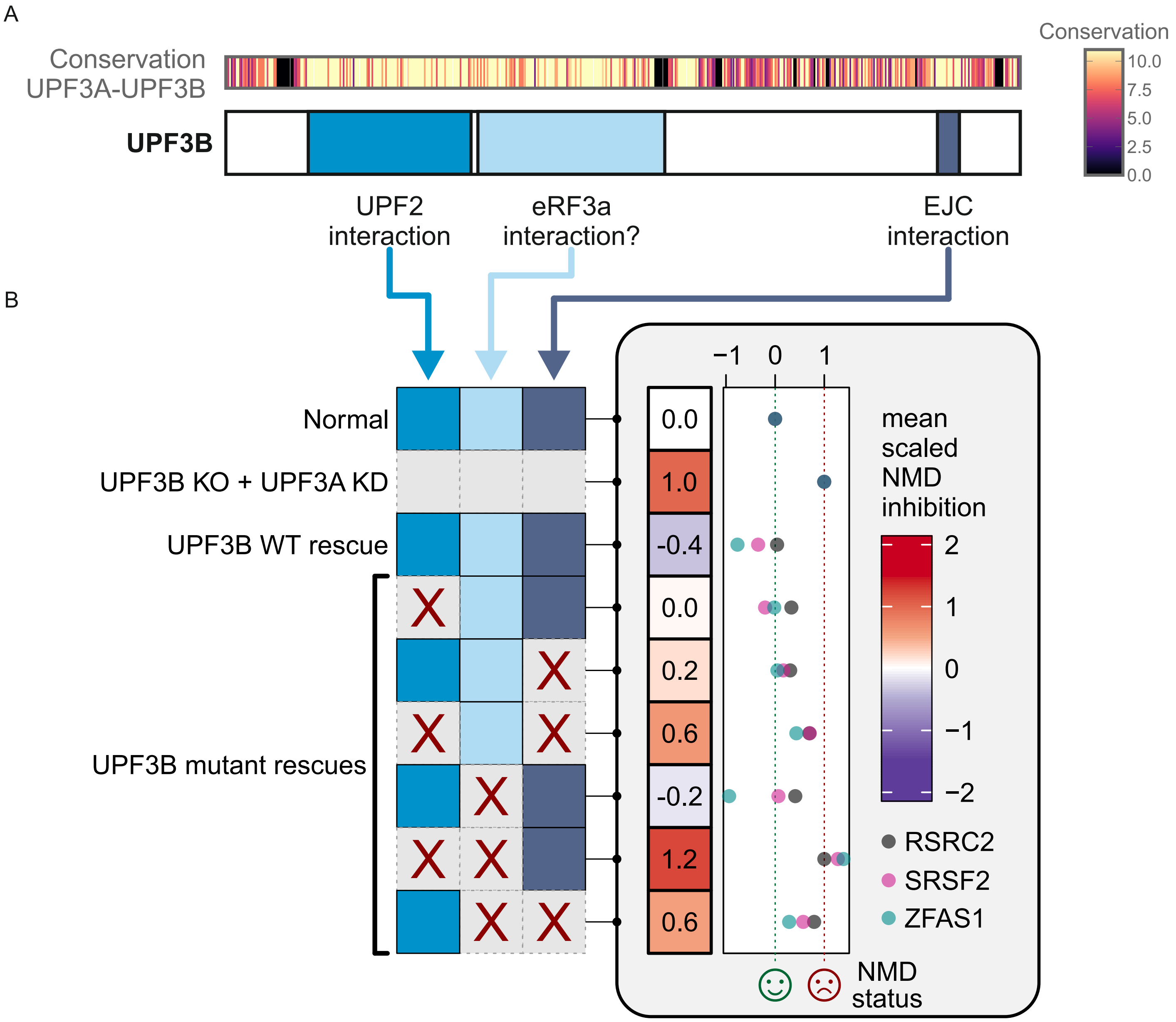

Appendix Figure S5 - Schematic representation of the combinatorial UPF3B domain functions.

A Schematic overview of UPF3B domain structure and the postulated functions. Conservation between UPF3A (UniProt ID: Q9H1J1-1) and UPF3B (UniProt ID: Q9BZI7-2) proteins as calculated via Clustal Omega and Jalview is shown.

B Impact on NMD activity by isolated or combined mutations of UPF3B interaction surfaces or domains is depicted schematically. The quantitative RT-PCR data of the UPF3B rescues for the targets RSRC2, SRSF2 and ZFAS1 (Fig. 6G) was used to determine the extend of NMD inhibition. For each target, the mean log2 fold changes of control (set as 0) and UPF3B KO + UPF3A KD (set as 1) were used to scale all other data. The mean scaled NMD inhibition is plotted individually for each target on the right and shown as overall mean in the heatmap. The combination of UPF3B mutations is depicted on the left.
