# Supplementary material for: Human UPF3A and UPF3B enable fault‐tolerant activation of nonsense‐mediated mRNA decay
Source: EMBO J. 2022 Apr 22;41(10):e109191. doi: 10.15252/embj.2021109191 (PMC9108619; doi:10.15252/embj.2021109191)
Supplement: Supplementary file 2 — Expanded View Figures PDF [file EMBJ-41-e109191-s012.pdf]

Expanded View Figures

Figure EV1. UPF3A overexpression does not cause upregulation of NMD-targets.

A Western blot analysis of unaltered HEK293 WT cells or with induced FLAG-UPF3A or FLAG-UPF3B expression. UPF3A, UPF3B, and FLAG levels were detected. Tubulin serves as control. Protein levels were quantified, normalized to tubulin expression, and shown as datapoints and mean ($n = 4$). Fold-changes of relevant conditions are shown.

B Skyline analysis of WT and UPF3A-overexpressing cells after whole proteome mass spec analysis. Quantifier intensities of UPF3A (left) and UPF3B (right) were normalized to actin (top) and tubulin (bottom) which were used as “loading controls”. Results are shown as datapoints and mean ($n = 4$). The means were used to calculate the respective fold-changes.

C Schematic overview of the analysis pipeline.

D Heatmap of mean log2 fold changes (log2FC) of previously reported UPF3A-responsive NMD targets (Fig 3C of Shum *et al*, 2016) as determined by DESeq2 using the indicated RNA-Seq data. The data from SMG7 KO with SMG6 KD (Data ref: Boehm *et al*, 2021) serve as positive control for NMD inhibition.

E Volcano plot showing the differential transcript usage (via IsoformSwitchAnalyzeR) in RNA-Seq data of HEK293 and HeLa WT cells overexpressing UPF3A. Isoforms containing GENCODE (release 33) annotated PTC (red, TRUE), regular stop codons (blue, FALSE) or having no annotated open reading frame (gray, NA) are indicated. The change in isoform fraction (dIF) is plotted against the -log10 adjusted *P*-value (P_{adj}). Density plots show the distribution of filtered isoforms in respect to the dIF, cutoffs were $|dIF| > 0.1$ and $P_{adj} < 0.05$. *P*-values were calculated by IsoformSwitchAnalyzeR using a DEXSeq-based test and corrected for multiple testing using the Benjamini–Hochberg method. OE = overexpression.

Source data are available online for this figure.

 © 2022 The Authors

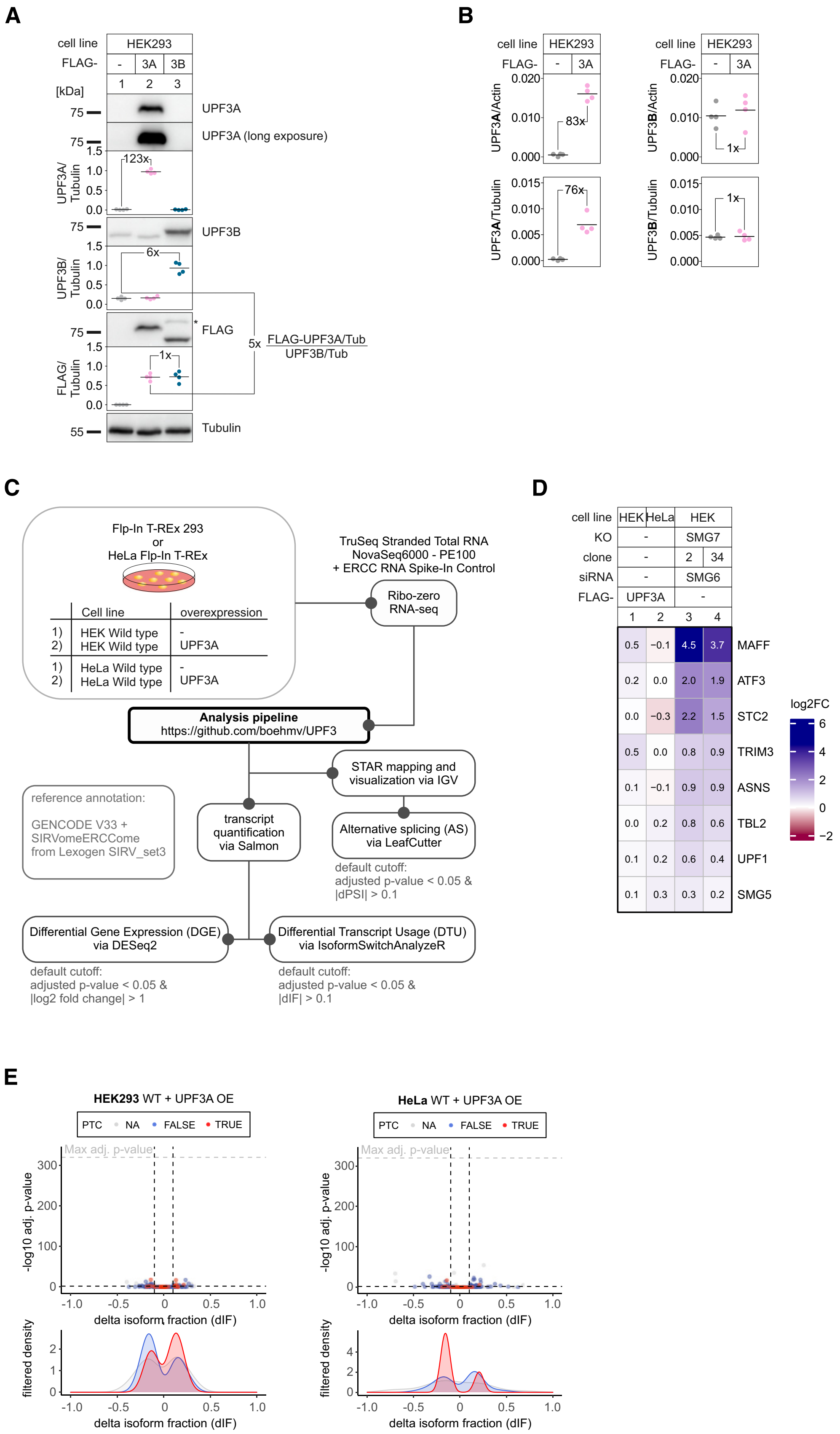

A
cell line HEK293
FLAG- - 3A 3B
1 2 3
[kDa]
75 UPF3A
75 UPF3A (long exposure)
UPF3A/ Tubulin
123x
UPF3B
UPF3B/ Tubulin
6x
FLAG
FLAG/ Tubulin
1x
5x FLAG-UPF3A/Tub UPF3B/Tub
55 Tubulin
B
cell line HEK293
FLAG- - 3A
UPF3A/Actin
83x
UPF3B/Actin
1x
UPF3A/Tubulin
76x
UPF3B/Tubulin
1x
C
Flp-In T-REx 293 or HeLa Flp-In T-REx
Cell line overexpression
1) HEK Wild type -
2) HEK Wild type UPF3A
1) HeLa Wild type -
2) HeLa Wild type UPF3A
TruSeq Stranded Total RNA NovaSeq6000 - PE100 + ERCC RNA Spike-In Control
Ribo-zero RNA-seq
Analysis pipeline https://github.com/boehmv/UPF3
reference annotation: GENCODE V33 + SIRVomeERCCome from Lexogen SIRV_set3
transcript quantification via Salmon
STAR mapping and visualization via IGV
Alternative splicing (AS) via LeafCutter
default cutoff: adjusted p-value < 0.05 & |dPSI| > 0.1
Differential Gene Expression (DGE) via DESeq2
default cutoff: adjusted p-value < 0.05 & |log2 fold change| > 1
Differential Transcript Usage (DTU) via IsoformSwitchAnalyzeR
default cutoff: adjusted p-value < 0.05 & |dIF| > 0.1
D
cell line HEK HeLa HEK
KO - SMG7
clone - 2 34
siRNA - SMG6
FLAG- UPF3A -
1 2 3 4
MAFF 0.5 −0.1 4.5 3.7
ATF3 0.2 0.0 2.0 1.9
STC2 0.0 −0.3 2.2 1.5
TRIM3 0.5 0.0 0.8 0.9
ASNS 0.1 −0.1 0.9 0.9
TBL2 0.0 0.2 0.8 0.6
UPF1 0.1 0.2 0.6 0.4
SMG5 0.1 0.3 0.3 0.2
log2FC
E
HEK293 WT + UPF3A OE
HeLa WT + UPF3A OE
PTC NA FALSE TRUE
Max adj. p-value
-log10 adj. p-value
delta isoform fraction (dIF)
filtered density

Figure EV1.

Figure EV2. Light NMD inhibition after UPF3B KD in UPF3A KO cells.

A PCR amplification of targeted genomic locus of UPF3A for Sanger sequencing analysis.

B, C The targeted exon region and anticipated PTC location following insertions (green) or deletions (red) are indicated for detected alleles of UPF3A in clone 14 (B) and 20 (C).

D Western blot analysis of WT and UPF3A KO cells (clones 14 and 20) with or without expression of FLAG-tagged UPF3A rescue construct. UPF3A and UPF3B protein levels were detected, Tubulin serves as control ($n = 1$).

E Quantitative RT–PCR of the samples from (D). Expression of four targets with significant DGE in both UPF3A KO clones (ZFAS1, BCHE, AMOT, CTSZ) was normalized to C1orf43 reference. For PHF21B and DDIT3, DGE analysis showed significant downregulation in both UPF3A KO clones and upregulation in SMG7 KO + SMG6 KD and UPF3 dKO cells. Expression was normalized to EMC7 reference. Data points and means are plotted as log2 fold change (log2FC) ($n = 3$).

F Schematic overview of the analysis pipeline.

G, H Volcano plots showing the differential transcript usage (via IsoformSwitchAnalyzeR) in various RNA-Seq data. Isoforms containing GENCODE (release 33) annotated PTC (red, TRUE), regular stop codons (blue, FALSE) or having no annotated open reading frame (gray, NA) are indicated. The change in isoform fraction (dIF) is plotted against the -log10 adjusted *P*-value (P_{adj}). Density plots show the distribution of filtered isoforms in respect to the dIF, cutoffs were $|dIF| > 0.1$ and $P_{adj} < 0.05$. *P*-values were calculated by IsoformSwitchAnalyzeR using a DEXSeq-based test and corrected for multiple testing using the Benjamini–Hochberg method.

I Volcano plots showing the differential gene expression analyses from the indicated RNA-Seq datasets. The log2 fold change is plotted against the -log10 adjusted *P*-value (P_{adj}). *P*-values were calculated by DESeq2 using a two-sided Wald test and corrected for multiple testing using the Benjamini–Hochberg method.

Source data are available online for this figure.

© 2022 The Authors

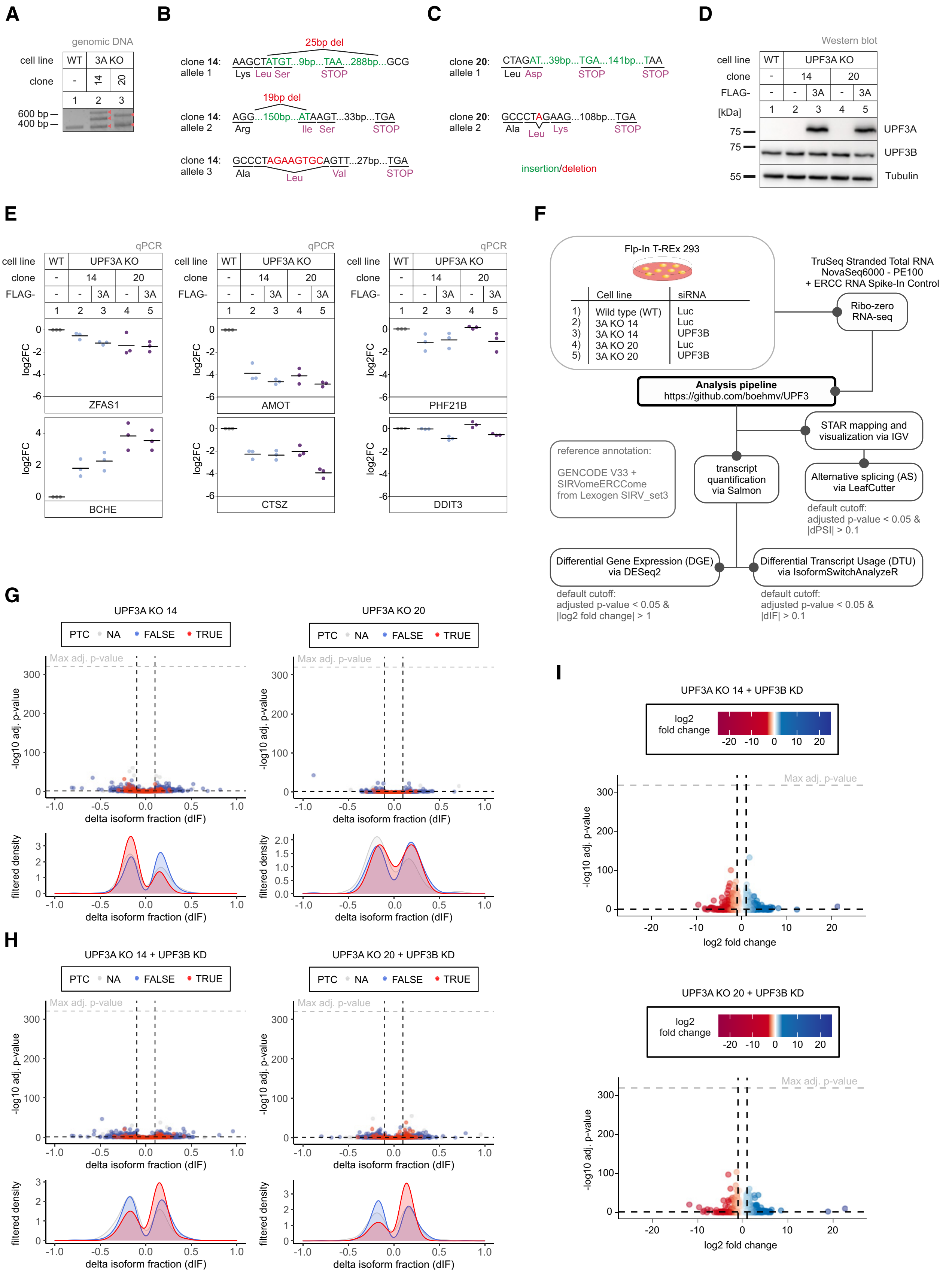
A
genomic DNA
cell line WT 3A KO
clone - 14 20
1 2 3
600 bp
400 bp
B
25bp del
clone 14: allele 1 AAGCTATGT...9bp...TAA...288bp...GCG
Lys Leu Ser STOP
19bp del
clone 14: allele 2 AGG...150bp...ATAAGT...33bp...TGA
Arg Ile Ser STOP
clone 14: allele 3 GCCCTAGAAGTGCAGTT...27bp...TGA
Ala Leu Val STOP
C
clone 20: allele 1 CTAGAT...39bp...TGA...141bp...TAA
Leu Asp STOP STOP
clone 20: allele 2 GCCCTAGAAG...108bp...TGA
Ala Leu Lys STOP
insertion/deletion
D
Western blot
cell line WT UPF3A KO
clone - 14 20
FLAG- - - 3A - 3A
[kDa] 1 2 3 4 5
75 UPF3A
75 UPF3B
55 Tubulin
E
qPCR
cell line WT UPF3A KO
clone - 14 20
FLAG- - - 3A - 3A
1 2 3 4 5
log2FC
ZFAS1
AMOT
PHF21B
BCHE
CTSZ
DDIT3
F
Flp-In T-REx 293
Cell line siRNA
1) Wild type (WT) Luc
2) 3A KO 14 Luc
3) 3A KO 14 UPF3B
4) 3A KO 20 Luc
5) 3A KO 20 UPF3B
TruSeq Stranded Total RNA
NovaSeq6000 - PE100
+ ERCC RNA Spike-In Control
Ribo-zero RNA-seq
Analysis pipeline
https://github.com/boehmv/UPF3
STAR mapping and visualization via IGV
reference annotation:
GENCODE V33 + SIRVomeERCCome from Lexogen SIRV_set3
transcript quantification via Salmon
Alternative splicing (AS) via LeafCutter
default cutoff: adjusted p-value < 0.05 & |dPSI| > 0.1
Differential Gene Expression (DGE) via DESeq2
default cutoff: adjusted p-value < 0.05 & |log2 fold change| > 1
Differential Transcript Usage (DTU) via IsoformSwitchAnalyzeR
default cutoff: adjusted p-value < 0.05 & |dIF| > 0.1
G
UPF3A KO 14
UPF3A KO 20
PTC NA FALSE TRUE
Max adj. p-value
-log10 adj. p-value
delta isoform fraction (dIF)
filtered density
H
UPF3A KO 14 + UPF3B KD
UPF3A KO 20 + UPF3B KD
I
UPF3A KO 14 + UPF3B KD
UPF3A KO 20 + UPF3B KD
log2 fold change
-20 -10 0 10 20
Max adj. p-value
-log10 adj. p-value
log2 fold change

Figure EV2.

Figure EV3. No NMD inhibition by UPF3A in the absence of UPF3B.

A, B The targeted exon region and anticipated PTC location following insertions (green) or deletions (red) are indicated for detected alleles of UPF3B in KO clone 90 (A) and 91 (B).

C Schematic overview of the analysis pipeline.

D, E Read coverage of RSRC2 (D) and SRSF2 (E) from the indicated RNA-seq sample data with or without UPF3A siRNA treatment shown as Integrative Genomics Viewer (IGV) snapshot. The canonical and NMD-sensitive isoforms are schematically indicated below.

F, G Volcano plots showing the differential gene expression analyses from the indicated RNA-Seq datasets. The log2 fold change is plotted against the -log10 adjusted *P*-value (P_{adj}). *P*-values were calculated by DESeq2 using a two-sided Wald test and corrected for multiple testing using the Benjamini–Hochberg method.

H Western blot analysis of HEK293 and HeLa WT and UPF3B KO cells (clone 90 for HEK293 cells) with or without expression of FLAG-tagged UPF3A rescue construct ($n = 1$). UPF3A, UPF3B and FLAG protein levels were detected, Tubulin serves as control. The asterisk indicates unspecific bands.

I Quantitative RT–PCR of the HEK293 and HeLa samples from (H). For RSRC2 and SRSF2 the ratio of NMD isoform to canonical isoform was calculated. ZFAS1 expression was normalized to C1orf43 reference. Data points and means are plotted as log2 fold change (log2FC) ($n = 3$).

Source data are available online for this figure.

© 2022 The Authors

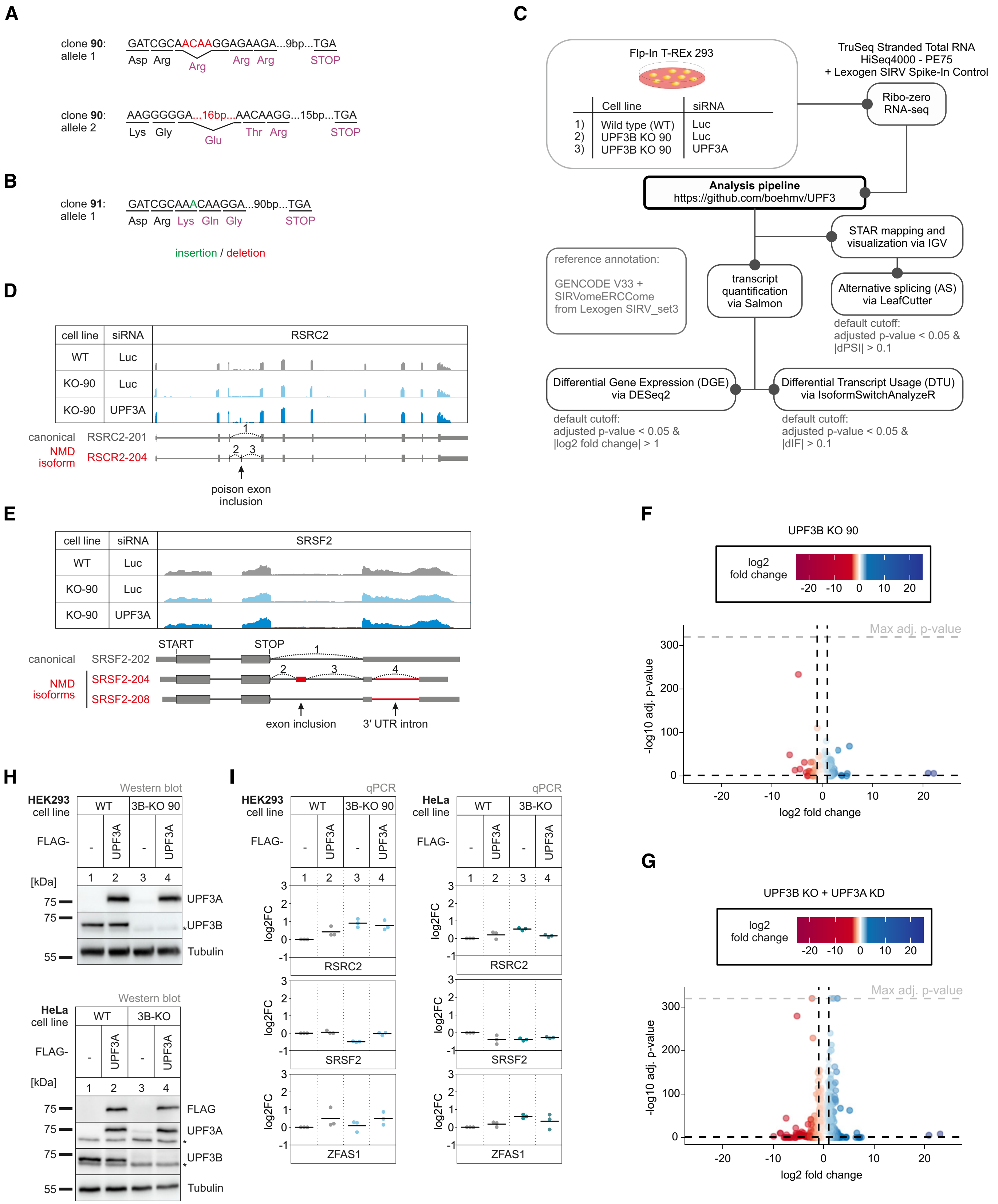

A
clone 90: allele 1
GATCGCAACAAGGAGAAGA...9bp...TGA
Asp Arg Arg Arg Arg STOP
clone 90: allele 2
AAGGGGGA...16bp...AACAAGG...15bp...TGA
Lys Gly Glu Thr Arg STOP
B
clone 91: allele 1
GATCGCAAACAAGGA...90bp...TGA
Asp Arg Lys Gln Gly STOP
insertion / deletion
C
Flp-In T-REx 293
Cell line
siRNA
1) Wild type (WT) Luc
2) UPF3B KO 90 Luc
3) UPF3B KO 90 UPF3A
TruSeq Stranded Total RNA
HiSeq4000 - PE75
+ Lexogen SIRV Spike-In Control
Ribo-zero RNA-seq
Analysis pipeline
https://github.com/boehmv/UPF3
STAR mapping and visualization via IGV
reference annotation:
GENCODE V33 + SIRVomeERCCome from Lexogen SIRV_set3
transcript quantification via Salmon
Alternative splicing (AS) via LeafCutter
default cutoff: adjusted p-value < 0.05 & |dPSI| > 0.1
Differential Gene Expression (DGE) via DESeq2
default cutoff: adjusted p-value < 0.05 & |log2 fold change| > 1
Differential Transcript Usage (DTU) via IsoformSwitchAnalyzeR
default cutoff: adjusted p-value < 0.05 & |dIF| > 0.1
D
cell line siRNA RSRC2
WT Luc
KO-90 Luc
KO-90 UPF3A
canonical RSRC2-201
NMD isoform RSCR2-204
poison exon inclusion
E
cell line siRNA SRSF2
WT Luc
KO-90 Luc
KO-90 UPF3A
START STOP
canonical SRSF2-202
NMD isoforms SRSF2-204 SRSF2-208
exon inclusion
3' UTR intron
F
UPF3B KO 90
log2 fold change
Max adj. p-value
-log10 adj. p-value
log2 fold change
G
UPF3B KO + UPF3A KD
log2 fold change
Max adj. p-value
-log10 adj. p-value
log2 fold change
H
Western blot
HEK293 cell line WT 3B-KO 90
FLAG- - UPF3A - UPF3A
[kDa] 1 2 3 4
75 UPF3A
75 UPF3B
55 Tubulin
HeLa cell line WT 3B-KO
FLAG- - UPF3A - UPF3A
[kDa] 1 2 3 4
75 FLAG
75 UPF3A
75 UPF3B
55 Tubulin
I
qPCR
HEK293 cell line WT 3B-KO 90
HeLa cell line WT 3B-KO
FLAG- - UPF3A - UPF3A
log2FC
RSRC2
SRSF2
ZFAS1

Figure EV3.

Figure EV4. Genes upregulated in UPF3 dKO cells are high confidence NMD targets.

A Schematic overview of the analysis pipeline.

B nVenn Diagrams showing the overlap of upregulated (upper panel) or downregulated genes (lower panel) in the UPF3 dKO cell lines 1 and 2, both with and without a supportive UPF3B KD. Log2 fold change < -1 (downregulated) or > 1 (upregulated) and adjusted *P*-value (P_{adj}) < 0.05. DGE = Differential Gene Expression.

C nVenn Diagram showing the overlap of upregulated genes in the two UPF3 dKO clones and previously analyzed SMG7 KO cells with SMG6 KD (Data ref.: Boehm *et al*, 2021) as control for cells with inhibited NMD. The overlap demonstrates high-confidence NMD targets. Cut-offs: log2FoldChange > 1 and adjusted *P*-value (P_{adj}) < 0.05. DGE = Differential Gene Expression.

D Volcano plots showing the differential transcript usage (via IsoformSwitchAnalyzeR) in various RNA-Seq data. Isoforms containing GENCODE (release 33) annotated PTC (red, TRUE), regular stop codons (blue, FALSE) or having no annotated open reading frame (gray, NA) are indicated. The change in isoform fraction (dIF) is plotted against the -log10 adjusted *P*-value (P_{adj}). Density plots show the distribution of filtered isoforms in respect to the dIF, cutoffs were $|dIF| > 0.1$ and $P_{adj} < 0.05$. *P*-values were calculated by IsoformSwitchAnalyzeR using a DEXSeq-based test and corrected for multiple testing using the Benjamini–Hochberg method.

© 2022 The Authors

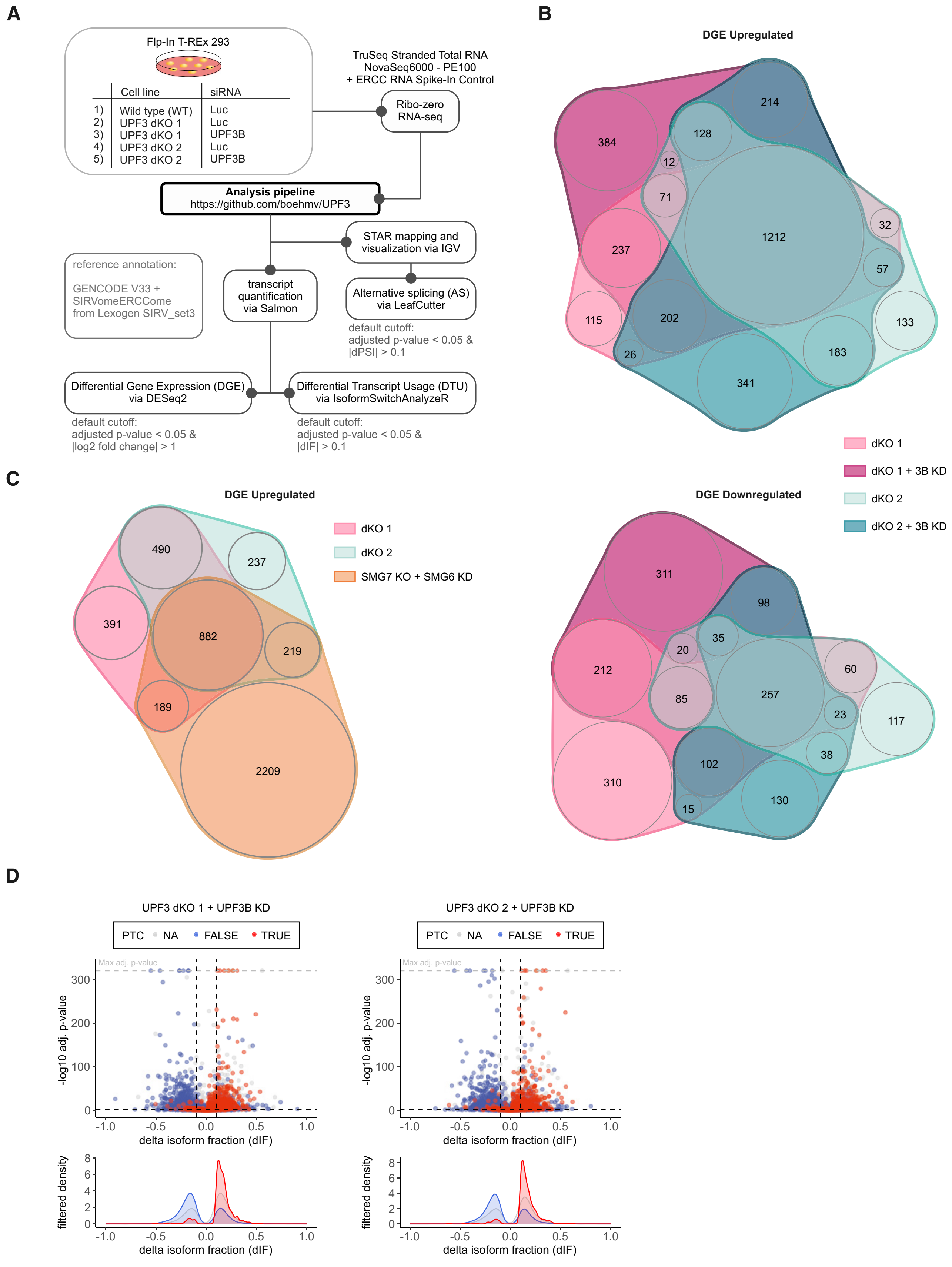
A
Flp-In T-REx 293
Cell line
siRNA
1) Wild type (WT) Luc
2) UPF3 dKO 1 Luc
3) UPF3 dKO 1 UPF3B
4) UPF3 dKO 2 Luc
5) UPF3 dKO 2 UPF3B
TruSeq Stranded Total RNA
NovaSeq6000 - PE100
+ ERCC RNA Spike-In Control
Ribo-zero RNA-seq
Analysis pipeline
https://github.com/boehmv/UPF3
STAR mapping and visualization via IGV
reference annotation:
GENCODE V33 + SIRVomeERCCome from Lexogen SIRV_set3
transcript quantification via Salmon
Alternative splicing (AS) via LeafCutter
default cutoff:
adjusted p-value < 0.05 &
|dPSI| > 0.1
Differential Gene Expression (DGE) via DESeq2
Differential Transcript Usage (DTU) via IsoformSwitchAnalyzeR
default cutoff:
adjusted p-value < 0.05 &
|log2 fold change| > 1
default cutoff:
adjusted p-value < 0.05 &
|dIF| > 0.1
B
DGE Upregulated
384
214
128
12
71
1212
32
237
57
115
202
133
26
183
341
DGE Downregulated
311
98
35
20
212
60
85
257
23
117
38
102
310
130
15
dKO 1
dKO 1 + 3B KD
dKO 2
dKO 2 + 3B KD
C
DGE Upregulated
490
237
391
882
219
189
2209
dKO 1
dKO 2
SMG7 KO + SMG6 KD
D
UPF3 dKO 1 + UPF3B KD
UPF3 dKO 2 + UPF3B KD
PTC
NA
FALSE
TRUE
Max adj. p-value
-log10 adj. p-value
delta isoform fraction (dIF)
filtered density

Figure EV4.

Figure EV5. Single mutations do not destroy UPF3B NMD functionality in HEK293 and HeLa cells.

A Western blot analysis of WT and UPF3B KO clone 90 cells with Luciferase and UPF3A KD, respectively. Monitored expression of globin WT and PTC39 reporters and the indicated UPF3B rescue constructs. Rescue construct protein levels were detected with anti-FLAG and anti-UPF3B (AK-141) antibodies. Tubulin serves as control (*n* = 1). The asterisk indicates an unspecific band.

B End-point RT–PCR detection of RSRC2 transcript isoforms in the samples from (A). The detected RSRC2 isoforms are indicated on the bottom, the NMD-inducing included exon is marked in red (e = exon). Relative mRNA levels of RSRC2 isoforms were quantified from bands of agarose gels (*n* = 3).

C Northern blot analysis of globin reporter and xrFrag. Ethidium bromide stained 28S and 18S rRNAs are shown as controls. Lanes 5 and 6 were mirrored because of a pipetting error. Quantification results are shown as data points and mean (*n* = 3).

D Western blot analysis of HeLa WT and UPF3B KO cells with Luciferase and UPF3A KDs respectively. Monitored expression of the FLAG-tagged UPF3B rescue construct shown in (Fig 6D). Rescue construct protein levels were detected with anti-FLAG and anti-UPF3B (AK-141) antibodies. Tubulin serves as control (*n* = 1). The asterisk indicates an unspecific band.

E Quantitative RT–PCR of the samples from (D). For RSRC2 and SRSF2, the ratio of NMD isoform to canonical isoform was calculated. GAS5 expression was normalized to EMC7 reference. Data points and means are plotted as log2 fold change (*n* = 3).

F Overview of TurboID-mediated proximity labeling of UPF3B WT and UPF3B mutant construct binding partners in UPF3B KO clone 90 with additional UPF3A KD. Transient UPF3B interactors are marked with biotin via TurboID catalysis. Biotinylated proteins are subsequently enriched with streptavidin beads.

G–J Volcano plots of mass spectrometry-based analysis of streptavidin-enriched biotinylated proteins in the respective comparison of conditions. (G) FLAG-TurboID-UPF3B against FLAG-TurboID control, (H) FLAG-TurboID-UPF3B ΔUPF2 interaction (K52D/RR56EE) against FLAG-TurboID-UPF3B, (I) FLAG-TurboID-UPF3B ΔEJC interaction (Δ421–434) against FLAG-TurboID-UPF3B, (J) FLAG-TurboID-UPF3B Δmiddle domain (Δ147–256) against FLAG-TurboID-UPF3B, all in UPF3B KO + UPF3A KD cells. Points labeled in purple indicate NMD factors; points labeled in turquoise indicate EJC components (*n* = 3 biologically independent samples).

Source data are available online for this figure.

© 2022 The Authors

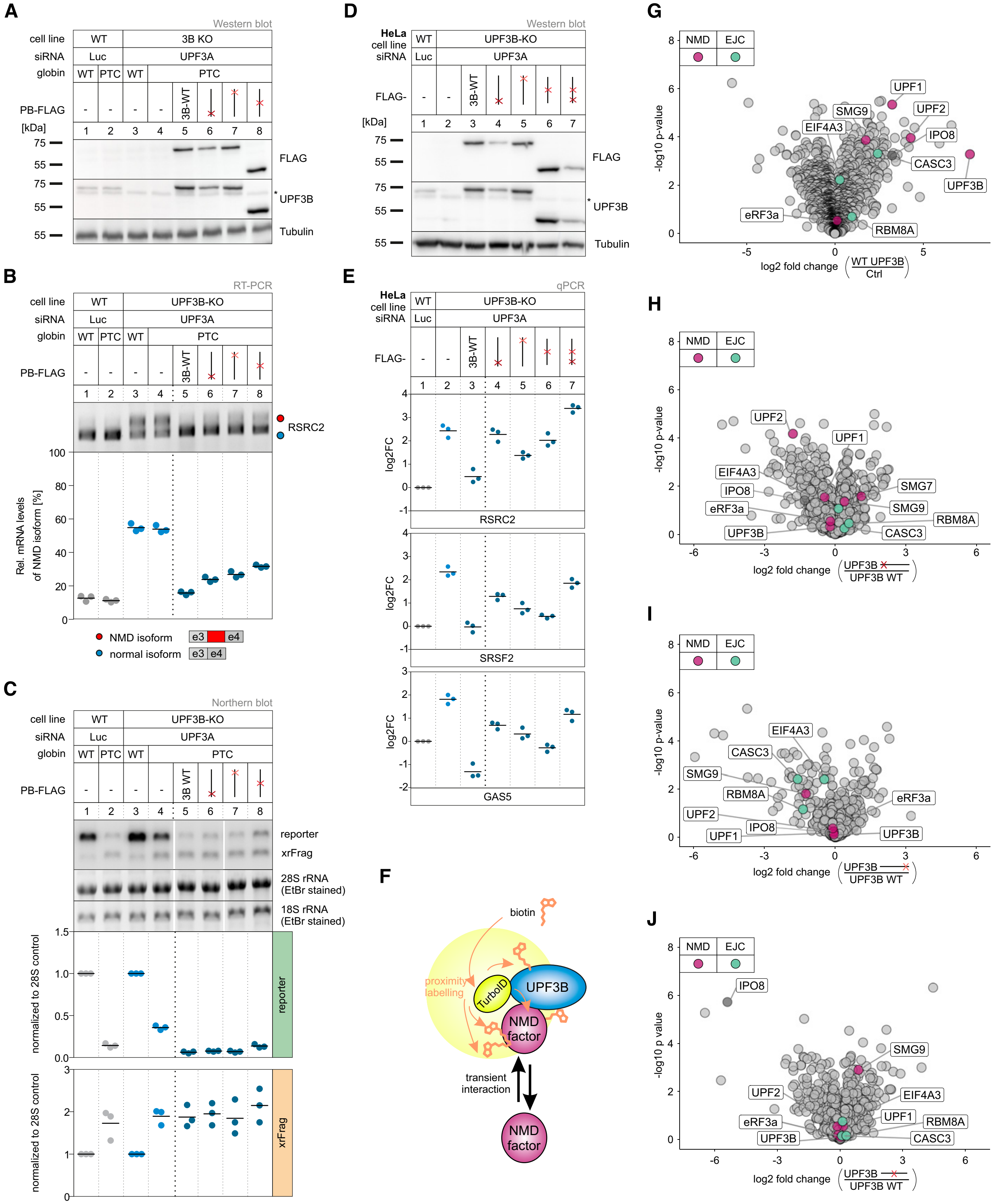
A
Western blot
cell line WT 3B KO
siRNA Luc UPF3A
globin WT PTC WT PTC
PB-FLAG 3B-WT
[kDa]
FLAG
UPF3B
Tubulin
B
RT-PCR
RSRC2
Rel. mRNA levels of NMD isoform [%]
NMD isoform e3 e4
normal isoform e3 e4
C
Northern blot
reporter
xrFrag
28S rRNA (EtBr stained)
18S rRNA (EtBr stained)
normalized to 28S control
D
Western blot
HeLa cell line WT UPF3B-KO
siRNA Luc UPF3A
FLAG-
E
qPCR
log2FC
RSRC2
SRSF2
GAS5
F
biotin
proximity labelling
TurboID
UPF3B
NMD factor
transient interaction
G
NMD EJC
UPF1 UPF2 SMG9 EIF4A3 IPO8 CASC3 UPF3B eRF3a RBM8A
-log10 p-value
log2 fold change (WT UPF3B / Ctrl)
H
UPF2 UPF1 EIF4A3 IPO8 eRF3a UPF3B SMG7 SMG9 RBM8A CASC3
log2 fold change (UPF3B / UPF3B WT)
I
EIF4A3 CASC3 SMG9 RBM8A UPF2 UPF1 IPO8 eRF3a UPF3B
J
IPO8 SMG9 UPF2 EIF4A3 UPF1 eRF3a RBM8A UPF3B CASC3
-log10 p value

Figure EV5.
