# Supplementary figures and images for: Human UPF3A and UPF3B enable fault‐tolerant activation of nonsense‐mediated mRNA decay
Source: EMBO J. 2022 Apr 22;41(10):e109191. doi: 10.15252/embj.2021109191 (PMC9108619; doi:10.15252/embj.2021109191)

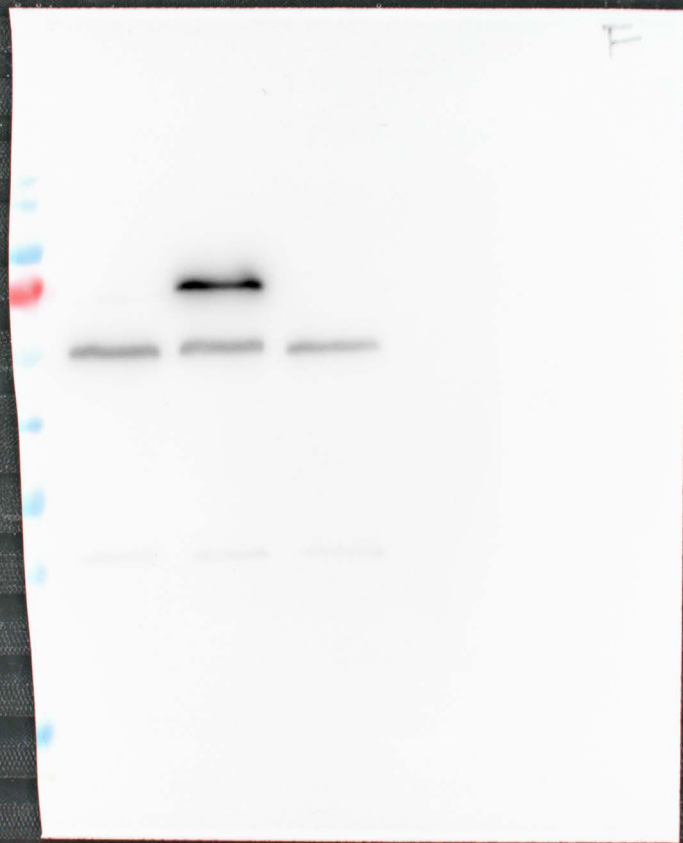

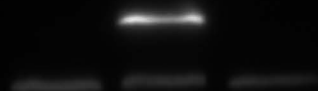

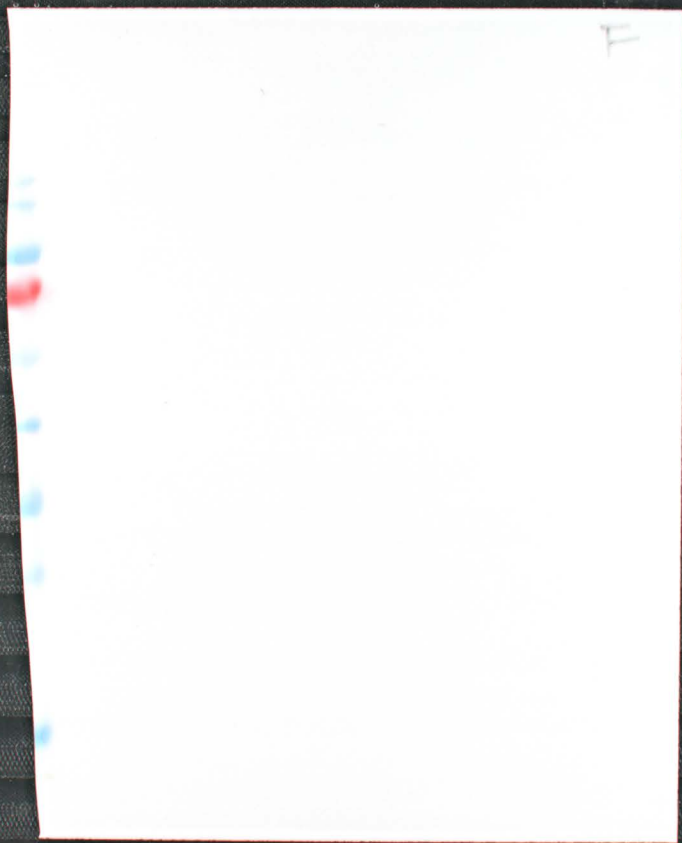

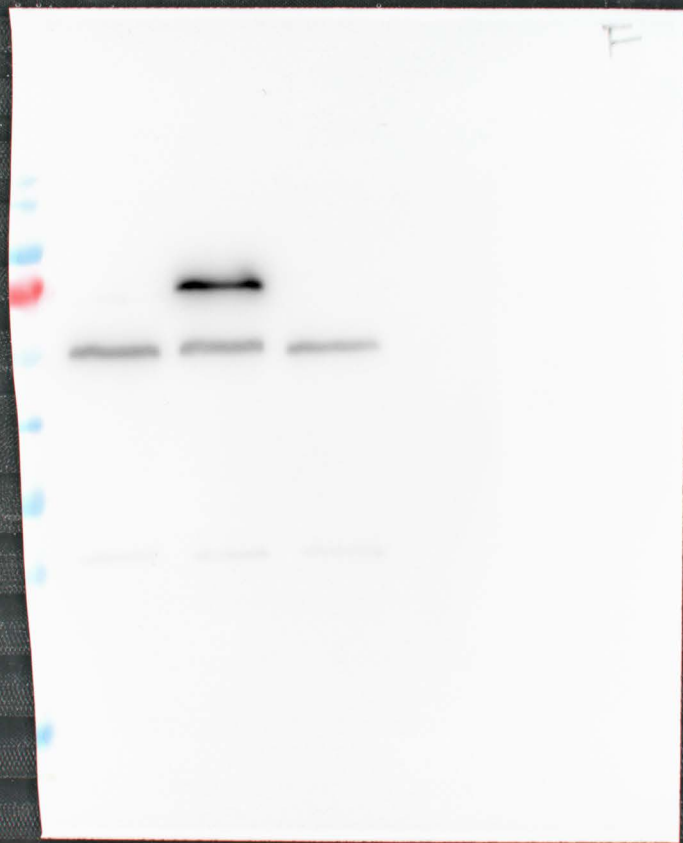

Supplement: Supplementary file 11 — Source Data for Figure 1 [file EMBJ-41-e109191-s017.zip › Fig1/Fig1B_HEK_UPF3A_OE_FLAG.pdf]

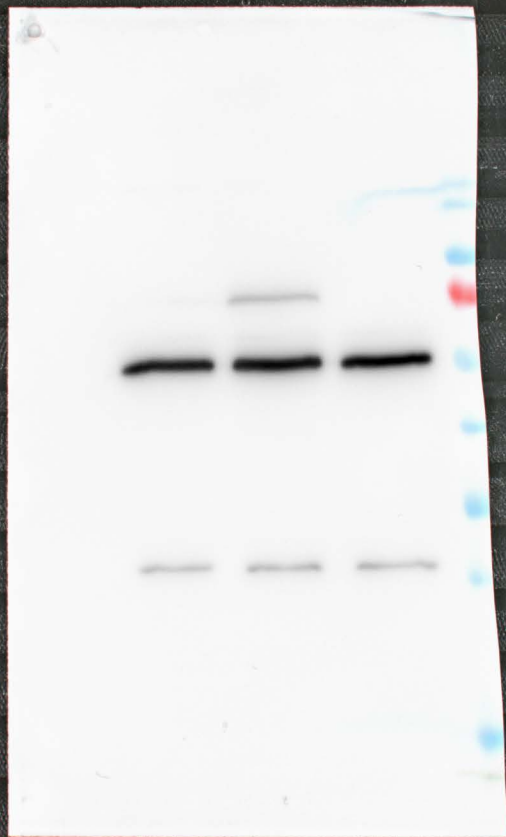

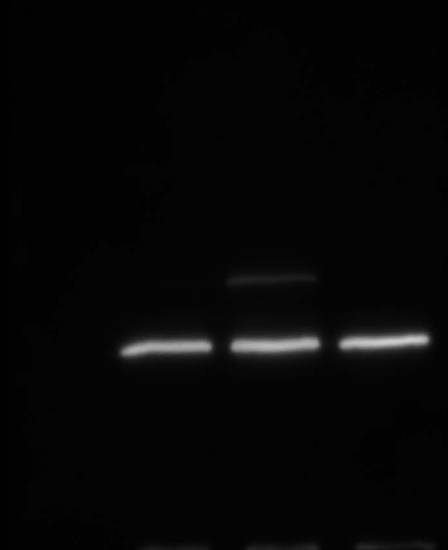

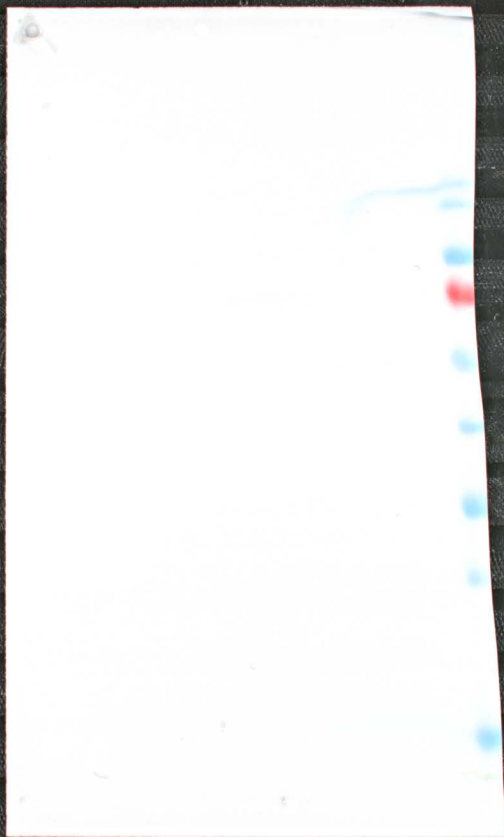

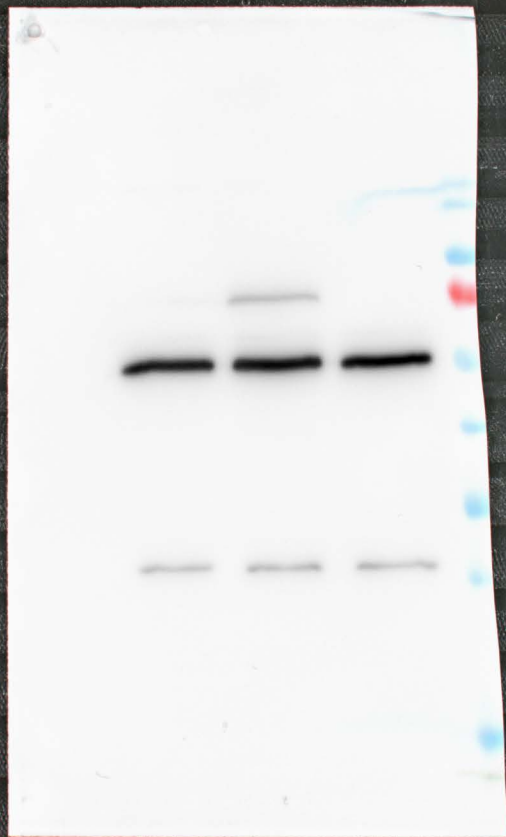

Supplement: Supplementary file 11 — Source Data for Figure 1 [file EMBJ-41-e109191-s017.zip › Fig1/Fig1B_HEK_UPF3A_OE_Tubulin.pdf]

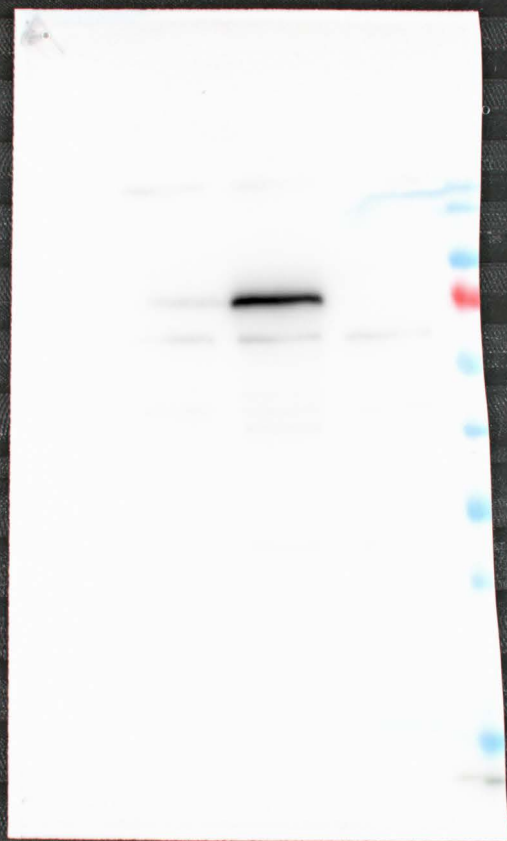

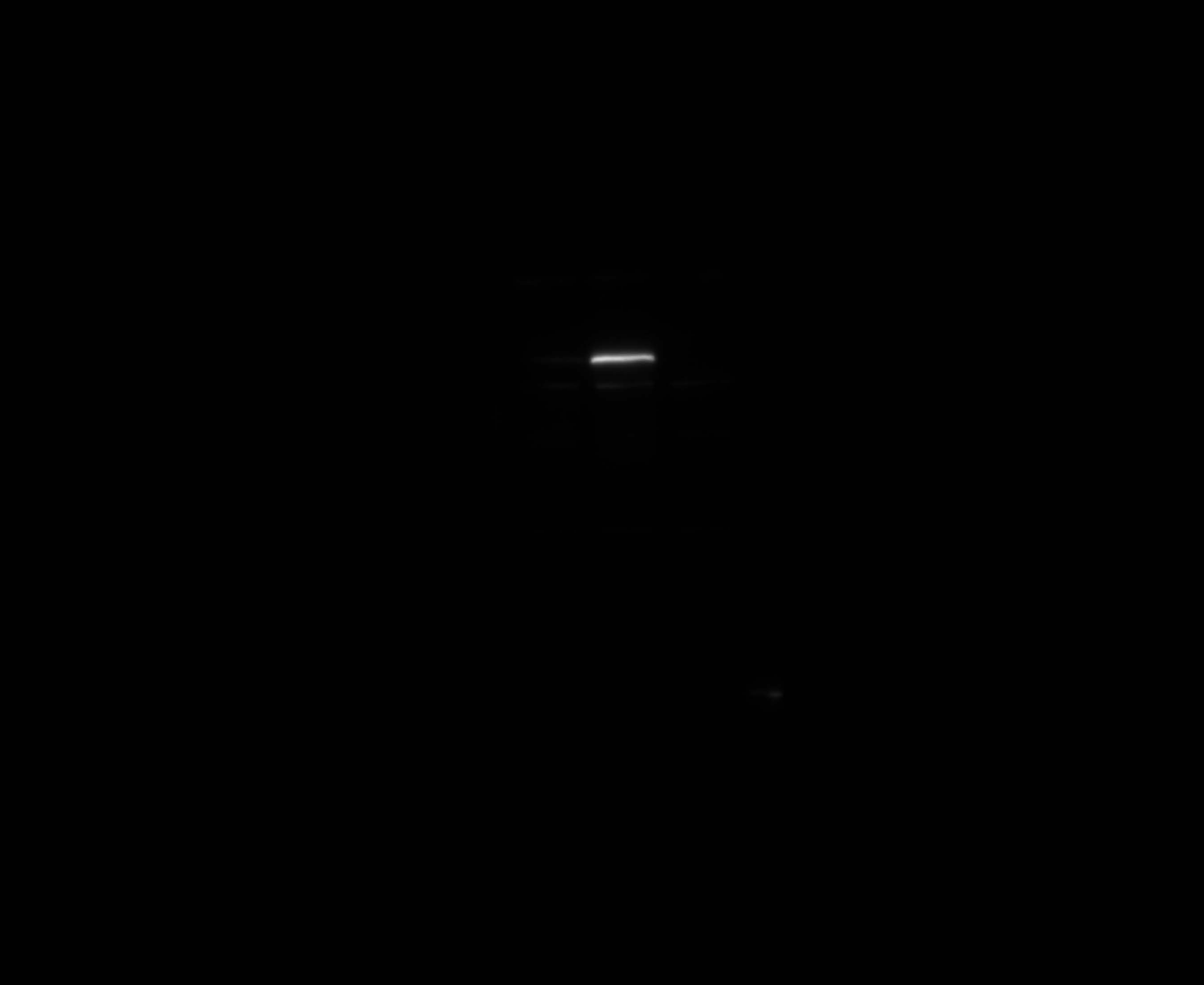

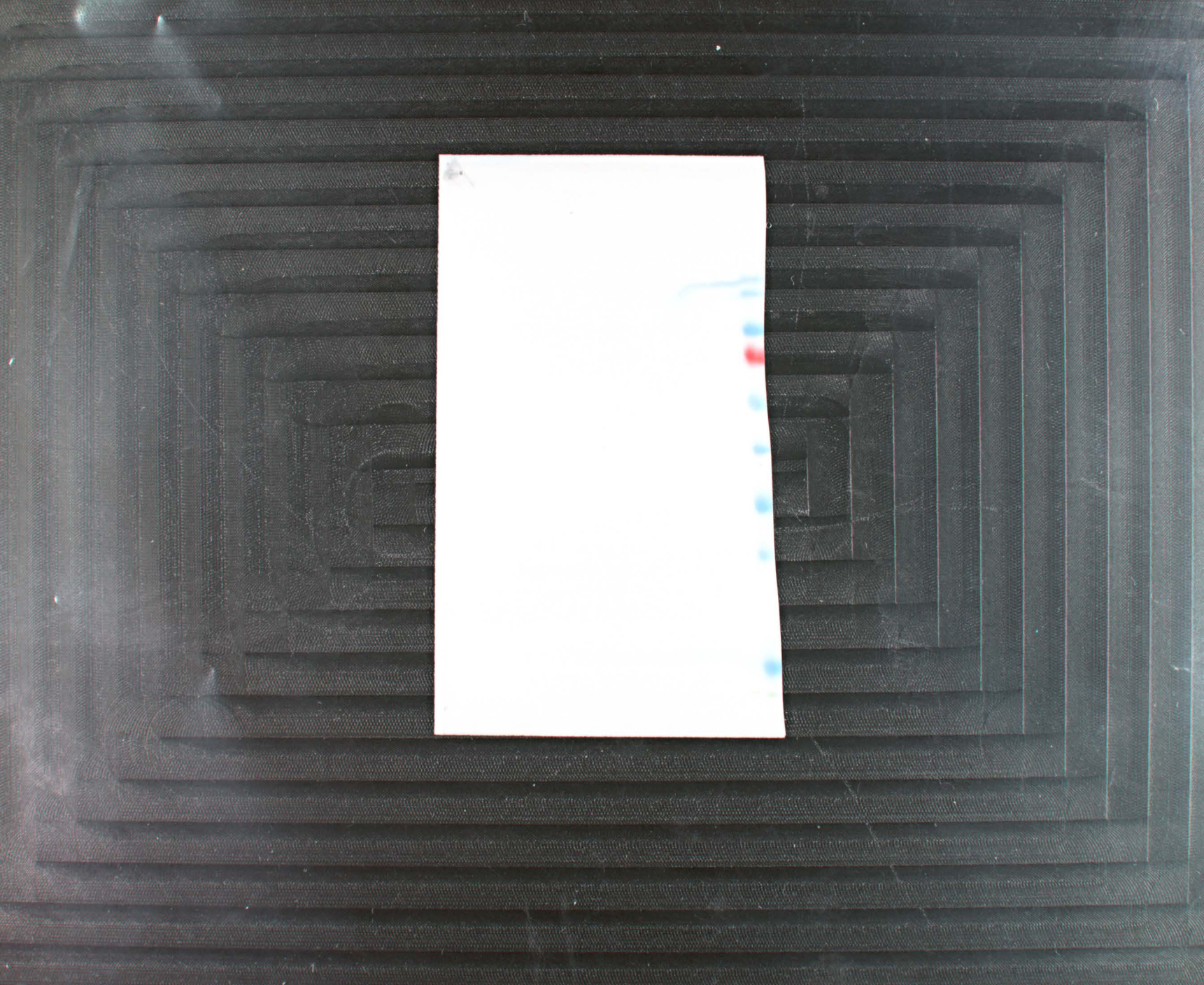

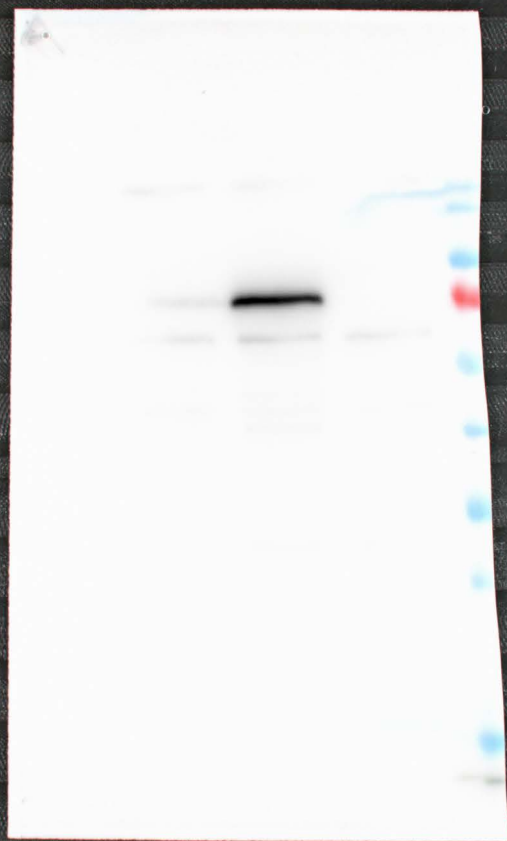

Supplement: Supplementary file 11 — Source Data for Figure 1 [file EMBJ-41-e109191-s017.zip › Fig1/Fig1B_HEK_UPF3A_OE_UPF3A.pdf]

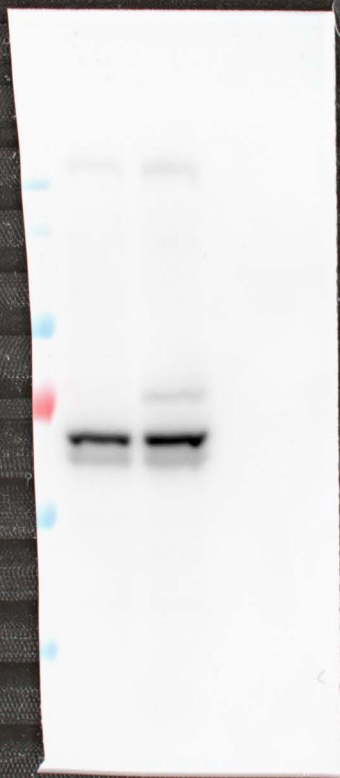



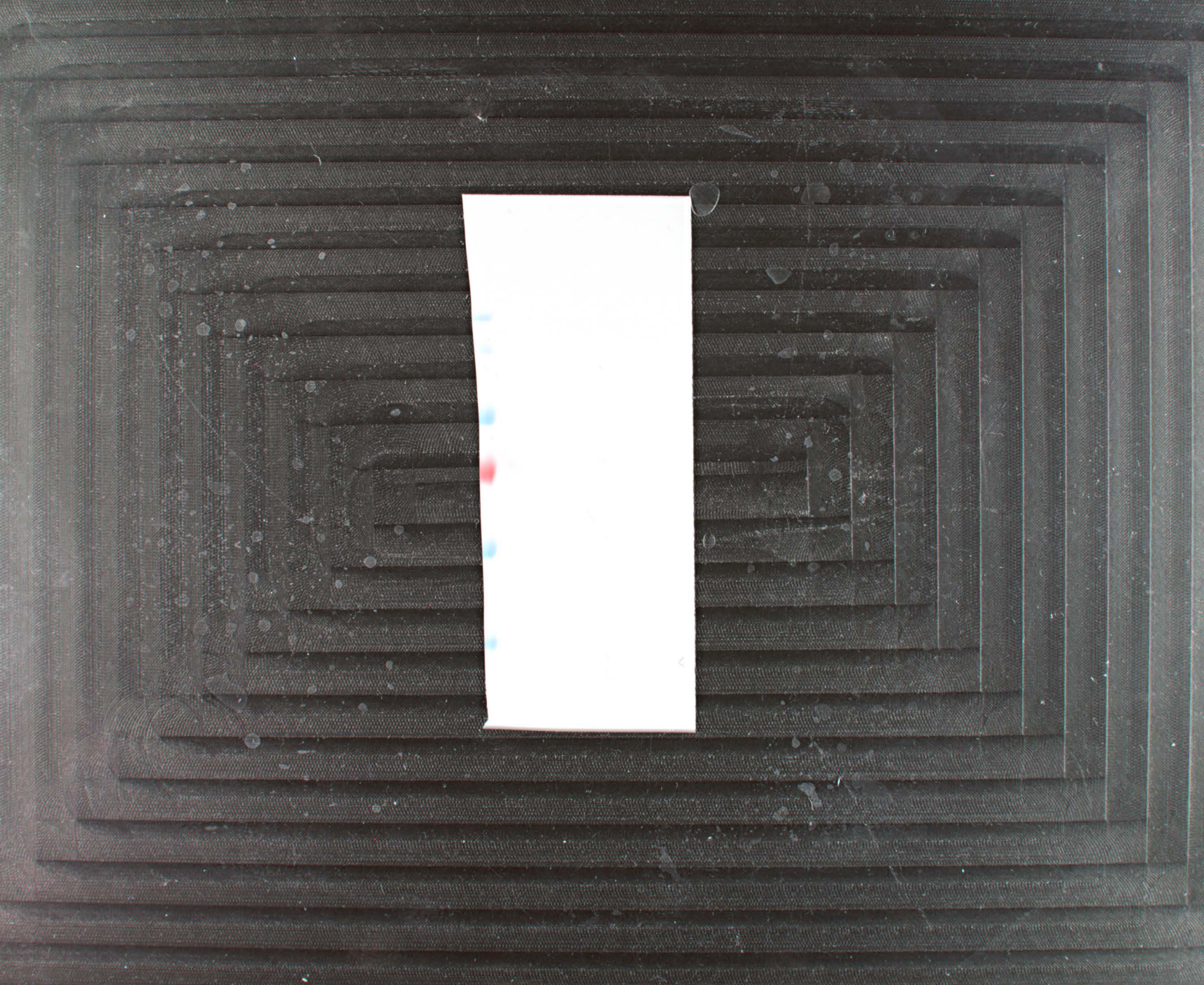

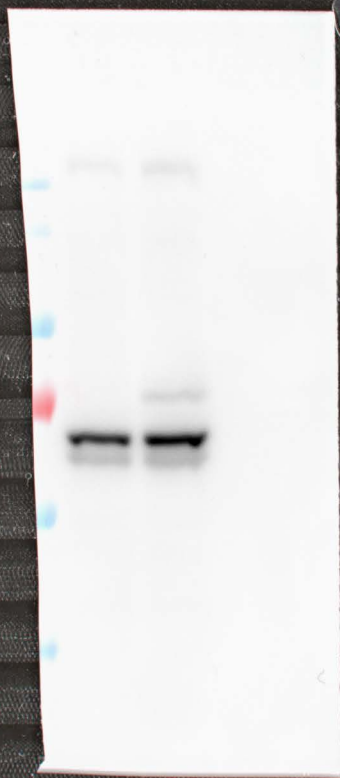

Supplement: Supplementary file 11 — Source Data for Figure 1 [file EMBJ-41-e109191-s017.zip › Fig1/Fig1B_HEK_UPF3A_OE_UPF3B.pdf]

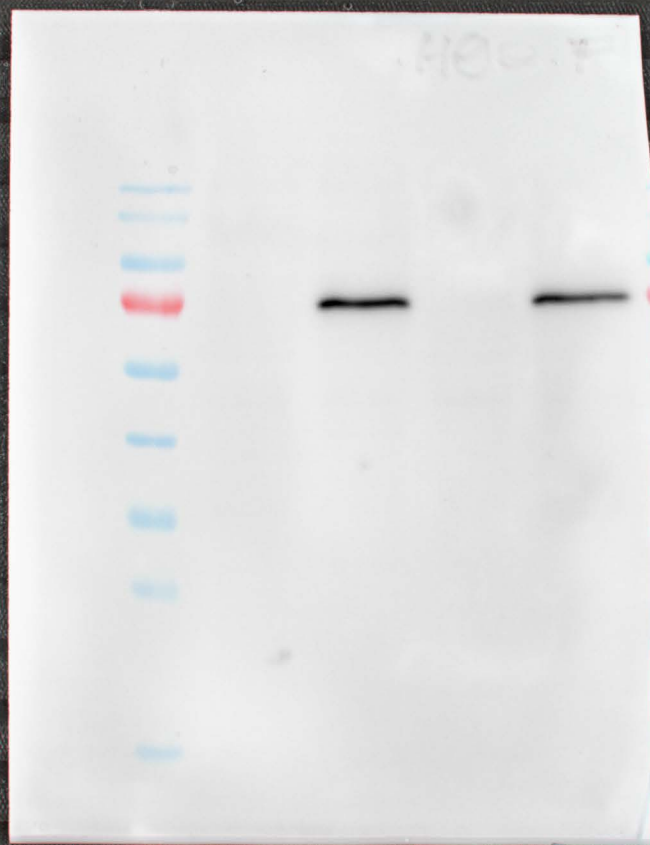

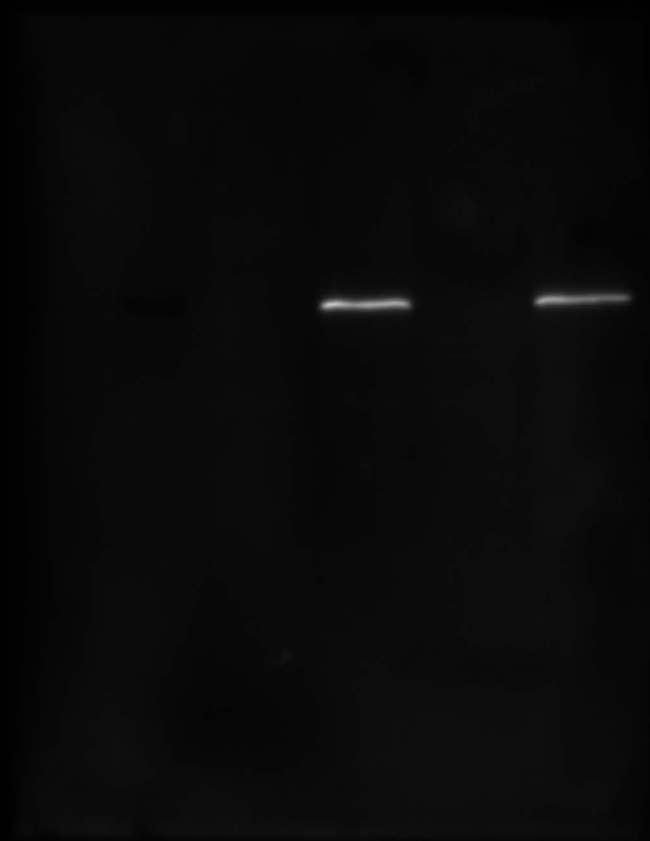

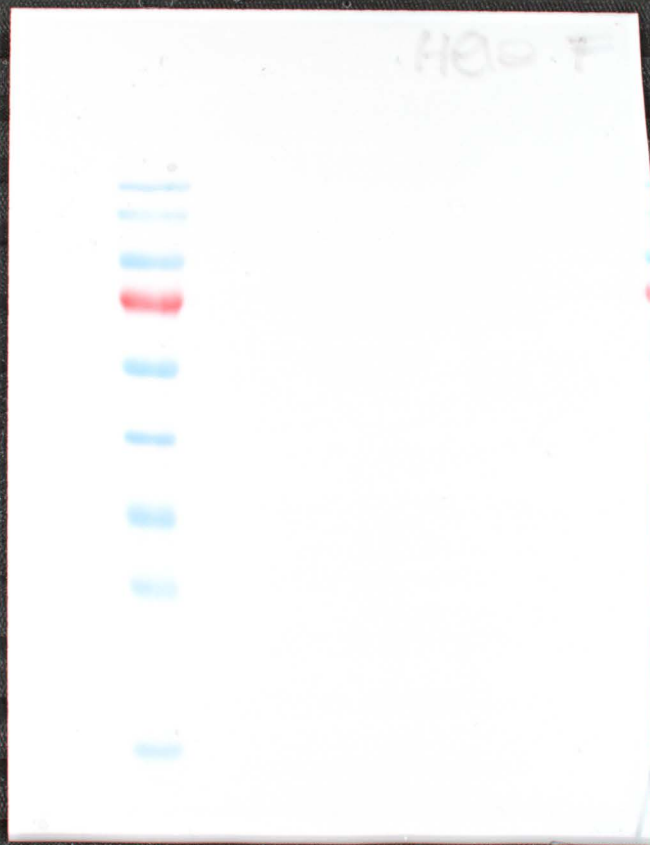

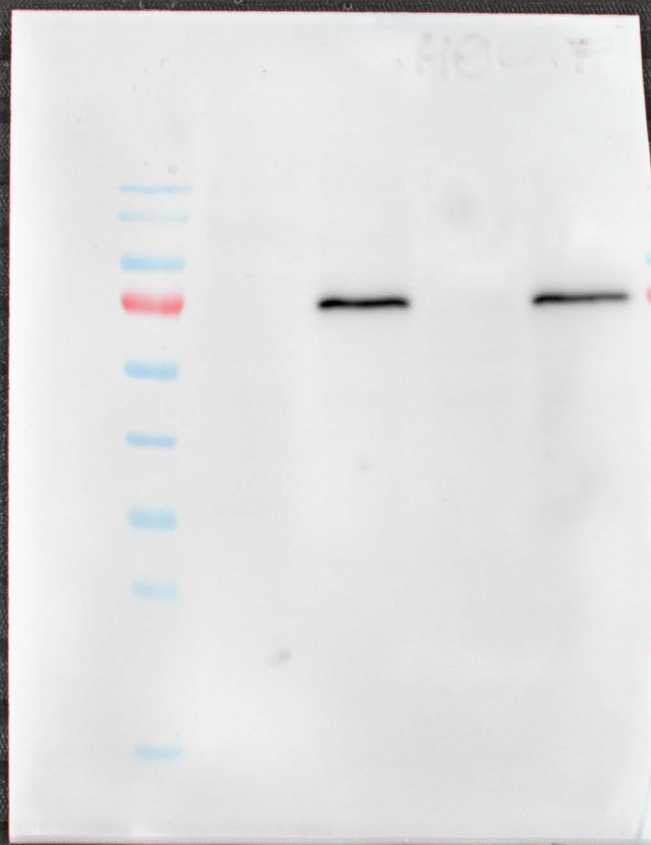

Supplement: Supplementary file 11 — Source Data for Figure 1 [file EMBJ-41-e109191-s017.zip › Fig1/Fig1B_HeLa_UPF3A_OE_FLAG.pdf]

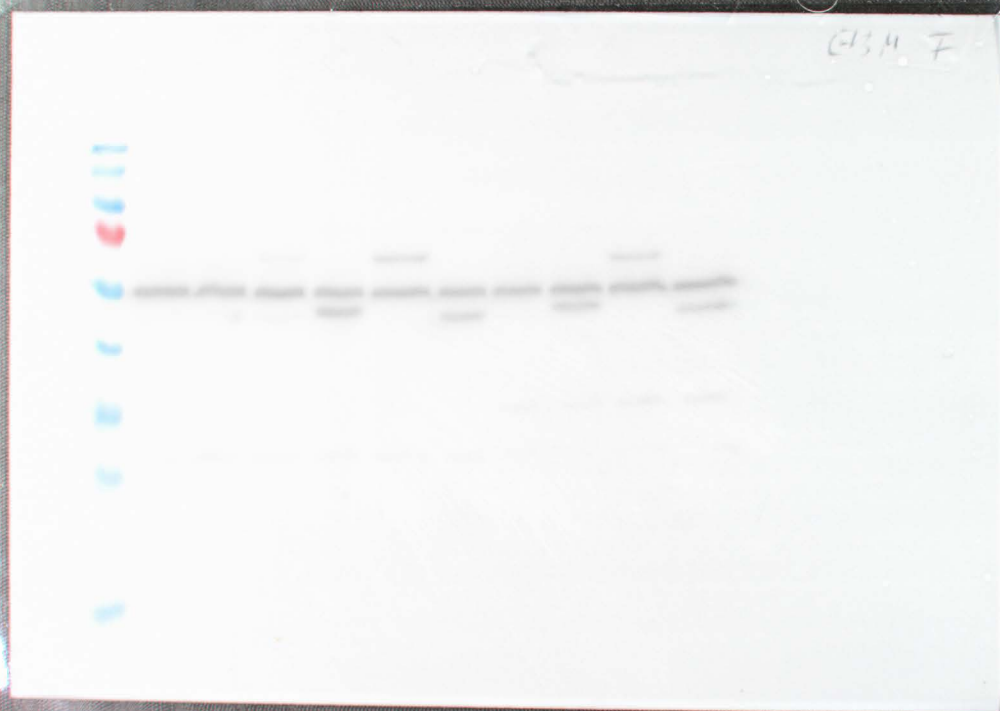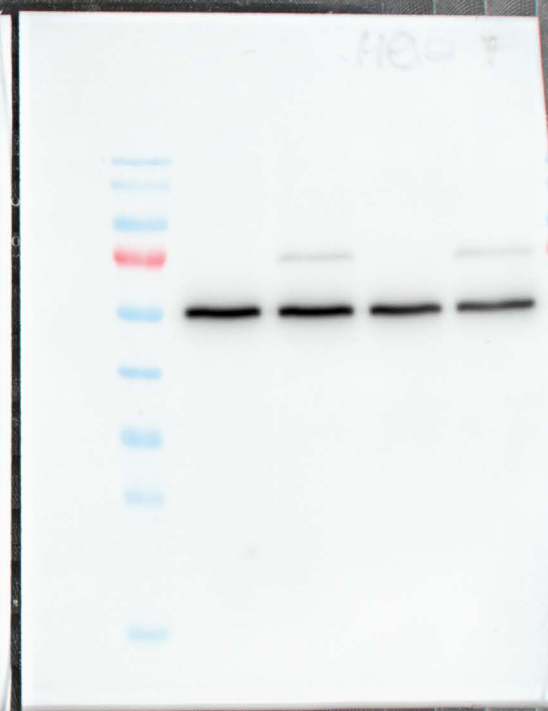

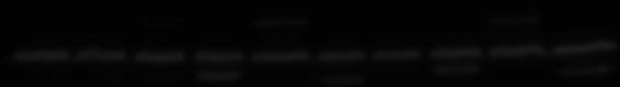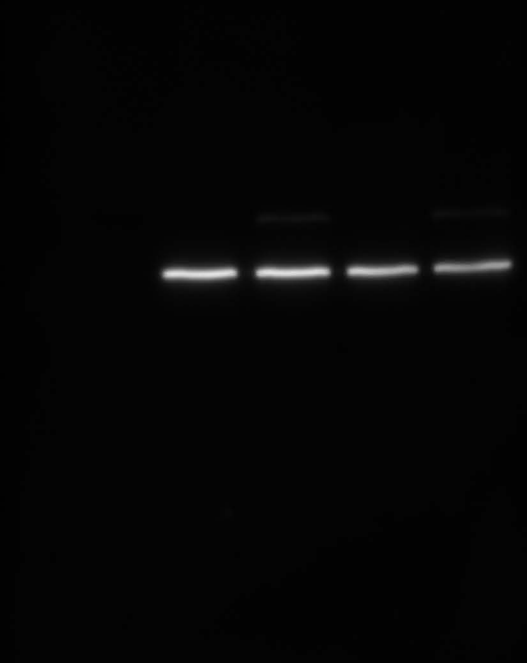

CH 11 F

HO 11 F

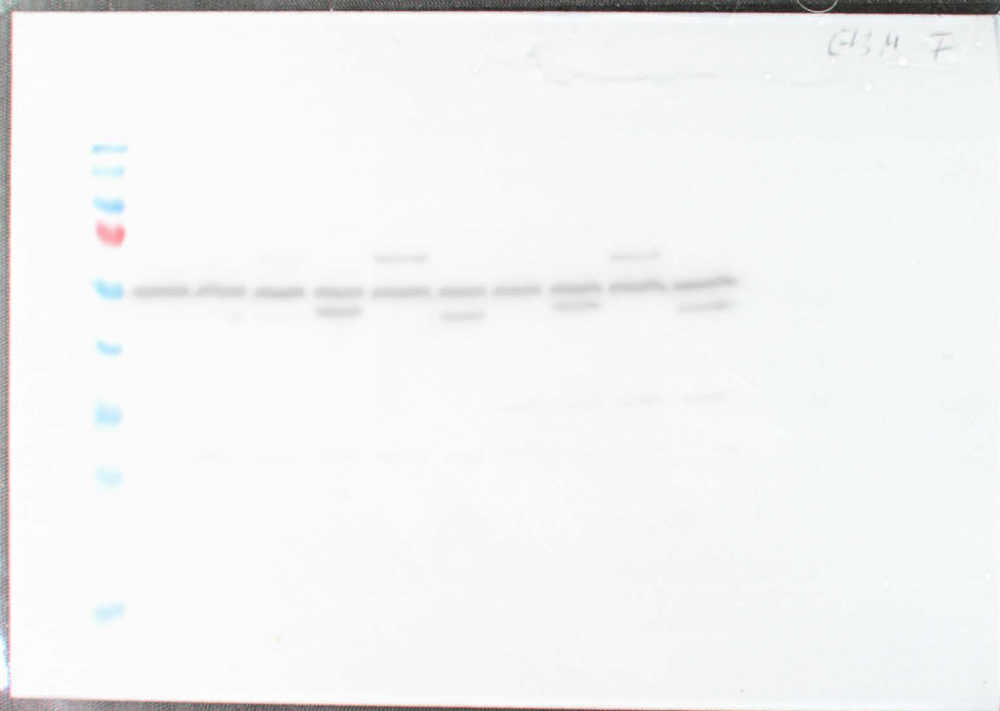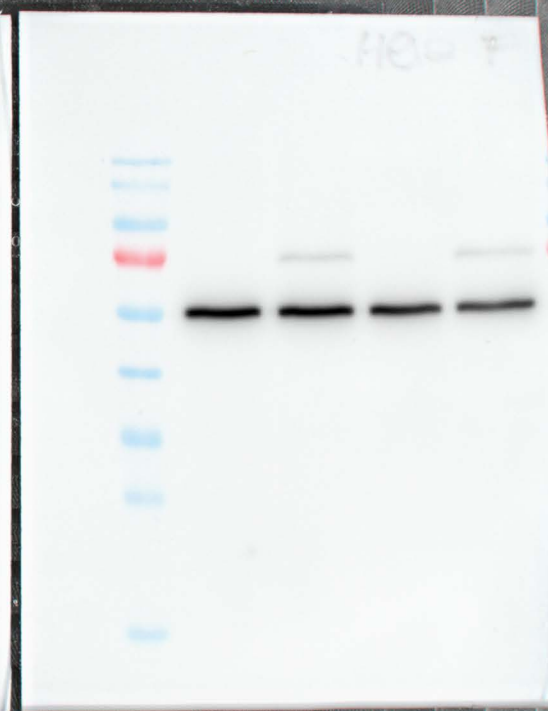

Supplement: Supplementary file 11 — Source Data for Figure 1 [file EMBJ-41-e109191-s017.zip › Fig1/Fig1B_HeLa_UPF3A_OE_Tubulin.pdf]

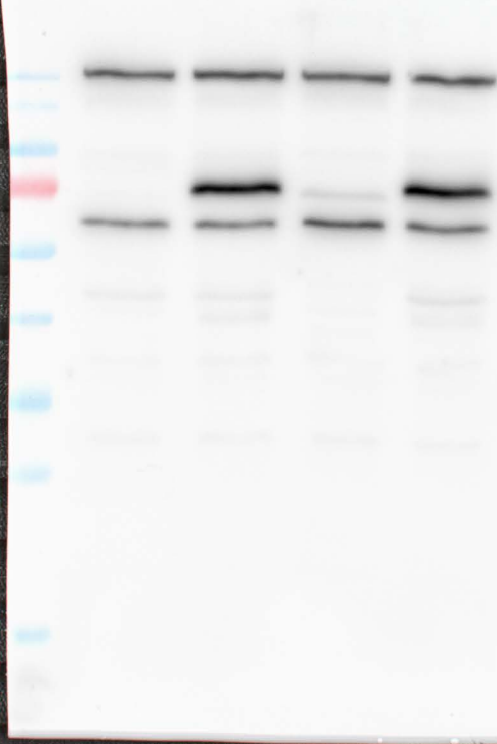

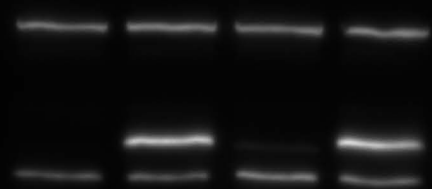

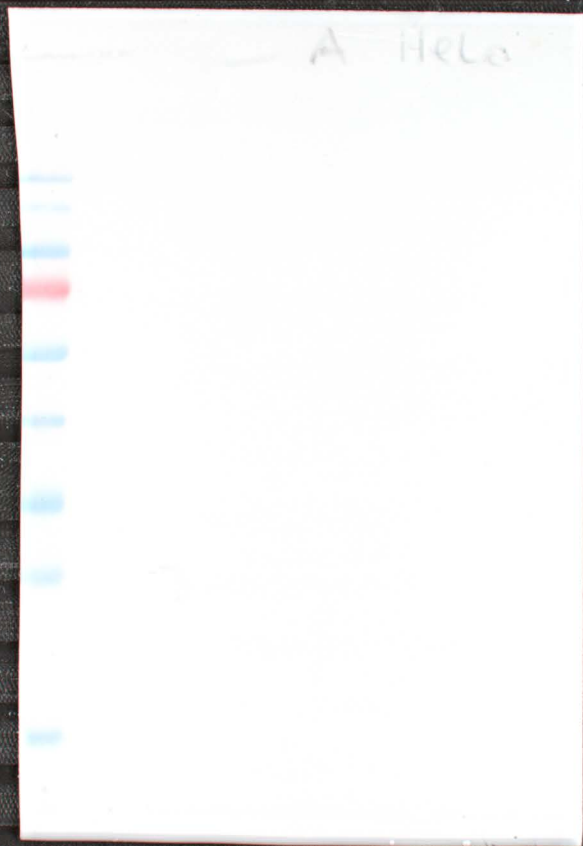

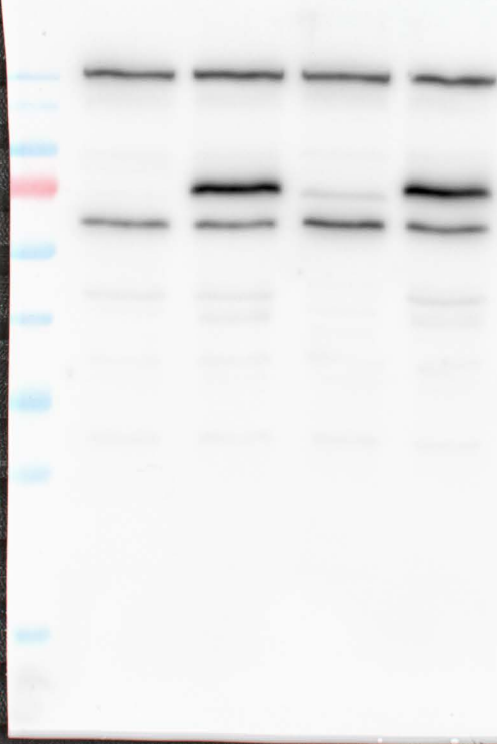

Supplement: Supplementary file 11 — Source Data for Figure 1 [file EMBJ-41-e109191-s017.zip › Fig1/Fig1B_HeLa_UPF3A_OE_UPF3A.pdf]

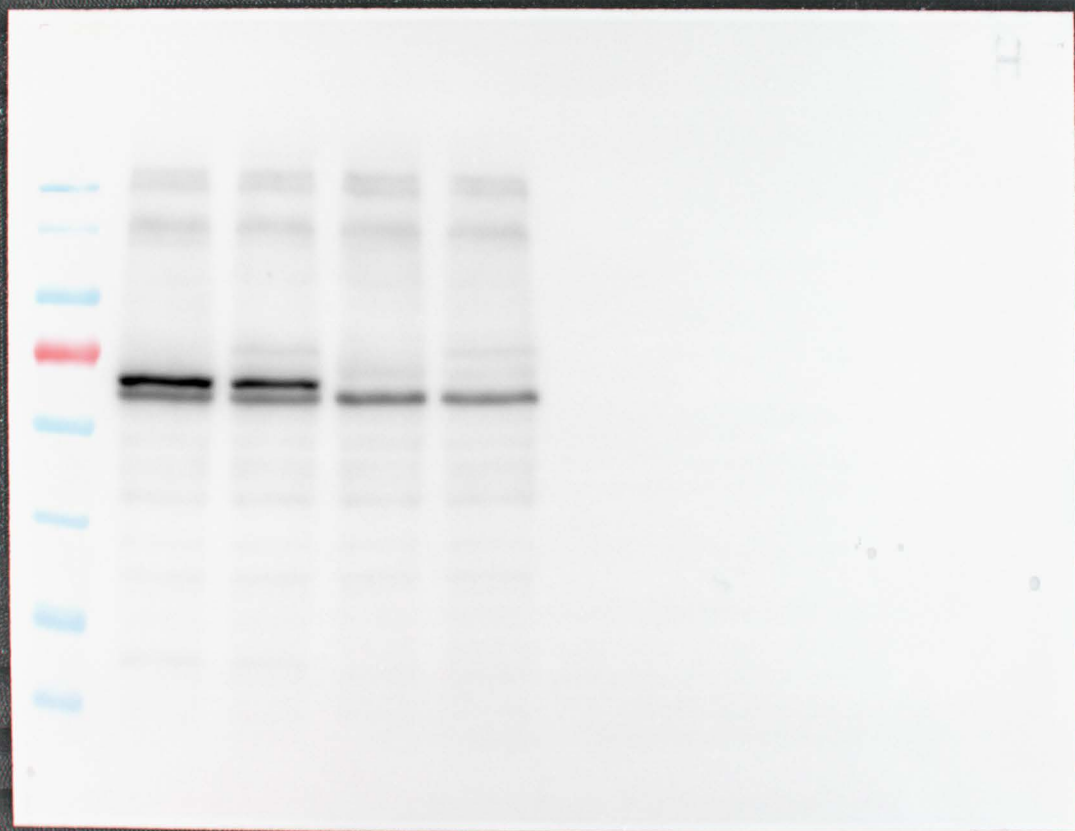



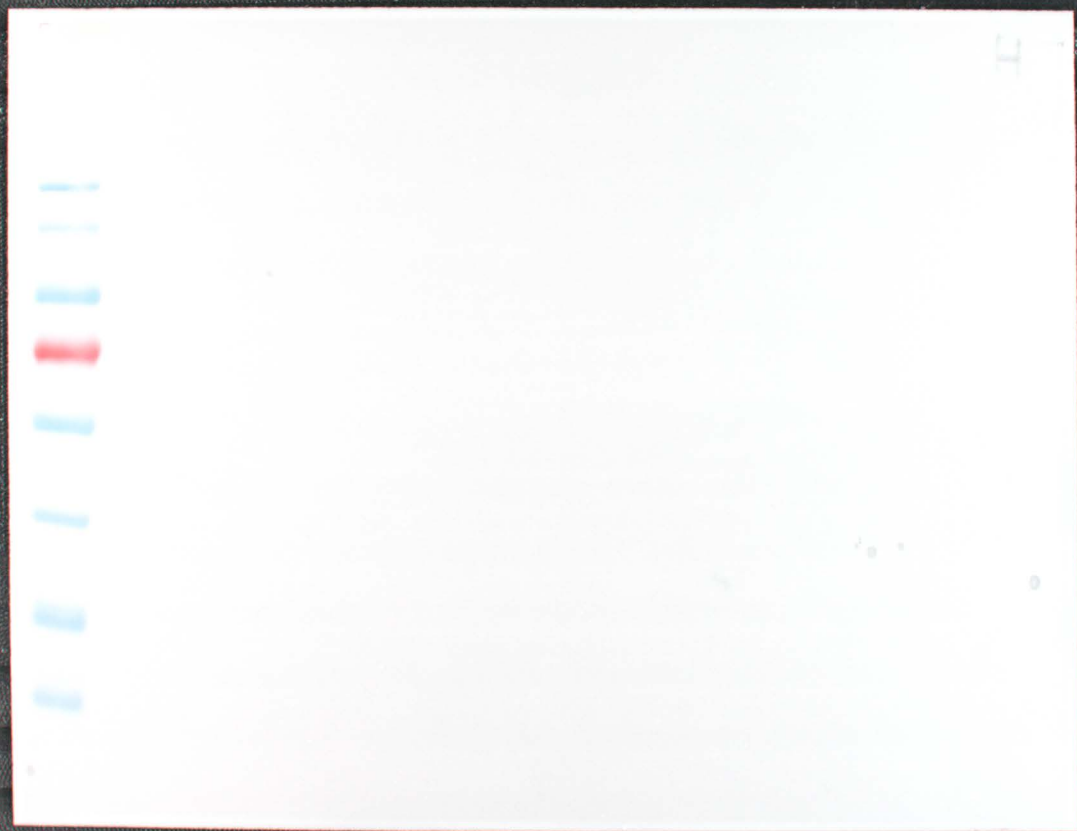

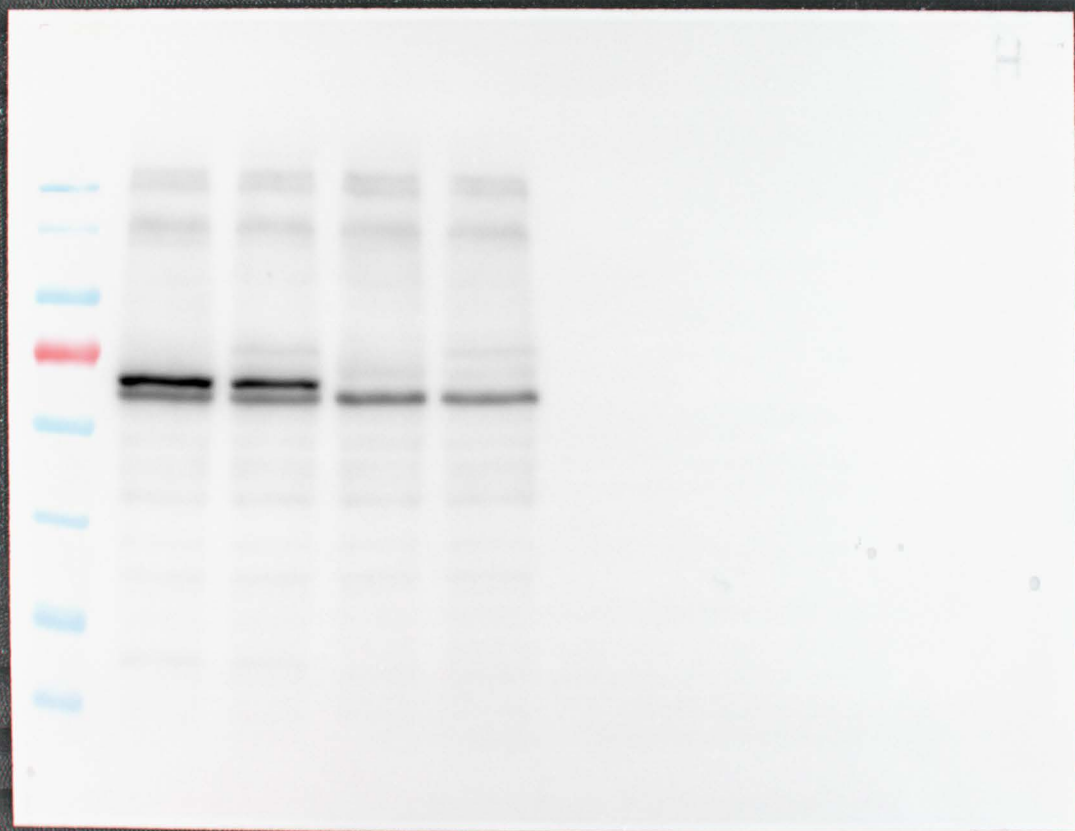

Supplement: Supplementary file 11 — Source Data for Figure 1 [file EMBJ-41-e109191-s017.zip › Fig1/Fig1B_HeLa_UPF3A_OE_UPF3B.pdf]

3

118  
94  
66  
45  
33  
22  
15  
10  
6  
4

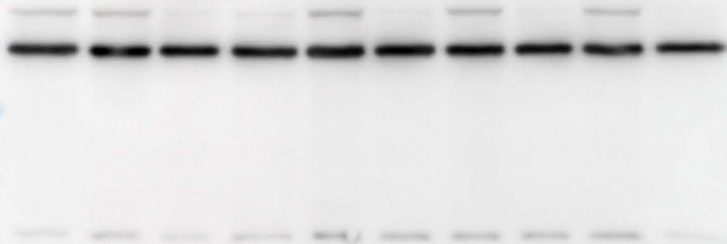

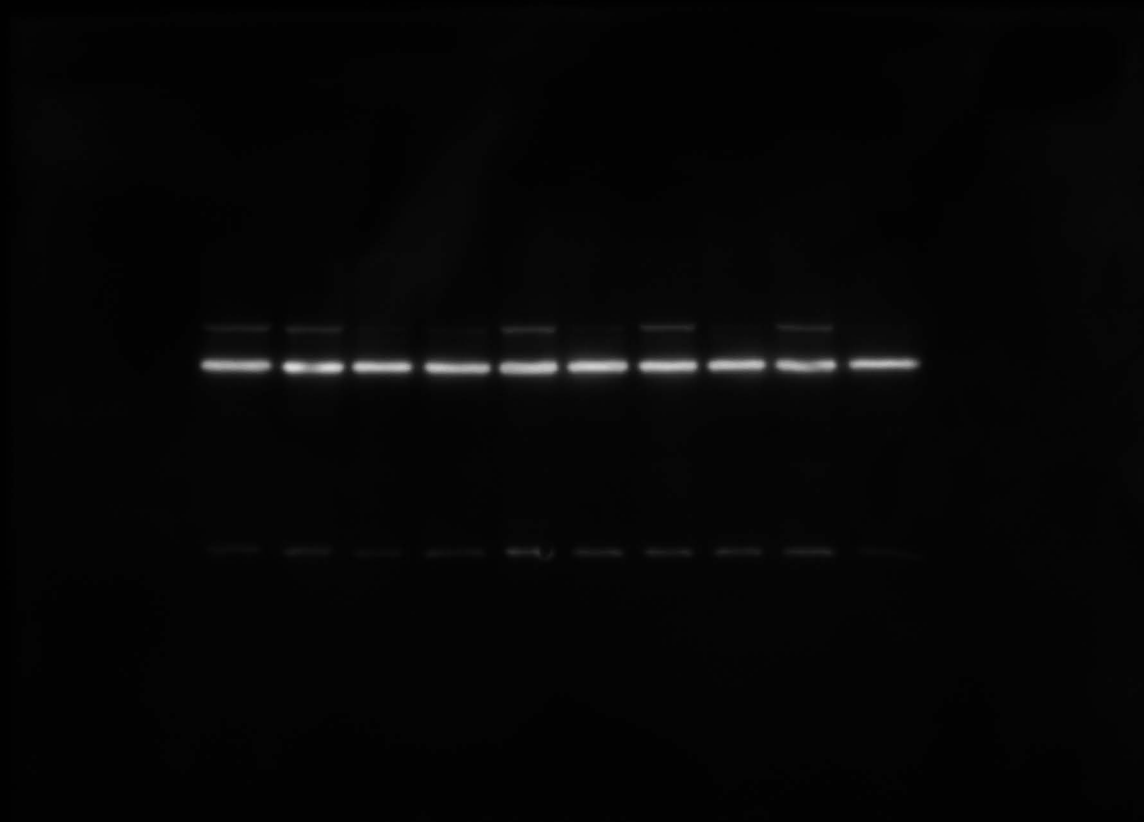

B

113311

3

118  
94  
66  
45  
33  
22  
15  
10  
6  
4

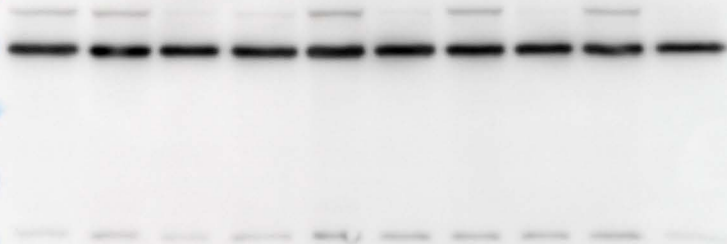

Supplement: Supplementary file 12 — Source Data for Figure 2 [file EMBJ-41-e109191-s014.zip › Fig2/Fig2A_3AKO_Tubulin.pdf]

A

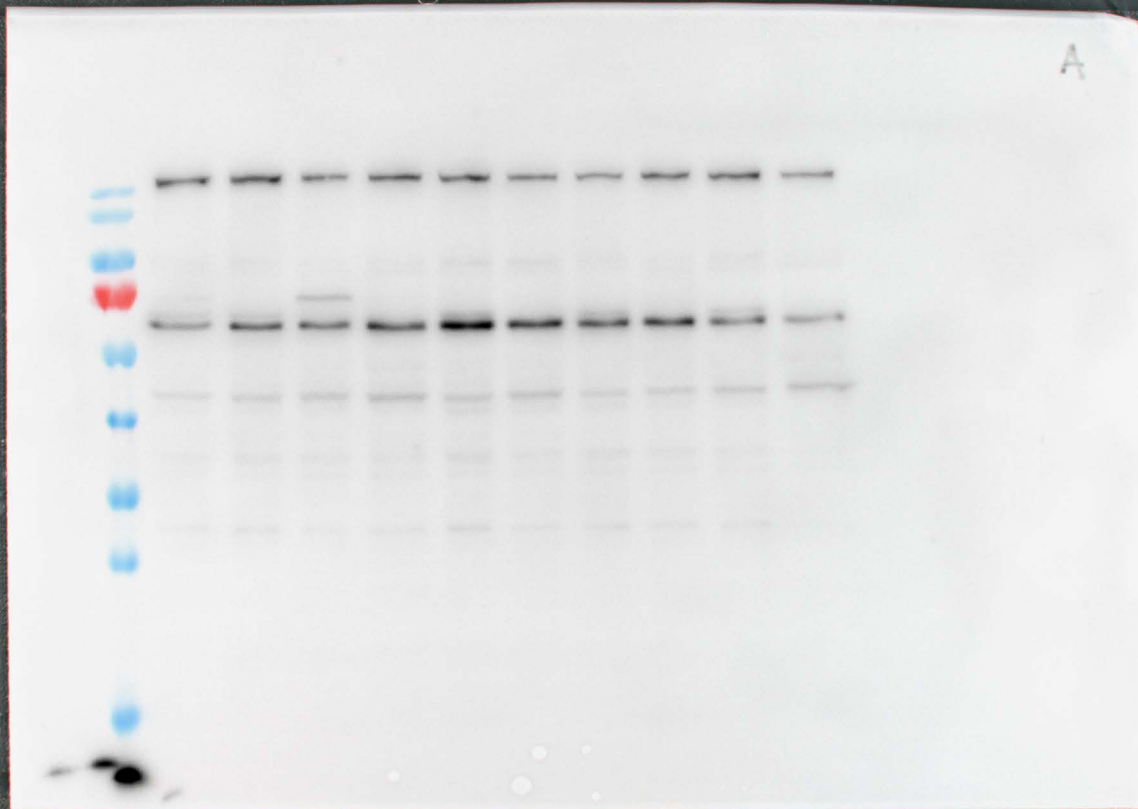



A

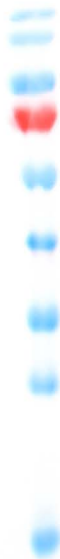

A

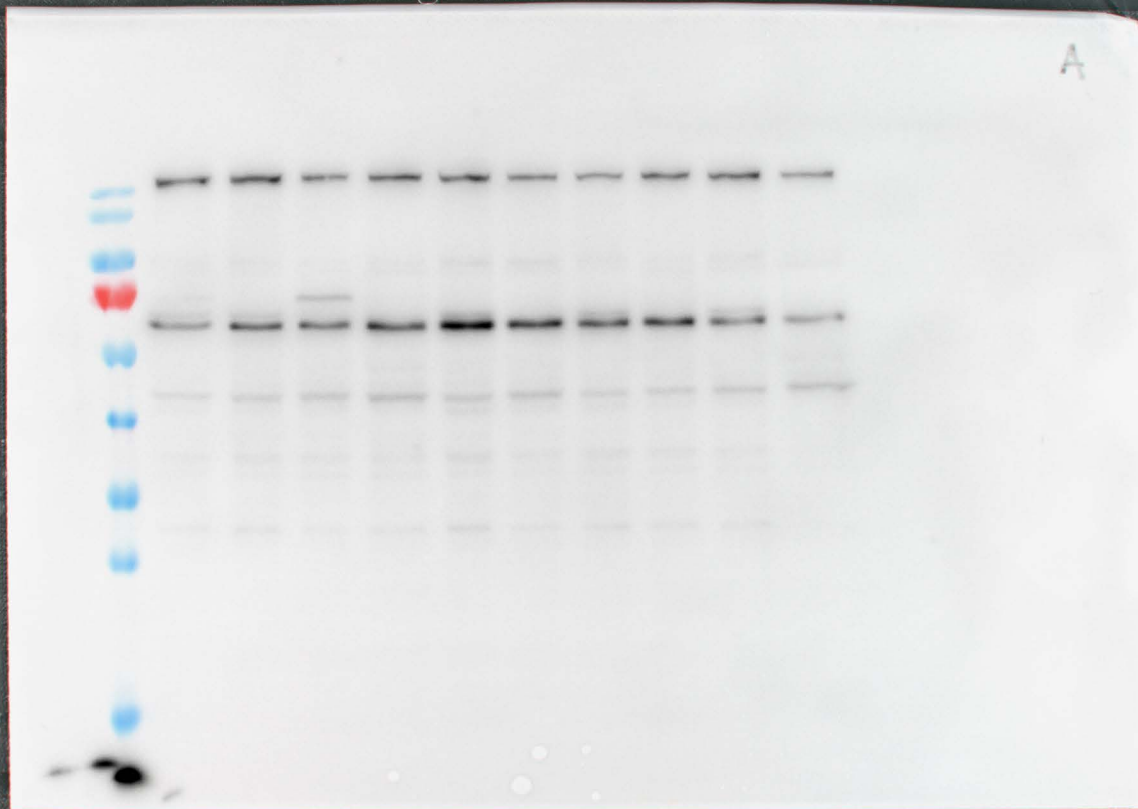

Supplement: Supplementary file 12 — Source Data for Figure 2 [file EMBJ-41-e109191-s014.zip › Fig2/Fig2A_3AKO_UPF3A.pdf]

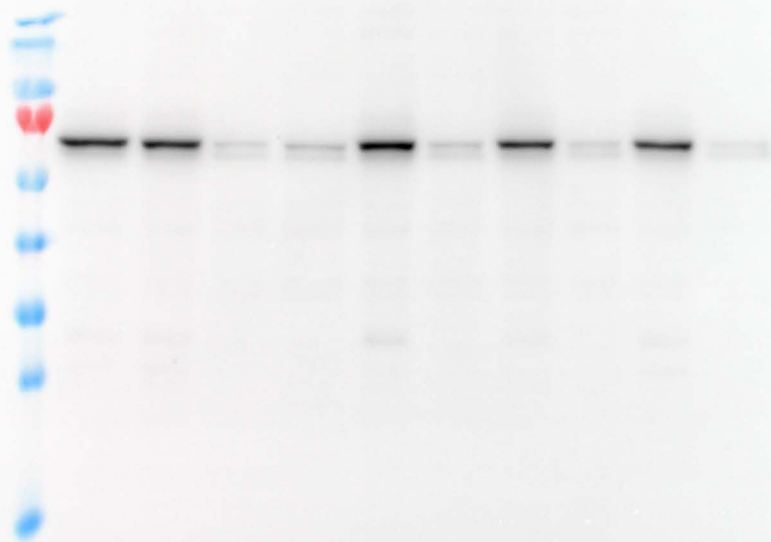



11/11/11

00

3

100 50 25 12.5 6.25 3.125 1.5625 0.78125 0.390625 0.1953125 0.09765625 0.048828125 0.0244140625 0.01220703125 0.006103515625 0.0030517578125 0.00152587890625 0.000762939453125 0.0003814697265625 0.00019073486328125 0.000095367431640625 0.0000476837158203125 0.00002384185791015625 0.000011920928955078125 0.0000059604644775390625 0.00000298023223876953125 0.000001490116119384765625 0.0000007450580596923828125 0.00000037252902984619140625 0.000000186264514923095703125 0.0000000931322574615478515625 0.00000004656612873077392578125 0.000000023283064365386962890625 0.0000000116415321826934814453125 0.00000000582076609134674072265625 0.000000002910383045673370361328125 0.0000000014551915228366851806640625 0.00000000072759576141834259033203125 0.000000000363797880709171295166015625 0.0000000001818989403545856475830078125 0.00000000009094947017729282379150390625 0.000000000045474735088646411895751953125 0.0000000000227373675443232059478759765625 0.00000000001136868377216160297393798828125 0.000000000005684341886080801486968994140625 0.0000000000028421709430404007434844970703125 0.00000000000142108547152020037174224853515625 0.000000000000710542735760100185871124267578125 0.0000000000003552713678800500929355621337890625 0.00000000000017763568394002504646778106689453125 0.000000000000088817841970012523233890533447265625 0.0000000000000444089209850062616169452667236328125 0.00000000000002220446049250313080847263336171640625 0.000000000000011102230246251565404236316680858203125 0.0000000000000055511151231257827021181583340291015625 0.00000000000000277555756156289135105907916701455078125 0.000000000000001387778780781445675529539583507275390625 0.0000000000000006938893903907228377647697917536376953125 0.00000000000000034694469519536141888238489587681884765625 0.000000000000000173472347597680709441192447938409423828125 0.0000000000000000867361737988403547205962239692047119140625 0.00000000000000004336808689942017736029811198460235595703125 0.000000000000000021684043449710088680149055992301177978515625 0.0000000000000000108420217248550443400745279961505889892578125 0.00000000000000000542101086242752217003726399807529449462890625 0.00000000000000000271050543121376108501863199903764724731453125 0.000000000000000001355252715606880542509315999518823623657265625 0.0000000000000000006776263578034402712546579997594118118286328125 0.00000000000000000033881317890172013562732899987970590591431640625 0.000000000000000000169406589450860067813664499939852952957158203125 0.0000000000000000000847032947254300339068322499699264764785791015625 0.00000000000000000004235164736271501695341612498496323823928955078125 0.000000000000000000021175823681357508476708062492481619119644775390625 0.0000000000000000000105879118406787542383540312462408095598223876953125 0.00000000000000000000529395592033937711917701562312040477991119384765625 0.000000000000000000002646977960169688559588507811560202389955596923828125 0.0000000000000000000013234889800848442797942539057801011949777984619140625 0.00000000000000000000066174449004242213989712695289005059748889923095703125 0.000000000000000000000330872245021211069948563476445025298744449615478515625 0.0000000000000000000001654361225106055349742817382225126493722248077392578125 0.00000000000000000000008271806125530276748714086911125632468611240386962890625 0.000000000000000000000041359030627651383743570434555628162343056201934814453125 0.0000000000000000000000206795153138256918717852172778140811715281009674072265625 0.00000000000000000000001033975765691284593589260863890704058576405048370361328125 0.000000000000000000000005169878828456422967946304319453520292882025241851806640625 0.0000000000000000000000025849394142282114839731521597267601464410126209259033203125 0.00000000000000000000000129246970711410574198657607986338007322050631046295166015625 0.000000000000000000000000646234853557052870993288039931690036610253155231475830078125 0.0000000000000000000000003231174267785264354966440199658450183051265776157379150390625 0.00000000000000000000000016155871338926321774832200998292250915256328880786895751953125 0.000000000000000000000000080779356694631608874161004991461254576281644403934478759765625 0.0000000000000000000000000403896783473158044370805024957306272881408222019672393798828125 0.00000000000000000000000002019483917365790221854025124786531364407041110098361968994140625 0.000000000000000000000000010097419586828951109270125623932656822035205550491809844970703125 0.0000000000000000000000000050487097934144755546350628119663284110176027752459048519553515625 0.00000000000000000000000000252435489670723777731753140598316420550880138762295242597767578125 0.000000000000000000000000001262177448353618888658765702991582102754400693811476212988837890625 0.0000000000000000000000000006310887241768094443293828511957910513772003469057381064944169453125 0.00000000000000000000000000031554436208840472216469142559789552568860017345286905324720847265625 0.000000000000000000000000000157772181044202361082345712798947762844300086726434526623604236328125 0.0000000000000000000000000000788860905221011805411728563994738814221500433632172633166521181640625 0.00000000000000000000000000003944304526105059027058642819973694071107502168160863165832605908203125 0.000000000000000000000000000019721522630525295135293214099868470355537510840804315829163029541015625 0.0000000000000000000000000000098607613152626475676466070499342351777687554204021579145815147705078125 0.00000000000000000000000000000493038065763132378382330352496711758888437771020107895729075738525390625 0.000000000000000000000000000002465190328815661891911651762483558794442188855100539478645378692626953125 0.0000000000000000000000000000012325951644078309459558258812417793972210944275502697393226893463134765625 0.00000000000000000000000000000061629758220391547297791294062088969861054721377513486966134467315673828125 0.000000000000000000000000000000308148791101957736488956470310444849305273606887567434830672336578369140625 0.0000000000000000000000000000001540743955509788682444782351552224246526368034437837174153361682891845703125 0.00000000000000000000000000000007703719777548943412223911757761121232631840172189185870766808414459228515625 0.000000000000000000000000000000038518598887744717061119558788805606163159200860945929353834042072296142578125 0.0000000000000000000000000000000192592994438723585305597793944028030815796004304729646769170210361480712890625 0.00000000000000000000000000000000962964972193617926527988969722014015078980021523648233845851051807403564453125 0.000000000000000000000000000000004814824860968089632639944848610070075394900107618241169229255259037017822265625 0.0000000000000000000000000000000024074124304840448163199724243050350376974500538091205846146276295185089111328125 0.00000000000000000000000000000000120370621524202240815998621215251751884872502690456029230731381475925445556640625 0.000000000000000000000000000000000601853107621011204079993106076258759424362513452280146153656907379627227783203125 0.0000000000000000000000000000000003009265538105056020399965530381293797121812567261400730768284536898136138916015625 0.00000000000000000000000000000000015046327690525280101999827651906468985609062836307003653841422684490680694580078125 0.000000000000000000000000000000000075231638452626400509999138259532344928045314181535001826920713422453403472900390625 0.0000000000000000000000000000000000376158192263132002549995691297661724640226570907675009134603567112267017364501953125 0.00000000000000000000000000000000001880790961315660012749978456488308623201132854538375045673017835561335086822509765625 0.000000000000000000000000000000000009403954806578300063749892282441543116005664272691875022865089177806675434112548828125 0.0000000000000000000000000000000000047019774032891500318749461412207715580028321363459375114325445889033377170562744140625 0.00000000000000000000000000000000000235098870164457501593747307061038577900141606817296875571627229445166885852813720703125 0.000000000000000000000000000000000001175494350822287507968736535305192889500708034086484377858136147225834429264068603515625 0.0000000000000000000000000000000000005877471754111437539843682676525964447500354017032421889290680736129172146320343017578125 0.00000000000000000000000000000000000029387358770557187699218413382629822237501770085162109446453403680645860731601715087890625 0.000000000000000000000000000000000000146936793852785938496092066913149111187500850042581047232267018403229303658008575438953125 0.0000000000000000000000000000000000000734683969263929692480460334565745555937504250212905236161133542016146518290042877194765625 0.00000000000000000000000000000000000003673419846319648462402301672828727779687521251104526180805667710080732591450214385973828125 0.000000000000000000000000000000000000018367099231598242312011508364143638898437606255522630904028338550403662957251071929869140625 0.0000000000000000000000000000000000000091835496157991211560057541820718194492188031277613154520141667752018314786255359649345703125 0.00000000000000000000000000000000000000459177480789956057800287709103590972460940156388065772600708338760091573931276798246728515625 0.000000000000000000000000000000000000002295887403949780289001438545517954862304700781940328863003541693800457869656383991233642578125 0.0000000000000000000000000000000000000011479437019748901445007192727589774311523503909701644315017708469002289348281919956168212890625 0.00000000000000000000000000000000000000057397185098744507225035963637948871557617519548508221575088542345011446741409599780841064453125 0.000000000000000000000000000000000000000286985925493722536125179818189744357788087597742541107875442711725057233707047998904205322265625 0.0000000000000000000000000000000000000001434929627468612680625899090948721788940437988712705539377213558625286168535239994521026611328125 0.00000000000000000000000000000000000000007174648137343063403129495454723608944702189943563527696886067793126430842676199972605133056640625 0.000000000000000000000000000000000000000035873240686715317015647477273618044723510949717817638484430338965632154213380999863025665283203125 0.0000000000000000000000000000000000000000179366203433576585078237386368090223617554748589088192422151694828160771066904999315128326416015625 0.00000000000000000000000000000000000000000896831017167882925391186931840451118087773724295440962110758474140803855334524996575641632080078125 0.000000000000000000000000000000000000000004484155085839414626955934659202255590438868621477204810553792370704019276672624982878208160400390625 0.0000000000000000000000000000000000000000022420775429197073134779673296011277952194343107386024052768961853520096383363124914391040802001953125 0.00000000000000000000000000000000000000000112103877145985365673898366480056389760971715536930120263844809267600481916815624571955204010009765625 0.0000000000000000000000000000000000000000005605193857299268283694918324002819488048585776846506013192240463380024095840781242859576020050048828125 0.00000000000000000000000000000000000000000028025969286496341418474591620014097440242928884232530065961202316900120479203906214297880100250244140625 0.00000000000000000000000000000000000000000014012984643248170709237295810007048720121464442116265032980601158450060239601953107148940050012502222140625 0.000000000000000000000000000000000000000000070064923216240853546186479050035243600607322210581325164903005792250030119600976535744700250006251111328125 0.00000000000000000000000000000000000000000003503246160812042677309323952501762180030366110529066258245150289612501505980048826787235012500031255556640625 0.000000000000000000000000000000000000000000017516230804060213386546619762508810900151830552645331291225751448062507529900244133936175062500015627778125 0.000000000000000000000000000000000000000000008758115402030106693273309881254405450075915276322665645612875724031250376495012206696887503125000078138890625 0.000000000000000000000000000000000000000000004379057701015053346636654940627202725037957638161332822806437862015625018822506103348443751562500000394694453125 0.00000000000000000000000000000000000000000000218952885050752667331832747031360136251897881908066641140321893100781250094112505516667187578125000001973472265625 0.00000000000000000000000000000000000000000000109476442525376333665916373515680068125948940954033320

Supplement: Supplementary file 12 — Source Data for Figure 2 [file EMBJ-41-e109191-s014.zip › Fig2/Fig2A_3AKO_UPF3B.pdf]

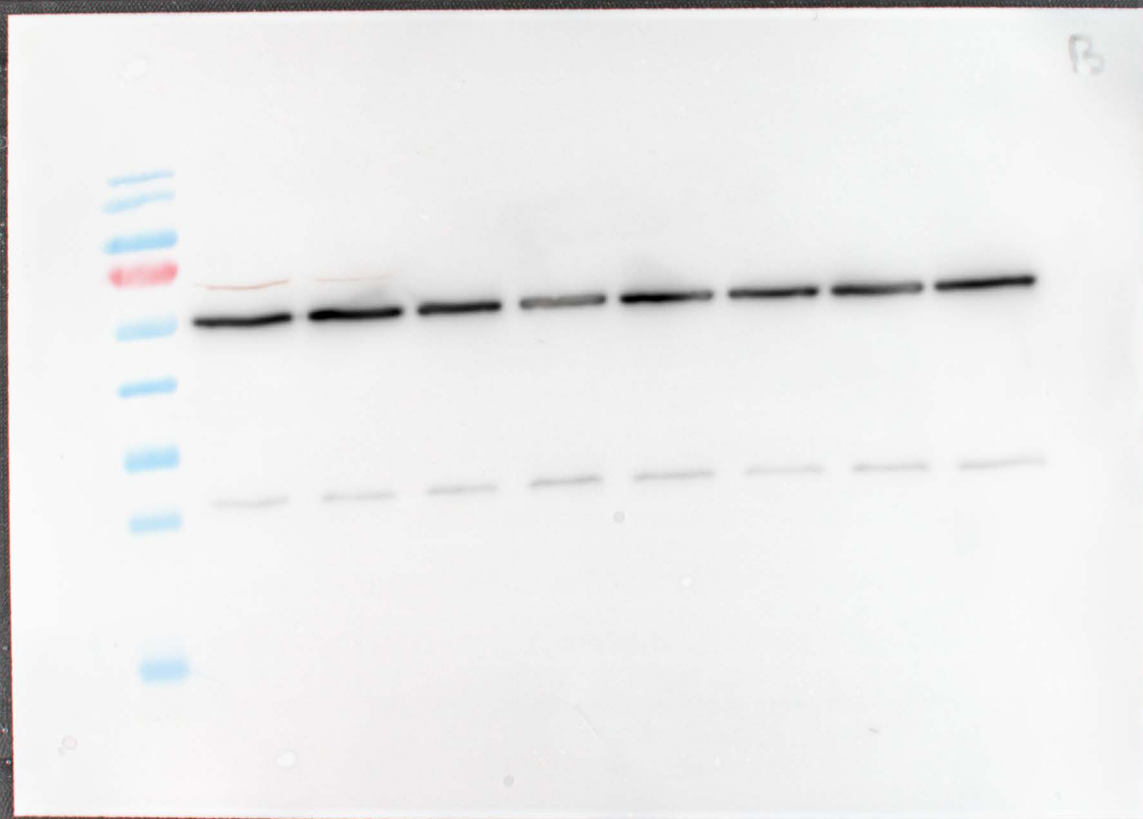

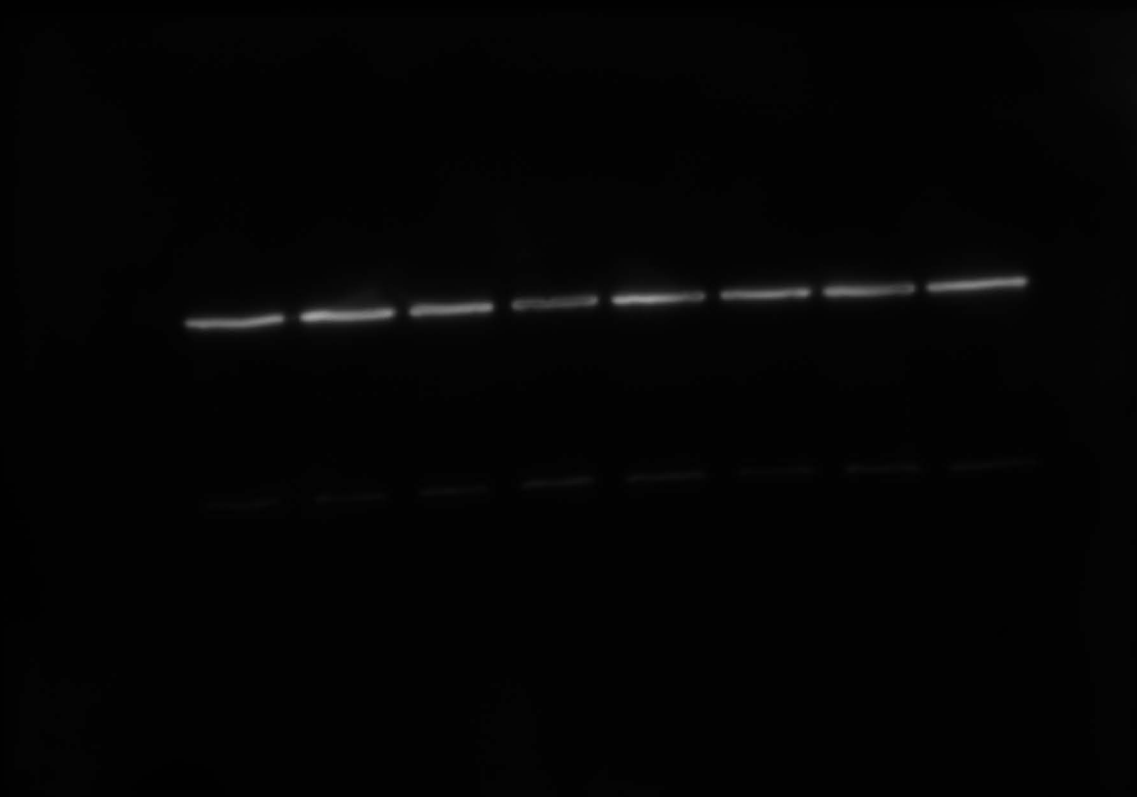

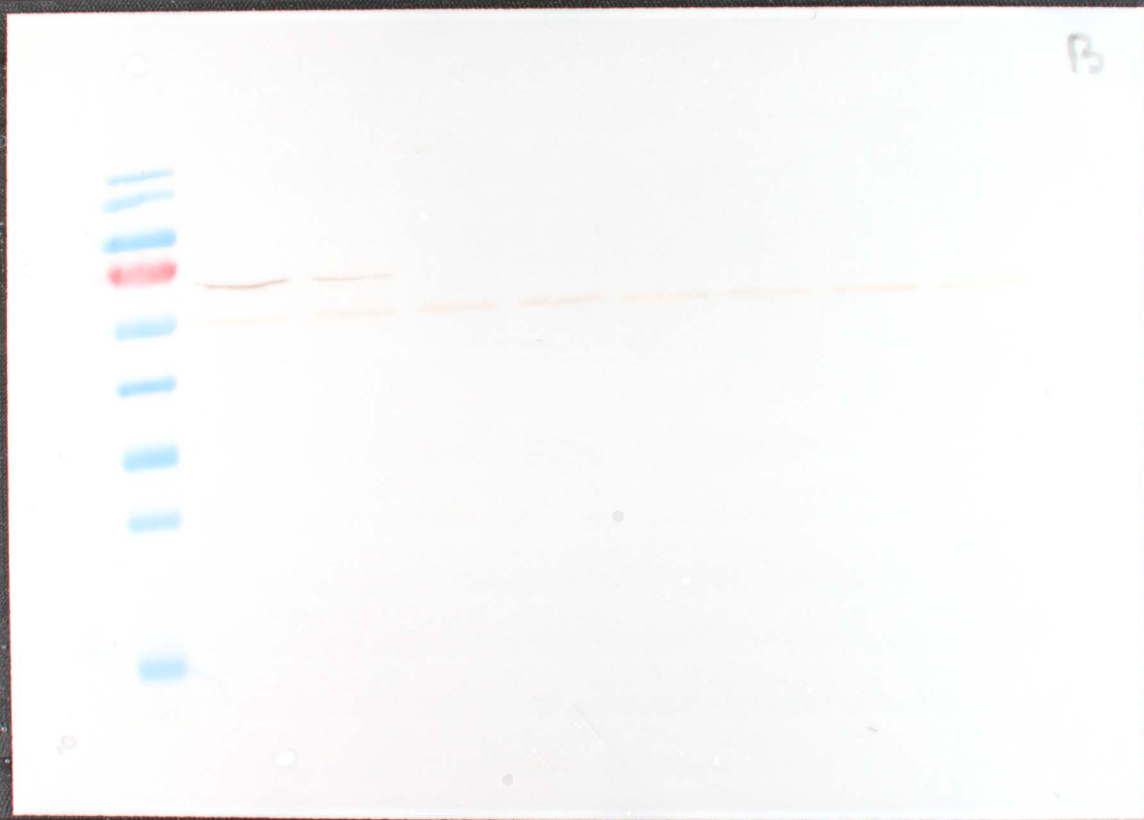

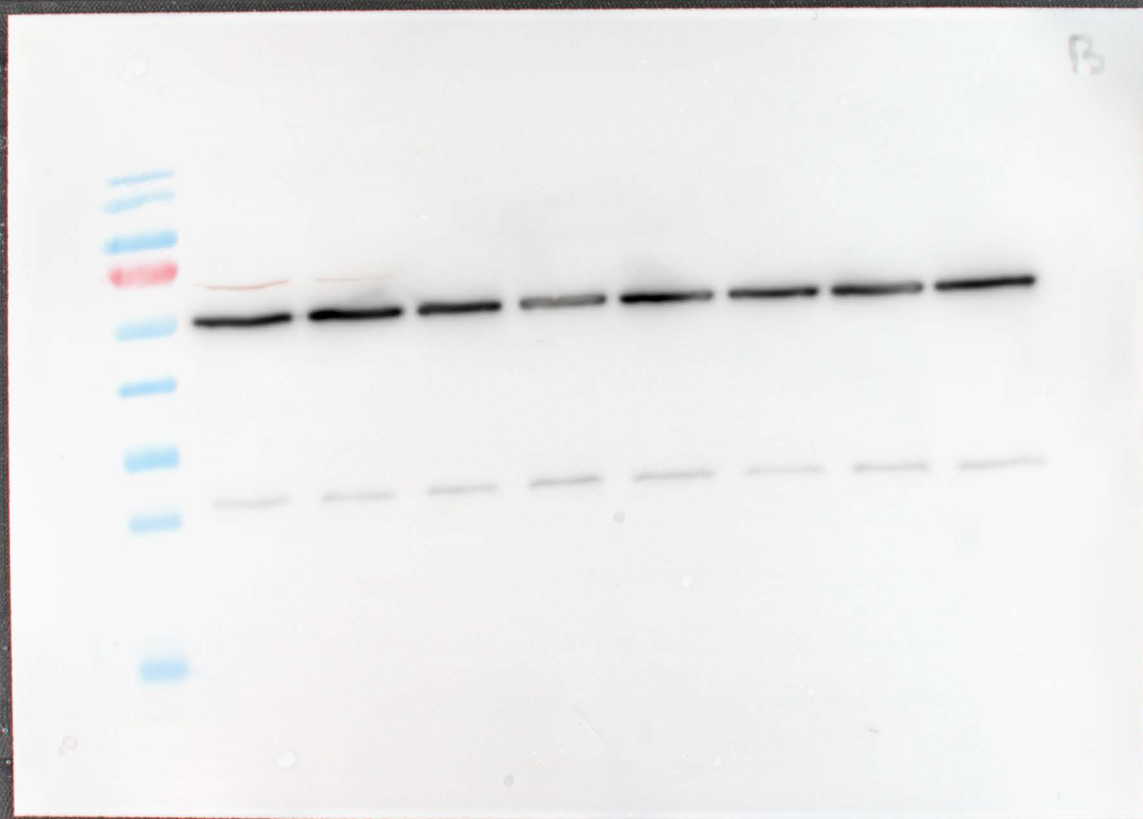

Supplement: Supplementary file 13 — Source Data for Figure 3 [file EMBJ-41-e109191-s011.zip › Fig3/Fig3A_UPF3B_KOs_Tubulin.pdf]

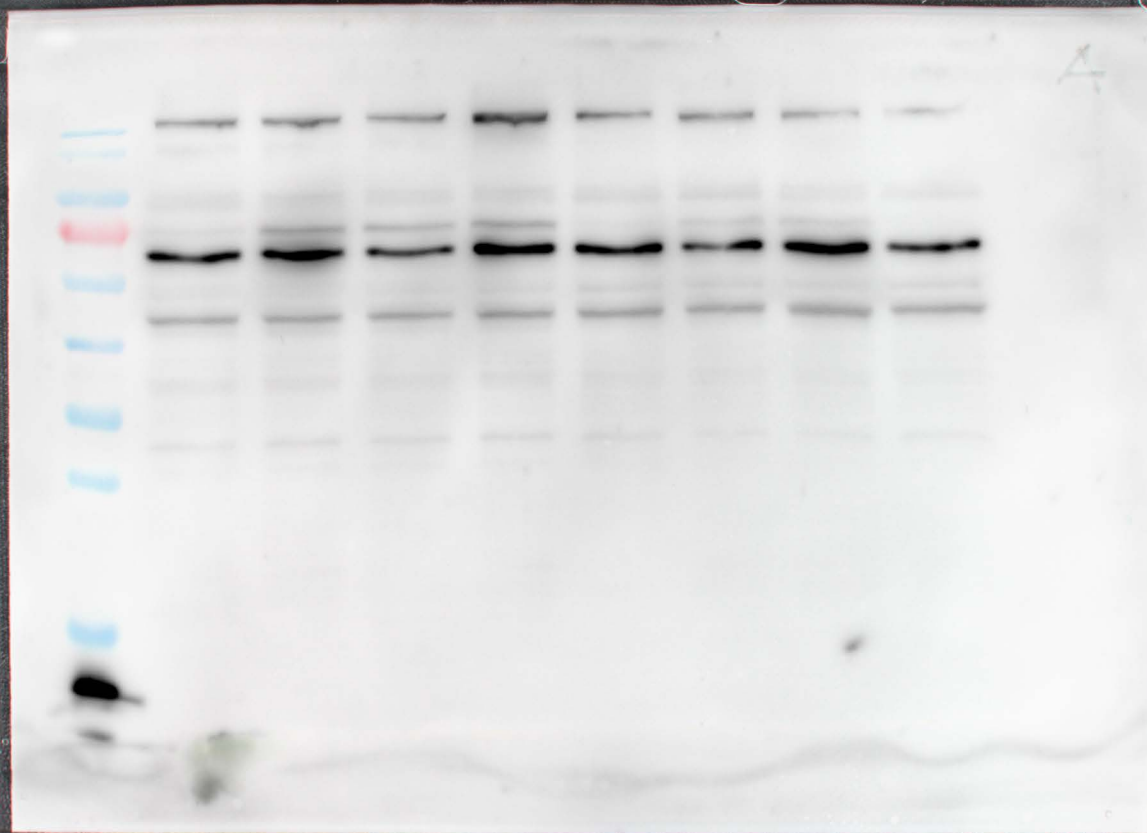

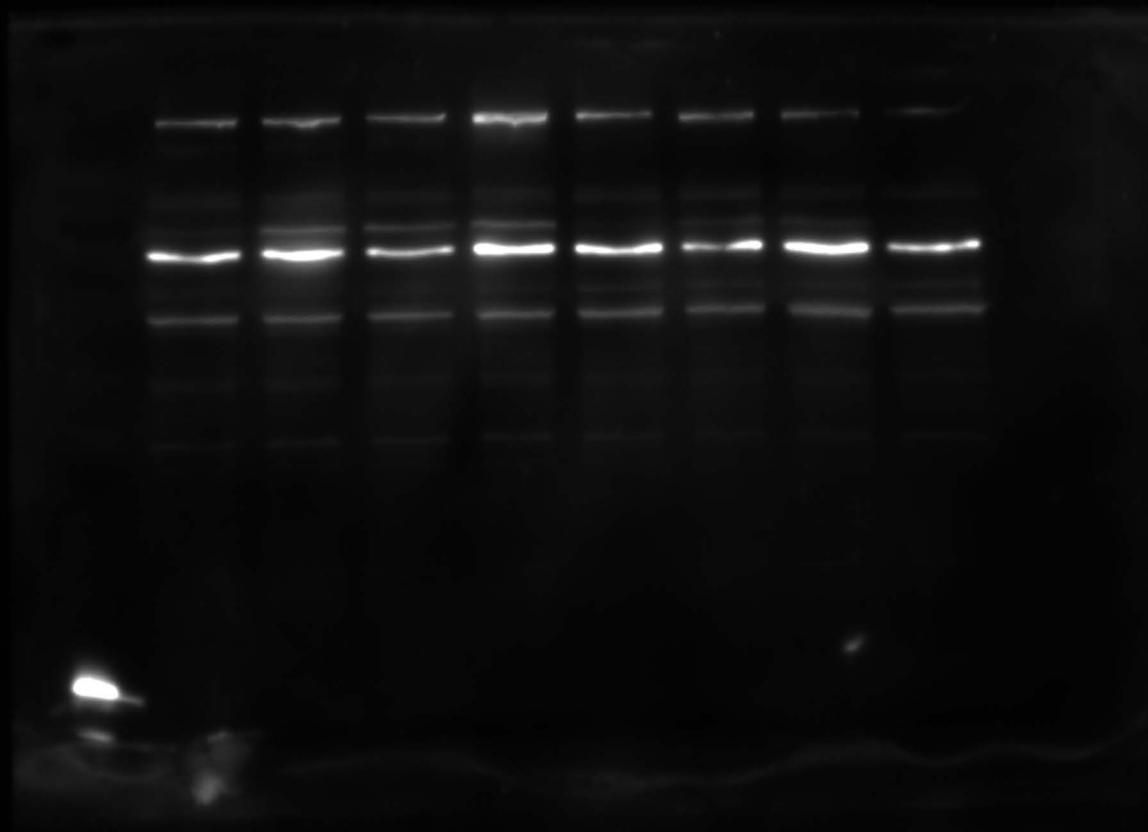

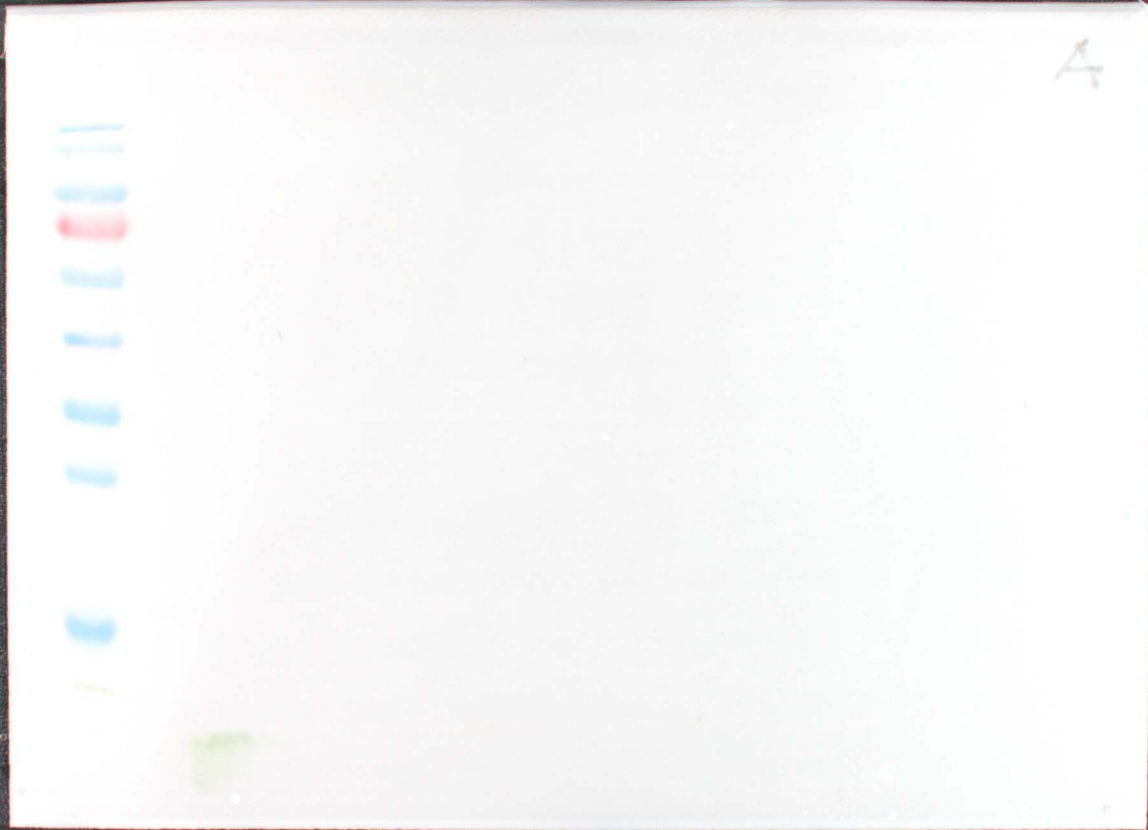

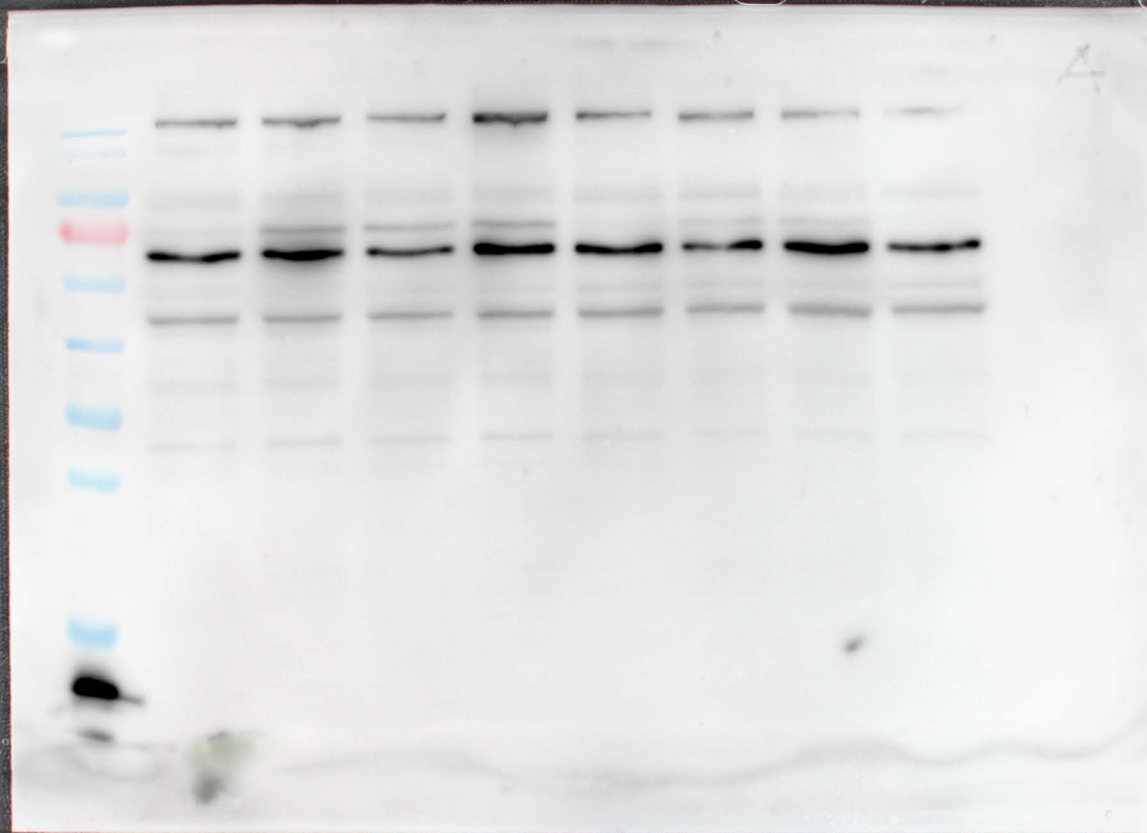

Supplement: Supplementary file 13 — Source Data for Figure 3 [file EMBJ-41-e109191-s011.zip › Fig3/Fig3A_UPF3B_KOs_UPF3A.pdf]

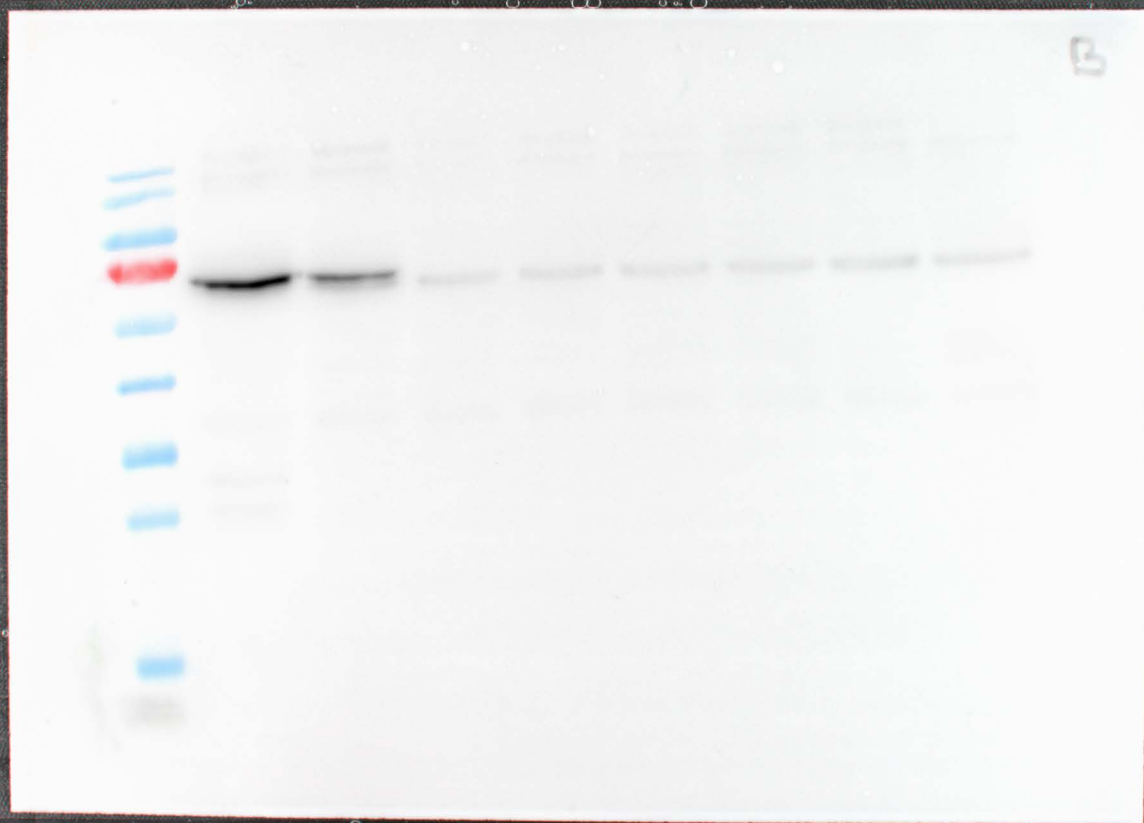

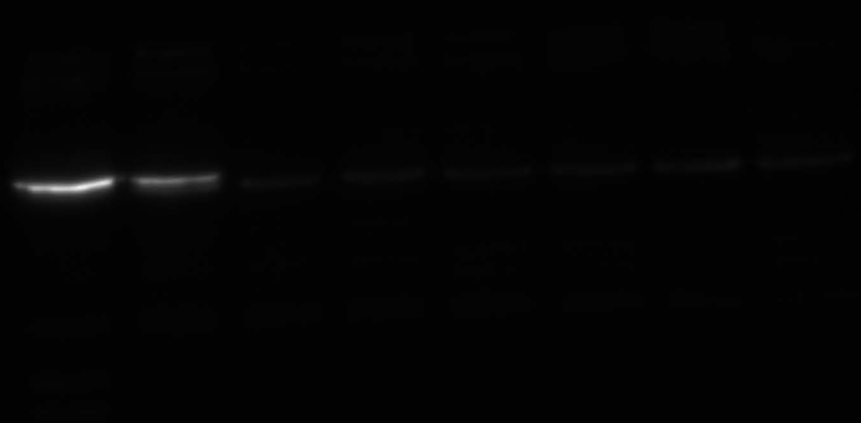

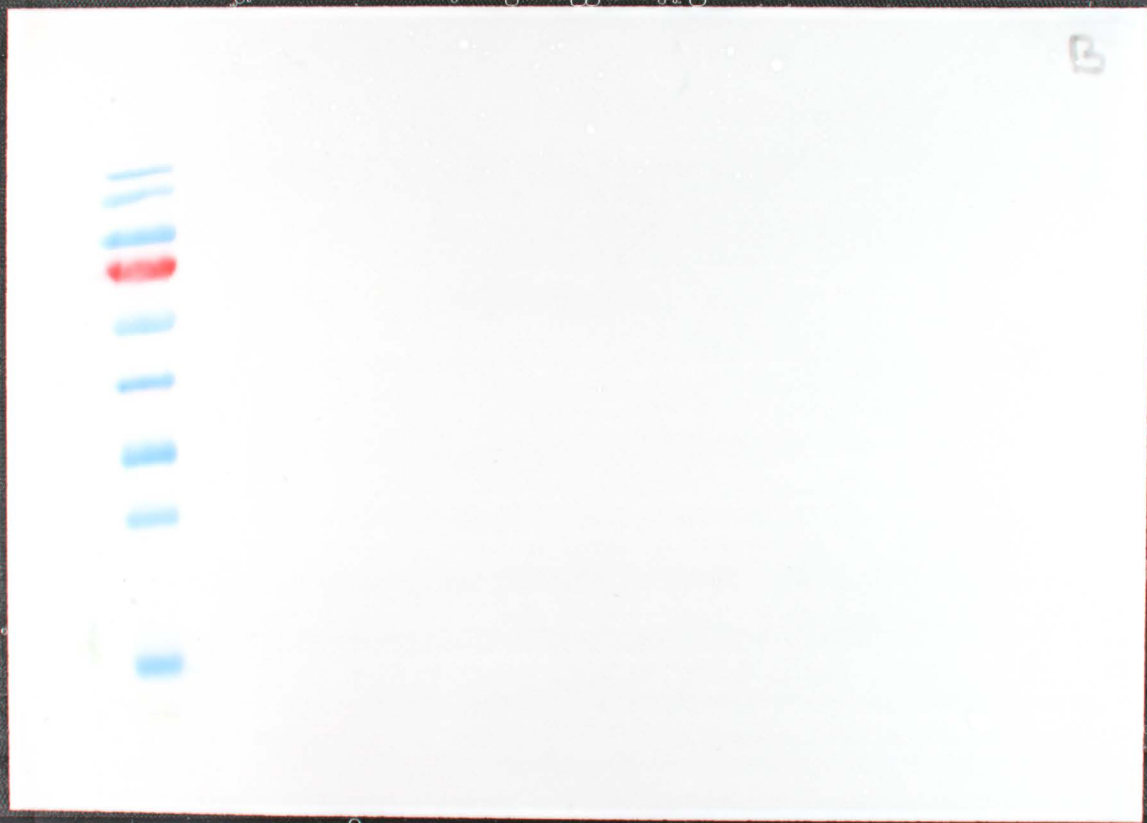

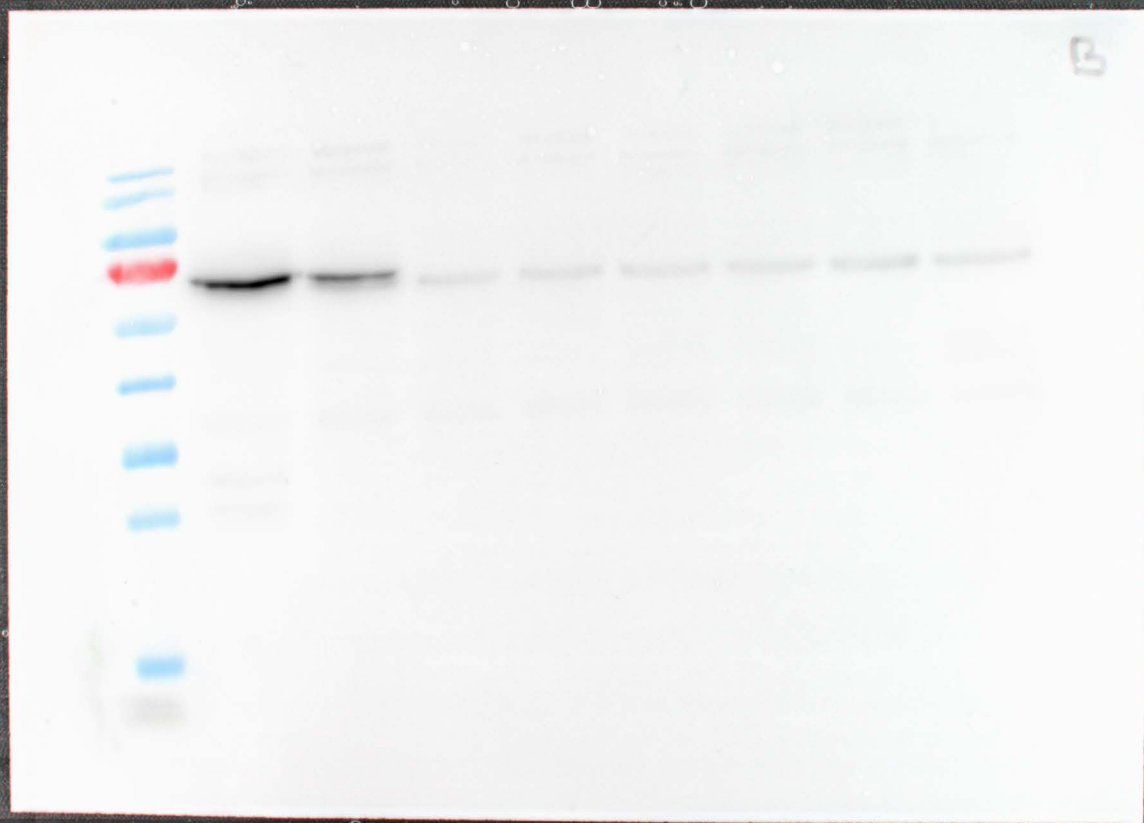

Supplement: Supplementary file 13 — Source Data for Figure 3 [file EMBJ-41-e109191-s011.zip › Fig3/Fig3A_UPF3B_KOs_UPF3B.pdf]

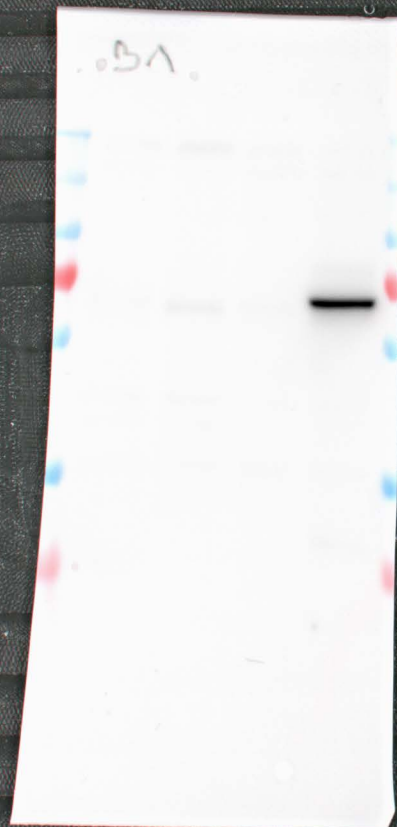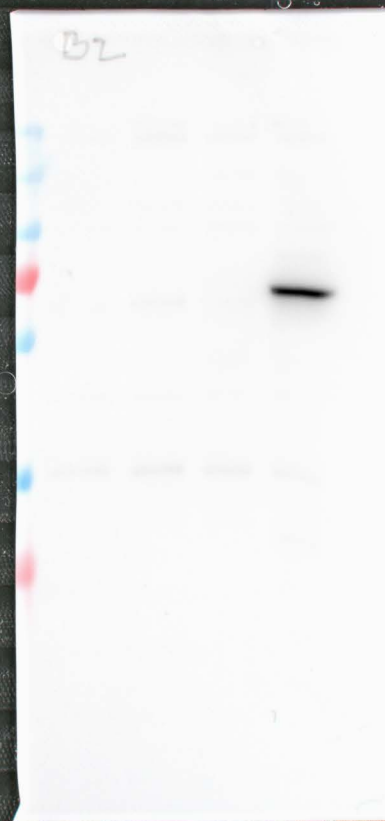

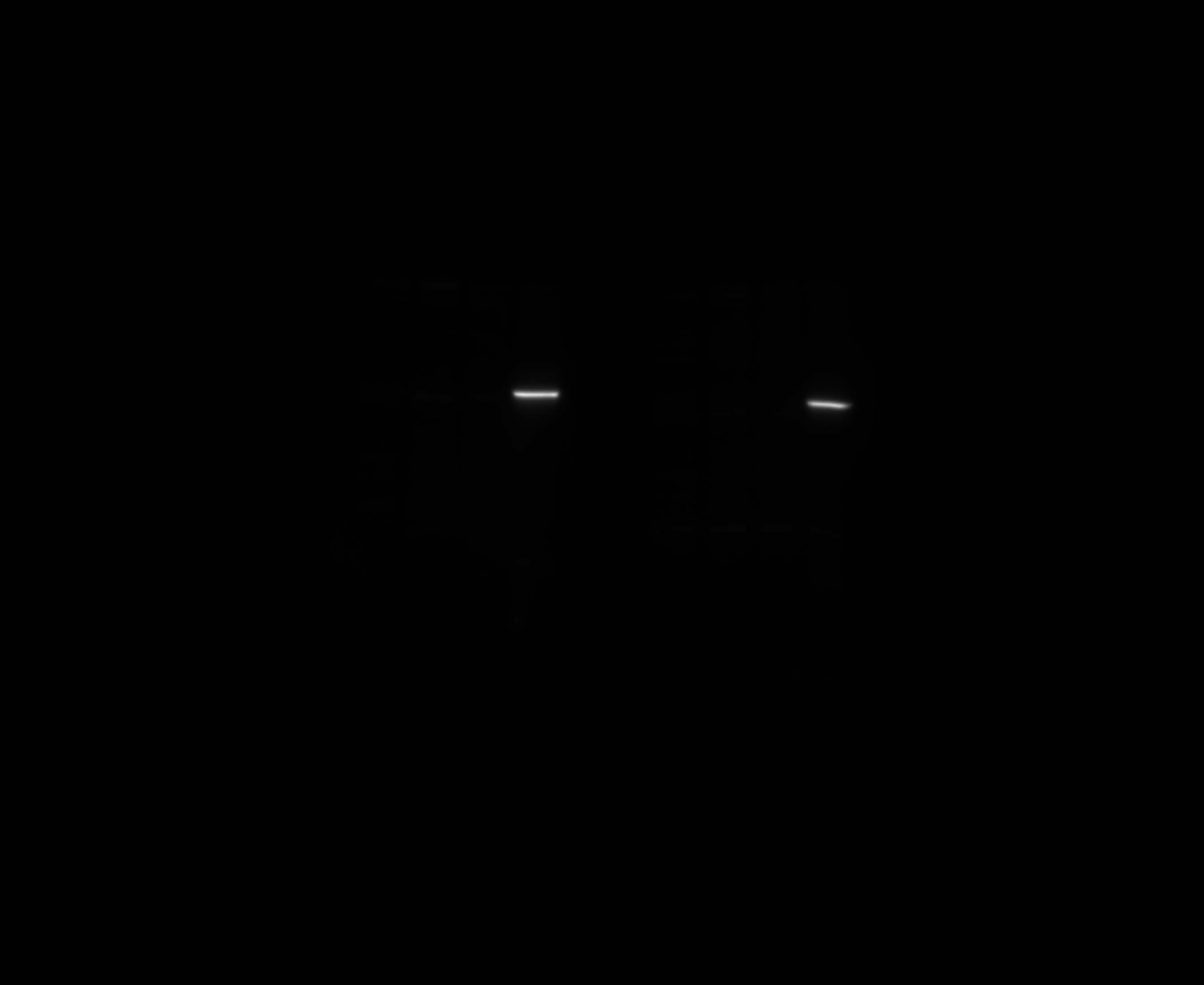

B1.

B2

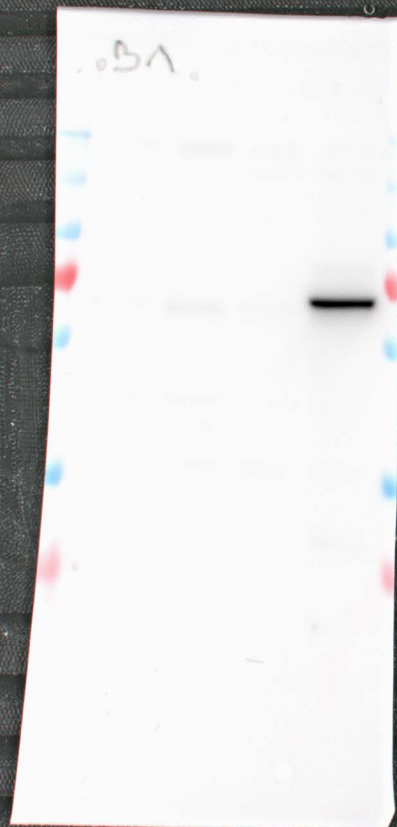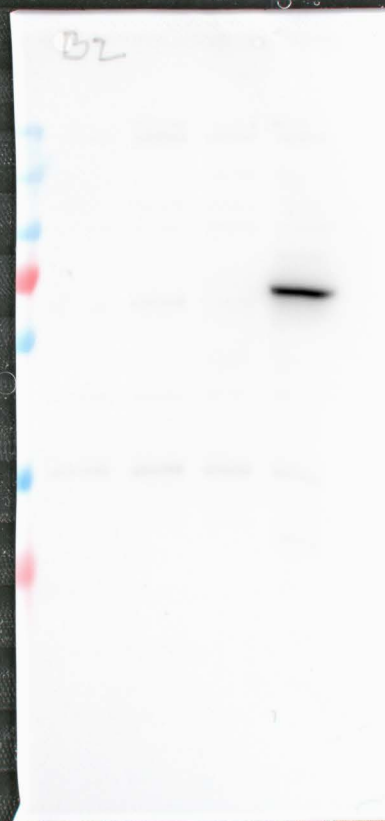

Supplement: Supplementary file 14 — Source Data for Figure 4 [file EMBJ-41-e109191-s010.zip › Fig4/Fig4A_UPF3_dKO_AK-141left_AK-157right_mirrored.pdf]

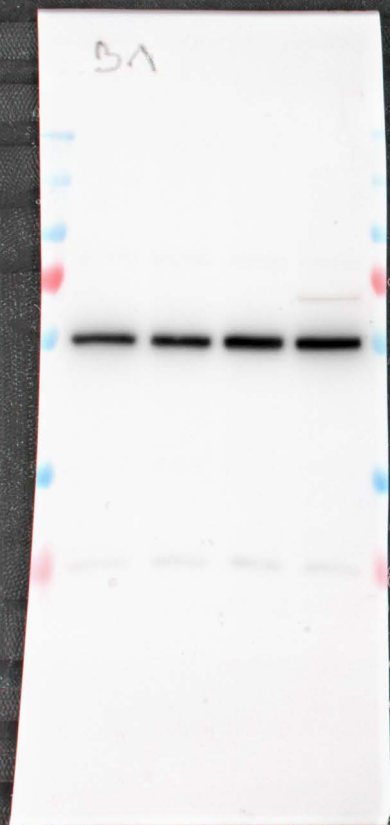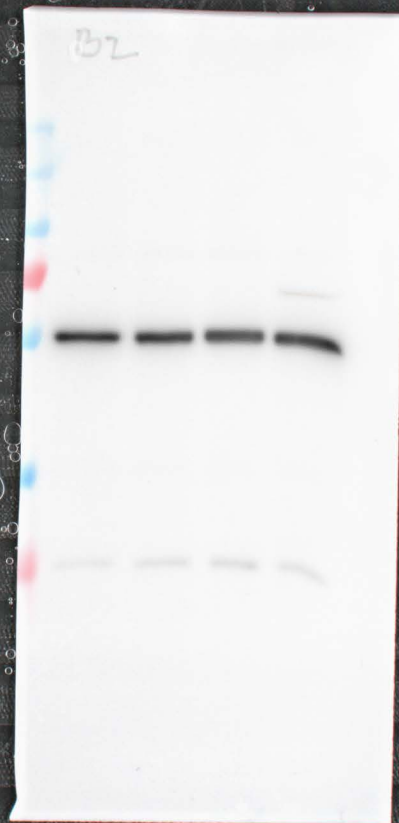

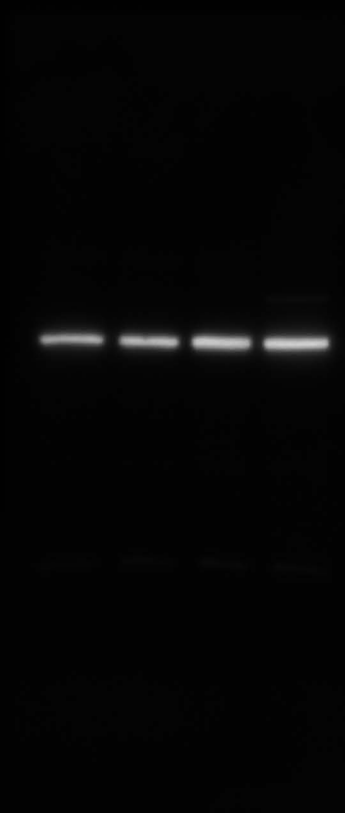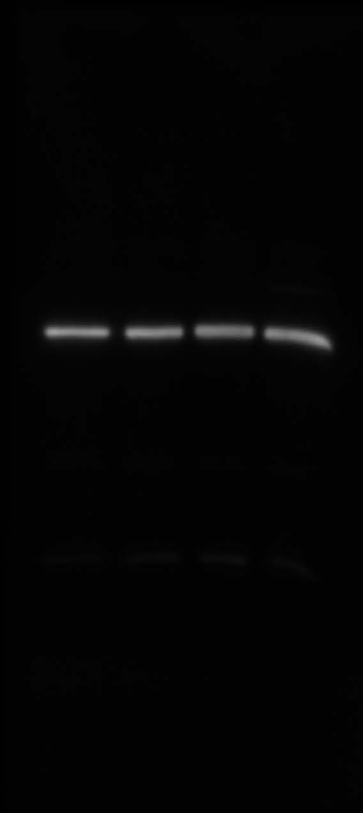

BA

BZ

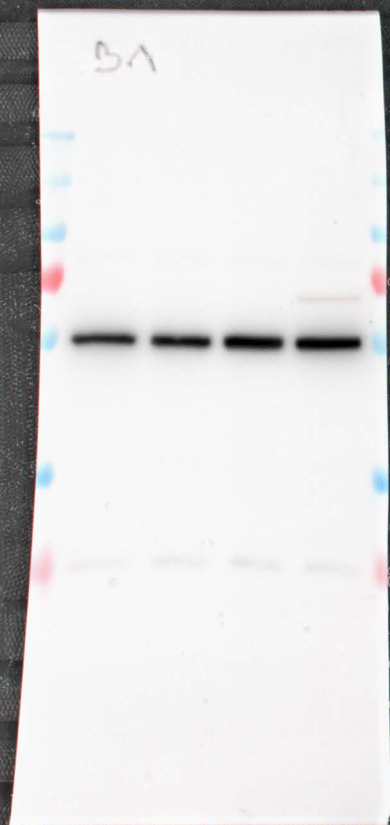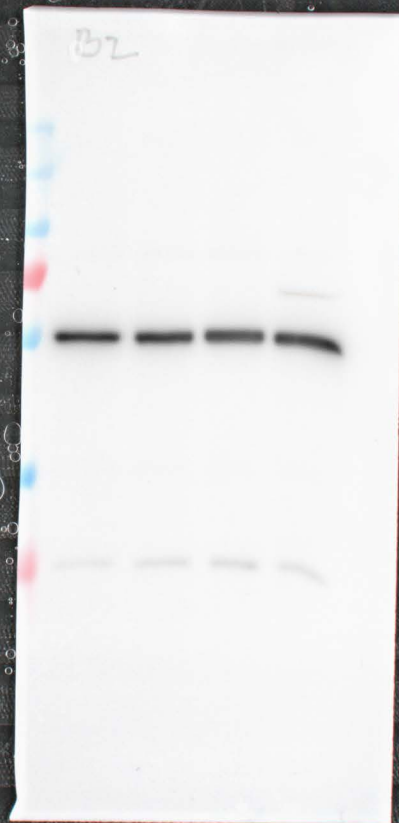

Supplement: Supplementary file 14 — Source Data for Figure 4 [file EMBJ-41-e109191-s010.zip › Fig4/Fig4A_UPF3_dKO_Tubulin_mirrored.pdf]

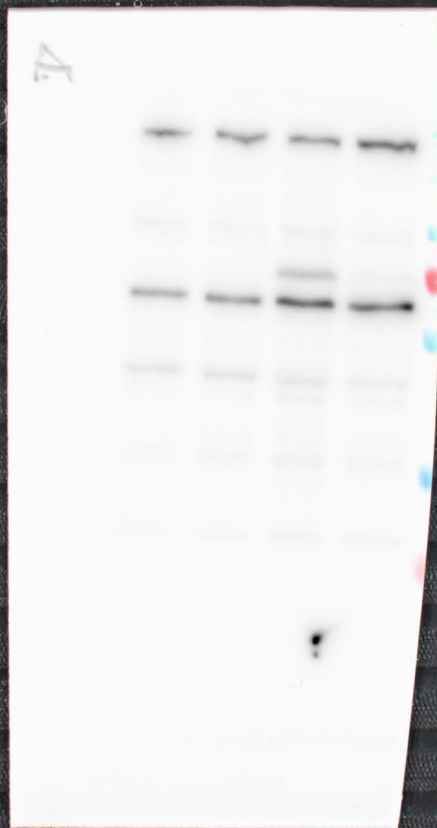



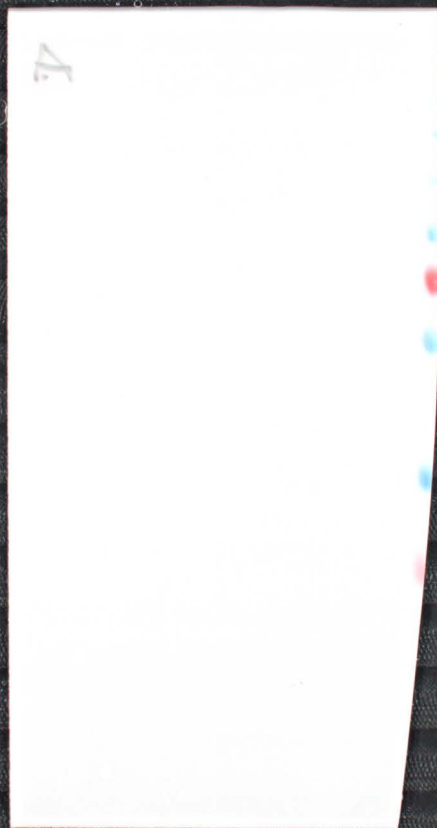

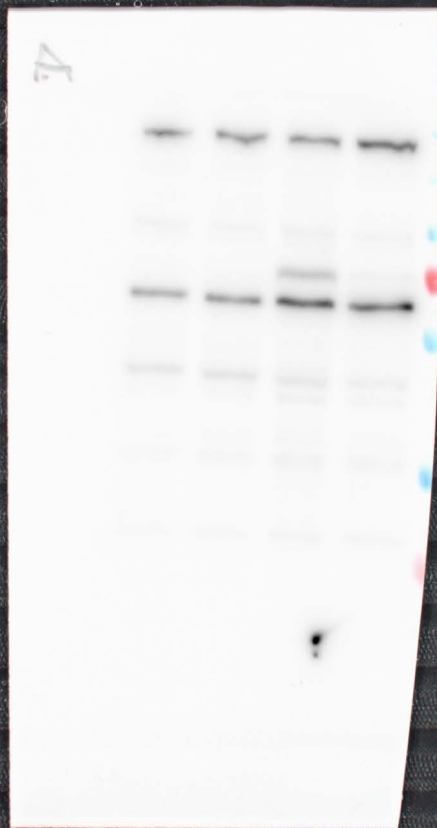

Supplement: Supplementary file 14 — Source Data for Figure 4 [file EMBJ-41-e109191-s010.zip › Fig4/Fig4A_UPF3_dKO_UPF3A_mirrored.pdf]

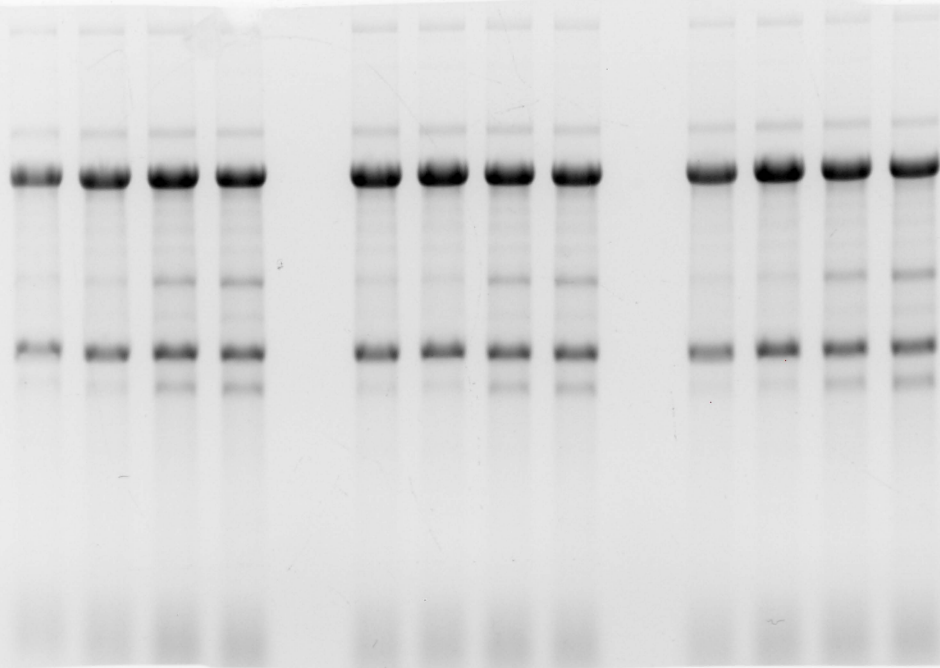

Supplement: Supplementary file 14 — Source Data for Figure 4 [file EMBJ-41-e109191-s010.zip › Fig4/Fig4E_NB_EtBr_gel.pdf]

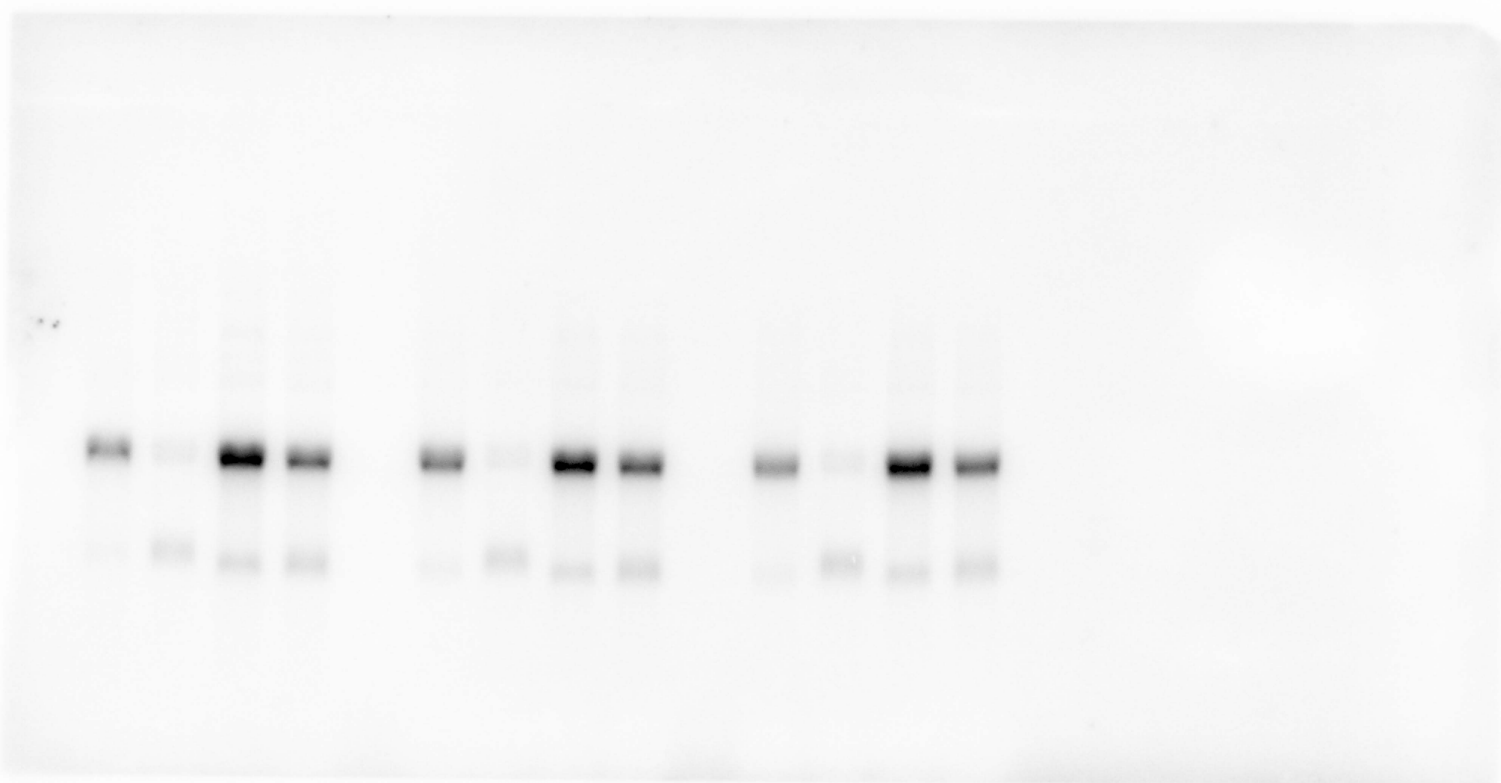

Supplement: Supplementary file 14 — Source Data for Figure 4 [file EMBJ-41-e109191-s010.zip › Fig4/Fig4E_NorthernBlot.pdf]

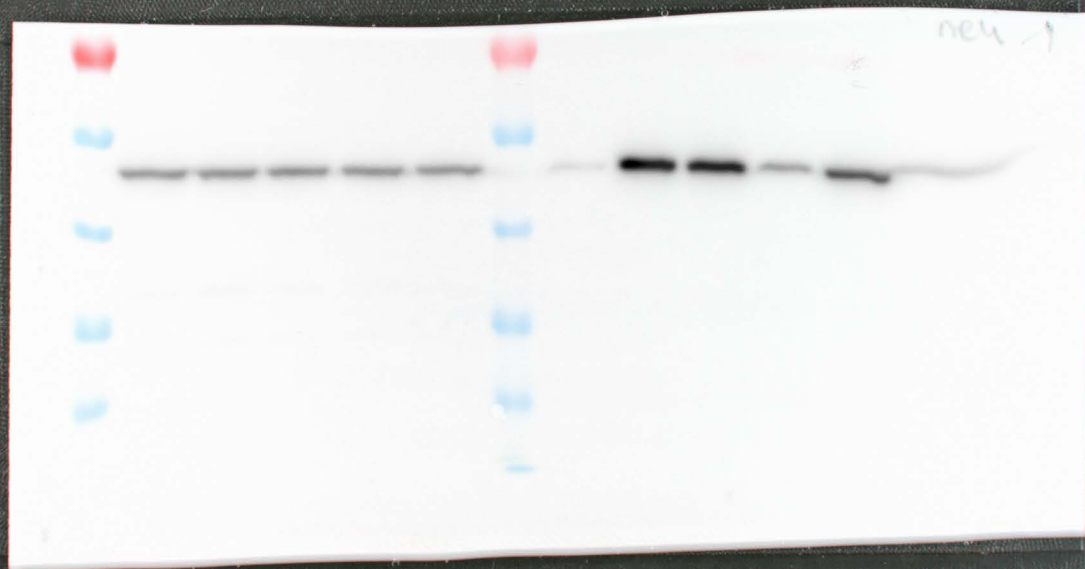

— — — — —

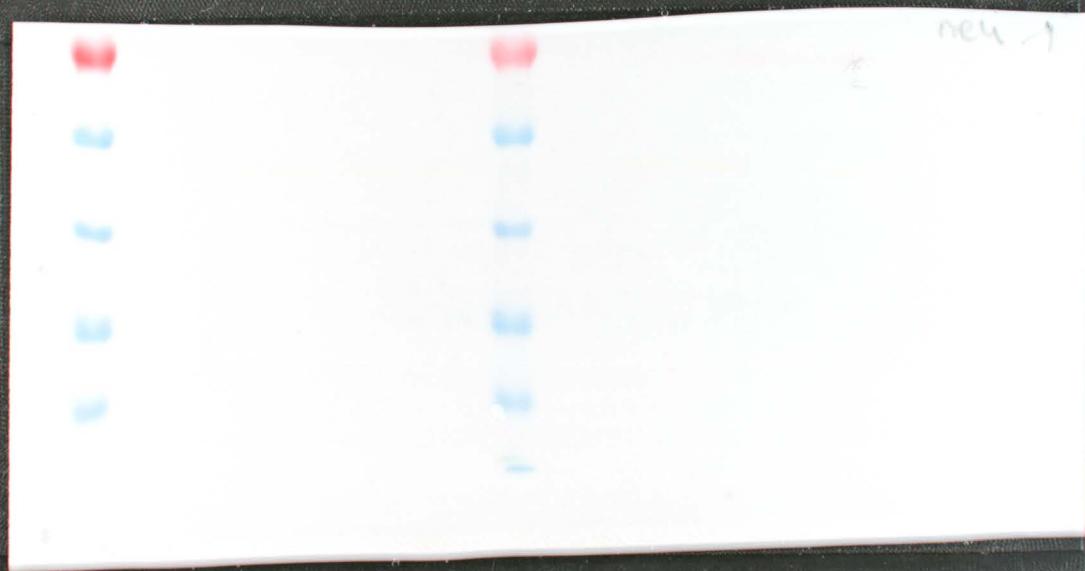

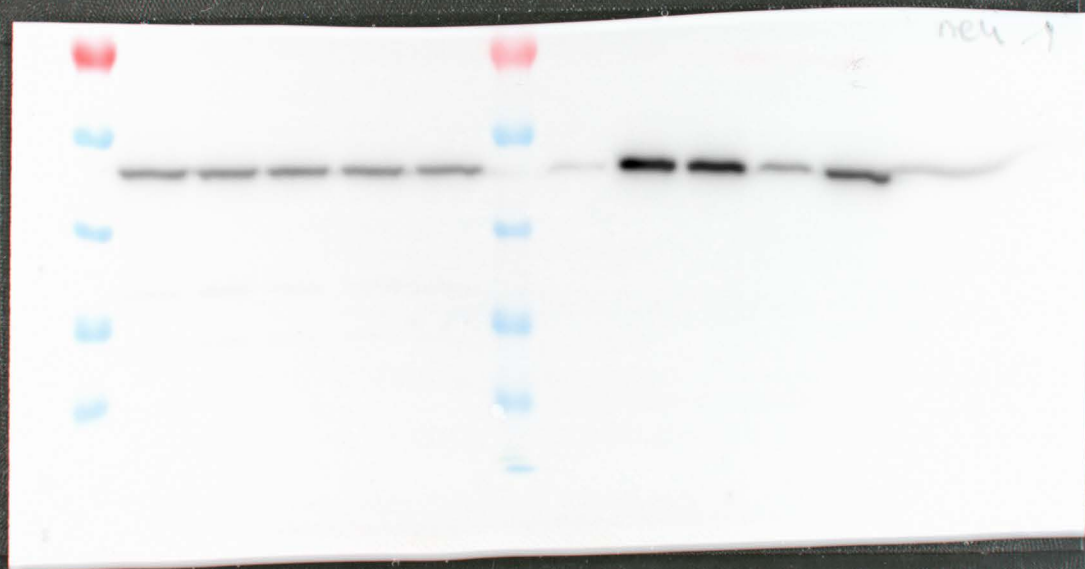

Supplement: Supplementary file 15 — Source Data for Figure 6 [file EMBJ-41-e109191-s005.zip › Fig6/Fig6E_FLAG-IP_EIF4A3.pdf]

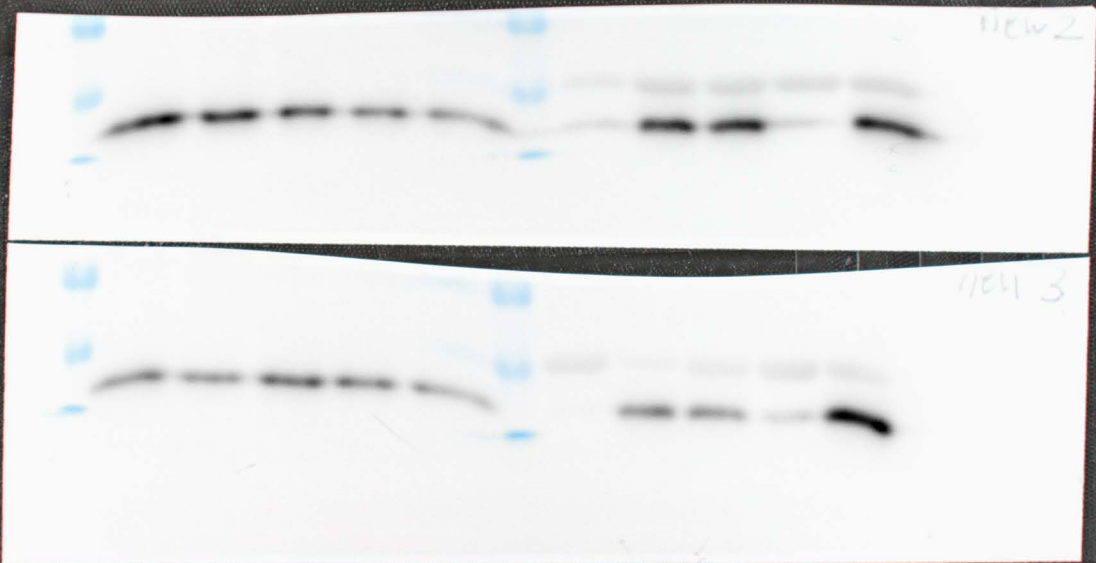

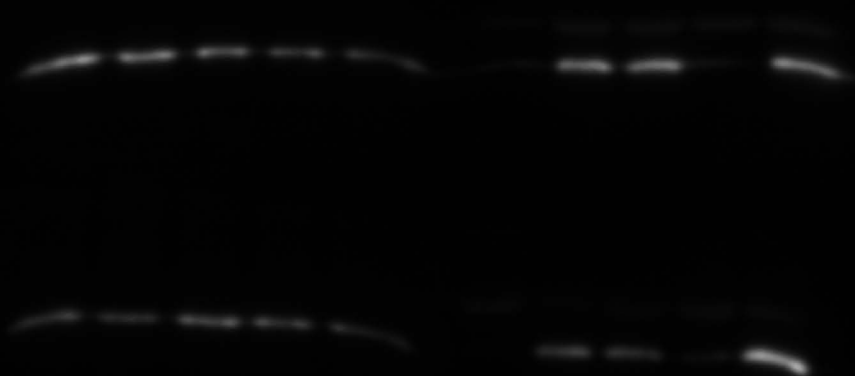

new 2

new 3

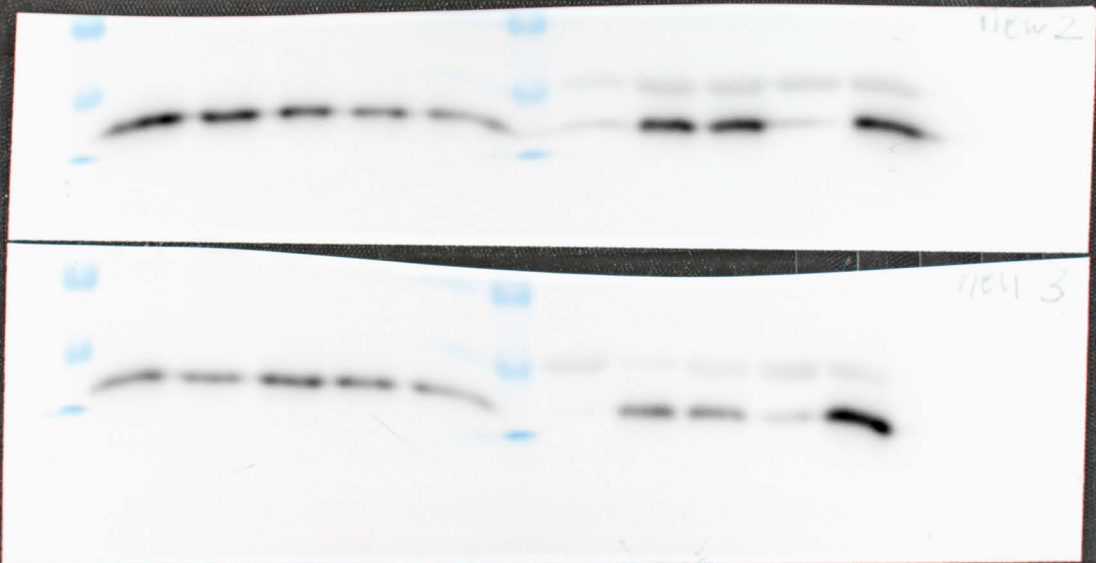

Supplement: Supplementary file 15 — Source Data for Figure 6 [file EMBJ-41-e109191-s005.zip › Fig6/Fig6E_FLAG-IP_RBM8A.pdf]

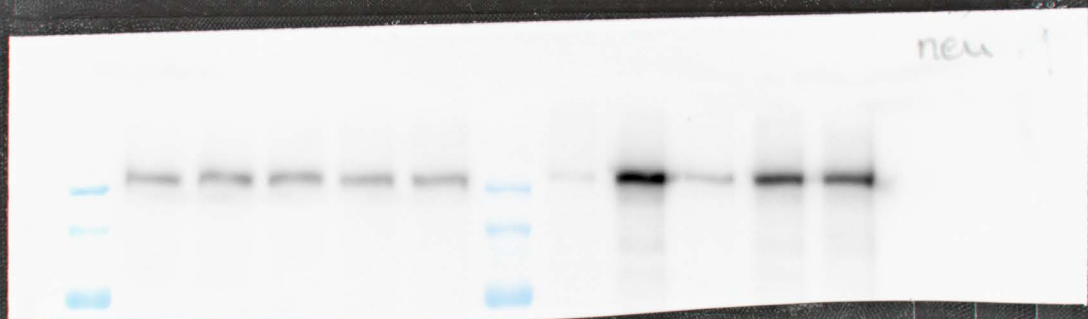



neu

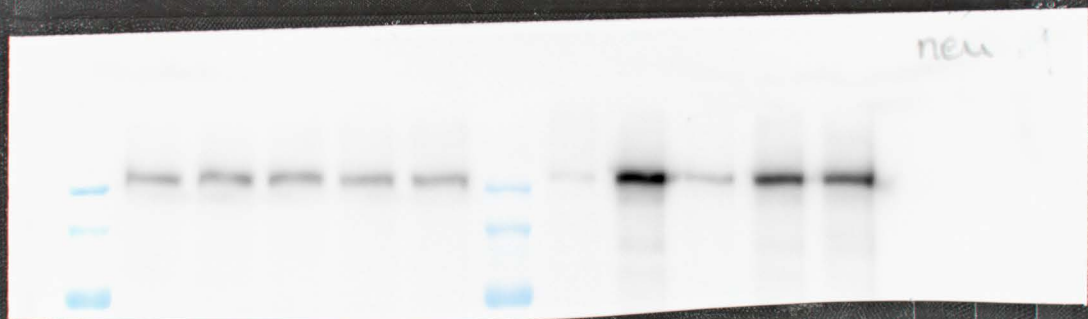

Supplement: Supplementary file 15 — Source Data for Figure 6 [file EMBJ-41-e109191-s005.zip › Fig6/Fig6E_FLAG-IP_UPF2.pdf]

FL R

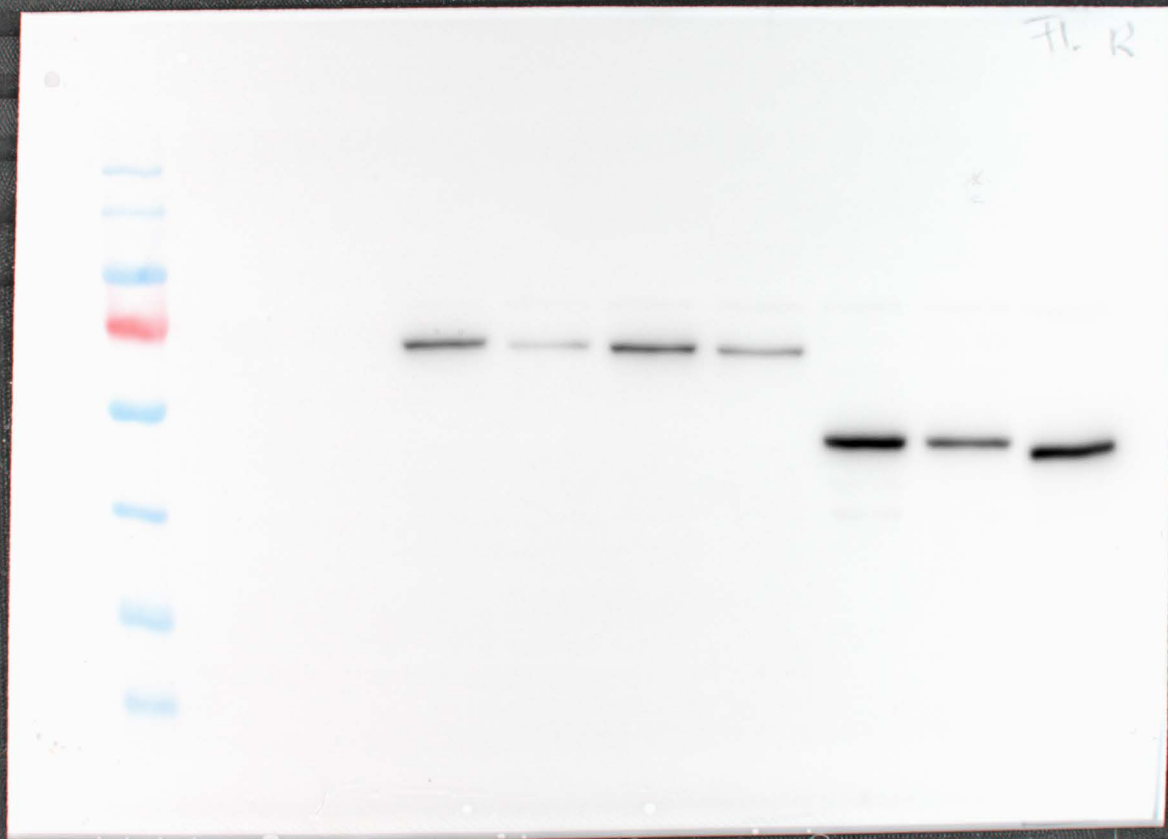

— — — —

— — —

FL R

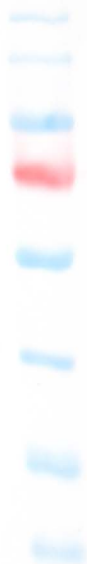

FL R

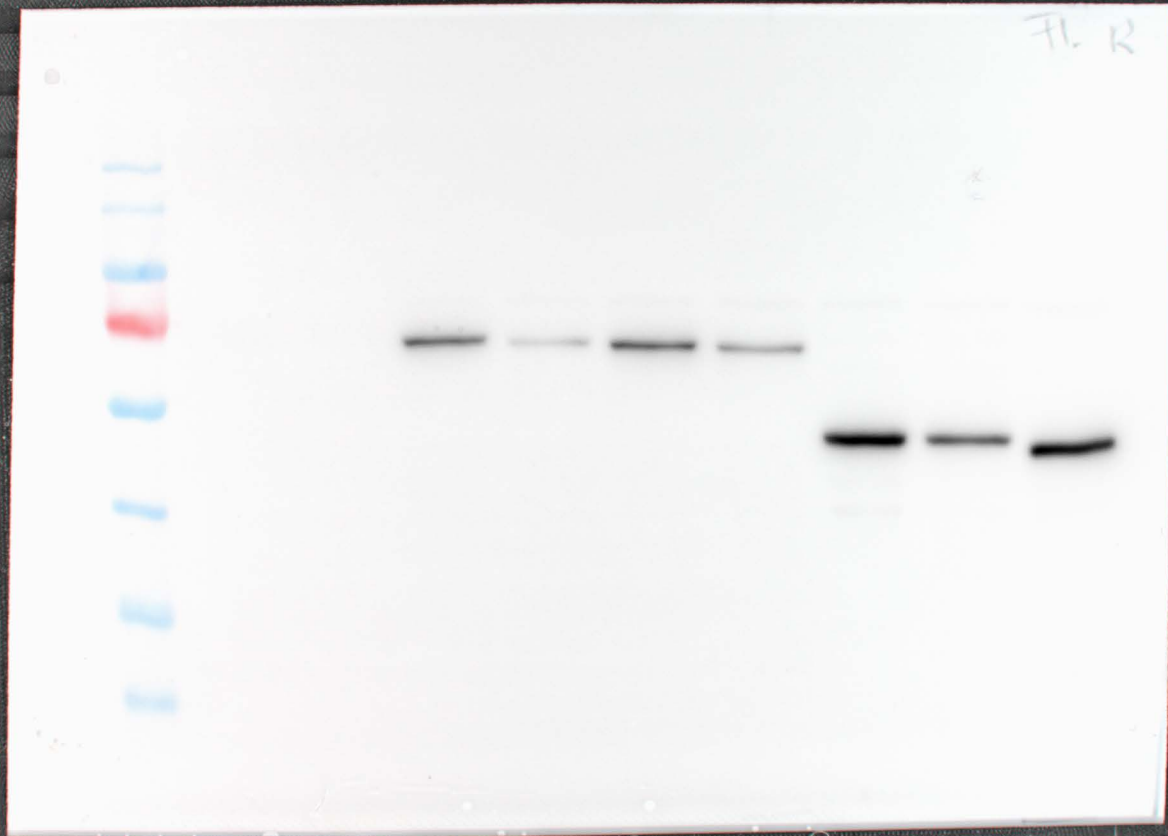

Supplement: Supplementary file 15 — Source Data for Figure 6 [file EMBJ-41-e109191-s005.zip › Fig6/Fig6F_Rescues_FLAG.pdf]

FL R

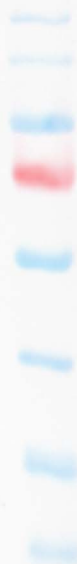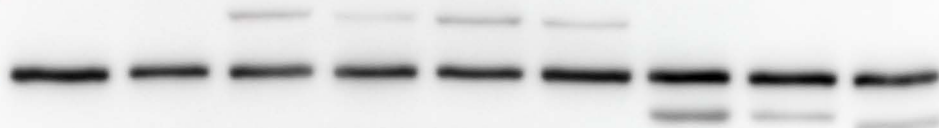

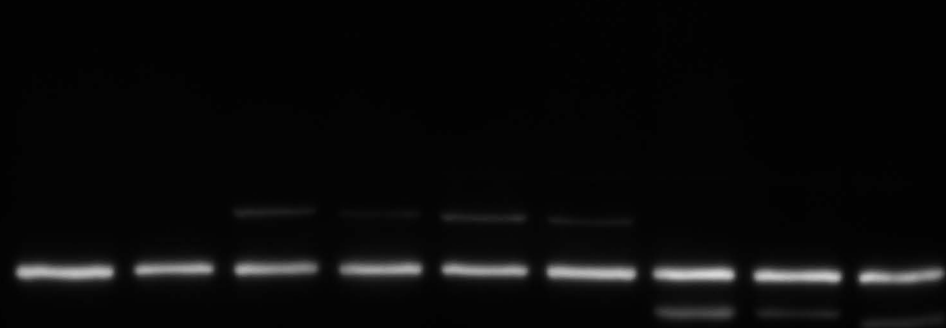

71 R

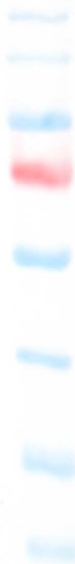

FL R

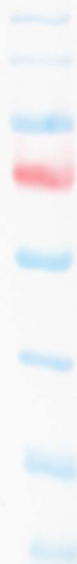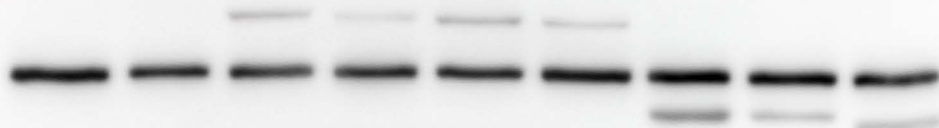

Supplement: Supplementary file 15 — Source Data for Figure 6 [file EMBJ-41-e109191-s005.zip › Fig6/Fig6F_Rescues_Tubulin.pdf]

3B 12es

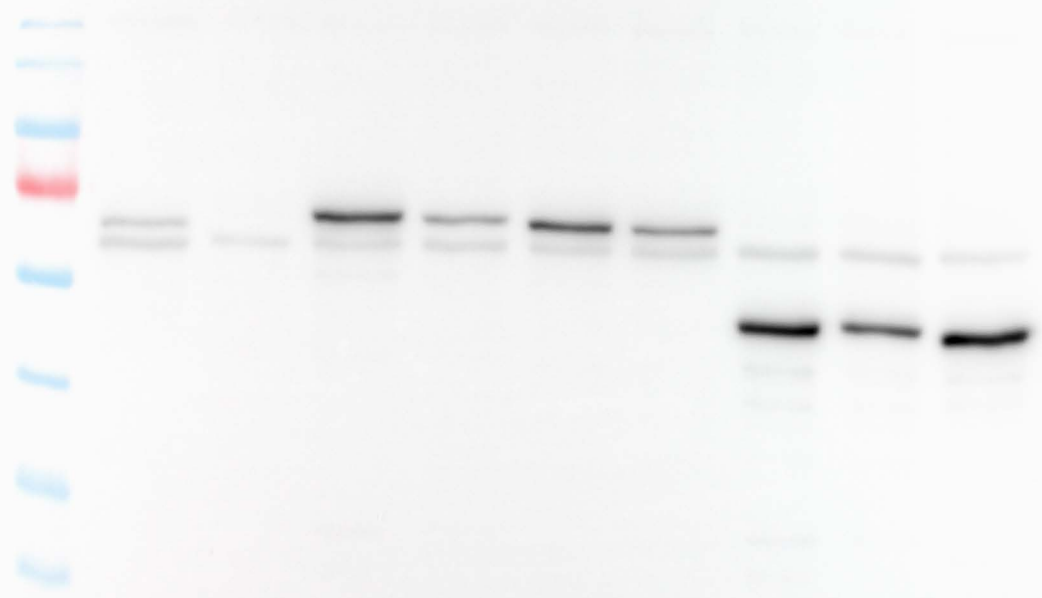

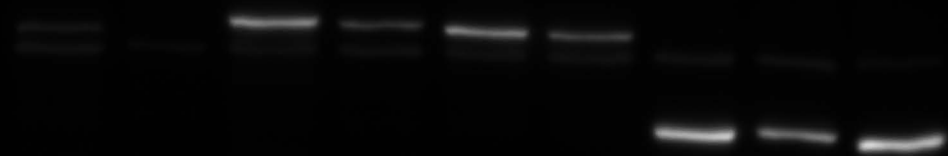

30 Res

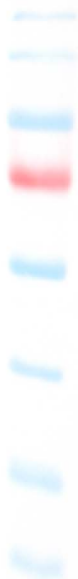

3B 12es

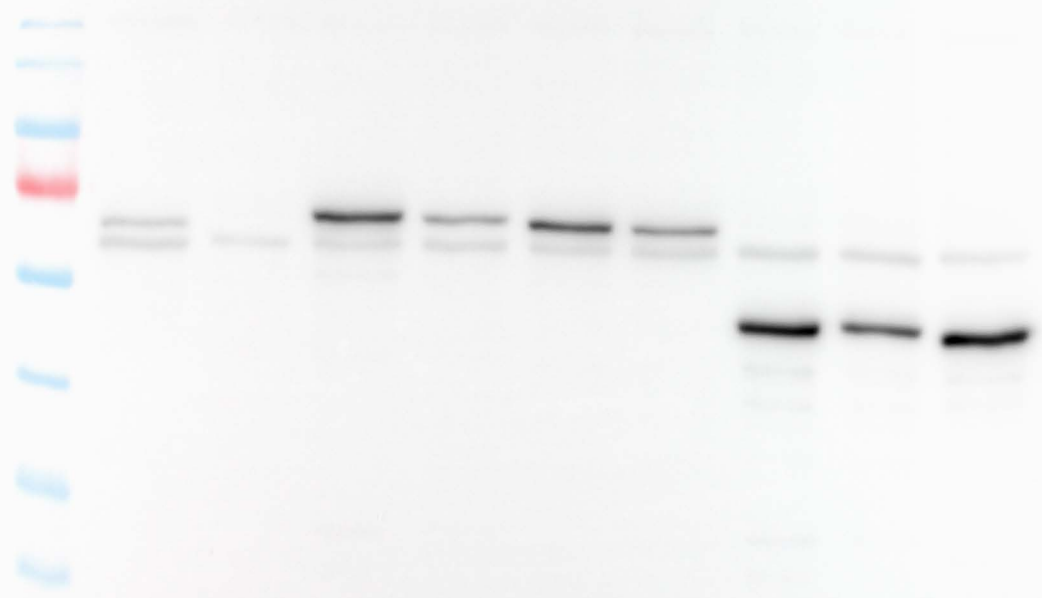

Supplement: Supplementary file 15 — Source Data for Figure 6 [file EMBJ-41-e109191-s005.zip › Fig6/Fig6F_Rescues_UPF3B.pdf]

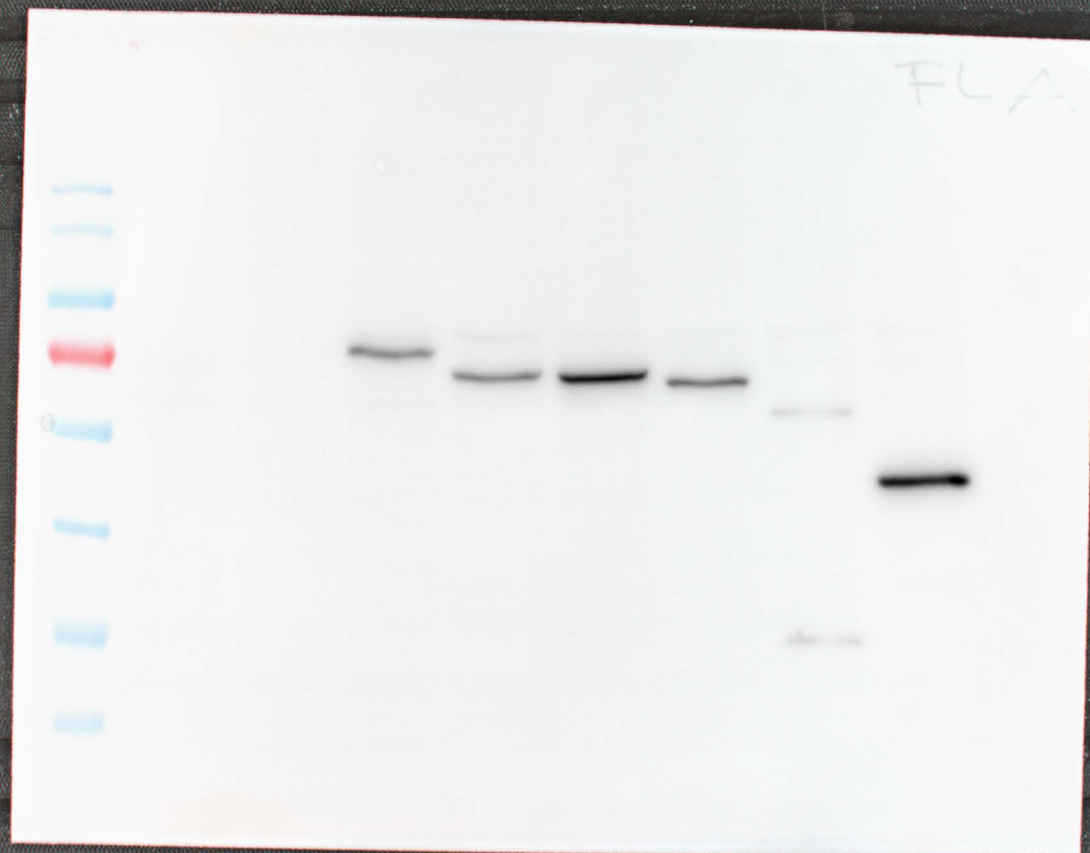

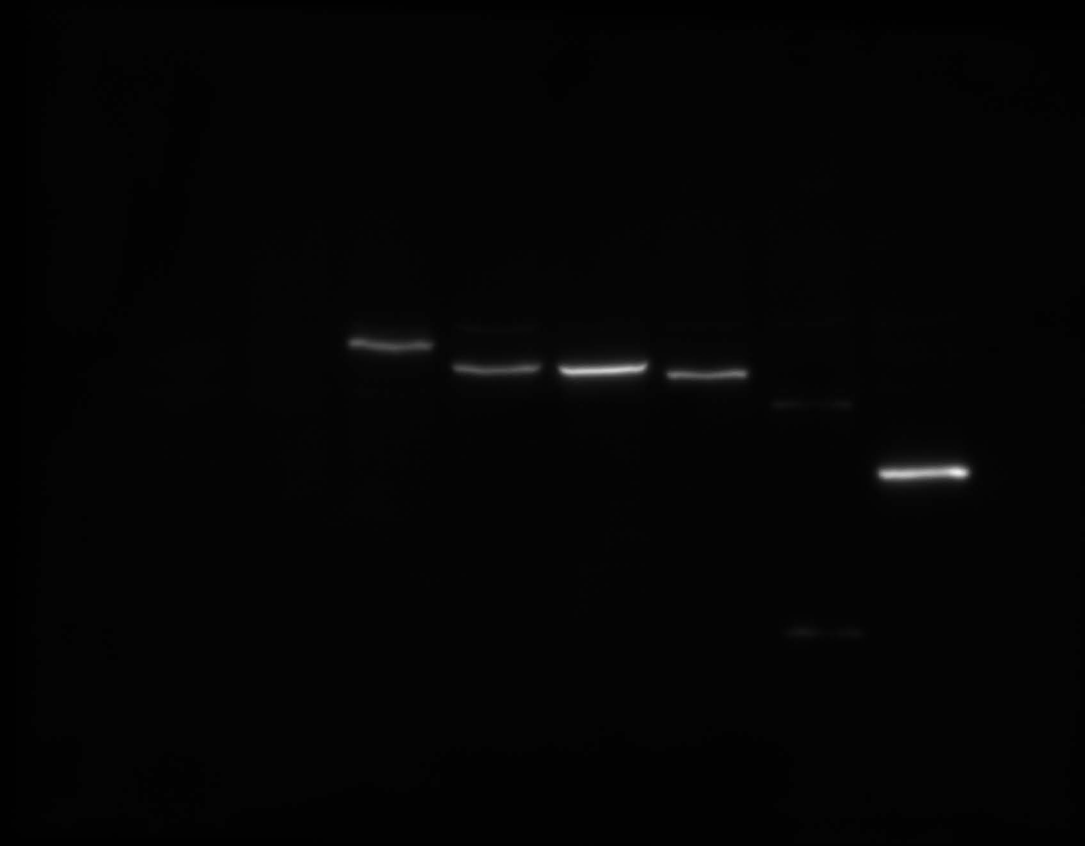

FLA

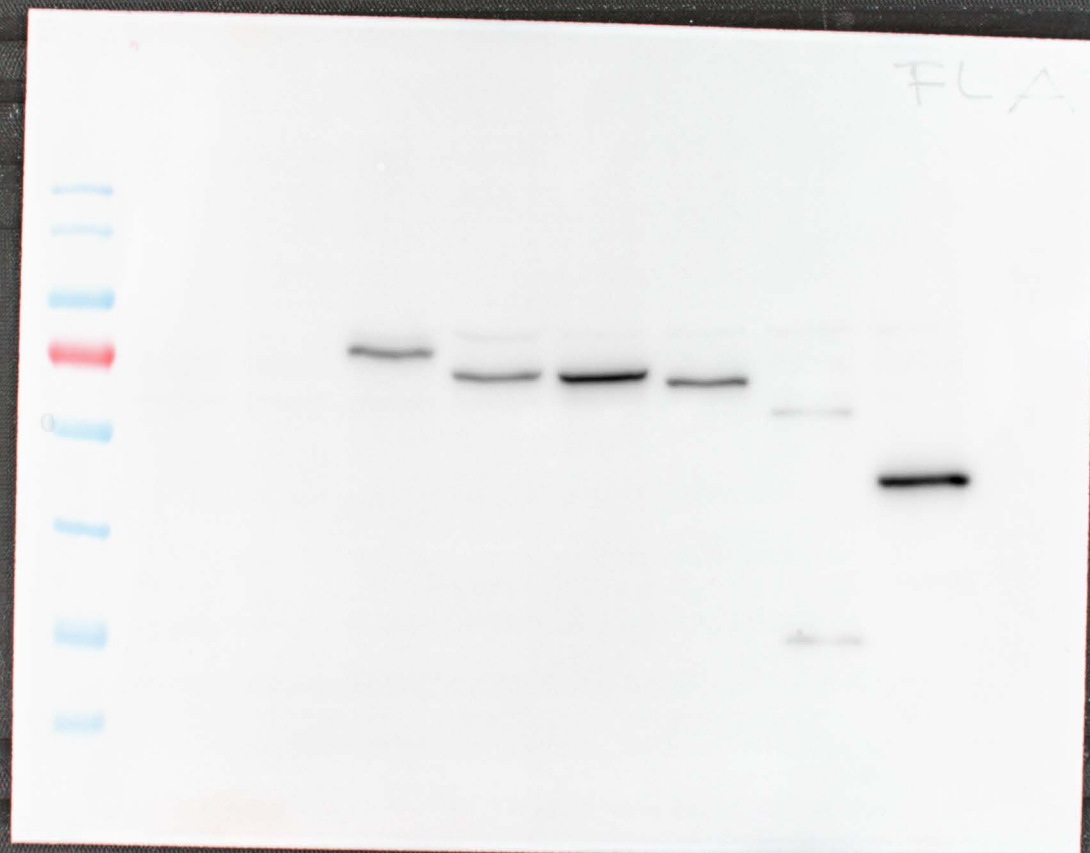

Supplement: Supplementary file 16 — Source Data for Figure 7 [file EMBJ-41-e109191-s003.zip › Fig7/Fig7B_Rescues_set2_FLAG.pdf]

FLA

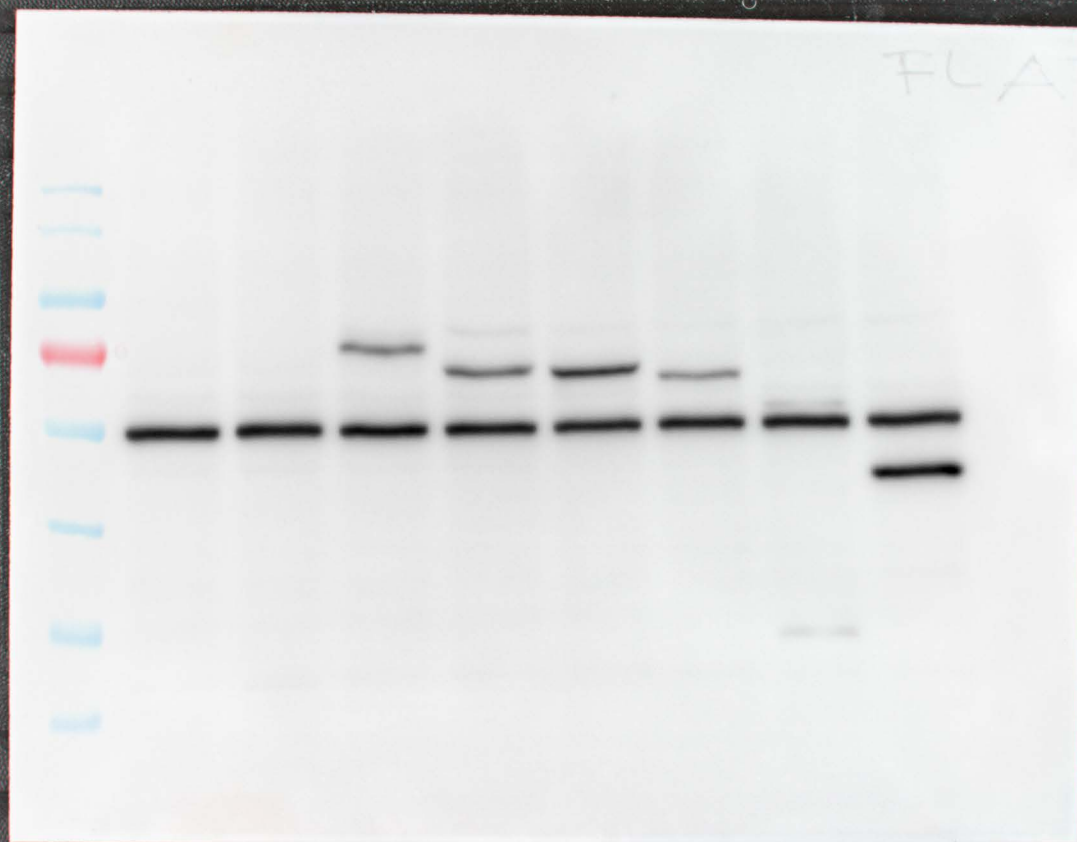

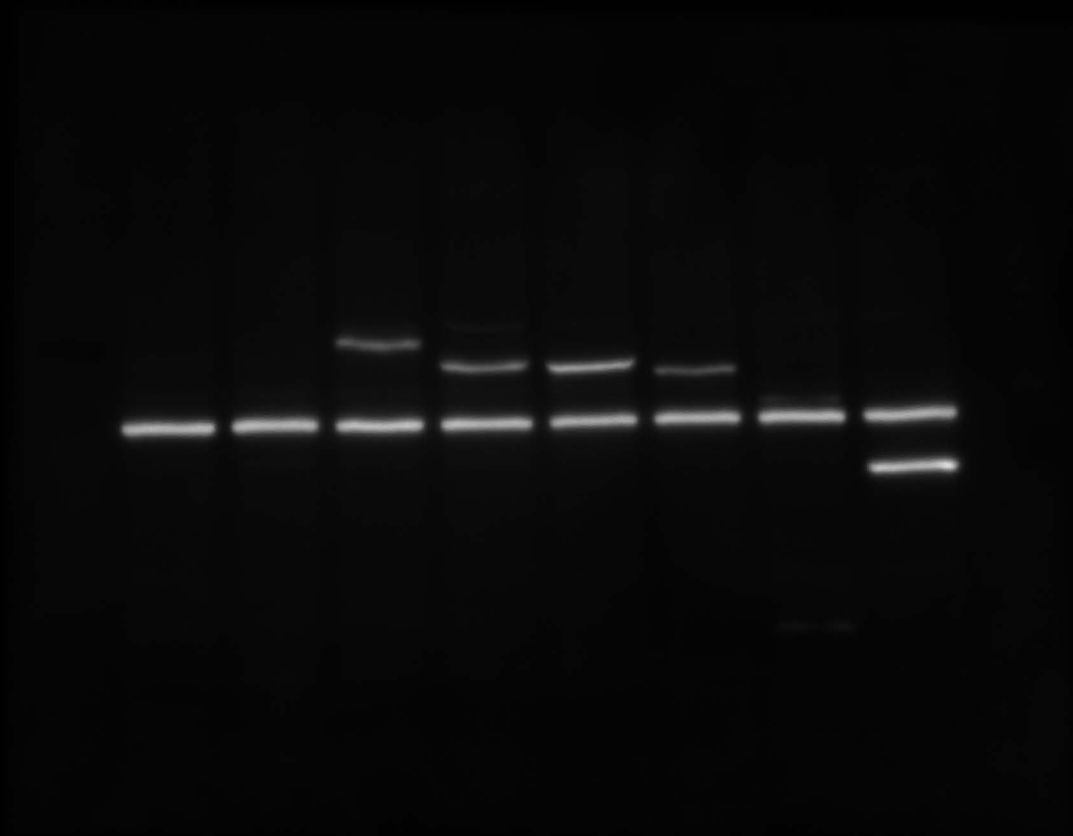

FLA

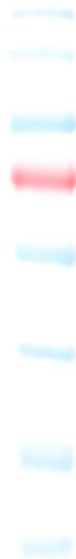

FLA

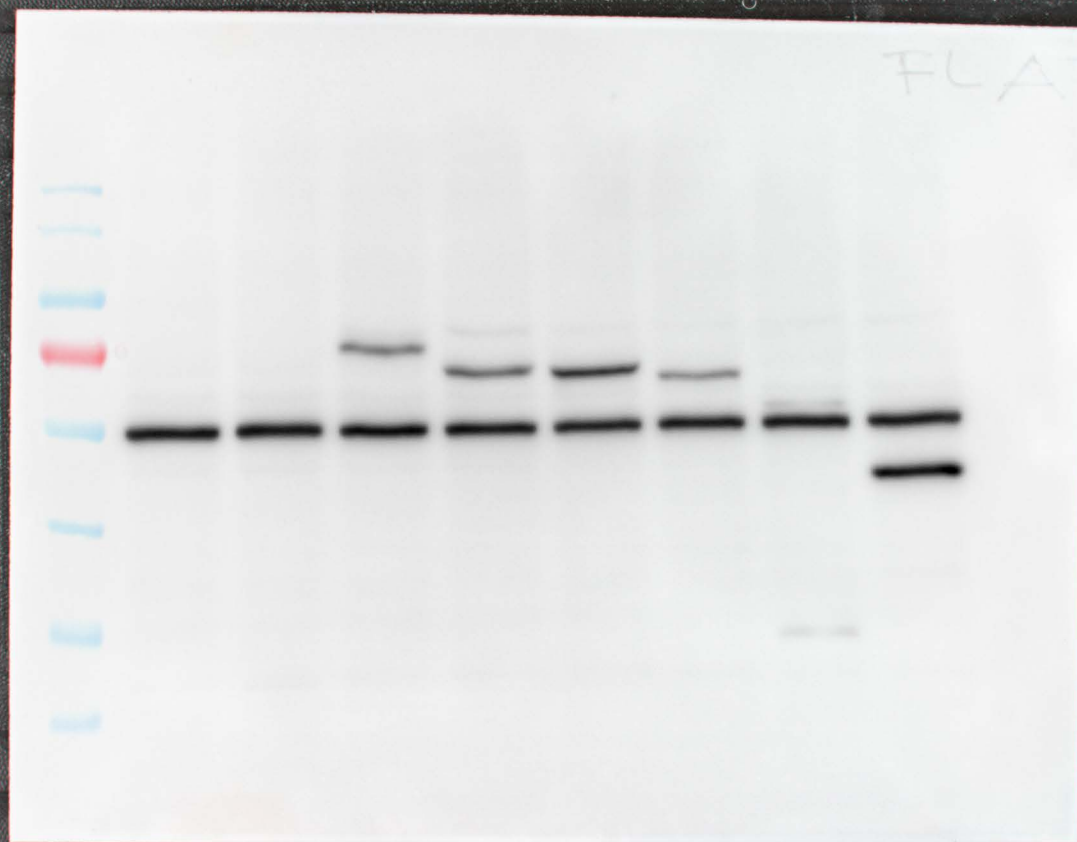

Supplement: Supplementary file 16 — Source Data for Figure 7 [file EMBJ-41-e109191-s003.zip › Fig7/Fig7B_Rescues_set2_Tubulin.pdf]

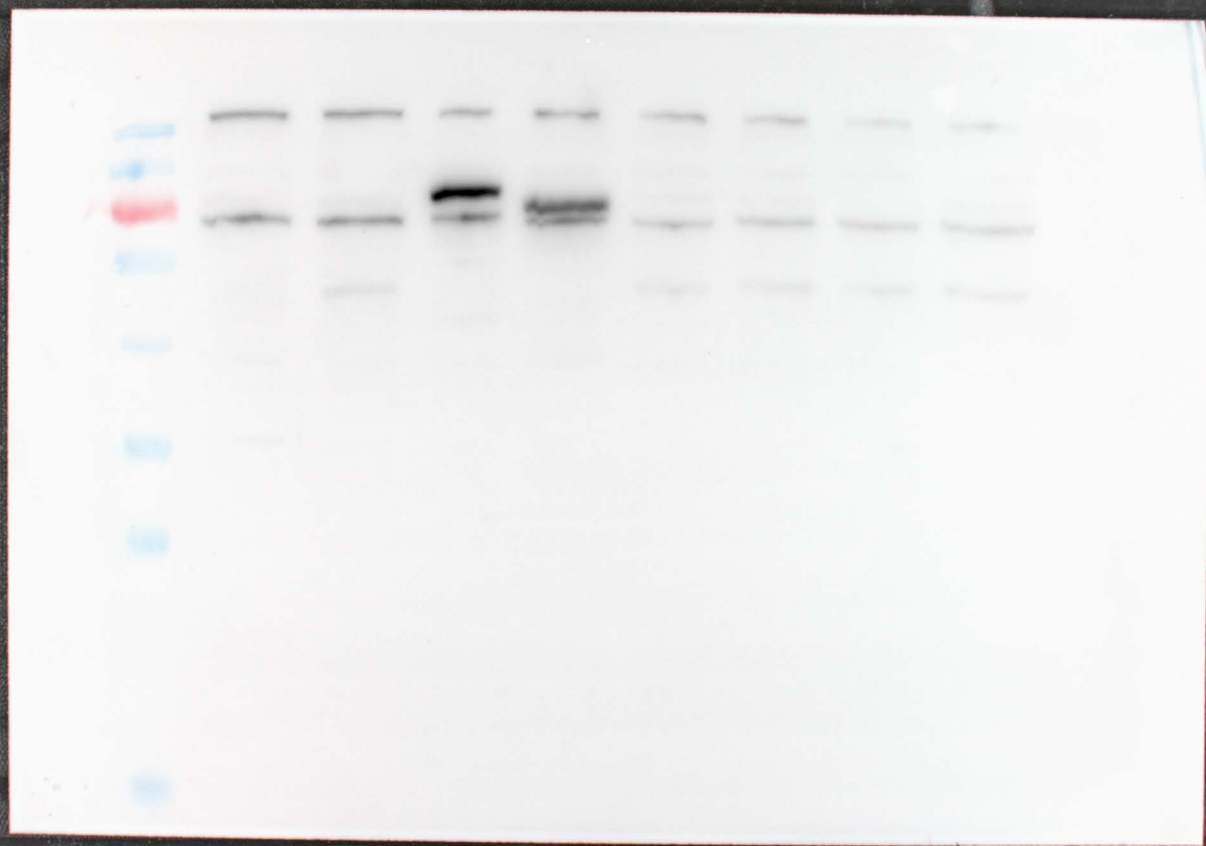



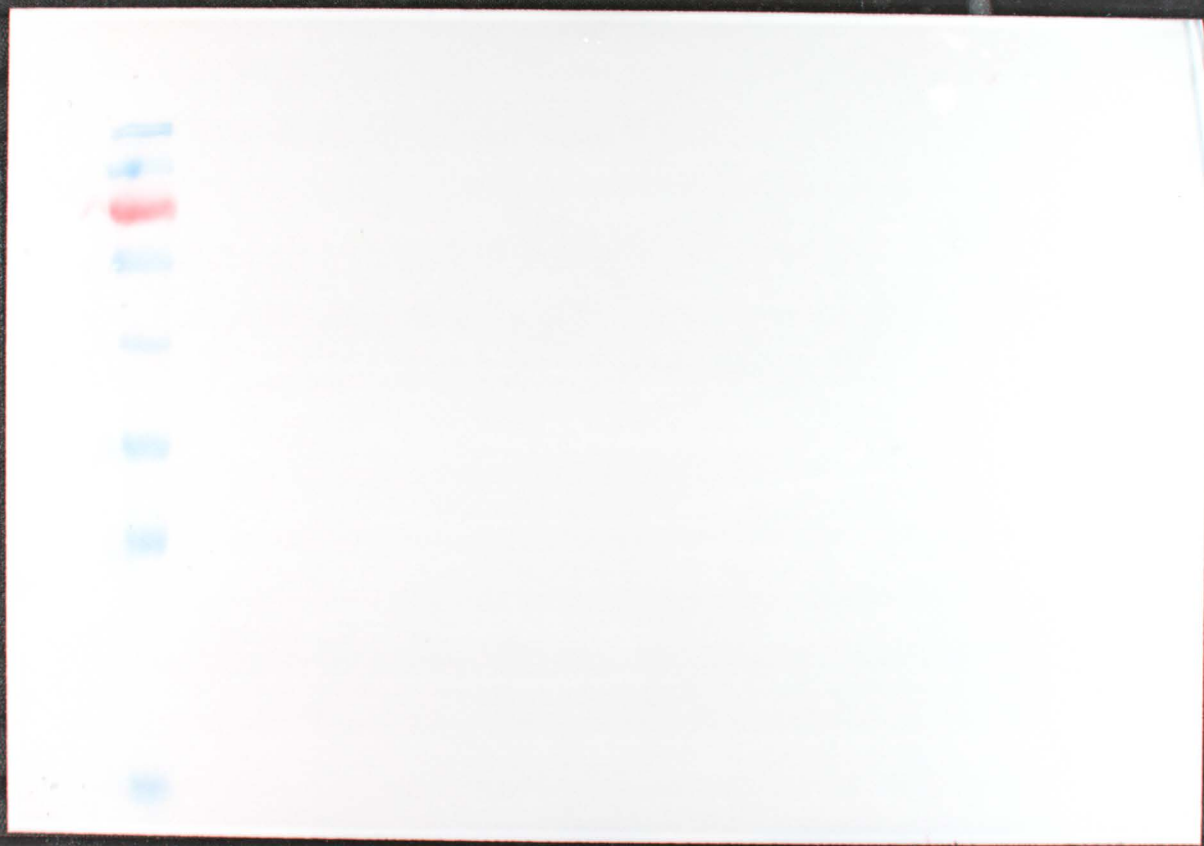

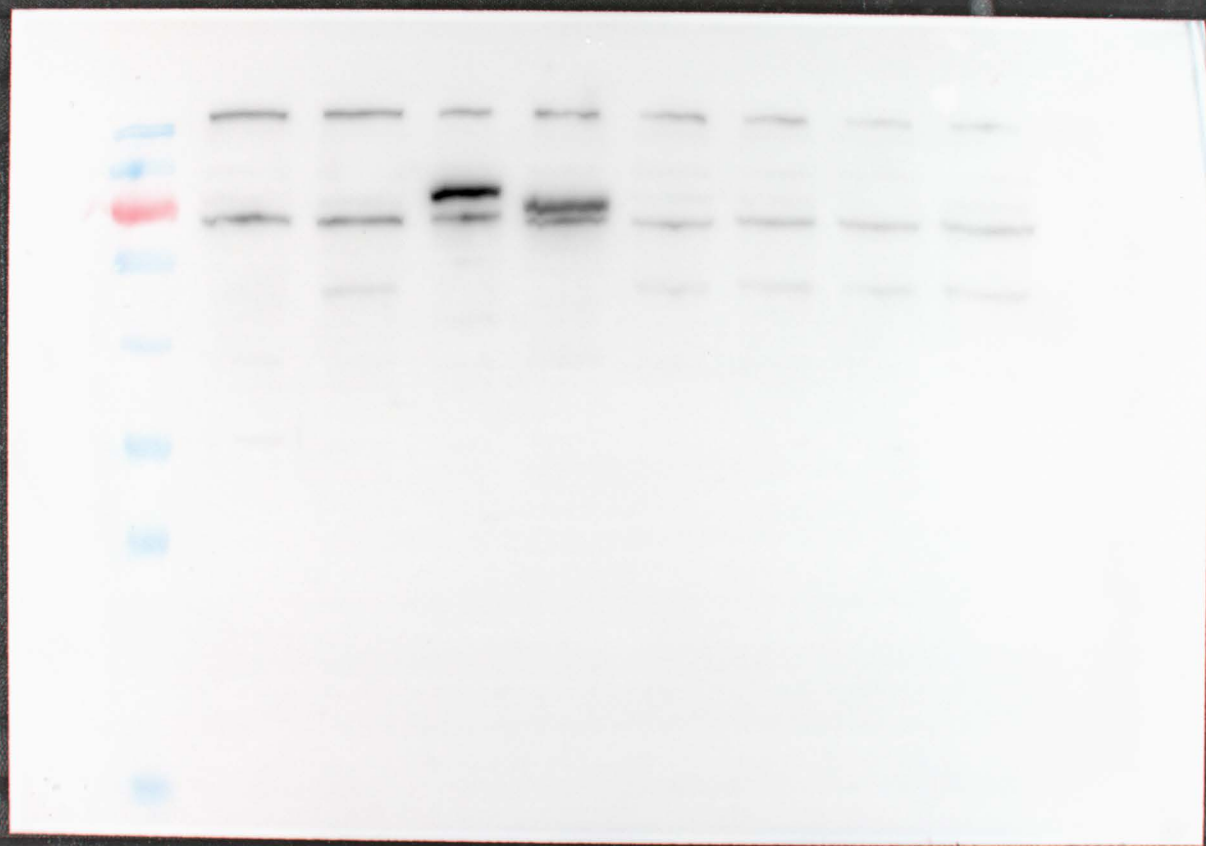

Supplement: Supplementary file 16 — Source Data for Figure 7 [file EMBJ-41-e109191-s003.zip › Fig7/Fig7B_Rescues_set2_UPF3A.pdf]

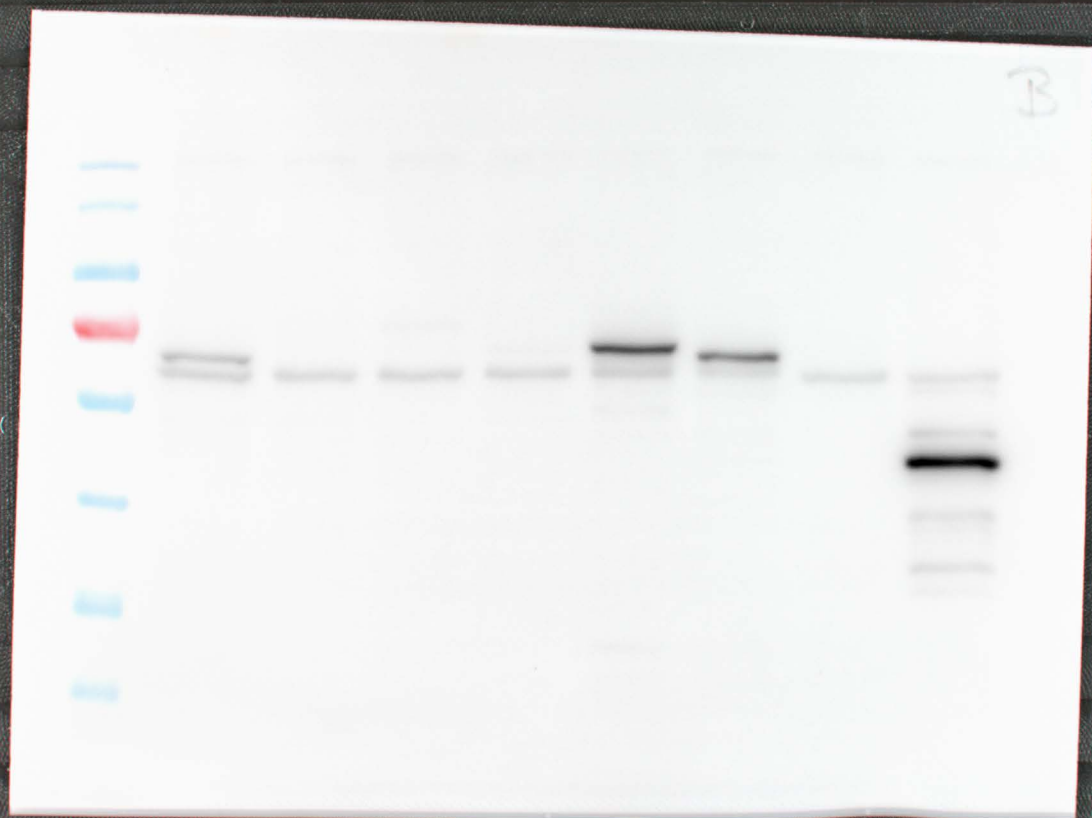

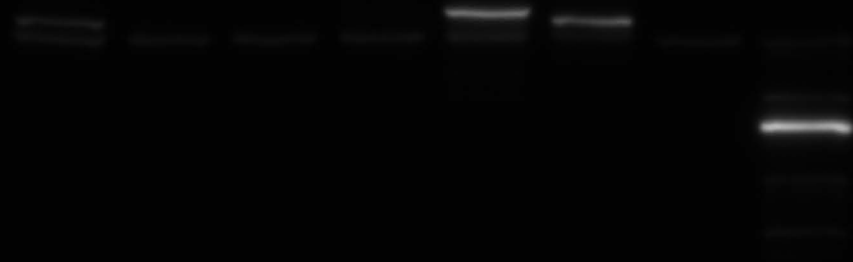

B

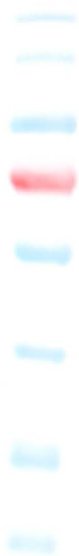

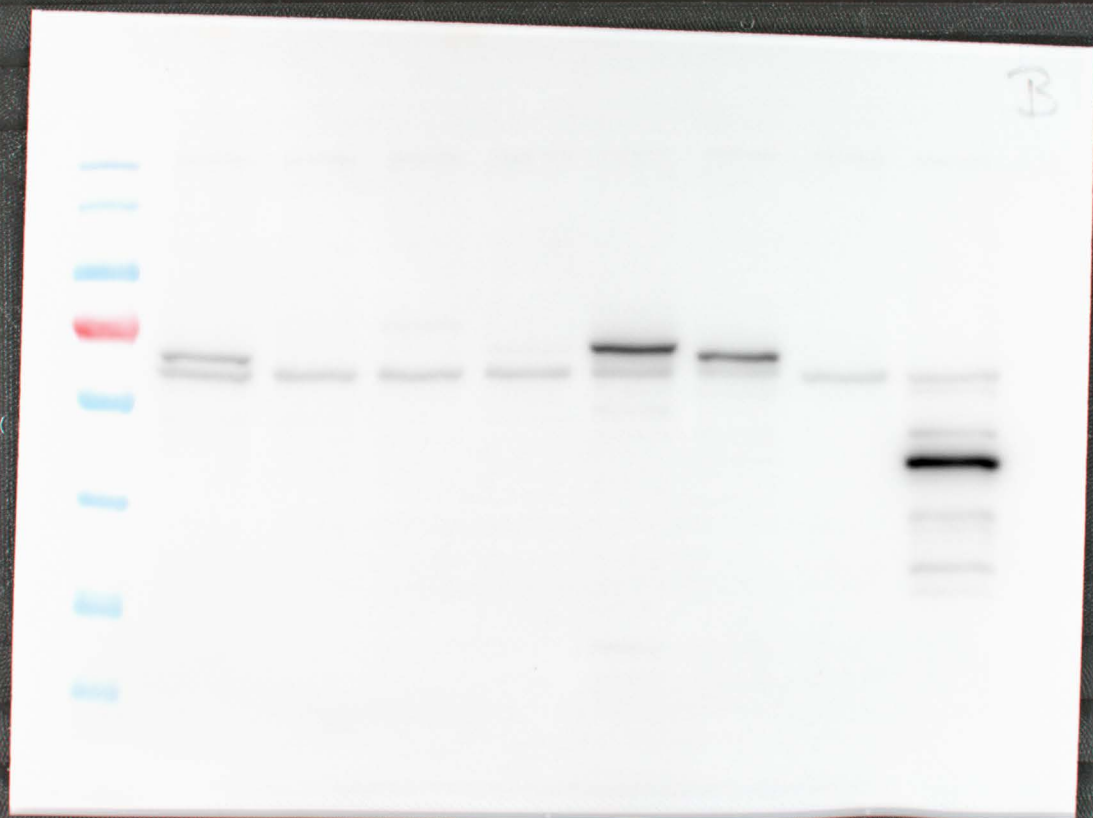

Supplement: Supplementary file 16 — Source Data for Figure 7 [file EMBJ-41-e109191-s003.zip › Fig7/Fig7B_Rescues_set2_UPF3B.pdf]
