# Supplementary material for: Human UPF3A and UPF3B enable fault‐tolerant activation of nonsense‐mediated mRNA decay
Source: EMBO J. 2022 Apr 22;41(10):e109191. doi: 10.15252/embj.2021109191 (PMC9108619; doi:10.15252/embj.2021109191)
Supplement: Supplementary file 17 [file EMBJ-41-e109191-s002.pdf]

THE EMBO JOURNAL

Human UPF3A and UPF3B enable fault-tolerant activation of nonsense-mediated mRNA decay

Damaris Wallmeroth, Jan-Wilm Lackmann, Sabrina Kueckelmann, Janine Altmüller, Christoph Dieterich, Volker Böhm, and Niels Gehring
DOI: 10.15252/embj.2021109191

Corresponding author(s): Niels Gehring (ngehring@uni-koeln.de) , Volker Böhm (boehmv@uni-koeln.de)

Review Timeline:

Submission Date:	12th Jul 21
Editorial Decision:	27th Aug 21
Revision Received:	30th Nov 21
Editorial Decision:	18th Jan 22
Revision Received:	18th Mar 22
Accepted:	31st Mar 22

Editor: Stefanie Boehm

Transaction Report:

(Note: With the exception of the correction of typographical or spelling errors that could be a source of ambiguity, letters and reports are not edited. Depending on transfer agreements, referee reports obtained elsewhere may or may not be included in this compilation. Referee reports are anonymous unless the Referee chooses to sign their reports.)

Thank you for submitting your manuscript on the function of human UPF3A and UPF3B The EMBO Journal. We have now received three referee reports on your study, which are included below for your information. In light of the referees' comments, we would like to invite you to prepare and submit a revised manuscript.

As you will see, the reviewers acknowledge the value of the reported finding to the field, but they also raise several major concerns that must be adequately addressed before the study can be considered further for publication. In particular, referee #2 and #3 stress that several conclusions are not fully supported by the provided data and are in too speculative in their opinion. It will thus be crucial to revise the manuscript to ensure that all statements are adequately supported by experimental data and discussed in the context of previous work, including revising the proposed model and its discussion (ref#2- summary, specific points- 4,5; ref#3- points 1, 2, 3, 4, 5, 6, 7, 13, 14, 15, 17, 18, 20). In addition, it will also be important to further address referee #3's point regarding the role UPF3A as an NMD repressor (point 8, 9) experimentally, as well as by revising the text and adding to the discussion. Please also carefully consider all other referee comments and revise the manuscript and figures as appropriate, as well as providing a detailed response to each comment.

Please note that it is our policy to allow only a single round of major revision. Acceptance depends on a positive outcome of a second round of review and therefore on the completeness of your responses included in the next, final version of the manuscript. We realize that lab work worldwide may currently still be affected by the COVID-19/SARS-CoV-2 pandemic and that an experimental revision can be delayed. We can extend the revision time when needed, and we have extended our 'scooping protection policy' to cover the period required for a full revision. However, it is nonetheless important to clarify any questions and concerns at this stage. Therefore, I encourage you to review the referees' comments and to contact me to discuss specific points or a preliminary revision plan in case of any uncertainties or if any additional questions regarding this revision come up.

Referee #1:

The manuscript "UPF3A and UPF3B are redundant and modular activators of nonsense-mediated mRNA decay in human cells" by Wallermoth and colleagues revisits the roles of the human UPF3 paralogues, UPF3A and UPF3B in NMD. The predominant NMD-activating paralogue was believed to be UPF3B. The established model in the NMD field propounds that UPF3B acts as a bridge between the exon-junction complex and UPF2, which in turn recruits the RNA helicase UPF1 and activates it, triggering NMD. The formation of the decay-inducing complex, a large multi-component of assembly of the eukaryotic release factors, the protein kinase SMG1 and UPF1 on one hand and the EJC-UPF3-UPF2 on the other, was thought to unequivocally commit PTC-containing mRNA transcripts to NMD. Although a lot of work has already been done towards establishing this model, the need for two UPF3 paralogues and their distinct roles has not been investigated in as much detail. UPF3A was believed to be a weak NMD activator in comparison to UPF3B, till a fairly recent study from 2016 by the Wilkinson lab suggested that UPF3A and UPF3B have opposing roles in NMD, where UPF3B is an NMD activator and UPF3A is an NMD inhibitor.

In this study, the authors set out to investigate the roles of the UPF3 paralogues using technological advances that have enabled molecular and cell biologists to revisit old models on a high throughput scale. They created UPF3 CRISPR-Cas knockout cell lines and also inducible overexpression cell lines to study the effect of depletion or abundance of UPF3A on NMD. Surprisingly, enhancement or loss of UPF3A does not appear to impact NMD, refuting the previous claim that UPF3A is an NMD inhibitor. Furthermore, depletion of UPF3B alone does not significantly impact NMD levels in cells as UPF3A is upregulated under these conditions and can mediate NMD. This is a highly significant finding as it shows that an element of redundancy is built into the NMD machinery.

The other significant finding, in my opinion, is that UPF3's role in NMD is not defined entirely by its ability to interact with the EJC or UPF2. A previous study by Neu-Yilik and colleagues reported the interaction of UPF3 with the eukaryotic release factors in inefficient translation termination, suggesting a new role of UPF3 in NMD. An even earlier study by Conti and co-workers showed that the NMD endonuclease SMG6 can be recruited to this pathway via its interaction with the EJC or UPF1 (independent of phosphorylation). These data compel us to rethink NMD is mediated and to take into consideration the many ways in which this pathway can be triggered and the numerous transient (and compensatory) interactions that take place in this pathway.

Overall, I find this a very well-designed and well-constructed study which provides plenty of food for thought for researchers in the field. I have no major criticisms or concerns.

Minor concerns:

• It would be nice if, in the discussion, the authors elaborated on the numerous transient interactions that have been identified in NMD so far, so as to dispel the notion of "stable" complex formation (such as SURF, DECID, EJC-UPF3-UPF2-UPF1) being essential for NMD. This would be a great service to the field.
• I always find it nice to see a gel/western blot confirming proteomics/IP-MS data, but I acknowledge that this, perhaps, is no longer necessary.

Referee #2:

UPF3A and UPF3B are redundant and modular activators of nonsense-mediated mRNA decay in human cells

In this manuscript, Wallmeroth et al. evaluate the role of UPF3 paralogs, UPF3A and UPF3B, in targeting of mRNA to nonsense-mediated decay (NMD). Early studies demonstrated UPF3B as being expressed at higher levels in mammalian cells and playing the major role in eliciting NMD presumably through its binding to EJC complexes and mediating interactions with UPF2, UPF1 and the terminating ribosome; more recent reports, however, have suggested UPF3A can act as a NMD repressor and antagonize NMD as a consequence of its reduced binding to EJCs. To resolve these data, the authors performed RNA-Seq on HEK293 cells either stably over-expressing FLAG-tagged UPF3A or cells in which UPF3A was knocked out and found no significant or reproducible changes in gene expression under either condition. These results contradict previous findings in HeLa cells showing that over-expression of UFP3A inhibits NMD in human cells, and, in contrast, suggest that UPF3A is dispensable for NMD (in the presence of UPF3B). Notably, KO of UPF3B, the purported major UPF3 factor in mammals also failed to lead to significant changes in gene expression or up-regulation of known NMD targets. In contrast, siRNA-mediated depletion of UPF3A in UPF3B KO cells caused significant up-regulation of NMD targets, supporting the conclusion that UPF3A and 3B are redundant and that UPF3A can compensate for loss of UPF3B in promoting NMD in these cells. Analysis of UFP3A/3B double KO cells confirmed these results and identified over 1000 transcript unregulated in these cells, 890 of which were previously characterized NMD substrates.

To determine whether UPF3A and 3B function similarly in interacting with EJC proteins and promoting interactions with the NMD machinery, FLAG-tagged UPF2 was immunoprecipitated from cells and interacting proteins identified through mass spectrometry. In wild type cells, UPF2 was shown to co-purify with both UPF3B and 3A, various EJC proteins and additional NMD components; however, interactions with EJC components (other than CASC3) were severely reduced in the absence of UPF3B and further eliminated in cells lacking both UPF3A and 3B. These data suggest that while UFP3A is able to functionally compensate for UPF3B in mediating NMD in UPF3B KO cells, its role may be independent of bridging an interaction between UPF2 and the EJC. Finally, using complementation assays in UPF3B KO/UPF3A KD cells, alleles of UPF3B unable to bind either UPF2 or EJC were found to be competent for NMD (but not mutants lacking both activities), suggesting these individual interactions are not required for UPF3B function in NMD.

Sufficient data is presented to support the conclusion that UPF3A does not act to repress NMD in HEK293 cells and that UPF3A and UPF3B have redundant functions in promoting NMD in these cells. In addition, the data leads to a hypothesis by which UPF3A or 3B can function in NMD independent of an interaction with EJC proteins and, in the case of UPF3B, an interaction with UPF2; however, this model would be better supported by experimental data demonstrating loss of the alleged protein interactions in this system. The presented model for UPF3 function in NMD is highly speculative and not experimentally tested or resolved from this present work.

Specific concerns:

UPF3B mutant alleles purported to disrupt interactions with UPF2 or EJC, are critical reagents in this study and need to be experimentally validated (by coIP, for example) in this cell system. Additionally, these mutants should be further evaluated using the robust PTC-containing β-globin reporter assay shown in Fig. 4E.

The authors should provide longer exposures of western blots presented in Fig. 1B, 2A, and 3A to enable clear evaluation of UPF3A levels in over-expression and knock-down cell lines. Additionally, the authors should comment on the large differences in gene expression changes observed for the different UPF3A KO clones.

It is unclear why the UPF3A/B double KO cells see additional changes in gene expression upon additional siRNA-mediated depletion of UPF3B. The authors indicate that UPF3B is still present in the double 'knockout' cells, but if true, then it is inaccurate to refer to these cell lines as knock-outs.

The model in Fig 1A is confusing and unnecessary. Additionally, the model for UPF3 function in NMD presented in Fig. 8 is highly speculative and not derived from data gleaned from the present study and is misleading to the reader (note that the bottom panel of 8C is particularly difficult to follow).

There is a general paucity of direct presentation and quantitative discussion of the data and in many instances experimental findings are over-sold. For example, it is premature to refer to UPF3A and UPF3B as either 'modular activators' of NMD (title) or 'fault-tolerant NMD activators' (abstract).

Referee #3:

Wallmeroth et al. provide an intriguing study on 2 key factors in the NMD RNA turnover pathway: UPF3A and UPF3B (which I

will refer to as 3A and 3B, respectively). There is widespread interest in 3A and 3B because of their association with human disease (e.g., 3B mutations cause human intellectual disability) and the evidence implicating these two factor as key regulators/factors of the NMD pathway.

Using the immortalized human embryonic kidney cell line, HEK293, for most of their studies, the authors conduct a wide range of interesting experiments to address questions about 3A/3B. They provide evidence for the following main points: First, they show that 3B has a role but is not essential for NMD in HEK293 cells, thereby verifying past reports studying other cell types in vitro and tissues in vivo. Second, they show that 3A and 3B are partially functionally redundant at a genome-wide level, which verifies results from past studies on specific NMD target mRNAs. Third, they provide evidence that 3A does not act as a global NMD repressor in HEK293 cells, as suggested by an earlier study in several other cell types. Fourth, they provide exciting evidence that 3B does not act in NMD by the molecular mechanism previously widely accepted by the field. In particular, previous evidence had strongly suggested that 3B is a key tethering molecule that bridges the NMD factor, UPF2, with the exon-junction complex (EJC), the latter of which is a "NMD amplifier" complex bound on RNA near exon-exon junctions after RNA splicing. The authors' indicates that this "tethering model" is incorrect, which is a significant advance for the field. Fifth, they identify a novel central domain in 3B that functions in NMD, leading to a new model as to how 3B functions. Sixth, the authors demonstrated that a naturally occurring 3A isoform that lacks exon 4 (which partially overlaps with the new middle domain) lacks the ability to support NMD. Deletion of this region in 3B has the same effect. Collectively, their results from mutating different domains in 3A and 3B support an exciting model in which these 2 factors drives NMD by "exert(ing) multiple functions at different time points of NMD and in association with different complexes."

This is a focused and well-written MS that provides key information for the RNA field, as well as for those trying to understanding human diseases caused by defects in 3A/3B. The main problem with this MS revolves around the feeling that the authors want to maximize impact by drawing sweeping conclusions from sometimes limited (and perhaps biased) data analysis. In other words, the authors sometimes do not seem to acknowledge that biology is often "messy" and thus nature typically does not conform to simplistic conclusions. That said, the experiments conducted in this report are generally top notch, so with more in-depth data analysis, coupled with revised writing, this will likely become an outstanding paper of considerable interest.

(1) Abstract. The phrase "contradicting reports" is incorrect, as different approaches were used. Early studies used the tethering approach to demonstrate that 3A is weak NMD factor, whereas the most recent study knocked down and over-expressed 3A to provide evidence it is both a NMD factor and a NMD repressor. It is suggested to instead say something like: "...past reports have provided evidence that UPF3A is both a NMD repressor and activator."
(2) The authors often use the term "UPF3" instead of referring specifically to either the UPF3A or UPF3B paralog, or both. This should be rectified by referring to one or both (unless the context dictates the use of the general term). Examples where the term "UPF3" is inappropriately used are lines 36, 92, 95, 302, 310, 429, and 432.
(3) The concept that NMD is partially functional in the absence of 3B is not new; several studies have demonstrated this previously, which should be acknowledged in the Introduction or Results.
(4) The concept that 3A and 3B are partially functionally redundant is also not new; past studies should be cited in the Introduction or Results, and the new results from the authors put in this context.
(5) Shum et al. The description of Shum et al. in the Introduction (line 121) and Discussion (line 371) fails to mention a key point: this paper provided evidence that 3A is not only a NMD repressor but also a NMD activator (its activity differs depending on biological context and transcript). This is an important point with regard to some of the studies in this MS (see below).
(6) Line 125: they authors claimed to have "resolved the controversy about the functions of UPF3A and UPF3B..." In my opinion, the authors have provided important information on the roles of 3A and 3B in NMD, but they have not reconciled all past studies on this topic.
(7) Results section titles. While most of the section titles are nicely short and to the point, most make definitive statements that require some modifications. For example, the first section title - "UPF3A overexpression or knockout does not affect NMD efficiency" - does not mention that this conclusion is based only on analysis in HEK293 cells. Furthermore, as described below in point 8, the authors have evidence that 3A DOES affect NMD in HEK293 cells, and further analysis may reveal that this is more extensive than currently described by the authors. The remaining section titles also do not indicate that their conclusions are based on studies in HEK293 cells. These titles need to be qualified or softened.
(8) Evidence that 3A a NMD repressor. The authors make the claim that 3A is not a broadly-acting NMD repressor in immortalized HEK293 cells. While this is supported by the RNAseq data the authors show, they do not make much effort to determine whether 3A has ANY NMD repressor activity (e.g., that acts on specific NMD target mRNAs) in HEK293 cells. This is important, as Shum et al. 2016 previously provided evidence that 3A is a NMD repressor in several other cells types; e.g., pluripotent cells and normal fibroblasts in vitro, and normal germ cells and olfactory cells in vivo. Furthermore, at least 3 lines of evidence from the authors of the present MS support that 3A might be a NMD repressor: First, they found that more mRNAs were downregulated than upregulated in response to 3A knockout (ko) (Fig. 2F). Second, Fig. EV2G shows that more PTC+ transcripts are downregulated than upregulated in response to 3B loss in one 3B-ko cell clone (while this was not observed in the other clone they generated, this at least indicates uncertainty as to 3A's role). Third, 2 of the 3 specific mRNAs the authors analyzed by qPCR were downregulated in response to 3B ko (Fig. 2E). The authors reported that the mRNA downregulated in both clones, ZFAS1, was not upregulated by 3A rescue, but it is not clear to what degree 3A was force expressed, as its level was not compared with endogenous 3A in control cells. Was it insufficiently expressed? Was it overexpressed and thus not physiological? This issue is critical to assess, not only for this experiment, but for other experiments in which 3A was force expressed. The authors should also explain why these 3 particular NMD target mRNAs were chosen to be examined. Finally, to

evaluate 3A in a more rigorous fashion, it is strongly suggested to perform qPCR analysis on a larger set of NMD target mRNAs chosen in an unbiased (but justified) fashion.
(9) Examining the extent of which 3A is a NMD repressor. The authors should examine the extent at which 3A is a NMD repressor in HEK293 cells by using their existing RNAseq datasets. First, the authors should examine the nature of the PTC+ transcripts that are downregulated in 3B-ko cells and compare them with unregulated and upregulated PTC+ transcripts. Perhaps there are specific cis element or other sequence features (e.g., GC content) that correlates with NMD being repressed by 3A. Second, the authors should look for overlap between the many high-confidence human NMD target mRNAs identified by other studies (e.g., based on RIPseq, RNA half-life, or pUPF1 occupancy) and mRNAs downregulated in 3B-ko cells identified by the authors. This would establish NMD target mRNAs repressed by 3A in HEK293 cells. This is also important, as not all "PTC+ transcripts" defined by the authors' current analysis are necessarily direct NMD targets; e.g., NMD silencer elements have been defined that allow NMD escape, and alternative processing events can obscure interpretation. Conversely, many of the transcripts defined as "PTC-" ARE NMD targets; e.g., many mRNAs with long 3'UTRs are degraded by NMD. Third, the authors should evaluate whether 3A might have predilection for repressing the decay of transcripts with NMD-inducing features besides an exon-exon junction downstream of a stop codon, such as a long 3' UTR or an upstream ORF. The bottom line is the authors should analyze their 3A ko and control RNAseq datasets more carefully to more definitively assess whether 3A is a NMD repressor in HEK293 cells, and, if so, to what extent, and whether it has some kind of selectivity.
(10) Line 186. Where is the DTU analysis of 3A-ko cells?
(11) The authors tested whether 3A forced expression would inhibit NMD in the context of 3B ko (Fig. EV3H & I). They claim no negative effects, but only 3 NMD targets were selected (without any justification why these 3 were picked). Of note, SRSF2 showed a trend towards being downregulated, which was rescued by 3A.
(12) A potential concern with the 3A- and/or 3B-ko clones generated by the authors is these cells might compensate for this loss over time (since these cell clones would have undergone many, many cell doublings, whereas transient knockdown [kd] cells would not). This compensation might eliminate some of the regulation that normally occurs. This caveat should be mentioned.
(13) 3A functions independently of being a bridging factor. The authors indicated on line 290 that it was SURPRISING that the ability of UPF2 to associate with the EJC (an event considered critical to activate NMD) was not compensated for by 3A in 3B-ko cells. It is not clear why the authors considered this surprising, as it was previously established that 3A has a poorly conserved EJC-interaction domain and 3A was empirically shown to have very weak interactions with the EJC. It is suggested that the authors rephrase.
(14) Lines 118 and 368. To my knowledge, the evidence from Nguyen et al. 2012 that 3A compensates for 3B is based on a comparison between 2 patients. This should either be acknowledged or this point be omitted.
(15) Discussion paragraph 3. First sentence (line 379): the phrase "more or less definite answers" sounds contradictory. It is suggested to omit this sentence.
(16) The third sentence of Discussion paragraph 3 starts with "First," but there is no "Second," etc. later in the paragraph.
(17) On line 384, the authors admit that the cells they used - HEK293 - may be unusual in NOT depending on 3B for NMD, but they fail to provide the evidence for this. In fact, many studies have shown that many transcripts are downregulated by 3B in other cell types (as also inferred by point 3, above). These papers and the types of cells they examined should be mentioned.
(18) Line 397. It is stated that "mouse UPF3A does not appear to be a general NMD inhibitor." While this is currently supported by the authors' evidence, the way it is written gives the impression (particularly in the context of earlier parts of this MS) that it was previously shown that 3A is only a NMD inhibitor. This is not the case, as Shum et al. 2016 provided evidence that 3A is also a NMD activator, as indicated in point 5, above. Thus, it is suggested to change the wording on line 397 to say something like: "...verify that mouse UPF3A is not a general NMD inhibitor (Shum et al. 2016).
(19) Line 403. It is suggested to revise to something such as: "As expected,...."
(20) Line 416. This model supported by the authors' data is quite intriguing. It is suggested to start a paragraph with this model and then briefly discuss the evidence in support of and against it, as well as brief future directions.
(21) Discussion point? Gene duplication is an extremely common event during evolution, but only rarely does the duplicated gene copy persist, as there needs to be sufficient selection pressure to allow it to be fixed in the population. UPF3 duplicated at the dawn of the vertebrate lineage (approximately 500 million years ago) and both copies (3A and 3B) still remain today in virtually all vertebrates. It is not surprising that 3B has persisted, as it is a well-established NMD factor. But what about 3A? It is highly unlikely that it has persisted because it serves as a back-up factor for 3B. While there is little doubt (based on the authors' evidence) that this is ONE function of 3A, it is hard to imagine how this back-up function was selected for, as evolution does not anticipate potentially useful functions; instead it acts on functions that are of value in the present. Thus, presumably 3A has another function that has allowed it to persist for 500 million years. The ability to negatively regulate NMD (act as a volume control) is a good candidate to be such a function. A natural situation in which 3B and 3A levels are both highly regulated is male germ cells as they progress through meiosis (Shum et al. 2016).

General response to the reviewers

We very much appreciate the interest of all three reviewers in our work and are grateful for their thorough evaluation of the manuscript and for their constructive criticisms. In the revised version we have addressed their comments by modifying the text and by including new experimental data as described in this detailed point-by-point response. Text changes made to the manuscript during the revision are colored in blue.

The highlights of the revised manuscript include:

- Validation of major findings in HeLa cells.
- New interaction data generated by TurboID proximity labelling and mass spectrometry.
- Substantially revised introduction, discussion and model (Figure 8).

We also analyzed our data in detail with respect to an NMD-inhibiting function of UPF3A (see response to reviewer 3).

Referee #1:

The manuscript "UPF3A and UPF3B are redundant and modular activators of nonsense-mediated mRNA decay in human cells" by Wallermoth and colleagues revisits the roles of the human UPF3 paralogues, UPF3A and UPF3B in NMD. The predominant NMD-activating paralogue was believed to be UPF3B. The established model in the NMD field propounds that UPF3B acts as a bridge between the exon-junction complex and UPF2, which in turn recruits the RNA helicase UPF1 and activates it, triggering NMD. The formation of the decay-inducing complex, a large multi-component of assembly of the eukaryotic release factors, the protein kinase SMG1 and UPF1 on one hand and the EJC-UPF3-UPF2 on the other, was thought to unequivocally commit PTC-containing mRNA transcripts to NMD. Although a lot of work has already been done towards establishing this model, the need for two UPF3 paralogues and their distinct roles has not been investigated in as much detail. UPF3A was believed to be a weak NMD activator in comparison to UPF3B, till a fairly recent study from 2016 by the Wilkinson lab suggested that UPF3A and UPF3B have opposing roles in NMD, where UPF3B is an NMD activator and UPF3A is an NMD inhibitor.

In this study, the authors set out to investigate the roles of the UPF3 paralogues using technological advances that have enabled molecular and cell biologists to revisit old models on a high throughput scale. They created UPF3 CRISPR-Cas knockout cell lines and also inducible overexpression cell lines to study the effect of depletion or abundance of UPF3A on NMD. Surprisingly, enhancement or loss of UPF3A does not appear to impact NMD, refuting the previous claim that UPF3A is an NMD inhibitor. Furthermore, depletion of UPF3B alone does not significantly impact NMD levels in cells as UPF3A is upregulated under these conditions and can mediate NMD. This is a highly significant finding as it shows that an element of redundancy is built into the NMD machinery.

The other significant finding, in my opinion, is that UPF3's role in NMD is not defined entirely by its ability to interact with the EJC or UPF2. A previous study by Neu-Yilik and colleagues reported the interaction of UPF3 with the eukaryotic release factors in inefficient translation termination, suggesting a new role of UPF3 in NMD. An even earlier study by Conti and co-workers showed that the NMD endonuclease SMG6 can be recruited to this pathway via its interaction with the EJC or UPF1 (independent of phosphorylation). These data compel us to rethink NMD is mediated and to take into consideration the many ways in which this pathway can be triggered and the numerous transient (and compensatory) interactions that take place in this pathway.

Overall, I find this a very well-designed and well-constructed study which provides plenty of food for thought for researchers in the field. I have no major criticisms or concerns.

We thank the referee for the positive evaluation of our work.

Minor concerns:

• It would be nice if, in the discussion, the authors elaborated on the numerous transient interactions that have been identified in NMD so far, so as to dispel the notion of "stable" complex formation (such as SURF, DECID, EJC-UPF3-UPF2-UPF1) being essential for NMD. This would be a great service to the field.

We would like to thank the reviewer for this suggestion. We have now added a paragraph to the discussion, in which we address the importance of transient interactions and the dispensability of stable complexes. For reasons of space and to maintain the focus of the manuscript, we have referred primarily to UPF3 in this context.

• I always find it nice to see a gel/western blot confirming proteomics/IP-MS data, but I acknowledge that this, perhaps, is no longer necessary.

We understand that validating the MS data with a western blot can increase visual understanding. After the revision, this manuscript now contains two different high-throughput interactome analyses (UPF2, UPF3B) as well as a validation of the UPF3B mutants by Western blot. We hope that this provides a good mix of different experimental approaches.

Referee #2:

UPF3A and UPF3B are redundant and modular activators of nonsense-mediated mRNA decay in human cells

In this manuscript, Wallmeroth et al. evaluate the role of UPF3 paralogs, UPF3A and UPF3B, in targeting of mRNA to nonsense-mediated decay (NMD). Early studies demonstrated UPF3B as being expressed at higher levels in mammalian cells and playing the major role in eliciting NMD presumably through its binding to EJC complexes and mediating interactions with UPF2, UPF1 and the terminating ribosome; more recent reports, however, have suggested UPF3A can act as a NMD repressor and antagonize NMD as a consequence of its reduced binding to EJCs. To resolve these data, the authors performed RNA-Seq on HEK293 cells either stably over-expressing FLAG-tagged UPF3A or cells in which UPF3A was knocked out and found no significant or reproducible changes in gene expression under either condition. These results contradict previous findings in HeLa cells showing that over-expression of UFP3A inhibits NMD in human cells, and, in contrast, suggest that UPF3A is dispensable for NMD (in the presence of UPF3B). Notably, KO of UPF3B, the purported major UPF3 factor in mammals also failed to lead to significant changes in gene expression or up-regulation of known NMD targets. In contrast, siRNA-mediated depletion of UPF3A in UPF3B KO cells caused significant up-regulation of NMD targets, supporting the conclusion that UPF3A and 3B are redundant and that UPF3A can compensate for loss of UPF3B in promoting NMD in these cells. Analysis of UFP3A/3B double KO cells confirmed these results and identified over 1000 transcript unregulated in these cells, 890 of which were previously characterized NMD substrates.

To determine whether UPF3A and 3B function similarly in interacting with EJC proteins and promoting interactions with the NMD machinery, FLAG-tagged UPF2 was immunoprecipitated from cells and interacting proteins identified through mass spectrometry. In wild type cells, UPF2 was shown to co-purify with both UPF3B and 3A, various EJC proteins and additional NMD components; however, interactions with EJC components (other than CASC3) were severely reduced in the absence of UPF3B and further eliminated in cells lacking both UPF3A and 3B. These data suggest that while UFP3A is able to functionally compensate for UPF3B in mediating NMD in UPF3B KO cells, its role may be independent of bridging an interaction between UPF2 and the EJC. Finally, using complementation assays in UPF3B KO/UPF3A KD cells, alleles of UPF3B unable to bind either UPF2 or EJC were found to be competent for NMD (but not mutants lacking both activities), suggesting these individual interactions are not required for UPF3B function in NMD.

Sufficient data is presented to support the conclusion that UPF3A does not act to repress NMD in HEK293 cells and that UPF3A and UPF3B have redundant functions in promoting NMD in these cells. In addition, the data leads to a hypothesis by which UPF3A or 3B can function in NMD independent of an interaction with EJC proteins and, in the case of UPF3B, an interaction with UPF2; however, this model would be better supported by experimental data demonstrating loss of the alleged protein interactions in this system. The presented model for UPF3 function in NMD is highly speculative and not experimentally tested or resolved from this present work.

The reviewer has a fair point in noticing that the model is somewhat speculative with respect to the independence of the individual domains of UPF3B. In fact, we spent quite some time testing central aspects on which the model was based.

First, we asked whether the UPF3B domains function independently of one another. To this end, we have generated cell lines expressing two different double mutants of UPF3B. If the domains are independent, two inactive double mutants should complement each other and result in a (better) rescue. However, we have been unable to observe a rescue for any combination of UPF3B mutants. We conclude that this aspect of our original model cannot be confirmed, although there are technical or biological explanations for this negative result.

Furthermore, we also tested the effects of the mutations on the various interactions of UPF3B by IP. As detailed below, the mutants behaved as expected. In addition, we analyzed the interactome of UPF3B and its mutants by TurboID proximity ligation to identify transient interactions. This approach independently confirmed the "correct" behavior of the mutants and identified interesting new interactions of the UPF3B middle domain, which could explain its importance for NMD.

Since one aspect of the original model (independence of domains) could not be validated, we have now included a revised, less speculative model (Figure 8).

Specific concerns:

UPF3B mutant alleles purported to disrupt interactions with UPF2 or EJC, are critical reagents in this study and need to be experimentally validated (by coIP, for example) in this cell system.

We agree with the reviewer that these are critical reagents that require experimental validation.

Please note that the Del-EJC mutant has been initially characterized in Gehring et al. 2003. (doi.org/10.1016/S1097-2765(03)00142-4) and the Del-UPF2 mutant in Boehm et al. 2014 (doi.org/10.1016/j.celrep.2014.09.012). Since they have not been validated together in one experiment, we now show IPs of these mutants and the Del-middle domain mutant in Figure 6E. These IPs confirm that the binding to the desired interaction partners was strongly reduced in the mutants.

Furthermore, we carried out TurboID-based proximity labeling of proteins interacting transiently with UPF3B and its mutants. Also in these TurboID Experiment we observed a strong decrease in proximity labeling of the respective interaction partners (Figure EV5F-J).

Additionally, these mutants should be further evaluated using the robust PTC-containing β-globin reporter assay shown in Fig. 4E.

We agree that the PTC-containing β-globin reporter assay is a well established and robust system to study NMD activity. The characterization of the mutants using the β-globin reporter (Figure EV5A-C) shows that the turnover of β-globin PTC39 is rescued by all three mutants. This finding is in good agreement with the qPCR analysis of endogenous NMD targets. In fact, the β-globin reporter seems – if anything – to be less sensitive, which might be due to the lower sensitivity of the Northern blot or to the very robust degradation of the reporter.

The authors should provide longer exposures of western blots presented in Fig. 1B, 2A, and 3A to enable clear evaluation of UPF3A levels in over-expression and knock-down cell lines.

We have now included exposures with higher contrast for UPF3A in these figures.

Additionally, the authors should comment on the large differences in gene expression changes observed for the different UPF3A KO clones.

This is an interesting point that we would like to explain in more detail. As we pointed out in the manuscript, most of these differences appear to be "clone-specific". The parental HEK 293 cells can be regarded as a pool of cells with slightly different, yet stable genomes (DOI: 10.1038/ncomms5767). Hence, it is possible that individual clones show different gene expression patterns due to their origin from a single clonal cell with a specific genome (DOI: 10.1038/ncomms5767). We assume that any phenotype of clones may be caused by the knocked-out gene, or may be caused by a genomic difference between the new line and its parental line. Therefore, we use at least two different clones for our analyses. In our view the overlap in gene expression changes of two clones represents clone-independent changes, which are caused by the knockout.

A gene ontology analysis of the UPF3A KO cell lines shows that the biological process with the highest significance is "regulation of signaling" in clone 14 and "response to glucocorticoid" in clone 20. However, these effects are probably not a result of the UPF3A KO.

It is unclear why the UPF3A/B double KO cells see additional changes in gene expression upon additional siRNA-mediated depletion of UPF3B. The authors indicate that UPF3B is still present in the double 'knockout' cells, but if true, then it is inaccurate to refer to these cell lines as knock-outs.

This point also seems to require clarification. According to the method by which the cells were generated and also based on the genomic and transcriptomic characterization, the UPF3B gene was knocked out in these cells. It has recently been shown that genome-edited cells are in some cases able to express a small amount of functional protein, for example by using alternative translation initiation. For the purpose of clean experiments, we decided to treat the KO cells with an additional KD in some cases. With this treatment we wanted to "eliminate as many of the potentially present remaining UPF3B proteins". However, we do not see any effects that cast doubt on the original assumption that these are KO cells in the sense of the word.

The graph below shows the <u>functional</u> effect of the additional UPF3B knockdown on eight known NMD substrates from the RNA-Seq data (UPF3A+B dKO samples marked in red; compare UPF3dKO1 with UPF3dKO1KD and UPF3dKO2 with UPF3dKO2KD). It is evident that the KD does not significantly enhance the inhibition of NMD, any observable effects are rather random and do not show a clear trend (i.e. go up and down).

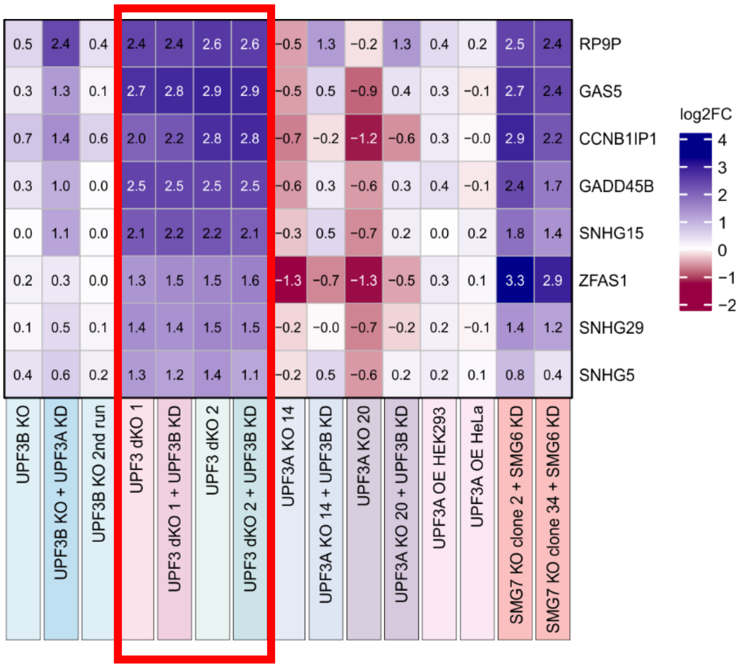

The model in Fig 1A is confusing and unnecessary. Additionally, the model for UPF3 function in NMD presented in Fig. 8 is highly speculative and not derived from data gleaned from the present study and is misleading to the reader (note that the bottom panel of 8C is particularly difficult to follow).

We agree with the reviewer that the model in Figure 1A was not optimal. But we think that a visual representation of the biological question can increase the overall understanding of the paper. Therefore, we have revised and simplified Figure 1A.

As already described above, we have completely revised the model. Part C, which was particularly criticized, can now be found in a more comprehensible form in Appendix Figure S3.

There is a general paucity of direct presentation and quantitative discussion of the data and in many instances experimental findings are over-sold. For example, it is premature to refer to UPF3A and UPF3B as either 'modular activators' of NMD (title) or 'fault-tolerant NMD activators' (abstract).

As part of rewriting the abstract, we have removed the term 'fault-tolerant NMD activators'. However, we find that the term "modular" fits very well to describe the architecture of UPF3A and 3B. With this term we are referring to the three domains of UPF3B, which are essential for the NMD when mutated in combination, but dispensable when mutated individually.

Referee #3:

Wallmeroth et al. provide an intriguing study on 2 key factors in the NMD RNA turnover pathway: UPF3A and UPF3B (which I will refer to as 3A and 3B, respectively). There is widespread interest in 3A and 3B because of their association with human disease (e.g., 3B mutations cause human intellectual disability) and the evidence implicating these two factor as key regulators/factors of the NMD pathway.

Using the immortalized human embryonic kidney cell line, HEK293, for most of their studies, the authors conduct a wide range of interesting experiments to address questions about 3A/3B. They provide evidence for the following main points: First, they show that 3B has a role but is not essential for NMD in HEK293 cells, thereby verifying past reports studying other cell types in vitro and tissues in vivo. Second, they show that 3A and 3B are partially functionally redundant at a genome-wide level, which verifies results from past studies on specific NMD target mRNAs. Third, they provide evidence that 3A does not act as a global NMD repressor in HEK293 cells, as suggested by an earlier study in several other cell types. Fourth, they provide exciting evidence that 3B does not act in NMD by the molecular mechanism previously widely accepted by the field. In particular, previous evidence had strongly suggested that 3B is a key tethering molecule that bridges the NMD factor, UPF2, with the exon-junction complex (EJC), the latter of which is a "NMD amplifier" complex bound on RNA near exon-exon junctions after RNA splicing. The authors' indicates that this "tethering model" is incorrect, which is a significant advance for the field. Fifth, they identify a novel central domain in 3B that functions in NMD, leading to a new model as to how 3B functions. Sixth, the authors demonstrated that a naturally occurring 3A isoform that lacks exon 4 (which partially overlaps with the new middle domain) lacks the ability to support NMD. Deletion of this region in 3B has the same effect. Collectively, their results from mutating different domains in 3A and 3B support an exciting model in which these 2 factors drives NMD by "exert(ing) multiple functions at different time points of NMD and in association with different complexes."

This is a focused and well-written MS that provides key information for the RNA field, as well as for those trying to understanding human diseases caused by defects in 3A/3B. The main problem with this MS revolves around the feeling that the authors want to maximize impact by drawing sweeping conclusions from sometimes limited (and perhaps biased) data analysis. In other words, the authors sometimes do not seem to acknowledge that biology is often "messy" and thus nature typically does not conform to simplistic conclusions. That said, the experiments conducted in this report are generally top notch, so with more in-depth data analysis, coupled with revised writing, this will likely become an outstanding paper of considerable interest.

(1) Abstract. The phrase "contradicting reports" is incorrect, as different approaches were used. Early studies used the tethering approach to demonstrate that 3A is weak NMD factor, whereas the most recent study knocked down and over-expressed 3A to provide evidence it is both a NMD factor and a NMD repressor. It is suggested to instead say something like: "...past reports have provided evidence that UPF3A is both a NMD repressor and activator."

We would like to thank the reviewer for pointing out that this sentence could be misinterpreted. It has been reworded and now reads: "While UPF3B has been demonstrated to support NMD, UPF3A was described either as an NMD activator or inhibitor."

(2) The authors often use the term "UPF3" instead of referring specifically to either the UPF3A or UPF3B paralog, or both. This should be rectified by referring to one or both (unless the context

dictates the use of the general term). Examples where the term "UPF3" is inappropriately used are lines 36, 92, 95, 302, 310, 429, and 432.

We agree with the reviewer that the use of "UPF3" could lead to misunderstandings. We have replaced "UPF3" with "UPF3B" (36), "UPF3A and UPF3B" (92, 95, 302, 429, 432) or "UPF3A+B" (310)

(3) The concept that NMD is partially functional in the absence of 3B is not new; several studies have demonstrated this previously, which should be acknowledged in the Introduction or Results.

We agree with the reviewer that our introduction did not provide a balanced view of the state of art with regard to the function of UPF3B and especially UPF3A. Therefore, we have revised the introduction, included more details and cited additional papers.

(4) The concept that 3A and 3B are partially functionally redundant is also not new; past studies should be cited in the Introduction or Results, and the new results from the authors put in this context.

We have addressed this point when we revised the introduction for point (3). We have also added additional references at the point where we discuss this finding in the discussion.

(5) Shum et al. The description of Shum et al. in the Introduction (line 121) and Discussion (line 371) fails to mention a key point: this paper <u>provided evidence that 3A is not only a NMD repressor but also a NMD activator</u> (its activity differs depending on biological context and transcript). This is an important point with regard to some of the studies in this MS (see below).

This comment somewhat surprised us, as we had remembered the content/main message of Shum et al. differently. To resolve this, we read Shum et al. again and summarize our main "impressions" here.

The second "Highlight" of the Shum et al. manuscript states: "UPF3A inhibits NMD, while its paralog, UPF3B, activates NMD". Furthermore, the "Summary" section points out that: "[…] we discovered that UPF3A acts primarily as a potent NMD inhibitor that stabilizes hundreds of transcripts." The closing paragraph of the introduction picks this statement up as well: "Our analysis revealed that UPF3A is actually a broadly acting NMD inhibitor."

There are only few passages in the text (e.g. in the results under "Genome-wide Impact of UPF3A" and in the second paragraph of the discussion) where UPF3A is also mentioned to be an NMD activator. The corresponding sentence in the discussion states: "Thus, we suggest that whether UPF3A serves as a NMD activator or repressor is likely to depend on context". However, in the first paragraph of the discussion the authors write "[…] while we demonstrated here that that its sister paralog, *UPF3A*, encodes a potent NMD repressor". Therefore, one can certainly not say that Shum et al. as a key point "<u>provided evidence that 3A is not only a NMD repressor but also a NMD activator</u>". This possibility was briefly shown in the genome-wide half-life analysis and "suggested" in the discussion, which in our view is different from being a key point.

What exactly have we written in our manuscript that was criticized here? In the introduction we wrote: "On the other hand, it was recently reported that loss of UPF3A results in increased transcript destabilization, and UFP3A overexpression in NMD inhibition." In the results Shum et al. write: "Indeed, we found that depletion of UPF3A destabilized multiple NMD target mRNAs, implying that UPF3A stabilizes NMD target RNAs" and "As a converse experiment, we overexpressed UPF3A and found that this impaired the magnitude of NMD (Figure S1E)". We feel that we have referred to the results of Shum et al. quite accurately.

In the discussion we wrote "Later, it was reported that in mouse cells UPF3B is an NMD activator and UPF3A is an NMD inhibitor (Shum et al., 2016).". Shum et al wrote in their introduction: "Our analysis revealed that UPF3A is actually a broadly acting NMD inhibitor. This discovery implies that UPF3A and UPF3B do not primarily work in a complementary or redundant manner as previously supposed; instead, they oppose each other, […]" There are also other passages in Shum et al. in which UPF3A and UPF3B are presented similarly. Also in this case, we believe that our text reflects quite closely what is written in Shum et al. However, we have reworded this sentence to "Later, it was reported that in mouse cells UPF3B is an NMD activator and UPF3A is mainly an NMD inhibitor, but potentially acting as an NMD activator for a small number of transcripts (Shum et al., 2016)".

(6) Line 125: they authors claimed to have "resolved the controversy about the functions of UPF3A and UPF3B..." In my opinion, the authors have provided important information on the roles of 3A and 3B in NMD, but they have not reconciled all past studies on this topic.

It is probably true that this controversy is not finally resolved with our manuscript. Therefore, we have changed the wording of the sentence, which now reads: "In this study, we provide important information about the functions of UPF3A and UPF3B in the NMD pathway using different UPF3A and UPF3B overexpression and knockout (KO) HEK293 cell lines."

(7) Results section titles. While most of the section titles are nicely short and to the point, most make definitive statements that require some modifications. For example, the first section title - "UPF3A overexpression or knockout does not affect NMD efficiency" - does not mention that this conclusion is based only on analysis in HEK2 93 cells. Furthermore, as described below in point 8, the authors have evidence that 3A DOES affect NMD in HEK293 cells, and further analysis may reveal that this is more extensive than currently described by the authors. The remaining section titles also do not indicate that their conclusions are based on studies in HEK293 cells. These titles need to be qualified or softened.

We have now carried out different confirmatory experiments in HeLa cells: overexpression of UPF3A in WT cells and RNA-Seq analysis (Figure 1B-E, EV1A-C), overexpression of UPF3A in UPF3B KO cells and qPCR analysis (Figure EV3H, I) and rescue experiments with UPF3B mutants (Figure EV5D,E). In all these cases, we did not observe fundamentally different results between HEK293 and Hela cells. We therefore believe that our results do not represent a peculiarity of HEK293 cells, but can be confirmed in other human cells.

It should also be noted that Yi et al. (co-submitted manuscript; DOI: 10.1101/2021.07.02.450872) find similar results as we do using HCT116 and HeLa cells.

(8) Evidence that 3A a NMD repressor. The authors make the claim that 3A is not a broadly-acting NMD repressor in immortalized HEK293 cells. While this is supported by the RNAseq data the authors show, they do not make much effort to determine whether 3A has ANY NMD repressor activity (e.g., that acts on specific NMD target mRNAs) in HEK293 cells. This is important, as Shum et al. 2016 previously provided evidence that 3A is a NMD repressor in several other cells types; e.g., pluripotent cells and normal fibroblasts in vitro, and normal germ cells and olfactory cells in vivo.

We agree with the reviewer that we have not intensively determined the specific UPF3A-repressor potential (e.g. if there are any NMD targets repressed by UPF3A in HEK293 cells) in the initial submission of the manuscript. We would first like to explain our reasoning for previously not having investigated this UPF3A NMD-repressing potential in more detail. Throughout our study we have generated various RNA-Seq datasets from different UPF3A, UPF3B or UPF3A+UPF3B knockout (+/- further knockdowns), as well as UPF3A overexpressing cell lines. To estimate the general

activating or repressing effect on NMD, we compared the RNA-Seq DGE and DTU analyses of these conditions to our recently generated SMG7-KO+SMG6-KD dataset, which displayed very strong NMD inactivation. In the case of knockout cell lines where different clones were available, we chose to minimize clone-specific effects by only considering consistently differently regulated targets in both clones as “true” regulated targets.

When comparing the overall individual and overlapping number of differentially expressed genes or differently used PTC+ transcripts, only the UPF3A+UPF3B double knockout cell lines showed a large number of consistently up-regulated and overlapping targets with SMG7-KO+SMG6-KD (804 targets for DGE and 169 targets for DTU; see also orange boxes in UpSet plots; see Figure 1 and Figure 2 below). In comparison to the UPF3A+UPF3B double knockout cell lines, all other conditions (UPF3A KO, UPF3A overexpression or UPF3B KO) either displayed a very low overall number of regulated targets (e.g. UPF3A overexpression and UPF3B KO) or low consistency between both clones (UPF3A KO). Reconciling the results of the three aforementioned conditions, we were not able to draw the conclusion that UPF3A is a broadly acting NMD repressor in human cells (details further discussed in the points below). Therefore, we did not further pursue the identification of individual genes or transcripts that are potentially repressed by UPF3A. We have tried to do so in this response and the results will be discussed in the points further below.

Furthermore, we would like to clarify one important point from our perspective: we do not claim that UPF3A has absolutely no NMD repressing activity at all (i.e. under all possible conditions, in all possible cell lines etc.). Our data could well be interpreted that a minimal number of genes and transcripts might be repressed by the isolated UPF3A KO (see discussion further below). However, our study rather focused on the interplay between UPF3A and UPF3B and which role these two factors play in the NMD machinery. In this light, we do not find compelling evidence that UPF3A is a broadly acting NMD repressor in human cells, but our data rather support the redundant functions of UPF3A and UPF3B as NMD activators. Again, we cannot rule out that in certain tissues under certain conditions UPF3A could act as an NMD repressor, but this is certainly not the case in HEK293 and - judging from our additional control experiments - in HeLa cells and thus would need to be investigated in another study.

We would also like to acknowledge that most of the tissues and cell types that were used in the Shum et al. 2016 study were of mouse origin, with HeLa cells being the only human cell type used (as far as we can tell). It might well be that the situation of interplay (stimulating/antagonistic functions) between UPF3A and UPF3B is different in mice versus humans.

A

B

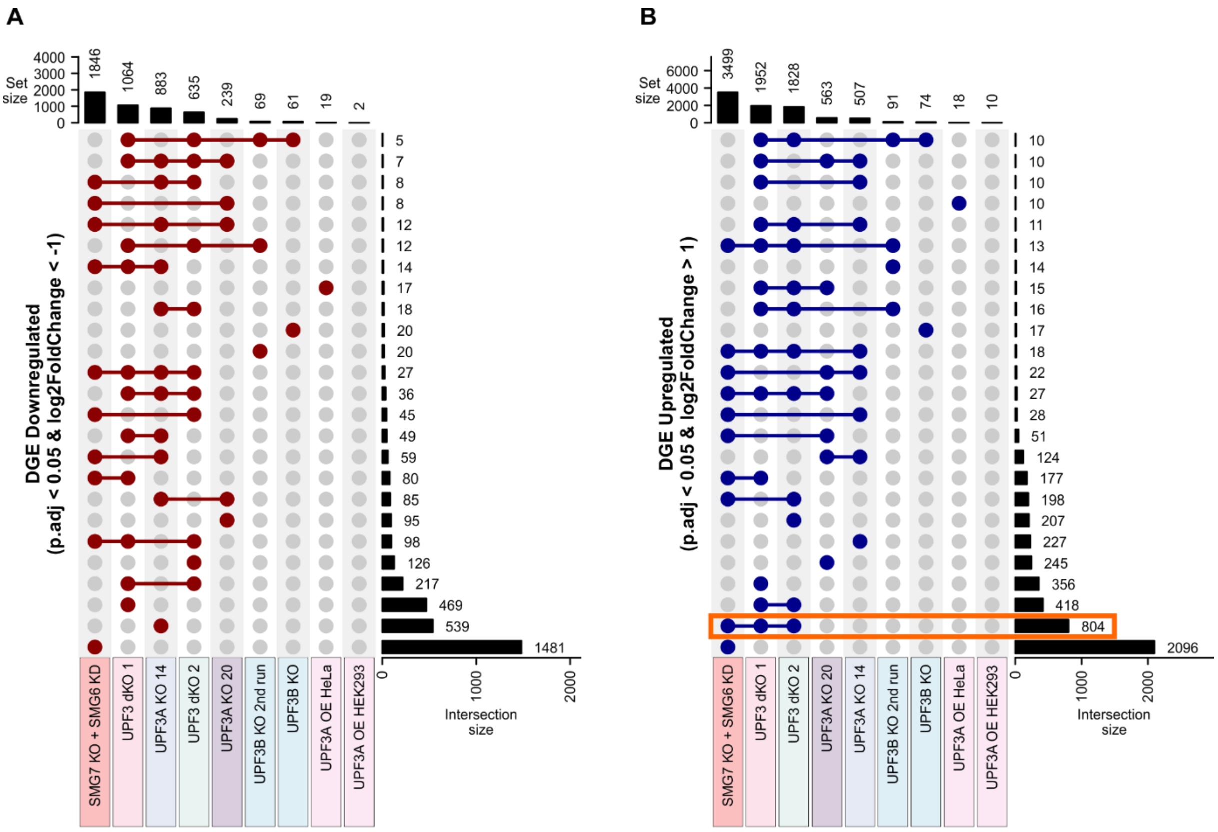

C

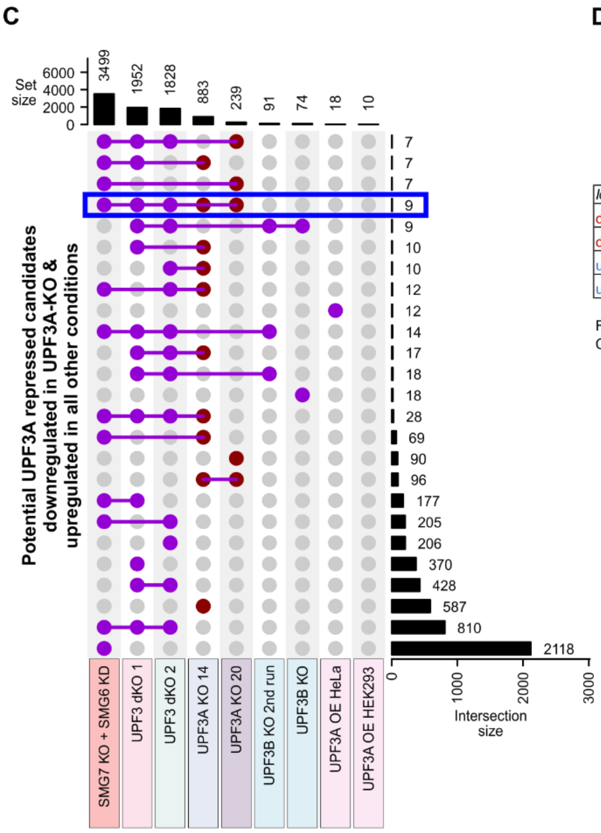

D

		UPF3A-OE-HEK293 (1 condition - no overlap)	UPF3A-OE-HeLa (1 condition - no overlap)	UPF3A-KO (overlap between clone 14 & 20)	UPF3B-KO (overlap between 2 RNA-Seq runs)	UPF3A&B-dKO (overlap between clone 1 & 2)	SMG7-KO + SMG6-KD (overlap between clone 2 & 34)
log2FC	*overlap*	*n*	*n*	*n*	*n*	*n*	*n*
down	FALSE	2	19	882	90	849	1874
down	**TRUE**	0	0	120	20	425	1846
up	FALSE	10	18	702	99	1036	1937
up	**TRUE**	0	0	184	33	1372	3499

Filter for: p.adj < 0.05
Classify based on log2FoldChange < -1 = "down" or log2FoldChange > 1 = "up"

The UpSet plots were capped at 25 intersections for the DGE analysis

Figure 1: Overlap of differential gene expression (DGE) analysis

A

B

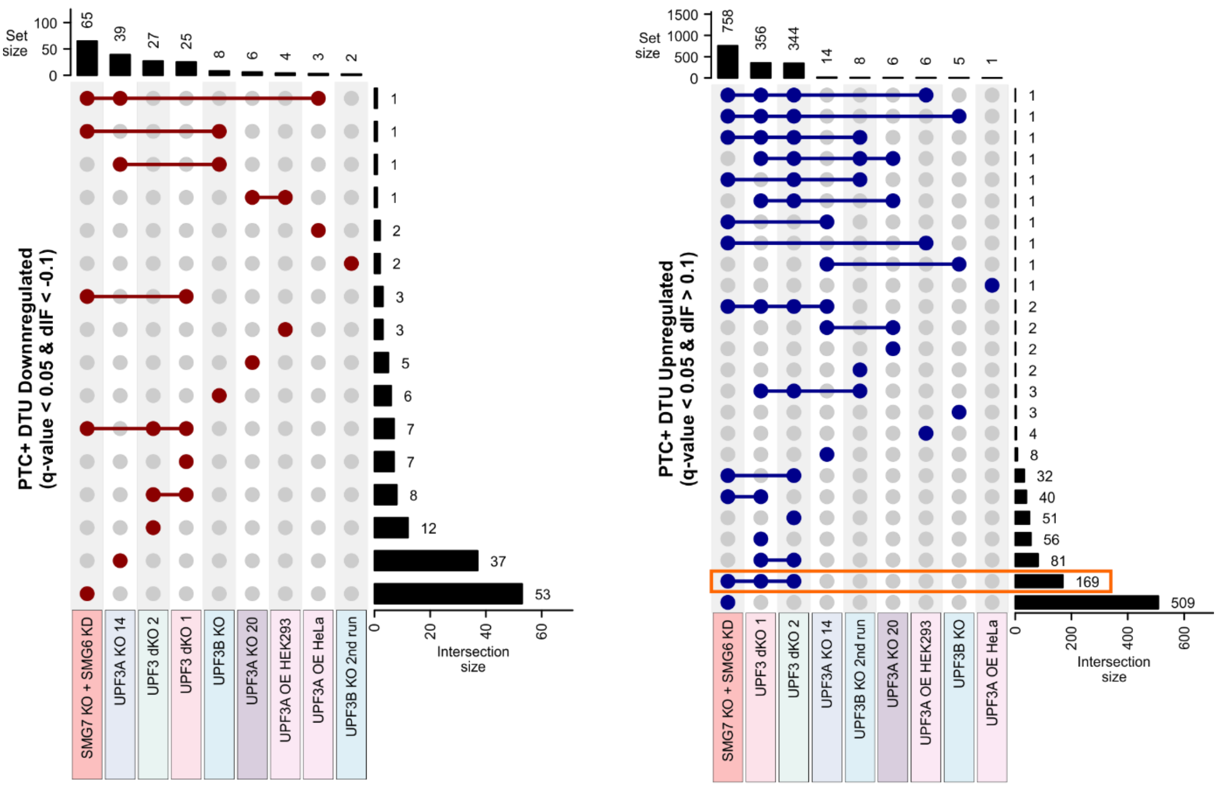

C

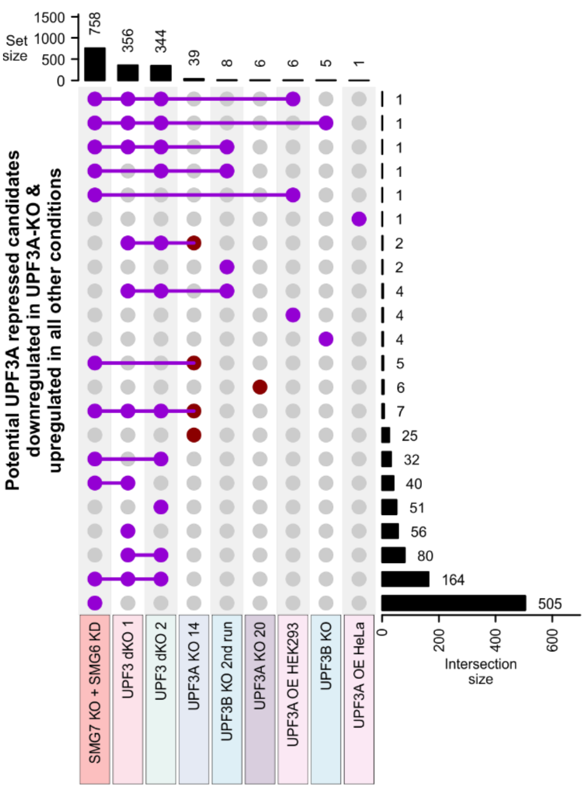

D

dIF	overlap	UPF3A-OE-HEK293 (1 condition - no overlap)	UPF3A-OE-HeLa (1 condition - no overlap)	UPF3A-KO (overlap between clone 14 & 20)	UPF3B-KO (overlap between 2 RNA-Seq runs)	UPF3A&B-dKO (overlap between clone 1 & 2)	SMG7-KO + SMG6-KD (overlap between clone 2 & 34)
		n	n	n	n	n	n
down	FALSE	4	3	45	10	22	80
down	TRUE	0	0	0	0	15	65
up	FALSE	6	1	16	13	180	341
up	TRUE	0	0	2	0	260	758

Filter for: PTC == "TRUE" & isoform_switch_q_value < 0.05
Classify based on dIF < -0.1 = "down" or dIF > 0.1 = "up"

Figure 2: Overlap of differential transcript usage (DTU) analysis

Furthermore, at least 3 lines of evidence from the authors of the present MS support that 3A might be a NMD repressor: First, they found that more mRNAs were downregulated than upregulated in response to 3A knockout (ko) (Fig. 2F).

Indeed, we found more downregulated genes (863 from clone 14 and 239 from clone 20) than upregulated genes (507 in clone 14 and 563 in clone 20) when combining the total number of regulated genes from both UPF3A KO clones (Figure 1D). However, we observed only a limited number of genes was consistently down- or upregulated in both clones (120 overlapping down-regulated vs. 184 overlapping up-regulated, Figure 1D). The low number of overlapping target genes rather indicates that clone specific effects in clones 14 and 20 are responsible for the minimal consistency. Furthermore, since more genes are consistently upregulated than downregulated upon UPF3A KO, which would rather indicate a role of UPF3A as NMD activator, we believe that the data does not support the reviewer's assumption that the high number of overall downregulated genes indicates a potential role of UPF3A as a general, broadly acting NMD repressor in human cells.

Second, Fig. EV2G shows that more PTC+ transcripts are downregulated than upregulated in response to 3B loss in one 3B-ko cell clone (while this was not observed in the other clone they generated, this at least indicates uncertainty as to 3A's role).

We believe the reviewer refers to the UPF3A KO clones (shown in Figure EV2G), which indeed displayed more downregulated PTC+ transcripts, at least in UPF3A KO clone 14 (39 downregulated vs. 14 upregulated), whereas clone 20 showed an equal distribution (6 downregulated vs. 6 upregulated). Upon closer inspection we found 7 PTC+ transcripts that are downregulated in UPF3A clone 14, as well as upregulated in SMG7KO+SMG6KD and upregulated in UPF3A+B dKO. Further 5 PTC+ transcripts are shared between UPF3A clone 14 and SMG7KO+SMG6KD (Figure 2C). From this comparison, we are left with 12 NMD-targeted transcripts that are downregulated in the UPF3A KO clone 14, whereas none of these are also found in the UPF3A KO clone 20. The lack of consistency between both UPF3A KO clones as well as the low number of potential repressed NMD targets does not seem to proof that UPF3A is indeed a broadly acting repressor in human cells.

In addition, we considered the reciprocal experiments as well, which are a) the UPF3A overexpression and b) the UPF3B KO cells. In both conditions, UPF3A is upregulated and at least in case of the UPF3B KO cells, UPF3A should be able to exert its potential repressive function without being outcompeted by UPF3B. Remarkably, none of the 12 PTC+ potentially repressed transcripts identified above are also found upregulated in UPF3A overexpression or UPF3B KO conditions. Taken together, the higher, but sill considerably low number of downregulated PTC+ transcripts in the one UPF3A clone is not a strong evidence for an NMD-inhibiting function of UPF3A, but likely represents random or clone-specific changes in gene expression.

Third, 2 of the 3 specific mRNAs the authors analyzed by qPCR were downregulated in response to 3B ko (Fig. 2E).

We assume the reviewer is referring to Fig. EV2E, in which the genes ZFAS1, AMOT, BCHE and CTSZ were monitored by qPCR upon KO and rescue of UPF3A. We agree with the reviewer that ZFAS1, AMOT, and CTSZ are indeed downregulated in the UPF3A KO clones, however we would like to point out that these seem to be UPF3A-independent effects, as the rescue (overexpression of UPF3A) did not normalize these lower expression levels. We suspect that these are again clone-specific effects that are not caused by the depletion of UPF3A protein.

The authors reported that the mRNA downregulated in both clones, ZFAS1, was not upregulated by 3A rescue, but it is not clear to what degree 3A was force expressed, as its level was not compared

with endogenous 3A in control cells. Was it insufficiently expressed? Was it overexpressed and thus not physiological? This issue is critical to assess, not only for this experiment, but for other experiments in which 3A was force expressed.

We show in Fig. EV2D the western blot from the UPF3A KO and rescue experiment, which shows that UPF3A was overexpressed (= "force expressed"). We believe that the expression levels were sufficient to exert a putative repressive function of UPF3A. Still, it is very tough to estimate how much UPF3A was overexpressed on the protein level, since the normal UPF3A levels are barely detectable when UPF3B is still present. Of note, we did not claim that these conditions are physiological, which quite frankly, is also true for the other KD, KO or rescue conditions. However, if UPF3A was a true general NMD repressor for those selected NMD targets, these expression levels should suffice to allow accumulation of the targets in the rescue conditions, which we did not observe.

The authors should also explain why these 3 particular NMD target mRNAs were chosen to be examined.

The selection of targets is now explained: "These targets represent three different classes of NMD substrates: RSRC2 mRNAs can acquire a PTC by alternative splicing, SRSF2 mRNAs can be spliced in the 3' UTR, and ZFAS1 is a non-coding snoRNA host gene containing only a short open reading frame."

Finally, to evaluate 3A in a more rigorous fashion, it is strongly suggested to perform qPCR analysis on a larger set of NMD target mRNAs chosen in an unbiased (but justified) fashion.

To identify in an unbiased manner the potentially UPF3A-repressed genes, we searched in our DGE results for an overlap of genes that:

- **Are downregulated in both UPF3A KO clones -> Evidence for targets potentially repressed by UPF3A**
- **Are upregulated in both UPF3A+B dKO clones -> Evidence for UPF3A/B-dependent NMD target**
- **Are upregulated in both SMG7 KO clones + SMG6 KD -> General evidence for NMD target**

This analysis showed that 9 genes display this particular overlap (Figure 1D, blue box). Only 3 more genes showed an overlap of "downregulated in both UPF3A KO clones" & "upregulated in both SMG7 KO clones + SMG6 KD"; of which one was barely expressed (FBN3) and another is an uncharacterized one-exon novel gene (ENSG00000271755), leaving only ANKRD37 as another potential candidate. To visualize these 12 genes in respect to expression in our RNA-Seq data, we depict their log2FoldChange from the RNA-Seq DGE data as heatmap (Error! Reference source not found.**).**

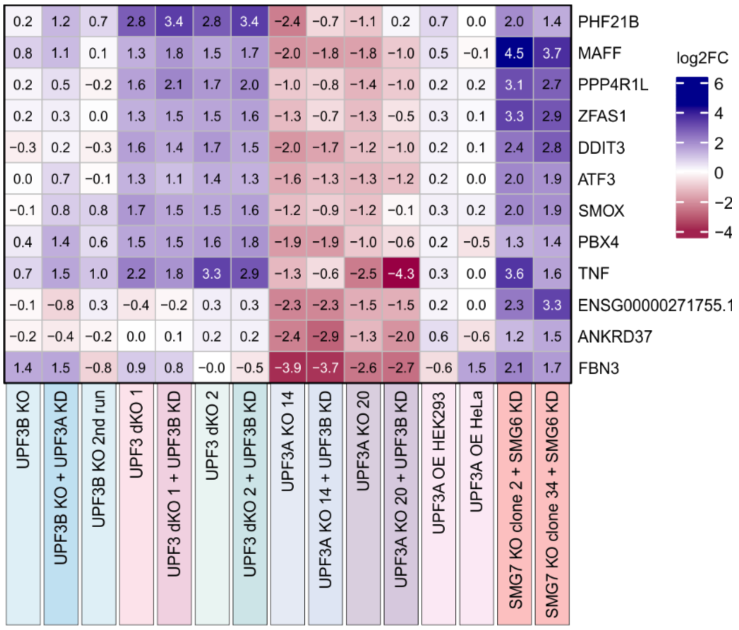

Figure 3: Heatmap of potential UPF3A-repressed genes

We randomly selected two potentially UPF3A-repressed targets for further qPCR analysis, excluding ENSG00000271755, the extremely low expressed candidates TNF and FBN3, as well as the already analyzed ZFAS1. The qPCR analysis of PHF21B in the UPF3A KO clones +/- rescue with FLAG-tagged UPF3A is now shown in addition to the already displayed targets in Figure EV2E. The also quite lowly expressed PBX4 is shown here in the response as Figure 4. Both additional targets showed no consistent UPF3A-dependent repressive effect, rather PHF21B and PBX4 seemed to be slightly downregulated in UPF3A clone 20 upon rescue with UPF3A.

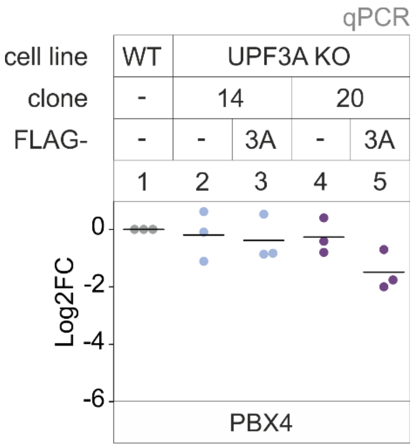

Figure 4: qPCR analysis of PBX4 as potentially repressed UPF3A-dependent gene.

(9) Examining the extent of which 3A is a NMD repressor. The authors should examine the extent at which 3A is a NMD repressor in HEK293 cells by using their existing RNAseq datasets. First, the authors should examine the nature of the PTC+ transcripts that are downregulated in 3B-ko cells and compare them with unregulated and upregulated PTC+ transcripts. Perhaps there are specific cis element or other sequence features (e.g., GC content) that correlates with NMD being repressed by 3A.

We believe the reviewer refers to PTC+ transcripts that are downregulated in UPF3A KO cells, since downregulated transcripts in UPF3B KO cells would be, if anything, an indicator for an NMD activating function of UPF3A. In total 65 PTC+ transcripts satisfied our computational cutoffs in the

DTU analysis when combining the results of both UPF3A KO clones 14 & 20. Of these, 45 transcripts were downregulated and 20 upregulated (of which 2 overlapped between clones 14 & 20). We compared those 65 transcripts with the overlapping PTC+ transcripts that are upregulated in both SMG7-KO clones + SMG6-KD (=unregulated).

We have analyzed the GC content of the transcript, the GC content of the 3' UTR, as well as the transcript, 5' UTR, CDS and 3' UTR lengths, and finally the number of exons for the three classes (3A-KO_down, 3A_KO_up and unregulated). Due to the low number of UPF3A-regulated PTC+ transcripts, all direct comparisons are not statistically significant (Figure 5, > 0.05 p-value; determined by Kolmogorov-Smirnoff test). Nevertheless, as a general trend, downregulated PTC+ transcripts upon UPF3A KO seem to exhibit:

- **Lower GC content (transcript and 3' UTR) than the upregulated or unregulated transcripts.**
- **Longer 3' UTRs and therefore longer transcripts than the upregulated or unregulated transcripts.**
- **Similar number of exons compared to unregulated.**

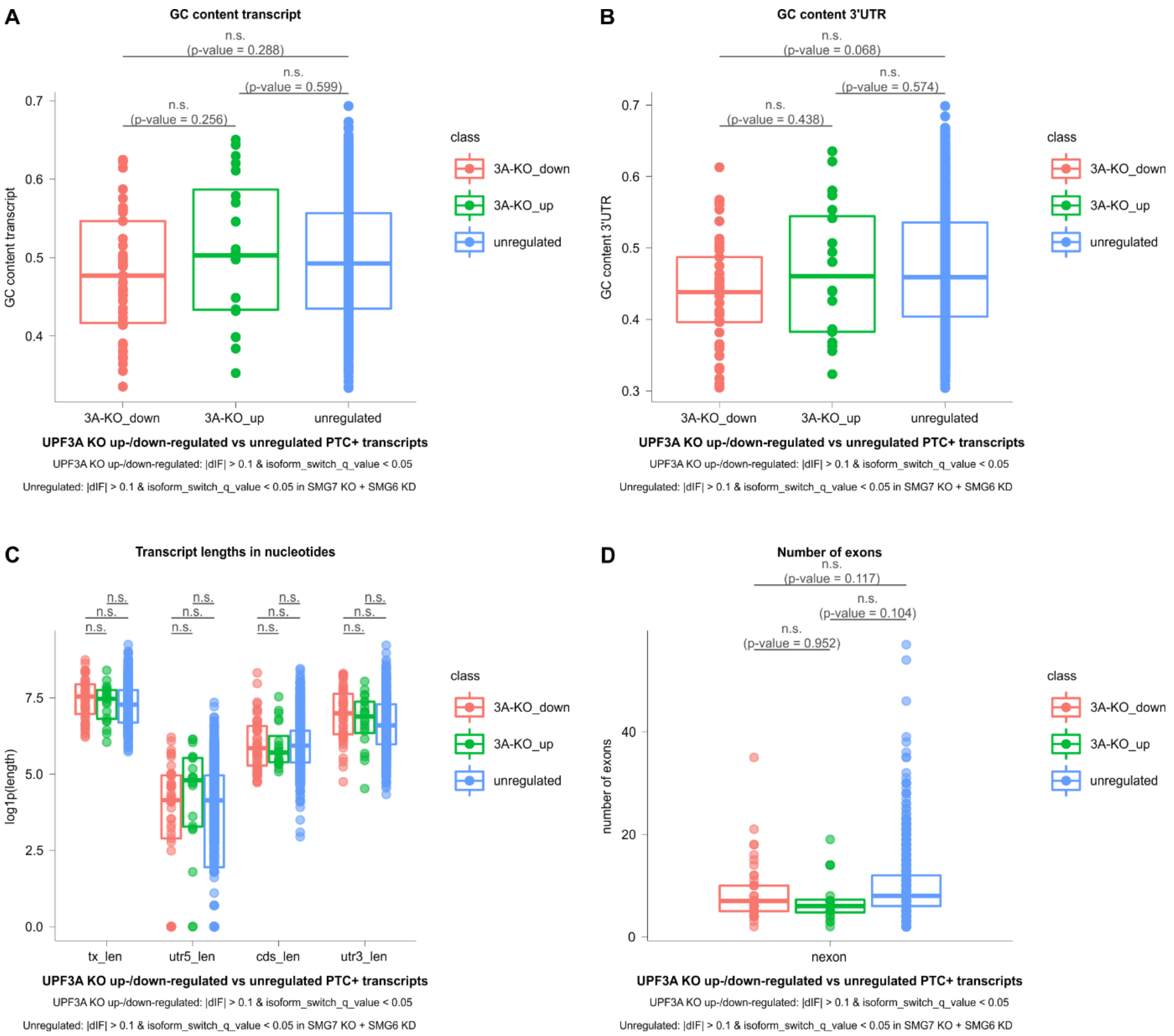

Figure 5: Sequence features of UPF3A KO regulated PTC+ transcripts

In conclusion, we do not observe striking and statistically significant differences in sequence features when comparing the UPF3A regulated PTC+ transcripts.

Second, the authors should look for overlap between the many high-confidence human NMD target mRNAs identified by other studies (e.g., based on RIPseq, RNA half-life, or pUPF1 occupancy) and mRNAs downregulated in 3B-ko cells identified by the authors. This would establish NMD target mRNAs repressed by 3A in HEK293 cells. This is also important, as not all "PTC+ transcripts" defined by the authors' current analysis are necessarily direct NMD targets; e.g., NMD silencer elements have been defined that allow NMD escape, and alternative processing events can obscure interpretation. Conversely, many of the transcripts defined as "PTC-" ARE NMD targets; e.g., many mRNAs with long 3'UTRs are degraded by NMD.

First, we again assume that the reviewer refers to the mRNAs downregulated in UPF3A KO cells, instead of UPF3B KO cells.

Second, in addition to the NMD-specific DTU analysis focusing on PTC+ transcripts, we also broadly investigated the DGE results to identify potential genes repressed by UPF3A, which do not necessarily have to rely on downstream EJCs as NMD activating signals. When overlapping the downregulated genes in both UPF3A KO clones with our SMG7 KO + SMG6 KD and/or our UPF3A+B dKO conditions, we found only the 9+3 potential targets discussed and shown above (see end of point 8). To further check whether these targets were found in previous NMD studies, we used the “meta_meta” analysis from Colombo et al. 2017, Supplemental Table S2 (doi: 10.1261/rna.059055.116.), which is based on various NMD factor knockdown + rescue (e.g. UPF1, SMG6 and SMG7). From the 12 targets, 7 were indeed found to be significant in the meta_meta analysis, with PPP4R1L, SMOX and PBX4 having the best p-values. However, only one of these 7 targets is also found in Tani et al. 2012 and Kurosaki et al. 2014, namely DDIT3. DDIT3 triggers NMD by using an uORF. We have analyzed the expression levels of DDIT3 upon UPF3A KO +/- rescue with UPF3A by qPCR and show the results in Figure EV2E. As with PHF21B and PBX4 (see point 8 above), no consistent UPF3A-dependent repressive effect could be observed, rather DDIT3 was mildly downregulated in both UPF3A clones upon rescue with UPF3A.

To visualize the magnitude of consistent DGE effects upon UPF3A KO in comparison to the UPF3A+B double knockout (dKO), we determined the overlap of the following subsets of targets with the significant (p-value < 0.05) meta_meta analysis target of Colombo et al. 2017, by plotting their meta_meta p-values:

- **“Down” in both UPF3A KO & “Up” in both SMG7 KO clones + SMG6 KD &| “Up” in both UPF3A+B dKO clones = “UPF3A KO down”**
- **“Up” in both UPF3A KO & “Up” in both SMG7 KO clones + SMG6 KD &| “Up” in both UPF3A+B dKO clones = “UPF3A KO up”**
- **As comparison, the effects of UPF3A+B dKO: Up” in both SMG7 KO clones + SMG6 KD & “Up” in both UPF3A+B dKO clones = “UPF3A+B dKO”**
- **As background: all significant (p-value < 0.05) meta_meta targets**

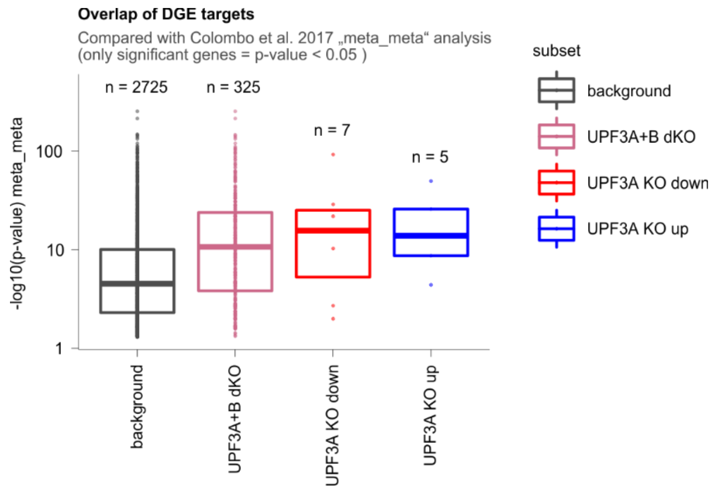

Figure 6: Overlap of DGE target subsets with the meta_meta analysis from Colombo et al. 2017

Figure 6 shows no clear trend favoring either up- or downregulated genes upon UPF3A KO considering the overlap in the meta_meta analysis. Additionally, the combined UPF3A+B dKO displays a much higher number of upregulated genes found as well in the meta_meta analysis. Collectively, we do not find evidence for a broadly repressed NMD activity upon UPF3A KO.

To further approach this question from a different angle, we used the high-confidence list of NMD targets stabilized/destabilized by UPF3A from the Shum et al. 2016 study (Table S2) and plotted the log2FoldChanges of our RNA-Seq datasets after filtering for statistically significant events (Figure 7). We assumed that plotting the DGE results for the previously described UPF3A-stabilized genes should primarily show downregulated genes upon UPF3A KO. However, we rather observed roughly equal distributions of up- and downregulated genes in the UPF3A KO conditions. Furthermore, most of those genes exhibited low log2FoldChanges (between -1 and 1), which do not indicate major changes in gene expression. Although we cannot exclude that the few more than log2FoldChange -1 downregulated genes are truly repressed by UPF3A, we would like to point out that this low number is in our opinion no evidence for the broad-acting role of UPF3A as an NMD repressor.

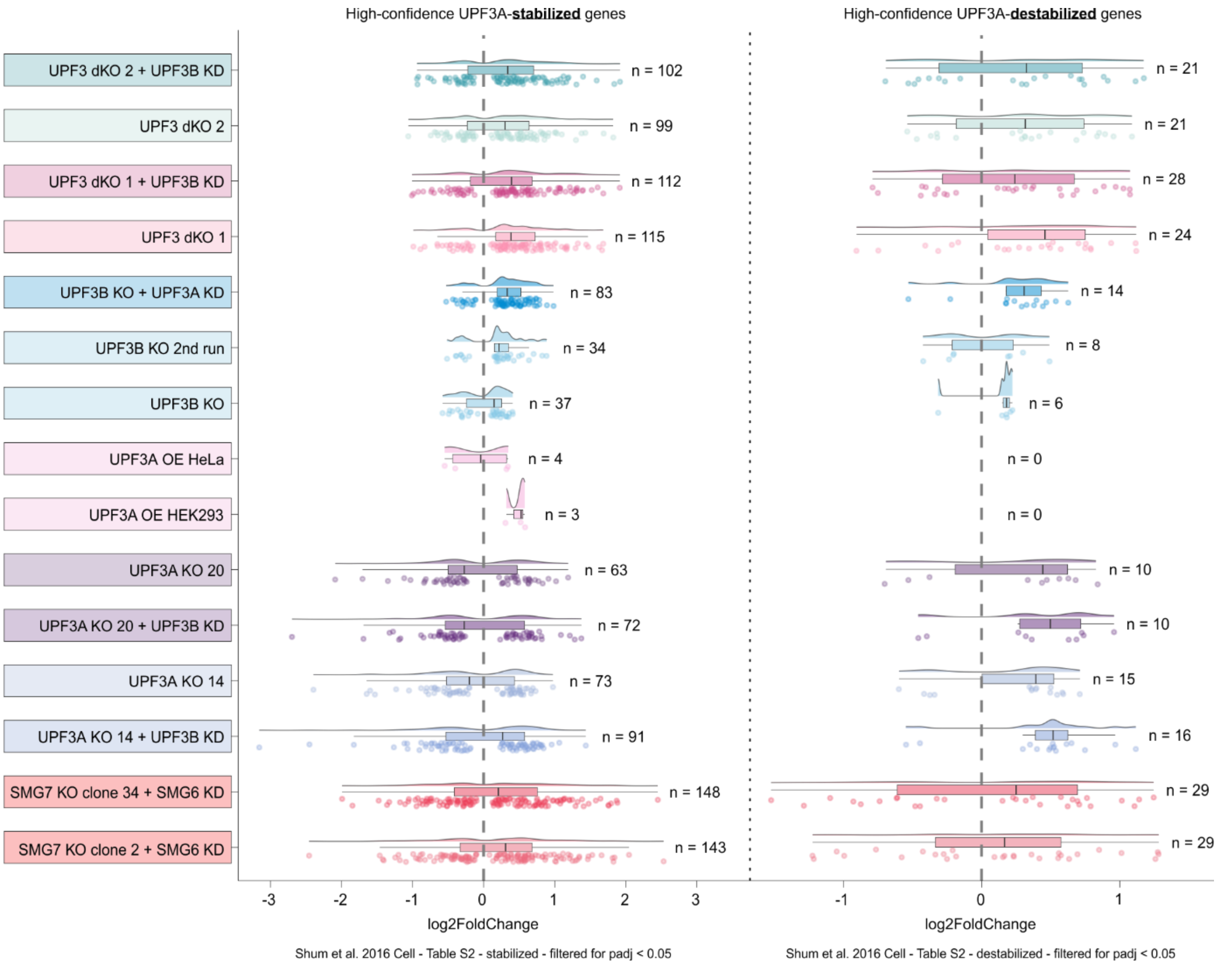

Figure 7: Raincloud plot of DGE analysis based on previously determined high-confidence UPF3A regulated genes

Third, the authors should evaluate whether 3A might have predilection for repressing the decay of transcripts with NMD-inducing features besides an exon-exon junction downstream of a stop codon, such as a long 3' UTR or an upstream ORF.

We have not specifically investigated the other NMD-inducing features (besides the EJC) in more detail, as we assumed that we need more potential UPF3A-repressed candidates to properly identify trends or specific features, such as long 3' UTRs.

The bottom line is the authors should analyze their 3A ko and control RNAseq datasets more carefully to more definitively assess whether 3A is a NMD repressor in HEK293 cells, and, if so, to what extent, and whether it has some kind of selectivity.

In conclusion, we thank the reviewer for bringing up this important issue, which was the more detailed investigation of the UPF3A-repressive potential in HEK293 cells using our existing RNA-Seq datasets. To further complement this investigation, we generated UPF3A overexpression HeLa cell lines to complement the UPF3A overexpression in HEK293 cells. Overall, we would like to again stress the point that UPF3A may have a repressive effect on a few selected targets, but we did not find – even with the excessive analyses shown above – any evidence that UPF3A is indeed a broad-acting NMD repressor in human cells, neither when knocked out nor when overexpressed.

Looking at our set of RNA-Seq data from a broader perspective, it becomes clear that the isolated KO of UPF3A or UPF3B, as well as the overexpression of UPF3A has very minor consistent effect on

NMD efficiency. The most compelling evidence for the overall rather redundant role of UPF3A and UPF3B as NMD activators comes from the double KO cells, which display clearly compromised NMD activity compared to the minor or inconsistent effects of UPF3A or UPF3B KO alone.

(10) Line 186. Where is the DTU analysis of 3A-ko cells?

Thank you for pointing this out. This analysis is shown in Figure EV2G and a reference to EV2G was added to the text.

(11) The authors tested whether 3A forced expression would inhibit NMD in the context of 3B ko (Fig. EV3H & I). They claim no negative effects, but only 3 NMD targets were selected (without any justification why these 3 were picked). Of note, SRSF2 showed a trend towards being downregulated, which was rescued by 3A.

The selection of targets is now explained within the context of the UPF3A KO cells, when they are used for the first time. "These targets represent three different classes of NMD substrates: RSRC2 mRNAs can acquire a PTC by alternative splicing, SRSF2 mRNAs can be spliced in the 3' UTR, and ZFAS1 is a non-coding snoRNA host gene containing only a short open reading frame."

We agree with this reviewer that SRSF2 showed a trend towards being downregulated in the UPF3B knockout cells and that this effect was reverted by UPF3A. However, both effects do not appear to be NMD specific. Firstly, one would expect that the UPF3B KO leads to an upregulation of NMD substrates such as SRSF2. The effect of UPF3A expression is trending in the "right" direction (i.e. if one assumes that UPF3A was an NMD inhibitor), but only when compared to UPF3B KO cells, not WT cells. The other two targets (RSRC2, ZFAS1) do not show a similar effect. However, if we compare the effects on all three targets in other experiments, we see that they tend to react similarly to NMD inhibition (e.g. Figures 3B, 4C, 6G) and only differ in the strength of the effects. Thus, we conclude that also in the UPF3B KO cells no robust NMD inhibition by UPF3A can be detected.

We would also like to point out here that UPF3B KO cells express more UPF3A than WT cells. If UPF3A was a general NMD inhibitor, NMD would be downregulated in UPF3B KO cells. Since we already analyzed the UPF3B KO cells by RNA-Seq, we refrained from analyzing also the UPF3B KO cells with UPF3A overexpression by RNA-Seq.

(12) A potential concern with the 3A- and/or 3B-ko clones generated by the authors is these cells might compensate for this loss over time (since these cell clones would have undergone many, many cell doublings, whereas transient knockdown [kd] cells would not). This compensation might eliminate some of the regulation that normally occurs. This caveat should be mentioned.

We thank the reviewer for pointing this out. Although this is theoretically conceivable, several arguments argue against this possibility. We have inserted the following sentences to address this criticism: "It is also possible that the KO cells compensate for the loss of the knocked-out gene, e.g. UPF3B, over time, which would not be the case for siRNA knockdowns. However, the strength of the effects observed in UPF3A+B KO cells clearly argues against this possibility."

(13) 3A functions independently of being a bridging factor. The authors indicated on line 290 that it was SURPRISING that the ability of UPF2 to associate with the EJC (an event considered critical to activate NMD) was not compensated for by 3A in 3B-ko cells. It is not clear why the authors considered this surprising, as it was previously established that 3A has a poorly conserved EJC-interaction domain and 3A was empirically shown to have very weak interactions with the EJC. It is suggested that the authors rephrase.

Our original wording could indeed cause confusion. Therefore, we have rephrased the text and removed "Contrary to our expectation". However, given the normal NMD activity in UPF3B KO cells, we were indeed surprised to find no bridge between UPF2 and the EJC.

(14) Lines 118 and 368. To my knowledge, the evidence from Nguyen et al. 2012 that 3A compensates for 3B is based on a comparison between 2 patients. This should either be acknowledged or this point be omitted.

We agree that in view of the few patients the data must be interpreted cautiously. Therefore, we have removed the sentences.

(15) Discussion paragraph 3. First sentence (line 379): the phrase "more or less definite answers" sounds contradictory. It is suggested to omit this sentence.

We agree that this sentence does not meet the clarity requirements of a scientific paper. Therefore, we have reformulated it: "With this work we have set out to understand the functions of the two proteins UPF3A and UPF3B in NMD."

(16) The third sentence of Discussion paragraph 3 starts with "First," but there is no "Second," etc. later in the paragraph.

We thank the reviewer for pointing this out. We did not notice this incomplete connection and removed "First".

(17) On line 384, the authors admit that the cells they used - HEK293 - may be unusual in NOT depending on 3B for NMD, but they fail to provide the evidence for this. In fact, many studies have shown that many transcripts are downregulated by 3B in other cell types (as also inferred by point 3, above). These papers and the types of cells they examined should be mentioned.

Although we agree with the reviewer that we have speculated that HEK293 cells may be unusually resistant to a UPF3B KO, we saw the same resistance in HeLa cells. In addition, there are probably as many papers reporting an effect as reporting no effect of a UPF3B knockdown. One should also keep in mind that many of these previous papers used reporter assays or microarray analyses, which are less robust than RNA-Seq. Hence, we find it rather unlikely that HEK293 and HeLa cells are the odd ones out.

(18) Line 397. It is stated that "mouse UPF3A does not appear to be a general NMD inhibitor." While this is currently supported by the authors' evidence, the way it is written gives the impression (particularly in the context of earlier parts of this MS) that it was previously shown that 3A is only a NMD inhibitor. This is not the case, as Shum et al. 2016 provided evidence that 3A is also a NMD activator, as indicated in point 5, above. Thus, it is suggested to change the wording on line 397 to say something like: "...verify that mouse UPF3A is not a general NMD inhibitor (Shum et al. 2016).

Since there are new data and not obtained in our own experiments, we decided not to discuss them. Therefore, we have removed the corresponding sentence.

(19) Line 403. It is suggested to revise to something such as: "As expected,...."

We agree that this sentence should be phrased better. We have adopted the reviewer's suggestion to write: " As expected, we observed that in the absence of UPF3A and UPF3B, the interaction between UPF2 and the EJC is lost."

(20) Line 416. This model supported by the authors' data is quite intriguing. It is suggested to start a paragraph with this model and then briefly discuss the evidence in support of and against it, as well as brief future directions.

Since the original model was criticized by Reviewer 2, we have completely revised it and incorporated new aspects that emerged during the revision into the text and figure. We hope that this reviewer will continue to find the model interesting.

(21) Discussion point? Gene duplication is an extremely common event during evolution, but only rarely does the duplicated gene copy persist, as there needs to be sufficient selection pressure to allow it to be fixed in the population. UPF3 duplicated at the dawn of the vertebrate lineage (approximately 500 million years ago) and both copies (3A and 3B) still remain today in virtually all vertebrates. It is not surprising that 3B has persisted, as it is a well-established NMD factor. But what about 3A? It is highly unlikely that it has persisted because it serves as a back-up factor for 3B. While there is little doubt (based on the authors' evidence) that this is ONE function of 3A, it is hard to imagine how this back-up function was selected for, as evolution does not anticipate potentially useful functions; instead it acts on functions that are of value in the present. Thus, presumably 3A has another function that has allowed it to persist for 500 million years. The ability to negatively regulate NMD (act as a volume control) is a good candidate to be such a function. A natural situation in which 3B and 3A levels are both highly regulated is male germ cells as they progress through meiosis (Shum et al. 2016).

We agree with the reviewer that the preservation of two redundant gene paralogues over 500 million years is surprising. It is conceivable that UPF3A has assumed additional functions compared to UPF3B. For example, it was recently postulated that UPF3A is involved in the genetic compensation response (DOI: 10.1038/s41586-019-1057-y).

However, one cannot completely exclude the possibility that functionally identical homologues are preserved evolutionarily over a long time. For example, there are two MAGOH homologues in mammals that presumably perform the same function. However, the MAGOH genes seem to be transcriptionally regulated in a different way, which suggests that they are active in different cell types or at different developmental stages. Whether this could also apply to UPF3A and UPF3B remains to be investigated.

Thank you for submitting the revised version of your manuscript. We have now received reports from the three initial referees (see comments below). While referee #1 finds that her/his comments have been satisfactorily resolved, referees #2 and #3 still have a number of concerns that should be addressed in an exceptional second round of revision. Therefore, please revise the text accordingly and expand the discussion as appropriate. Please also provide a detailed point-by-point response to each of the comments. In addition, in this revised version, please also address a number of editorial issues that are listed in detail below. Please make all edits in the manuscript file the data editors have added their notes to using the "track changes" option (please see below).

Referee #1:

The new manuscript reads well and is strengthened by the additional experiments. The authors addressed my concerns (which were few and minor to start with) satisfactorily. I have no further comments and strongly recommend publication of this manuscript.

Referee #2:

UPF3A and UPF3B are redundant and modular activators of nonsense-mediated mRNA decay in human cells.

Wallmeroth et al.

This revised manuscript by Wallmeroth and colleagues addresses many of the concerns raised by this reviewer and is an important contribution to the field. Notwithstanding, there are still a number of clarifications that the authors should consider addressing to increase the clarity and impact of the work.

1. Some indication of the level of overexpression of UPF3A in Fig. 1 would be helpful. It is clear that it is significantly elevated as compared to endogenous UPF3A (but by how much?), but how does this overexpressed level compare to endogenous UPF3B?
2. Data is still presented in a descriptive manner. For example, line 219 - what is a 'strong upregulation of UPF3A after...'? The western blots should be quantified and the level of change in protein abundance reported.
3. Discussions related to the previously observed interaction between UPF3B and eRF3a need to indicate that this interaction was detected in vitro (and in the absence of UPF1 or UPF2), particularly in light of the newly included data that eRF3a was not found to be enriched in PL experiments using UPF3B-TurboID fusions (i.e. no detectible in vivo interaction).
4. Lines 379-381 (and 391) - it is not appropriate to ascribe an 'essential' function (albeit unknown) based on negative data. As the authors are surely aware, UPF3 proteins lacking exon 4 might be inactive for a myriad of reasons - including, for example, protein folding.
5. The concept of transient interactions (Line 459) between UPF proteins to elicit NMD is not supported by the data. PL experiments indicate that all of the UPF3 mutant constructs which continue to function in NMD maintain a strong interaction with UPF1.
6. 'Synthetic lethality' is a genetic term with clear implications and should not be used to describe the concept that UPF3 may have two or more domains required for NMD.

Referee #3:

Wallmeroth et al. have responded well to many of the Revs comments. They also provide considerable new data. However, despite revised writing, considerable writing issues remain. A major problem is the authors still do not adequately cover some critical existing literature and they do not put enough effort into discussing their results in the context of this literature. We are in agreement with Rev 2 that "in many instances experimental findings are over-sold."

(1) Introduction. The paragraphs discussing the UPF3 paralogs do not properly set up the authors' findings. They are incomplete, seemingly incorrect in some places, and poorly written. We consider this a major concern, as the UPF3 paralogs are a focal point of this MS. Line 103: The paper(s) showing that UPF3A and UPF3B are in "constant competition" (odd phrase) need to be cited. While the evidence does not need to be explained in the MS, it should be provided in a rebuttal. Line 105: the Chan et al. reference indeed shows that UPF3A protein is unstable, but the authors should cite the reference showing/suggesting that "UPF3B binds tighter to UPF2 than UPF3A"? Also, what is this evidence? Line 107: Why "on the other hand"? The phrase "to a lesser extent UPF3A" is not accurate, as the sentence is referring to using a binding domain. A molecule either uses a given binding domain or it does not. Needs to be reworded if talking about affinity differences. Lines 117-119: "unclear for a long time" is vague and not a phrase commonly used in scientific writing. Likewise for the clause: "the subject of increasing discussion." Lines 116-134: it is strongly suggested to discuss UPF3A and UPF3B in two separate paragraphs. At present, the discussion goes back and forth between the two, and sometimes both paralogsare discussed simultaneously. While there is some logic to the way it is currently written, it is extremely difficult to follow. For the paragraph on UPF3A, it should be made clear that there is no controversy (at least to our knowledge) about UPF3A being a weak NMD factor (based mainly on tethering experiments). As for the evidence that UPF3A can serve as a NMD repressor, we suggest it made clear that evidence for this was obtained in a normal pluripotent cell line, as well as several normal cell types, including: (i) normal primary cells (murine embryo fibroblasts and mouse neural stem cells), and (ii) normal cells in in vivo (olfactory sensory neurons and FACS-purified spermatocytes). For the paragraph on UPF3B, it is important to thoroughly explain what was previously known about UPF3B's role in NMD, including the identification of its targets, as this is a major topic of the authors' MS. Below are some details on this.

(2) UPF3B does not function in NMD on its own in HEK293 cells. A major claim by the authors is that loss of UPF3B in the HEK293 cell lines upregulates few if any NMD target mRNAs. This indicates that UPF3B, on its own, has little or no role in NMD, even for a subset of NMD targets, in this particular cell line. They say in their rebuttal (in response to Rev 3, point 17) that this is not surprising, as "there are probably as many papers reporting an effect as reporting no effect of a UPF3B knockdown." First, they should reference the papers reporting "no effect." The only reference they cite in this regard in the Introduction is Metze et al., which is on Drosophila NMD, and thus is not directly relevant. Second, they should reference the full gamut of papers providing evidence that UPF3B DOES function in NMD. Papers showing that disruption of UPF3B upregulates many NMD targets (implying disrupted NMD) include Huang et al. Mol Cell 2011, Nguyen et al. Mol Psych 2012, Huang et al. Mol Psych 2018, Tan et al. eLIFE 2020, and Domingo et al. HMG 2020. Most of these papers completely knocked out UPF3B. All but the earliest paper were performed using RNAseq analysis, and thus UPF3B-dependent NMD targets were identified in a relatively unbiased way. In many cases, several criteria were used to distinguish direct and indirect targets. Importantly, some of these studies were performed not only in human cell lines, but in normal mouse cells and tissues in vivo, indicative of clear physiological relevance. The co-submitted MS, Yi et al., also reported that KO of UPF3B impairs NMD; they identified many UPF3B-dependent NMD targets by performing RNAseq analysis on a UPF3B KO colorectal cell line. Thus, the authors' finding that HEK293 UPF3B KO cells do NOT have impaired NMD appears to be very unusual. This is not a criticism, as it may be that HEK293 have unusual biological properties that confer UPF3B independence, which is important to report to the field. One final note: it was not entirely clear to what extent the authors determined that NMD in HEK293 cells was impervious to UPF3B KO. The authors made several claims indicating that these cells have normal NMD, such as the "increase in NMD-sensitive transcripts could not be observed with "plain UPF3B KO cells" (line 238; should omit "plain") and "upregulation of transcripts annotated with a PTC only in the UPF3B KO cells with additionally downregulated UPF3A (Figs 3D and 3E)" (line 236). Regardless of whether the authors feel that HEK293 have entirely normal NMD or nearly normal NMD, it is important that the authors' interesting findings in UPF3B KO HEK293 cells is discussed in light of the relevant literature.

(3) UPF3B KO in HeLa cells. The authors also claim that UPF3B KO also has no effect on NMD in HeLa cells, but as far as we could determine, this was only based on qPCR analysis of 3 NMD target mRNAs (Fig. EV3I). Of note, this figure shows that both RSRC2 and ZFAS1 mRNA are both modestly upregulated, indicative of impaired NMD, but whether this is statistically significantly is not clear.

(4) Is UPF3A an NMD repressor in HeLa or HEK293 cells? We appreciate the considerable efforts made by the authors to address in this rebuttal letter. Some NMD target mRNAs are downregulated in the UPF3A KO HEK293 clones, indicative of UPPF3A normally acting as a NMD repressor, but we agree that it is difficult to draw firm conclusion.

a. Low reproducibility between HEK293 KO clones. One key reason for difficulty in drawing firm conclusions is there is exceedingly little overlap in the regulated transcripts between the two KO clones 14 and 20 (only 21% of upregulated genes overlapped; 184 of 886; Fig. 2F). Thus, there were some NMD target mRNAs downregulated in the UPF3A KO HEK293 clones, indicative of UPPF3A normally acting as a NMD repressor, but because this only occurred in one clone, not the other, it was not possible to draw conclusions. This was also a concern of Rev 2. It is critical that the authors explicitly state that this low overlap impairs determination of whether or NMD is impacted by UPF3A loss.

b. The UPF3A rescue experiment is complicated by huge level of overexpression. Another complicating factor is the authors ruled out all regulation in UPF3A KO HEK293 cells that was not reversed by a "UPF3A rescue." The problem with this is that rather than a rescue experiment, this really was an overexpression experiment, as UPF3A was massively overexpressed (most likely >>10 fold relative to WT cells; Fig. EV2D). It would not be surprising if this degree of overexpression would no longer confer regulation conferred by WT levels of UPF3A. In the absence of a titration experiment, the authors must mention this caveat. Alternatively, remove these rescue/overexpression experiments and cite all the NMD target mRNAs undergoing downregulation in UPF3A KO cells as evidence of UPF3A's NMD repressor function.

c. Is UPF3A a NMD repressor in HeLa cells? The authors found that UPF3A overexpression in HeLa cells did NOT repress NMD, whereas both Shum et al. 2016 and Yi et al. (based on beta-globin NMD reporter; co-submitted MS) found that UPF3A overexpression in HeLa cells DID repress NMD. We think that one likely explanation for the difference between the authors' findings and the other 2 studies is the very high stable overexpression of UPF3A (Fig. 1B) may have caused compensatory response in the HeLa cell line used by Wallmeroth et al., leading to normalized NMD. In contrast, Shum et al. force expressed UPF1 transiently. It is not clear to us whether Yi et al. transiently or stably overexpressed UPF1. A second possible explanation is the massive level of UPF1 achieved by the authors (appears >>10-fold above normal; Fig. 1B) greatly surpassed the optimal concentration for NMD repression. A third explanations is that different HeLa sublines were probably used by the different studies (there are probably hundreds of such lines, both purposefully generated and not). Mycoplasma infection is another explanation. The authors should provide at least some discussion of these possibilities in the Discussion.

(5) Discussion paragraph on whether UPF3A functions as a NMD repressor in different contexts. Above, we are discussing some possible reasons for why UPF3A repressor activity was not observed in HEK293 and HeLa cells. A related, but different, point is whether UPF3A has repressor activity in other cells. The authors currently deal with this point on lines 431-437, but their discussion is superficial. We suggest that at the very least, the following be discussed: (i) immortalized/malignant vs. normal cell differences and (ii) mouse vs. human differences. With regard to point (i), it should be stated that Shum et al. provided evidence that UPF3A is a NMD repressor in a wide range of cells, including (1) the P19 pluripotent cell line (which has normal properties, including the ability to be differentiated into neurons), (2) normal primary cells (murine embryo fibroblasts and mouse neural stem cells), and (3) normal cells in in vivo (olfactory sensory neurons and FACS-purified spermatocytes). In contrast, the authors' study examined UPF3A activity in malignant and immortalized cell lines (HeLa and HEK293, respectively). We feel this caveat should be explicitly stated, particularly given the authors' different results from Shum et al.

(6) xrRNA experiment (Fig. 4E). This data does not support the claim of "functional redundancy" (line 272) because the authors did not examine reporter decay products for the single KO cells (in addition to the double KO cells). This should be tested or the writing revised.

Minor concerns:

Title. The term "modular activators" is not clear. For example it could be construed as meaning that UPF3A and UPF3B are modules in a larger macromolecule. How about something like: "UPF3A and UPF3B are both NMD activators harboring redundant domains."

Abstract. While the authors' revisions improve content, there are awkward passages. Line 30: change to something like: "...support NMD, evidence suggests that UPF3A is both a NMD repressor and a weak NMD activator." Lines 36-37: this sentence does not flow from the previous sentence; some introduction to understanding UPF3B mechanism is needed. Also, it is suggested to only say that multiple mutants are required to see strong impairment of rescue; omit mentioning that single mutants had little effect (as this is implied). This allows one to omit the next sentence and have more space to introduce this experiment or make other points. Lines 39-41: this sentence is confusing. Not clear why redundant domains means that the factors would be redundant. The whole Abstract should be carefully scrutinized and re-crafted.

Line 135. It is suggested to revise to something like: "...we elucidate roles and molecularly dissect the UPF3 paralogs...."

Line 315. This section title is awkward. Consider changing to something like: "Partially redundant UPF3B domains drive NMD."

Lines 318-323. It is very difficult to follow the logic here, as written. It is suggested to simply say in a single sentence that we were not able use the dKO cells because of residual UPF3B and thus we instead elected to.... Also, combine the 2 paragraphs into one.

Line 325. Add "us" between "enabled" and "to".

Line 382. Rephrase using the term "overlap" instead of "close proximity."

Line 414. In this Discussion section, the authors indicate that "we see at most a weak inhibition of NMD in our HEK293 UPF3B KO cells". The context of this finding should be presented in terms of the many papers showing that NMD IS perturbed in response to UPF3B perturbation; i.e., complete loss or knockdown of UPF3B in human cell lines and numerous normal mouse cells and tissues (see above). While it is well-established that loss of UPF3B does not ablate the entire NMD pathway, many, many NMD target mRNAs have been shown to be upregulated/stabilized in response to loss of UPF3B.

Line 436. The experiment from Yi et al. referred to is not relevant here, as this was designed to test UPF3A's NMD activator function, not its repressor function. Thus, if the authors want to mention Yi et al's interesting finding, it should be mentioned elsewhere.

Line 503-505. We have never heard the word "disconfirmed" used before. Also, we don't think it accurately describes the results. It is suggested to completely revise the sentence in terms of what the authors showed, such as that they defined roles of the UPF3 paralogs and their functional domains.

Figs. None have their fig number labeled.

Clinical relevance. It is strongly suggested to at least briefly mention the potential clinical significance of the authors' findings. For example, it is strongly suggested to refer to the Nguyen et al. HMG 2013 paper whose analysis of >50,000 individuals showed that neuro-developmental disorders are strongly correlated with UPF3A copy number alterations. Given that mutations that UPF3B mutations have been shown to both cause intellectual disability and upregulate UPF3A, this also seems a worth a brief mention with respect to the authors' findings. A discussion of clinical relevance will likely significantly increase the impact of the authors' nice work.

General response to the reviewers – 2nd round of revisions

Once again, we very much appreciate the comments and constructive criticism of the reviewers, which overall raised important aspects that we would like to address with this second revision. The reviewer's comments are in black and our comments in this response are labelled in **bold red** font. The highlights of the second round of revision include:

- Substantially expanded introduction and discussion concerning UPF3A and UPF3B, with more focus on the relation of our results to the existing literature
- New experimental quantification of overexpressed UPF3A compared to endogenous UPF3A or UPF3B (Figures EV1A and EV1B)
- New overview figures of RNA-seq analyses (DGE, DTU, DTE) summarizing the overall numbers of differentially regulated events in all conditions (Appendix Figures S1 and S4)

Referee #2:

This revised manuscript by Wallmeroth and colleagues addresses many of the concerns raised by this reviewer and is an important contribution to the field. Notwithstanding, there are still a number of clarifications that the authors should consider addressing to increase the clarity and impact of the work.

1. Some indication of the level of overexpression of UPF3A in Fig. 1 would be helpful. It is clear that it is significantly elevated as compared to endogenous UPF3A (but by how much?), but how does this overexpressed level compare to endogenous UPF3B?

This is an interesting question which we addressed with two approaches:

1) **We performed a series of Western blot analyses including lysates from control cells or cells expressing FLAG-tagged UPF3B or UPF3A, to determine the expression levels of UPF3A in the context of UPF3B and endogenous UPF3A. Since the affinities of the UPF3A and UPF3B antibodies cannot be compared, we used the FLAG antibody to normalize these two antibodies to each other. Due to the very low abundance/absence of UPF3A in the control conditions, quantification was challenging. Nevertheless, the replicates suggested 45-205x (123x on average, Fig EV1A) FLAG-UPF3A compared to endogenous UPF3A and 5x compared to endogenous UPF3B. Our Western blot result is in good agreement with a recent publication showing that UPF3A is expressed about two orders of magnitude lower than UPF3B in HEK cells (DOI: 10.1126/science.abi6983)**
2) **As a more quantitative approach we did a whole proteome mass spec analysis of HEK293 WT and UPF3A OE cell lysates. Due to the low abundance of UPF3A in the control cells, a more specialized computational approach was necessary to determine UPF3A protein levels. The Skyline analysis of quantifier peptides revealed an average of 76x/83x higher expression of FLAG-UPF3A than endogenous UPF3A, normalized to actin or tubulin expression, respectively (Fig EV1B). But since the intensities do not directly resemble the absolute quantity of peptides, the comparison of FLAG-UPF3A to endogenous UPF3B is not possible with this approach.**

The quantified amount of FLAG-UPF3A clearly represents a significant overexpression compared to endogenous UPF3A, but is of the same order of magnitude as UPF3B. We consider the levels of FLAG-UPF3A to be sufficient to observe an NMD-inhibitory effect, whilst not raising the total amount of UPF3 in the cell to a non-physiological level.

2. Data is still presented in a descriptive manner. For example, line 219 - what is a 'strong upregulation of UPF3A after...'? The western blots should be quantified and the level of change in protein abundance reported.

We understand that the word "strong" is perceived as subjective here and removed it. Furthermore, we quantified UPF3A protein levels of the Western blot in Fig 3A, although it was technically challenging due to the low UPF3A levels in WT conditions. The two UPF3B KO clones 90 and 91 showed a 14- and 7-fold upregulation of UPF3A, respectively.

3. Discussions related to the previously observed interaction between UPF3B and eRF3a need to indicate that this interaction was detected in vitro (and in the absence of UPF1 or UPF2), particularly in light of the newly included data that eRF3a was not found to be enriched in PL experiments using UPF3B-TurboID fusions (i.e. no detectible in vivo interaction).

We thank the reviewer for this important remark. Neu-Yilik et al., showed that UPF3B and eRF3a interact *in vitro* (pulldown of purified proteins) and *in vivo* (co-immunoprecipitation of co-

expressed proteins). However, the role of the middle domain was only shown *in vitro*. We have included these details in the discussion.

4. Lines 379-381 (and 391) - it is not appropriate to ascribe an 'essential' function (albeit unknown) based on negative data. As the authors are surely aware, UPF3 proteins lacking exon 4 might be inactive for a myriad of reasons - including, for example, protein folding.

We thank the reviewer for this hint. We have now rewritten the respective sentences to reflect these limitations.

5. The concept of transient interactions (Line 459) between UPF proteins to elicit NMD is not supported by the data. PL experiments indicate that all of the UPF3 mutant constructs which continue to function in NMD maintain a strong interaction with UPF1.

The criticized statement was included in response to a suggestion by reviewer 1 in the previous round of revisions. We rewrote this part of the paragraph and mention that the interaction with UPF1 seems to the common denominator of all NMD-active UPF3B mutants

6. 'Synthetic lethality' is a genetic term with clear implications and should not be used to describe the concept that UPF3 may have two or more domains required for NMD.

We thank the reviewer for this hint. The phrase 'synthetic lethality' could indeed lead to misunderstandings and we changed it.

Referee #3:

Wallmeroth et al. have responded well to many of the Revs comments. They also provide considerable new data. However, despite revised writing, considerable writing issues remain. A major problem is the authors still do not adequately cover some critical existing literature and they do not put enough effort into discussing their results in the context of this literature. We are in agreement with Rev 2 that "in many instances experimental findings are over-sold."

We took the reviewers comments very seriously and they motivated us to rewrite and expand our introduction and discussion extensively. Please refer to our specific comments further below addressing the individual points.

(1) Introduction. The paragraphs discussing the UPF3 paralogs do not properly set up the authors' findings. They are incomplete, seemingly incorrect in some places, and poorly written. We consider this a major concern, as the UPF3 paralogs are a focal point of this MS.

Line 103: The paper(s) showing that UPF3A and UPF3B are in "constant competition" (odd phrase) need to be cited. While the evidence does not need to be explained in the MS, it should be provided in a rebuttal.

We understand that the phrase "constant competition" sounds unusual and rephrased the sentence and removed "constant". The paper providing the evidence for the competition between UPF3A and UPF3B was cited (Chan et al., 2009). It states that "these results led us to hypothesize that UPF3A and UPF3B compete for binding to UPF2. According to this 'competition' model, when UPF3B concentrations are high, most of the UPF2 molecules are occupied by UPF3B, and the 'free' UPF3A is inherently unstable."

Line 105: the Chan et al. reference indeed shows that UPF3A protein is unstable, but the authors should cite the reference showing/suggesting that "UPF3B binds tighter to UPF2 than UPF3A"? Also, what is this evidence?

It is correct that the binding affinity of UPF3A and UPF3B to UPF2 were not specifically measured. However, in steady state UPF3B is more abundant than UPF3A, which (wrongly) led us to believe that UPF3A binds weaker to UPF2. We carefully inspected the respective reference and rewrote the statement.

Line 107: Why "on the other hand"? The phrase "to a lesser extent UPF3A" is not accurate, as the sentence is referring to using a binding domain. A molecule either uses a given binding domain or it does not. Needs to be reworded if talking about affinity differences.

We reworded the sentence.

Lines 117-119: "unclear for a long time" is vague and not a phrase commonly used in scientific writing. Likewise for the clause: "the subject of increasing discussion."

We reworded the sentence.

Lines 116-134: it is strongly suggested to discuss UPF3A and UPF3B in two separate paragraphs. At present, the discussion goes back and forth between the two, and sometimes both paralogs are discussed simultaneously. While there is some logic to the way it is currently written, it is extremely difficult to follow.

We understand the concern that the reviewer raised, but we have decided to continue to introduce both UPF3 paralogs side-by-side, which also accounts for the fact that both proteins are

functionally and structurally very similar. However, we have rewritten the entire section about UPF3A and UPF3B. In combination with the additionally included information, we are confident that the introduction is now more comprehensive and easier to follow.

For the paragraph on UPF3A, it should be made clear that there is no controversy (at least to our knowledge) about UPF3A being a weak NMD factor (based mainly on tethering experiments).

We have now included that information in the introduction.

As for the evidence that UPF3A can serve as a NMD repressor, we suggest it made clear that evidence for this was obtained in a normal pluripotent cell line, as well as several normal cell types, including: (i) normal primary cells (murine embryo fibroblasts and mouse neural stem cells), and (ii) normal cells in in vivo (olfactory sensory neurons and FACS-purified spermatocytes).

We expanded the section that describes the evidence that UPF3A can serve as an NMD repressor, but we feel that it is not relevant to list all mouse cell types as our study focused on human cells.

For the paragraph on UPF3B, it is important to thoroughly explain what was previously known about UPF3B's role in NMD, including the identification of its targets, as this is a major topic of the authors' MS. Below are some details on this.

We agree with the reviewer and we have now devoted a quite large paragraph of the introduction to the role of UPF3B and the previous studies that were done to identify UPF3B-dependent targets. However, since a PubMed search with the keywords "UPF3", "UPF3A" or "UPF3B" yielded 219 papers (admittedly not all relevant to our study), which could not all be introduced in depth, we chose to focus on the relevant studies published in the last 10 years.

(2) UPF3B does not function in NMD on its own in HEK293 cells. A major claim by the authors is that loss of UPF3B in the HEK293 cell lines upregulates few if any NMD target mRNAs. This indicates that UPF3B, on its own, has little or no role in NMD, even for a subset of NMD targets, in this particular cell line. They say in their rebuttal (in response to Rev 3, point 17) that this is not surprising, as "there are probably as many papers reporting an effect as reporting no effect of a UPF3B knockdown." First, they should reference the papers reporting "no effect." The only reference they cite in this regard in the Introduction is Metze et al., which is on Drosophila NMD, and thus is not directly relevant.

It is correct that we have used the term "no effect". This would imply that not even one NMD-targeted transcript is differentially regulated e.g. in the UPF3B KO cells, which is not the case. In response, we have rewritten the statements made about our RNA-seq results. However, in comparison to the strong NMD-inhibiting conditions (UPF3 dKO or SMG7 KO + SMG6 KD) the numbers of e.g. upregulated PTC-positive transcripts are still very low. We will discuss this further below.

In addition, we now include in our introduction more papers (published in the last 10 years, as mentioned above) that show limited or mild effects of UPF3B depletion on NMD. Of note, the cited Metze et al. study (RNA. 2013 Oct;19(10):1432-48.doi: 10.1261/rna.038893.113.) is from the Mühlemann group and used siRNA-mediated knockdowns of e.g. UPF3B in HeLa cells. Therefore, we feel that it is indeed relevant to our study and we continue to cite it in our introduction.

Second, they should reference the full gamut of papers providing evidence that UPF3B DOES function in NMD. Papers showing that disruption of UPF3B upregulates many NMD targets (implying disrupted NMD) include Huang et al. Mol Cell 2011, Nguyen et al. Mol Psych 2012, Huang et al. Mol Psych 2018, Tan et al. eLIFE 2020, and Domingo et al. HMG 2020. Most of these papers completely knocked out UPF3B. All but the earliest paper were performed using RNAseq analysis, and thus

UPF3B-dependent NMD targets were identified in a relatively unbiased way. In many cases, several criteria were used to distinguish direct and indirect targets. Importantly, some of these studies were performed not only in human cell lines, but in normal mouse cells and tissues in vivo, indicative of clear physiological relevance.

First, we would like to emphasize that we do not claim that UPF3B has no function at all in NMD, but rather that UPF3A can compensate for the lack of UPF3B in our cells. Nevertheless, we now reference most of the suggested literature in our more detailed introduction or in the discussion. However, we feel it is necessary to point out a few issues with the papers that supposedly provide "evidence that UPF3B DOES function in NMD":

1) **The majority of the suggested publications used murine cell lines; only HeLa cells or immortalized lymphoblastoid cell lines (LCLs) were used as human cell types. It might well be that organism-specific differences exist and that the importance of UPF3B for NMD is higher in mice. We have now mentioned this more strongly in our manuscript (as also discussed further below).**
2) **As far as we can judge, the mentioned papers often used a less stringent cut-off for the bioinformatic analyses (e.g. adj. p-value < 0.05 and no effect size cutoff or fold change > 1.41), which could, at least to some extent, explain the differences in sheer numbers of up-/downregulated genes. Of note, in many cases these analyses detected not only many upregulated genes but also an almost as high number of downregulated genes in response to the UPF3B KO:**
    a. **320 up vs. 206 down (Nguyen et al. Mol Psych 2012)**
    b. **127 up vs. 108 down (Tan et al. eLIFE 2020)**
    c. **31% of 224 DEGs upregulated (Domingo et al. HMG 2020)**

    This raises the question of whether these upregulated genes are really NMD-specific (see also points 3 and 4 below).
3) **Further investigation of the upregulated genes revealed that in many cases only a small subset of these genes was deemed by the authors themselves as "high-confidence" NMD targets:**
    a. **"Interestingly, <u>only 15 of the [526] DEGs</u> were the same as those found to exhibit altered expression in HeLa cells depleted of individual NMD factor: […]." (Nguyen et al. Mol Psych 2012)**
    b. **"Thus, we instead compared with human genes shown to be regulated by NMD in human embryonic stem cells (upregulated in response to depletion of UPF1) and identified <u>10 genes in common with those [109 genes significantly upregulated]</u> upregulated in Upf3b-null mouse frontal cortex" (Huang et al. Mol Psych 2018)**
    c. **"Of these 82 stabilized and upregulated mRNAs, 52 have at least 1 of the 3 well-established [NMD-inducing features] NIFs, and thus we classified these 52 mRNAs as high-confidence mOSN NMD targets. […] <u>11 of these previously defined likely mouse NMD target mRNAs overlapped with the 52 high-confidence targets</u> identified in our study." (Tan et al. eLIFE 2020)**
4) **To our knowledge, these papers lack a positive control for strong NMD inhibition (like SMG7 KO + SMG6 KD or strong UPF1 downregulation) to put the results (e.g. number of upregulated genes) in a more global perspective. Without a proper reference condition, the upregulation of e.g. 100 genes (with limited overlap to known NMD targets) does not really prove substantial NMD inhibition in our view.**

The co-submitted MS, Yi et al., also reported that KO of UPF3B impairs NMD; they identified many UPF3B-dependent NMD targets by performing RNAseq analysis on a UPF3B KO colorectal cell line.

It is correct and we cannot fully explain this discrepancy without direct computational comparison of both datasets (from our and the Yi et al. study). The most likely explanation are differences in the RNA-Seq protocol, library preparation, bioinformatic analyses, parameters and computational cutoffs.

Thus, the authors' finding that HEK293 UPF3B KO cells do NOT have impaired NMD appears to be very unusual. This is not a criticism, as it may be that HEK293 have unusual biological properties that confer UPF3B independence, which is important to report to the field.

We have to strongly disagree with the reviewer concerning the statement that HEK293 cells supposedly have "unusual biological properties that confer UPF3B independence". If the basis of the statement is primarily the low number of upregulated genes in a DGE analysis, then the 74 upregulated genes in our HEK293 UPF3B KO cells are not that different from other studies (e.g. 36 genes upregulated in c.624A>G, 43 genes upregulated in c.867_868delAG and 23 genes upregulated in combined analysis; Domingo et al. HMG 2020; cutoffs: log2 fold change > 1 & padj < 0.05). Also, we do not believe that HEK293 cells are unusual, simply because we can experimentally attribute the lack of strong NMD inhibition in UPF3B KO cells to the functional redundancy of UPF3A. Thus, strong effects are only observed if both UPF3 paralogs are depleted.

One final note: it was not entirely clear to what extent the authors determined that NMD in HEK293 cells was impervious to UPF3B KO. The authors made several claims indicating that these cells have normal NMD, such as the "increase in NMD-sensitive transcripts could not be observed with "plain UPF3B KO cells" (line 238; should omit "plain") and "upregulation of transcripts annotated with a PTC only in the UPF3B KO cells with additionally downregulated UPF3A (Figs 3D and 3E)" (line 236). Regardless of whether the authors feel that HEK293 have entirely normal NMD or nearly normal NMD, it is important that the authors' interesting findings in UPF3B KO HEK293 cells is discussed in light of the relevant literature.

We thank the reviewer for this comment, as it motivated us to not only more precisely interpret our RNA-seq analyses in the Results section, but also to justify our interpretation in more depth in the Discussion section. To further support these interpretations, we have now included two new overview Appendix figures (Appendix Figs S1 and S4), which visually put the DGE, DTU and DTE results of e.g. UPF3B KO in the perspective of e.g. the UPF3 dKO cells.

(3) UPF3B KO in HeLa cells. The authors also claim that UPF3B KO also has no effect on NMD in HeLa cells, but as far as we could determine, this was only based on qPCR analysis of 3 NMD target mRNAs (Fig. EV3I). Of note, this figure shows that both RSRC2 and ZFAS1 mRNA are both modestly upregulated, indicative of impaired NMD, but whether this is statistically significantly is not clear.

We included our HeLa UPF3B KO as additional cell line next to our broadly characterized HEK293 cell lines and have selected well-established NMD targets of different classes that we use throughout the manuscript. Statistical analysis using student's t-test showed that indeed the upregulation of RSRC2 and ZFAS1 are statistically significant (p = 0.007 and 0.01, respectively). However, the effect strengths are very modest and for the third target SRSF2 we observe a downregulation, which is significant as well (p = 0.01). Due to the different directions of the changes (up and downregulation) and the weak effects in the three NMD targets, these results support our conclusion that UPF3B KO alone only mildly impairs NMD activity.

(4) Is UPF3A an NMD repressor in HeLa or HEK293 cells? We appreciate the considerable efforts made by the authors to address in this rebuttal letter. Some NMD target mRNAs are downregulated in the UPF3A KO HEK293 clones, indicative of UPPF3A normally acting as a NMD repressor, but we agree that it is difficult to draw firm conclusion.

We respectfully have to disagree with the statement of the reviewer that "NMD target mRNAs are downregulated in the UPF3A KO HEK293 clones, indicative of UPPF3A normally acting as an NMD repressor". We believe it is misleading to use the few downregulated targets in the UPF3A KO as proof that the regular role of UPF3A is to act as an NMD repressor. There are three important points here that we have to clarify:

1) **In virtually all of our RNA-Seq analyses from various NMD factor KO and/or KDs, we find PTC+ transcripts that are downregulated, albeit the number usually being much lower compared to the upregulated PTC+ transcripts (e.g. 758 upregulated vs 65 downregulated PTC+ in the overlap of both SMG7 KO + SMG6 KD conditions). Does this automatically mean that e.g. SMG6 and SMG7 are also normally acting as NMD repressors, just because we see some downregulated PTC+ transcripts?**
2) **The low numbers of downregulated PTC+ transcripts in the UPF3A KO clones clearly do not allow to draw the conclusion of a general or broad-acting role of UPF3A as an NMD repressor. This statement is especially problematic as – as mentioned above – other conditions show similar or even higher numbers of downregulated transcripts.**
3) **Even if UPF3A may act on a minimal, but selective number of NMD-targeted transcripts as an NMD repressor, how can the results of massive upregulation of PTC+ transcripts in the UPF3A-UPF3B dKO conditions (compared to UPF3A KO or UPF3B KO) be explained? This very clear effect is best explained by the functional redundancy of UPF3A and UFP3B as NMD-supporting factors. If UPF3A is truly "normally acting as a NMD repressor" as the reviewer proposes, we should rather see the opposite effect, e.g. PTC+ transcripts being downregulated in UPF3A-UPF3B dKO compared to the UPF3B KO cells.**

a. Low reproducibility between HEK293 KO clones. One key reason for difficulty in drawing firm conclusions is there is exceedingly little overlap in the regulated transcripts between the two KO clones 14 and 20 (only 21% of upregulated genes overlapped; 184 of 886; Fig. 2F). Thus, there were some NMD target mRNAs downregulated in the UPF3A KO HEK293 clones, indicative of UPPF3A normally acting as a NMD repressor, but because this only occurred in one clone, not the other, it was not possible to draw conclusions. This was also a concern of Rev 2. It is critical that the authors explicitly state that this low overlap impairs determination of whether or NMD is impacted by UPF3A loss.

We agree that the low overlap impairs the determination of whether NMD is impacted by the loss of UPF3A and we stated this in the text. Nevertheless, we believe that if the loss of UPF3A has substantial and strong impact on NMD (e.g. leading to more degradation of NMD targets), we should have observed this effect in our analyses with higher confidence. An extensive comment on this topic was already included in the response to the original concern of Reviewer #2.

b. The UPF3A rescue experiment is complicated by huge level of overexpression. Another complicating factor is the authors ruled out all regulation in UPF3A KO HEK293 cells that was not reversed by a "UPF3A rescue." The problem with this is that rather than a rescue experiment, this really was an overexpression experiment, as UPF3A was massively overexpressed (most likely >>10 fold relative to WT cells; Fig. EV2D). It would not be surprising if this degree of overexpression would no longer confer regulation conferred by WT levels of UPF3A.

We respectfully disagree with the comment that we "ruled out all regulation […]" concerning the overexpression of UPF3A in the UPF3A KO cells. In particular, according to the hypothesis that UPF3A would indeed be an NMD repressor, we should have observed strong inhibition of NMD when UPF3A is overexpressed, which we did not.

Moreover, the Western blot analysis in Fig EV1A shows that although expression of FLAG- UPF3A strongly exceeds the non-detectable protein levels of endogenous UPF3A, it is in range with endogenous UFP3B (5-fold higher). That UPF3A is indeed functional at the expressed levels is also shown in Fig 7C where the same FLAG-UPF3A construct was able to rescue the NMD-inhibitory effects of the UPF3 dKO.

In the absence of a titration experiment, the authors must mention this caveat. Alternatively, remove these rescue/overexpression experiments and cite all the NMD target mRNAs undergoing downregulation in UPF3A KO cells as evidence of UPF3A's NMD repressor function.

With all due respect, we do not believe that a titration experiment has to be performed to validate our rescue experiments. We mentioned that UPF3A is overexpressed and we do not try to describe it otherwise. We would also like to emphasize that all genetic interventions such as KO, KD or rescues are always "not physiological". We also decline the request of the reviewer to remove the entire experiment and are confident in our statement that in our cell lines UPF3A is not an NMD repressor.

c. Is UPF3A a NMD repressor in HeLa cells? The authors found that UPF3A overexpression in HeLa cells did NOT repress NMD, whereas both Shum et al. 2016 and Yi et al. (based on beta-globin NMD reporter; co-submitted MS) found that UPF3A overexpression in HeLa cells DID repress NMD. We think that one likely explanation for the difference between the authors' findings and the other 2 studies is the very high stable overexpression of UPF3A (Fig. 1B) may have caused compensatory response in the HeLa cell line used by Wallmeroth et al., leading to normalized NMD. In contrast, Shum et al. force expressed UPF1 transiently. It is not clear to us whether Yi et al. transiently or stably overexpressed UPF1.

Unfortunately, this statement is not correct, since we used stable cell lines into which inducible vectors were integrated to express UPF3A. Therefore, we do not overexpress UPF3A over a long time, but at maximum for 72 h after induction, which is in the range of typical transient transfections.

It is interesting that the reviewer mentions the Shum et al. study, where the force expression of UPF3A was apparently acceptable to do, but we are criticized for a similar approach (see point above). Rather, overexpression as consequence of expression from vectors or stably integrated constructs is a normal approach.

A second possible explanation is the massive level of UPF1 achieved by the authors (appears >>10-fold above normal; Fig. 1B) greatly surpassed the optimal concentration for NMD repression.

We do not understand the statement that UPF3A should have an optimal concentration for NMD repression. What is/was the evidence for this assumption? According to this statement, UPF3A would lose its inhibitory function when expressed at high levels. Since in Shum et al. - where the repressor activity was described - a transient transfection in HeLa cells with pCI-neo plasmids harboring strong CMV promotors was used, we expect the expression levels to exceed endogenous UPF3A levels (and potentially UPF3B levels) by far. However, we could not find western blots in the Shum et al. study that visualize the expression levels of UPF3A in respect to endogenous UPF3A or UPF3B.

A third explanations is that different HeLa sublines were probably used by the different studies (there are probably hundreds of such lines, both purposefully generated and not). Mycoplasma infection is another explanation. The authors should provide at least some discussion of these possibilities in the Discussion.

We included the possibility of different HeLa sublines being the cause for different results in the discussion. Nevertheless, our findings in HEK293 and HeLa cells substantially challenge the claim that UPF3A is a "broadly acting NMD suppressor" in human cells.

(5) Discussion paragraph on whether UPF3A functions as a NMD repressor in different contexts. Above, we are discussing some possible reasons for why UPF3A repressor activity was not observed in HEK293 and HeLa cells. A related, but different, point is whether UPF3A has repressor activity in other cells. The authors currently deal with this point on lines 431-437, but their discussion is superficial. We suggest that at the very least, the following be discussed: (i) immortalized/malignant vs. normal cell differences and (ii) mouse vs. human differences. With regard to point (i), it should be stated that Shum et al. provided evidence that UPF3A is a NMD repressor in a wide range of cells, including (1) the P19 pluripotent cell line (which has normal properties, including the ability to be differentiated into neurons), (2) normal primary cells (murine embryo fibroblasts and mouse neural stem cells), and (3) normal cells in in vivo (olfactory sensory neurons and FACS-purified spermatocytes). In contrast, the authors' study examined UPF3A activity in malignant and immortalized cell lines (HeLa and HEK293, respectively). We feel this caveat should be explicitly stated, particularly given the authors' different results from Shum et al.

We appreciate the comment of the reviewer and have in part included such a statement in our discussion. However, we feel that it is not required to mention all murine cell types that were used in the Shum et al. study. It is rather the overarching question whether NMD and the UPF3 paralogs in particular function differently in mice compared to humans. We state this remaining open question now in our discussion.

Concerning the discussion of immortalized vs normal cells, at least to our knowledge, none of the studies on human cells used "normal" cells, as lymphoblastoid cell lines are also immortalized. Therefore, it would be a distorted discussion to include this specific comparison.

(6) xrRNA experiment (Fig. 4E). This data does not support the claim of "functional redundancy" (line 272) because the authors did not examine reporter decay products for the single KO cells (in addition to the double KO cells). This should be tested or the writing revised.

We have removed the "functional redundancy" part of the sentence.

Minor concerns:

Title. The term "modular activators" is not clear. For example it could be construed as meaning that UPF3A and UPF3B are modules in a larger macromolecule. How about something like: "UPF3A and UPF3B are both NMD activators harboring redundant domains."

We rephrased the title to "Human UPF3A and UPF3B enable fault-tolerant activation of nonsense-mediated mRNA decay". In the discussion, we explain our choice of fault tolerance as a concept that describes the functional redundancy of UPF3A and UPF3B, as well as the apparent redundancy of the functional domains of UPF3B.

Abstract. While the authors' revisions improve content, there are awkward passages. Line 30: change to something like: "...support NMD, evidence suggests that UPF3A is both a NMD repressor and a weak NMD activator." Lines 36-37: this sentence does not flow from the previous sentence; some introduction to understanding UPF3B mechanism is needed. Also, it is suggested to only say that multiple mutants are required to see strong impairment of rescue; omit mentioning that single mutants had little effect (as this is implied). This allows one to omit the next sentence and have more space to introduce this experiment or make other points. Lines 39-41: this sentence is confusing. Not

clear why redundant domains means that the factors would be redundant. The whole Abstract should be carefully scrutinized and re-crafted.

We have revised the abstract.

Line 135. It is suggested to revise to something like: "...we elucidate roles and molecularly dissect the UPF3 paralogs...."

We appreciate the suggestion and changed the sentence.

Line 315. This section title is awkward. Consider changing to something like: "Partially redundant UPF3B domains drive NMD."

We have changed the section title.

Lines 318-323. It is very difficult to follow the logic here, as written. It is suggested to simply say in a single sentence that we were not able use the dKO cells because of residual UPF3B and thus we instead elected to.... Also, combine the 2 paragraphs into one.

We feel like the suggested reduction to one sentence would not properly represent the problem we faced. However, we incorporated the second suggestion.

Line 325. Add "us" between "enabled" and "to".

We corrected the sentence accordingly.

Line 382. Rephrase using the term "overlap" instead of "close proximity."

We thank the reviewer for the proposed change in wording and adapted it in the manuscript.

Line 414. In this Discussion section, the authors indicate that "we see at most a weak inhibition of NMD in our HEK293 UPF3B KO cells". The context of this finding should be presented in terms of the many papers showing that NMD IS perturbed in response to UPF3B perturbation; i.e., complete loss or knockdown of UPF3B in human cell lines and numerous normal mouse cells and tissues (see above). While it is well-established that loss of UPF3B does not ablate the entire NMD pathway, many, many NMD target mRNAs have been shown to be upregulated/stabilized in response to loss of UPF3B.

We thank the reviewer for this suggestion and integrated a new section to discuss our mild effects upon UPF3B KO in context of the relevant literature. The other issues mentioned here were addressed above.

Line 436. The experiment from Yi et al. referred to is not relevant here, as this was designed to test UPF3A's NMD activator function, not its repressor function. Thus, if the authors want to mention Yi et al's interesting finding, it should be mentioned elsewhere.

We integrated the experiment differently as indicated by the reviewer. However, the observed activating function of mUPF3A and hUPF3A in Yi *et al*'s experiment questions an NMD inhibitory function of the respective paralog, thus supporting our thesis.

Line 503-505. We have never heard the word "disconfirmed" used before. Also, we don't think it accurately describes the results. It is suggested to completely revise the sentence in terms of what the authors showed, such as that they defined roles of the UPF3 paralogs and their functional domains.

We removed the word “disconfirmed” and revised the sentence.

Figs. None have their fig number labeled.

As correctly noticed, we removed the figure labels to present the figures in “production quality”. In the published version, the labels won’t be present but rather included in the figure legends.

Clinical relevance. It is strongly suggested to at least briefly mention the potential clinical significance of the authors' findings. For example, it is strongly suggested to refer to the Nguyen et al. HMG 2013 paper whose analysis of >50,000 individuals showed that neuro-developmental disorders are strongly correlated with UPF3A copy number alterations. Given that mutations that UPF3B mutations have been shown to both cause intellectual disability and upregulate UPF3A, this also seems a worth a brief mention with respect to the authors' findings. A discussion of clinical relevance will likely significantly increase the impact of the authors' nice work.

We thank the reviewer for this constructive suggestion. However, we do not feel confident in associating copy-number alterations in UPF3A with our results about the molecular function of UPF3A and UPF3B.

2nd Revision - Editorial Decision 31st Mar 2022

Thank you again for submitting the final revised version of your manuscript and addressing the remaining points. I am pleased to inform you that we have now accepted it for publication in The EMBO Journal.

EMBO PRESS

YOU MUST COMPLETE ALL CELLS WITH A PINK BACKGROUND ➔

PLEASE NOTE THAT THIS CHECKLIST WILL BE PUBLISHED ALONGSIDE YOUR PAPER

Corresponding Author Name: Volker Böhm & Niels H. Gehring
Journal Submitted to: The EMBO Journal
Manuscript Number: EMBOJ-2021-109191

USEFUL LINKS FOR COMPLETING THIS FORM

http://www.antibodypedia.com
http://1degreebio.org
http://www.equator-network.org/reporting-guidelines/improving-bioscience-research-repor
http://grants.nih.gov/grants/olaw/olaw.htm
http://www.mrc.ac.uk/Ourresearch/Ethicsresearchguidance/Useofanimals/index.htm
http://ClinicalTrials.gov
http://www.consort-statement.org
http://www.consort-statement.org/checklists/view/32-consort/66-title
http://www.equator-network.org/reporting-guidelines/reporting-recommendations-for-tumo
http://datadryad.org
http://figshare.com
http://www.ncbi.nlm.nih.gov/gap
http://www.ebi.ac.uk/ega
http://biomodels.net/
http://biomodels.net/miriam/
http://jjj.biochem.sun.ac.za
https://osp.od.nih.gov/biosafety-biosecurity-and-emerging-biotechnology/
http://www.selectagents.gov/

Reporting Checklist For Life Sciences Articles (Rev. June 2017)

This checklist is used to ensure good reporting standards and to improve the reproducibility of published results. These guidelines are consistent with the Principles and Guidelines for Reporting Preclinical Research issued by the NIH in 2014. Please follow the journal's authorship guidelines in preparing your manuscript.

A- Figures
1. Data
The data shown in figures should satisfy the following conditions:

- ➔ the data were obtained and processed according to the field's best practice and are presented to reflect the results of the experiments in an accurate and unbiased manner.
- ➔ figure panels include only data points, measurements or observations that can be compared to each other in a scientifically meaningful way.
- ➔ graphs include clearly labeled error bars for independent experiments and sample sizes. Unless justified, error bars should not be shown for technical replicates.
- ➔ if n< 5, the individual data points from each experiment should be plotted and any statistical test employed should be justified
- ➔ Source Data should be included to report the data underlying graphs. Please follow the guidelines set out in the author ship guidelines on Data Presentation.

2. Captions
Each figure caption should contain the following information, for each panel where they are relevant:

- ➔ a specification of the experimental system investigated (eg cell line, species name).
- ➔ the assay(s) and method(s) used to carry out the reported observations and measurements
- ➔ an explicit mention of the biological and chemical entity(ies) that are being measured.
- ➔ an explicit mention of the biological and chemical entity(ies) that are altered/varied/perturbed in a controlled manner.

- ➔ the exact sample size (n) for each experimental group/condition, given as a number, not a range;
- ➔ a description of the sample collection allowing the reader to understand whether the samples represent technical or biological replicates (including how many animals, litters, cultures, etc.).
- ➔ a statement of how many times the experiment shown was independently replicated in the laboratory.
- ➔ definitions of statistical methods and measures:
  - common tests, such as t-test (please specify whether paired vs. unpaired), simple χ2 tests, Wilcoxon and Mann-Whitney tests, can be unambiguously identified by name only, but more complex techniques should be described in the methods section;
  - are tests one-sided or two-sided?
  - are there adjustments for multiple comparisons?
  - exact statistical test results, e.g., P values = x but not P values < x;
  - definition of 'center values' as median or average;
  - definition of error bars as s.d. or s.e.m.

Any descriptions too long for the figure legend should be included in the methods section and/or with the source data.

In the pink boxes below, please ensure that the answers to the following questions are reported in the manuscript itself. Every question should be answered. If the question is not relevant to your research, please write NA (non applicable). We encourage you to include a specific subsection in the methods section for statistics, reagents, animal models and human subjects.

B- Statistics and general methods

Please fill out these boxes ➔ (Do not worry if you cannot see all your text once you press return)

1.a. How was the sample size chosen to ensure adequate power to detect a pre-specified effect size?	No statistical methods were performed to predetermine sample size. The numbers of replicates (=sample size) are given in the Figure Legend for each experiment. For most experiments this sample size was three independent biological replicates, as is standard for similar molecular biology experiments. Comparable sample sizes were chosen that allow data reproducibility for each experimental conditions. For all data - if possible - positive and negative controls were included.
1.b. For animal studies, include a statement about sample size estimate even if no statistical methods were used.	NA
2. Describe inclusion/exclusion criteria if samples or animals were excluded from the analysis. Were the criteria pre-established?	No data exclusion criteria were pre-established besides default significance and effect cutoffs in analyses such as RNA-sequencing (values described in the manuscript), which are according to standards in the field. These default cutoffs were: Differential gene expression |log2 fold change|> 1 and adjusted p-value (padj) < 0.05; Differential splicing |deltapsi| > 0.1 and adjusted p-value (p.adjust) < 0.05; Differential transcript usage |dIF| > 0.1 and adjusted p-value (isoform_switch_q_value) < 0.05
3. Were any steps taken to minimize the effects of subjective bias when allocating animals/samples to treatment (e.g. randomization procedure)? If yes, please describe.	The samples were not randomized in this study, since we did not perform experiments or statistical analyses that require randomization.
For animal studies, include a statement about randomization even if no randomization was used.	NA
4.a. Were any steps taken to minimize the effects of subjective bias during group allocation or/and when assessing results (e.g. blinding of the investigator)? If yes please describe.	The investigators were not blinded during data collection because the experiments did not require blinding and were readily controlled without blinding. Investigators were not blinded during data analyses because the key findings are supported by quantitative measurements (with statistical testing where relevant) that do not rely heavily on subjective judgment for interpretation.
4.b. For animal studies, include a statement about blinding even if no blinding was done	NA
5. For every figure, are statistical tests justified as appropriate?	We only use statistical tests in high-throughput analyses (RNA-Seq, proteomics), where such tests are required to identify significant "hits". As we used only published and well accepted tools to analyze the data (e.g. DESeq2 for RNA-Seq and Perseus for proteomics), we feel that the statistical tests are justified as appropriate.
Do the data meet the assumptions of the tests (e.g., normal distribution)? Describe any methods used to assess it.	We relied on the bioinformatic tools (e.g. mentioned above) to perform the statistical analysis and did not "manually" further assess e.g. normal distribution.
Is there an estimate of variation within each group of data?	We relied on the bioinformatic tools (e.g. mentioned above) to perform the statistical analysis and did not "manually" further estimate the variation within each group of data.

Is the variance similar between the groups that are being statistically compared?	We relied on the bioinformatic tools (e.g. mentioned above) to perform the statistical analysis and did not "manually" further compare the variance between groups.

C- Reagents

6. To show that antibodies were profiled for use in the system under study (assay and species), provide a citation, catalog number and/or clone number, supplementary information or reference to an antibody validation profile. e.g., Antibodypedia (see link list at top right), 1DegreeBio (see link list at top right).	All antibodies used in this study are listed in Dataset EV7 with the catalog number and RRID (if applicable), except for three antibodies (Rabbit polyclonal anti-UPF3B, Rabbit polyclonal anti-UPF2 and Rabbit polyclonal anti-EIF4A3). The latter three antibodies were either custom-made by Eurogentech or GenScript, or were a gift from Jens Lykke-Andersen, UC San Diego. All commercially available primary antibodies were recommended or validated by the manufacturer for western blotting and human samples. All antibodies have been tested with suitable overexpression, knockdown, or knockout samples.
7. Identify the source of cell lines and report if they were recently authenticated (e.g., by STR profiling) and tested for mycoplasma contamination.	Parental Flp-In-T-REx-293 cells were purchased from Thermo Fisher Scientific; Cat# R78007; RRID:CVCL_U427. These cells were not further authenticated. The HeLa Flp-In T-REx cells were generated by Elena Dobrikova and Matthias Gromeier, Duke University Medical Center and authenticated previously in the Gehring Lab using the "Human Cell Line Authentication Services" from Eurofins Genomics, showing that these cells are indeed HeLa cells.<br>All other cell lines were derived from these two parental cell lines by either usage of the Alt-R CRISPR-Cas9 system (Integrated DNA Technologies) and/or stable integration of derivatives of pcDNA5/FRT/TO vector (Thermo Fisher Scientific) and/or PB-CuO-MCS-IRES-GFP-EF1α-CymR-Puro vector (System Biosciences). An overview of the used and generated cell lines is given in Dataset EV7. The parental cell lines were routinely tested and were free of mycoplasma contamination. Subsequent cell lines were not re-tested during the course of this study.

* for all hyperlinks, please see the table at the top right of the document

D- Animal Models

8. Report species, strain, gender, age of animals and genetic modification status where applicable. Please detail housing and husbandry conditions and the source of animals.	NA
9. For experiments involving live vertebrates, include a statement of compliance with ethical regulations and identify the committee(s) approving the experiments.	NA
10. We recommend consulting the ARRIVE guidelines (see link list at top right) (PLoS Biol. 8(6), e1000412, 2010) to ensure that other relevant aspects of animal studies are adequately reported. See author guidelines, under 'Reporting Guidelines'. See also: NIH (see link list at top right) and MRC (see link list at top right) recommendations. Please confirm compliance.	NA

E- Human Subjects

11. Identify the committee(s) approving the study protocol.	NA
12. Include a statement confirming that informed consent was obtained from all subjects and that the experiments conformed to the principles set out in the WMA Declaration of Helsinki and the Department of Health and Human Services Belmont Report.	NA
13. For publication of patient photos, include a statement confirming that consent to publish was obtained.	NA
14. Report any restrictions on the availability (and/or on the use) of human data or samples.	NA
15. Report the clinical trial registration number (at ClinicalTrials.gov or equivalent), where applicable.	NA
16. For phase II and III randomized controlled trials, please refer to the CONSORT flow diagram (see link list at top right) and submit the CONSORT checklist (see link list at top right) with your submission. See author guidelines, under 'Reporting Guidelines'. Please confirm you have submitted this list.	NA
17. For tumor marker prognostic studies, we recommend that you follow the REMARK reporting guidelines (see link list at top right). See author guidelines, under 'Reporting Guidelines'. Please confirm you have followed these guidelines.	NA

F- Data Accessibility

18: Provide a "Data Availability" section at the end of the Materials & Methods, listing the accession codes for data generated in this study and deposited in a public database (e.g. RNA-Seq data: Gene Expression Omnibus GSE39462, Proteomics data: PRIDE PXD000208 etc.) Please refer to our author guidelines for 'Data Deposition'.<br><br>Data deposition in a public repository is mandatory for:<br>a. Protein, DNA and RNA sequences<br>b. Macromolecular structures<br>c. Crystallographic data for small molecules<br>d. Functional genomics data<br>e. Proteomics and molecular interactions	We have provided a full data availability statement in the manuscript.
19. Deposition is strongly recommended for any datasets that are central and integral to the study; please consider the journal's data policy. If no structured public repository exists for a given data type, we encourage the provision of datasets in the manuscript as a Supplementary Document (see author guidelines under 'Expanded View' or in unstructured repositories such as Dryad (see link list at top right) or Figshare (see link list at top right).	NA
20. Access to human clinical and genomic datasets should be provided with as few restrictions as possible while respecting ethical obligations to the patients and relevant medical and legal issues. If practically possible and compatible with the individual consent agreement used in the study, such data should be deposited in one of the major public access-controlled repositories such as dbGAP (see link list at top right) or EGA (see link list at top right).	NA
21. Computational models that are central and integral to a study should be shared without restrictions and provided in a machine-readable form. The relevant accession numbers or links should be provided. When possible, standardized format (SBML, CellML) should be used instead of scripts (e.g. MATLAB). Authors are strongly encouraged to follow the MIRIAM guidelines (see link list at top right) and deposit their model in a public database such as Biomodels (see link list at top right) or JWS Online (see link list at top right). If computer source code is provided with the paper, it should be deposited in a public repository or included in supplementary information.	A comprehensive description of the used computer code for the RNA-Seq analysis is available at GitHub [https://github.com/boehmv/UPF3]

G- Dual use research of concern

22. Could your study fall under dual use research restrictions? Please check biosecurity documents (see link list at top right) and list of select agents and toxins (APHIS/CDC) (see link list at top right). According to our biosecurity guidelines, provide a statement only if it could.	NA
