# Supplementary material for: Human UPF3A and UPF3B enable fault‐tolerant activation of nonsense‐mediated mRNA decay
Source: EMBO J. 2022 Apr 22;41(10):e109191. doi: 10.15252/embj.2021109191 (PMC9108619; doi:10.15252/embj.2021109191)

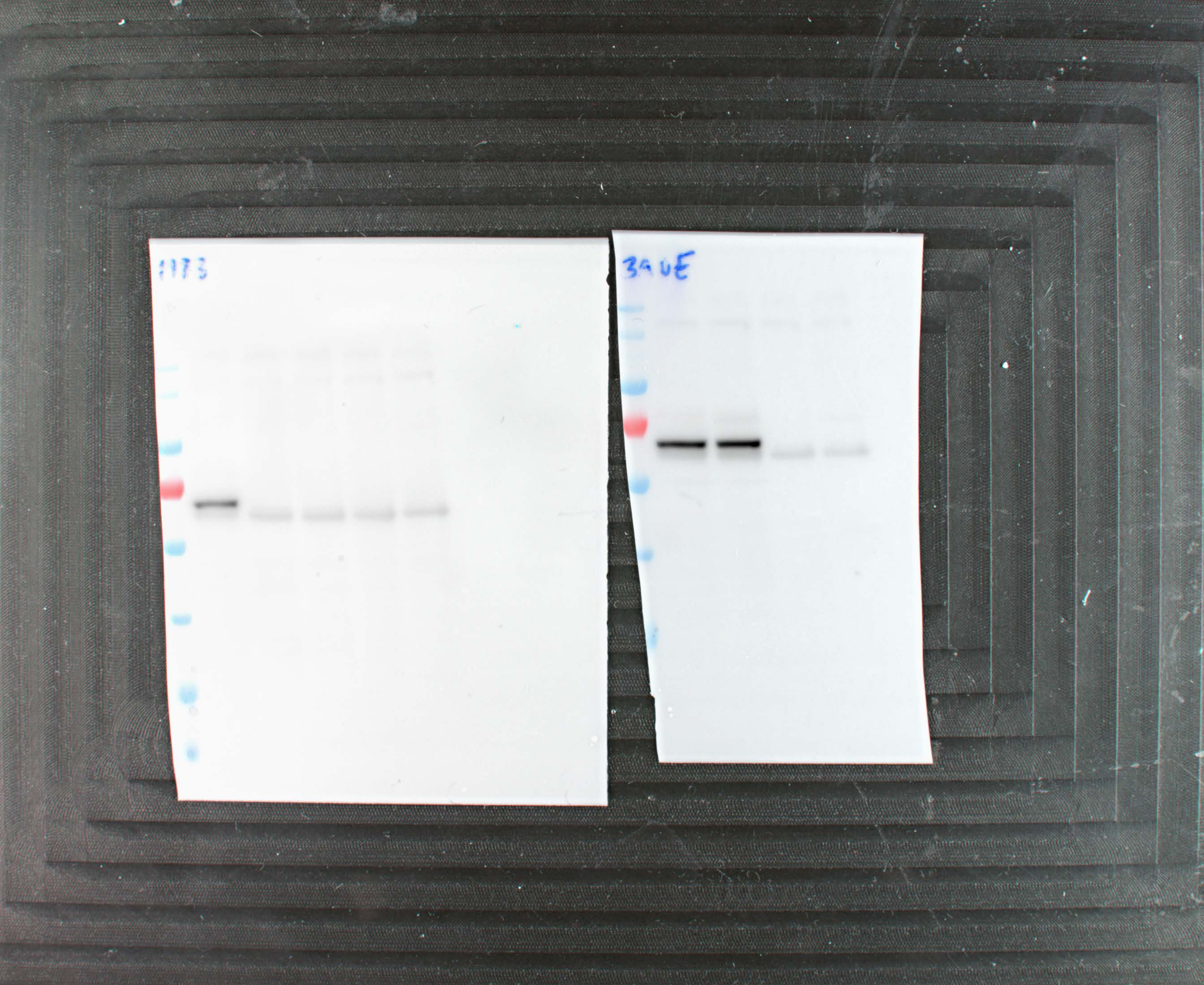
1173

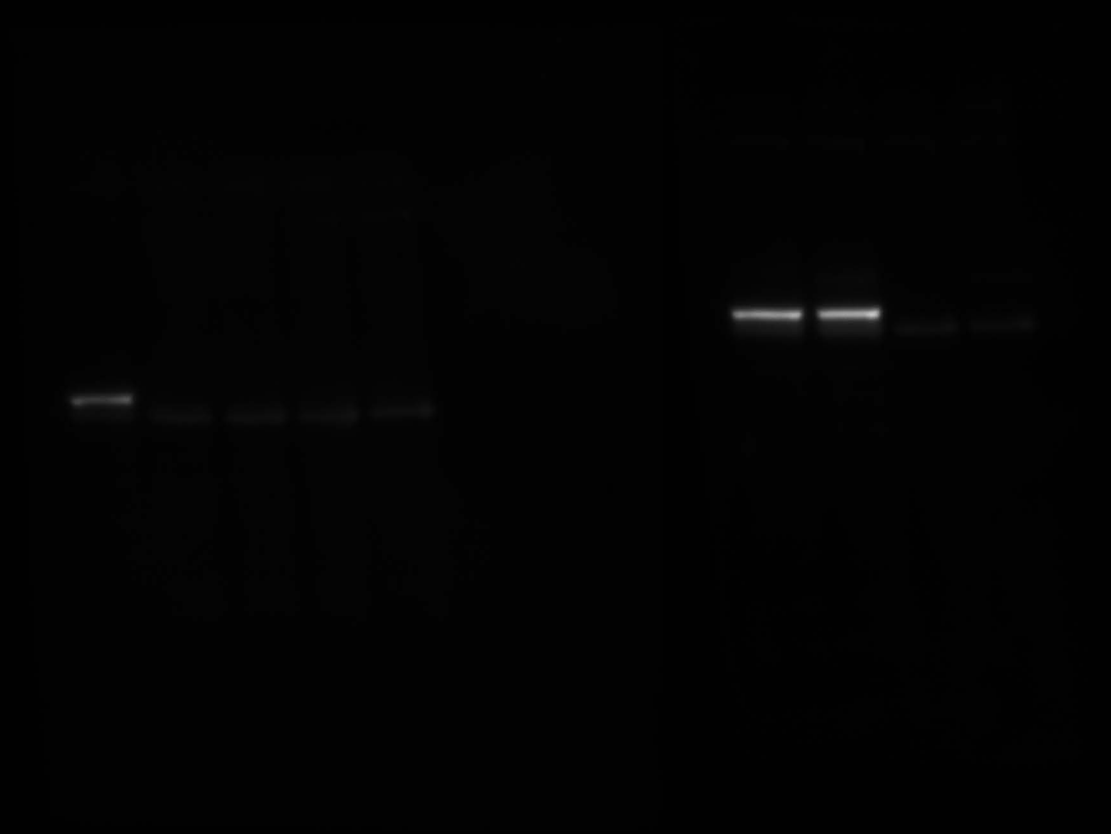

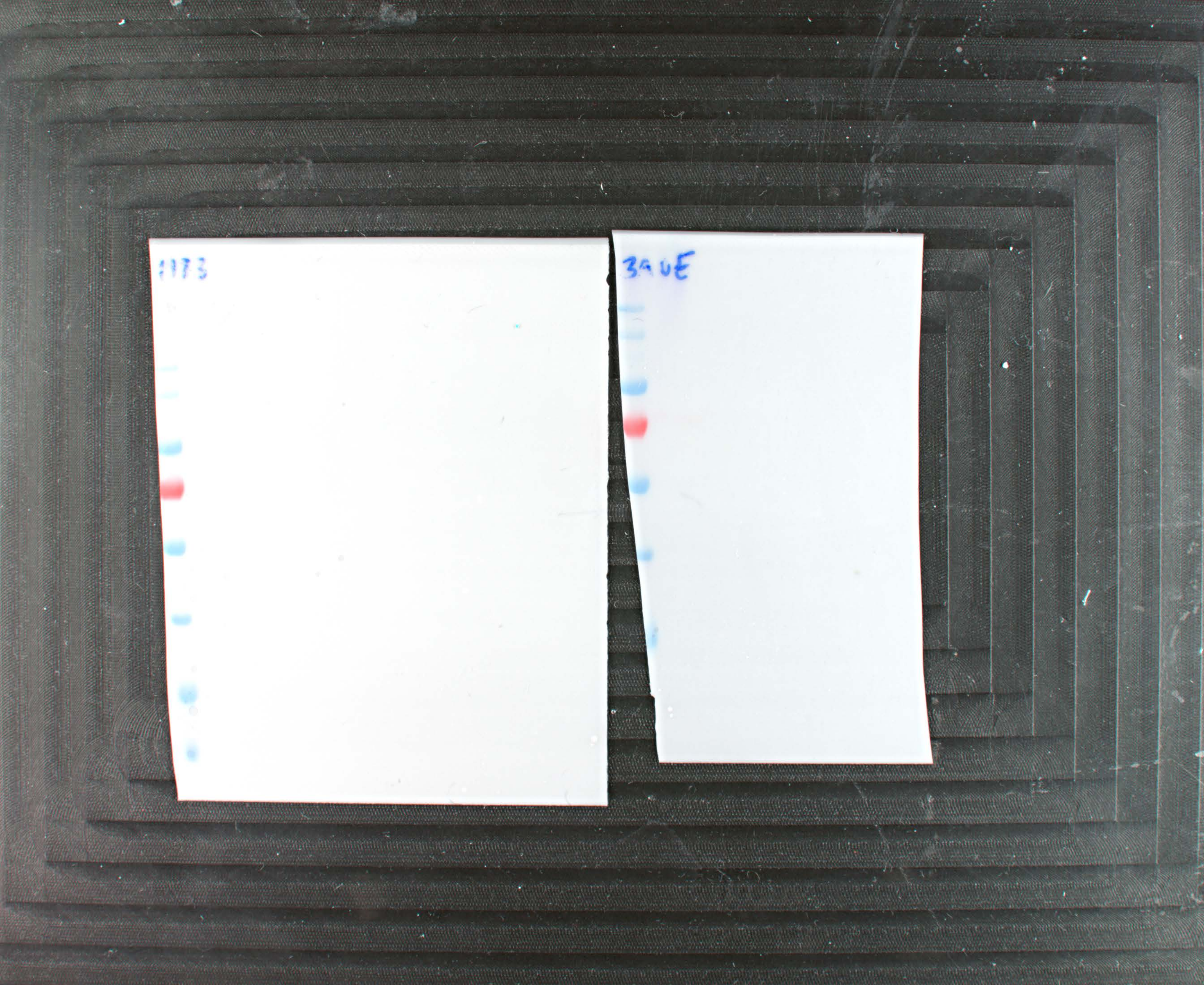

1173
39UE

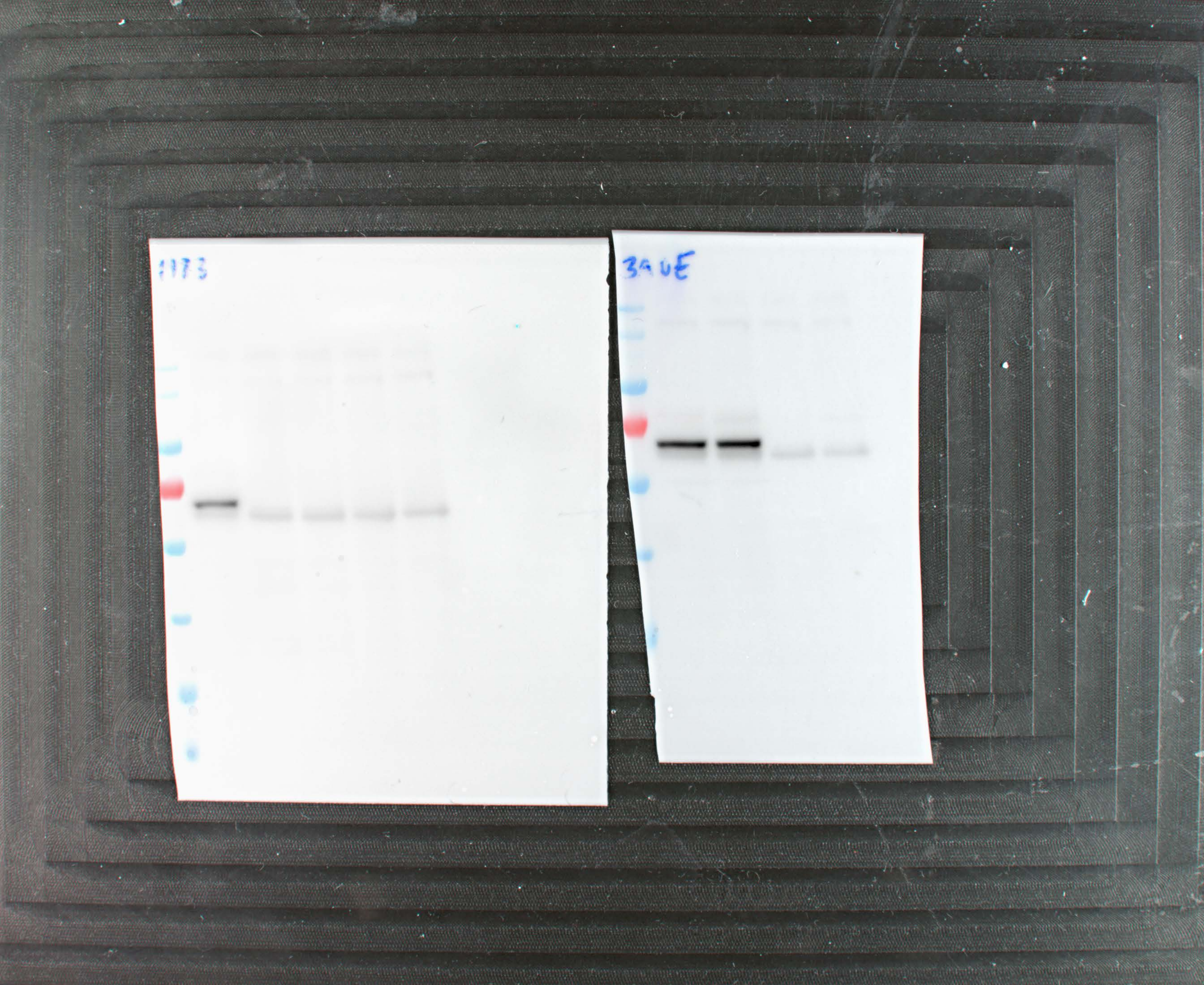

Supplement: Supplementary file 10 — Source Data for Expanded View and Appendix [file EMBJ-41-e109191-s001.zip › source_data_EVfig3.zip!/FigEV3/FigEV3H_HEK_UPF3A_rescue_UPF3B_(right blot).pdf]
