# Supplementary material for: Human UPF3A and UPF3B enable fault‐tolerant activation of nonsense‐mediated mRNA decay
Source: EMBO J. 2022 Apr 22;41(10):e109191. doi: 10.15252/embj.2021109191 (PMC9108619; doi:10.15252/embj.2021109191)

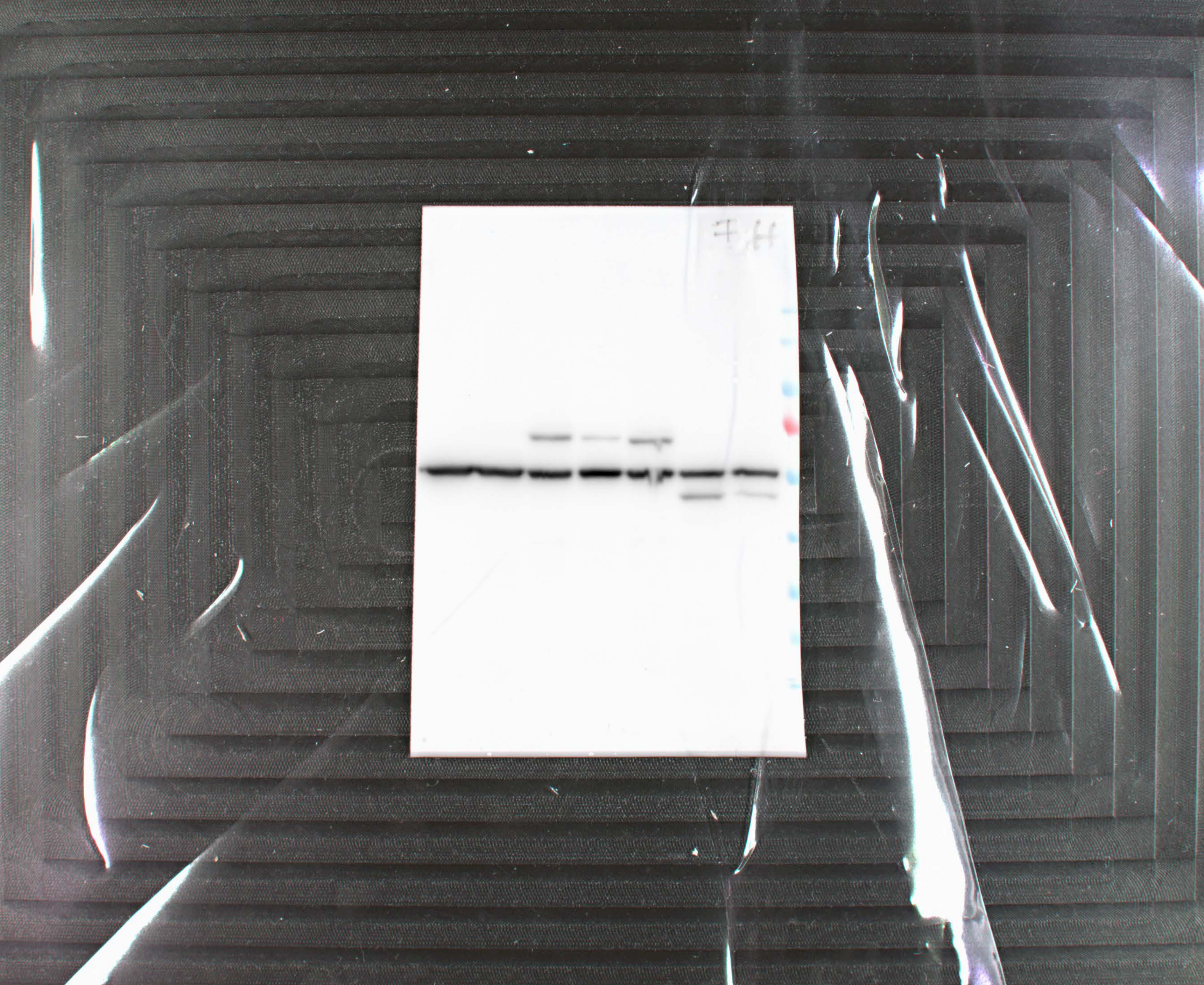

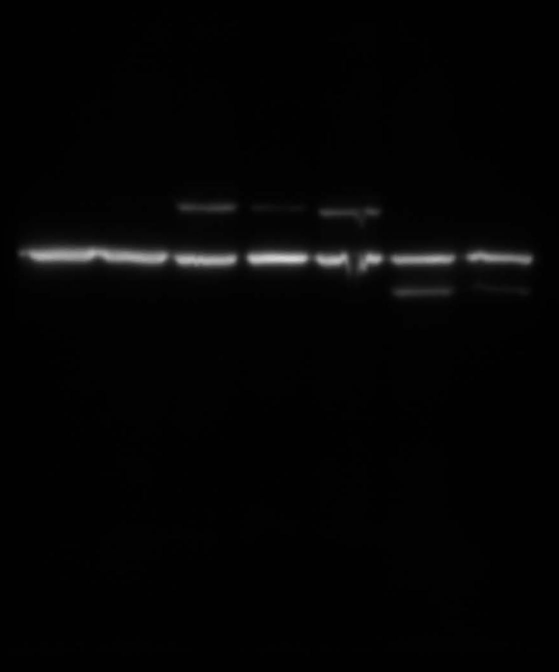

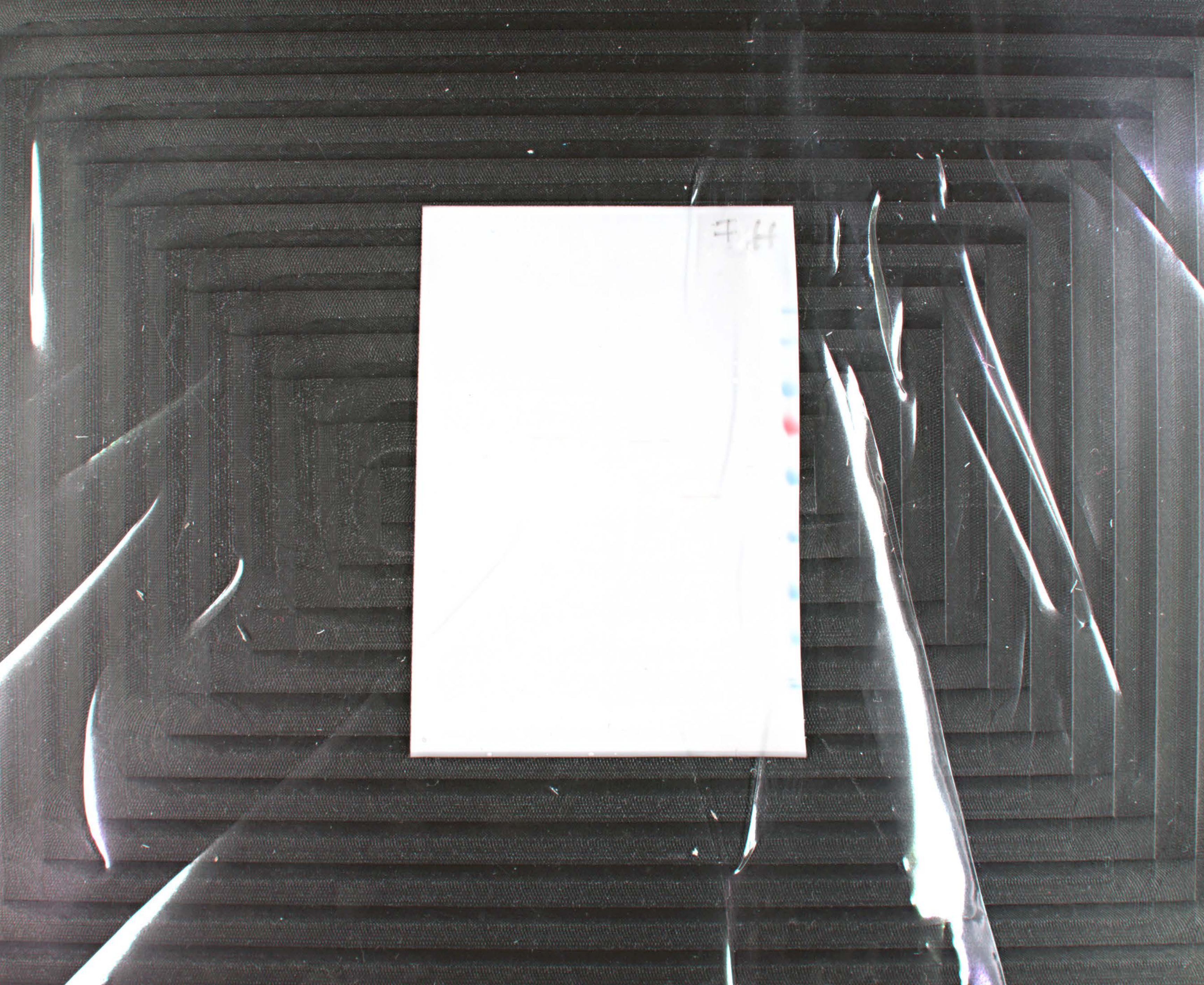

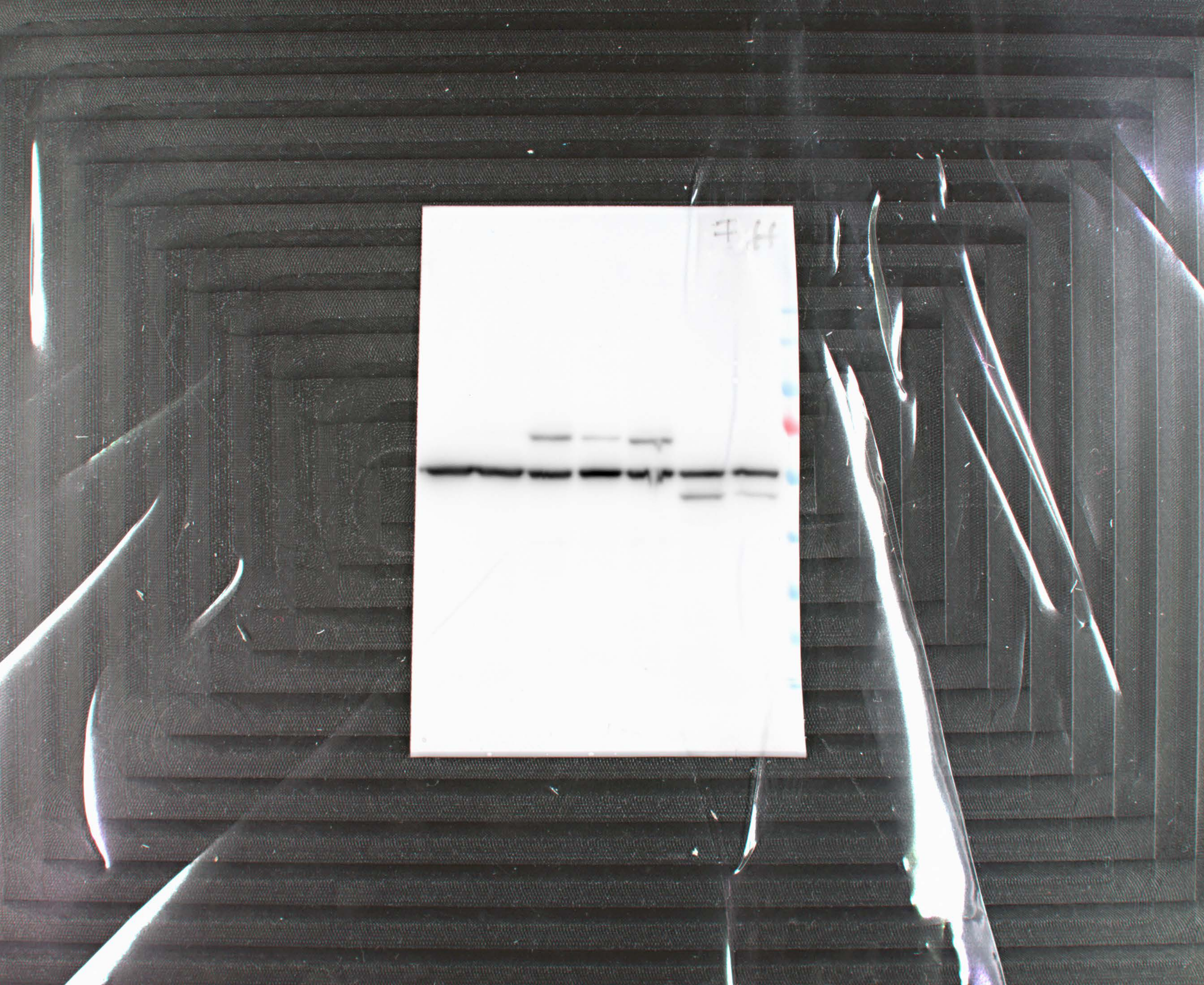

Supplement: Supplementary file 10 — Source Data for Expanded View and Appendix [file EMBJ-41-e109191-s001.zip › source_data_EVFig5.zip!/FigEV5/FigEV5D_HeLa_rescues_Tubulin.pdf]
